# Supplementary material for: Divergent synthesis of N-heterocycles via controllable cyclization of azido-diynes catalyzed by copper and gold
Source: Nat Commun. 2017 Nov 23;8:1748. doi: 10.1038/s41467-017-01853-1 (PMC5701061; doi:10.1038/s41467-017-01853-1)
Supplement: Supplementary file 1 — Supplementary Information [file 41467_2017_1853_MOESM1_ESM.pdf]

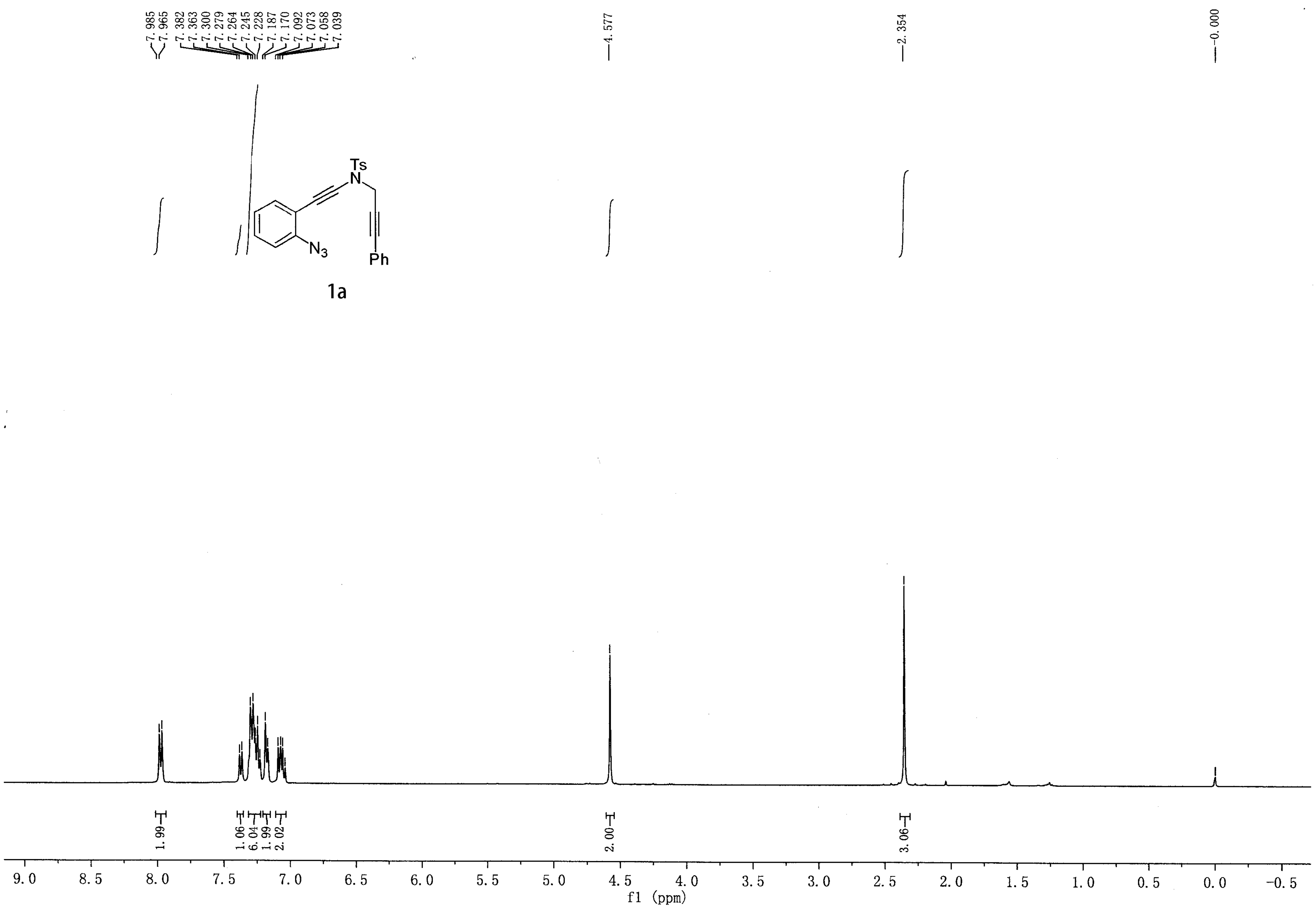

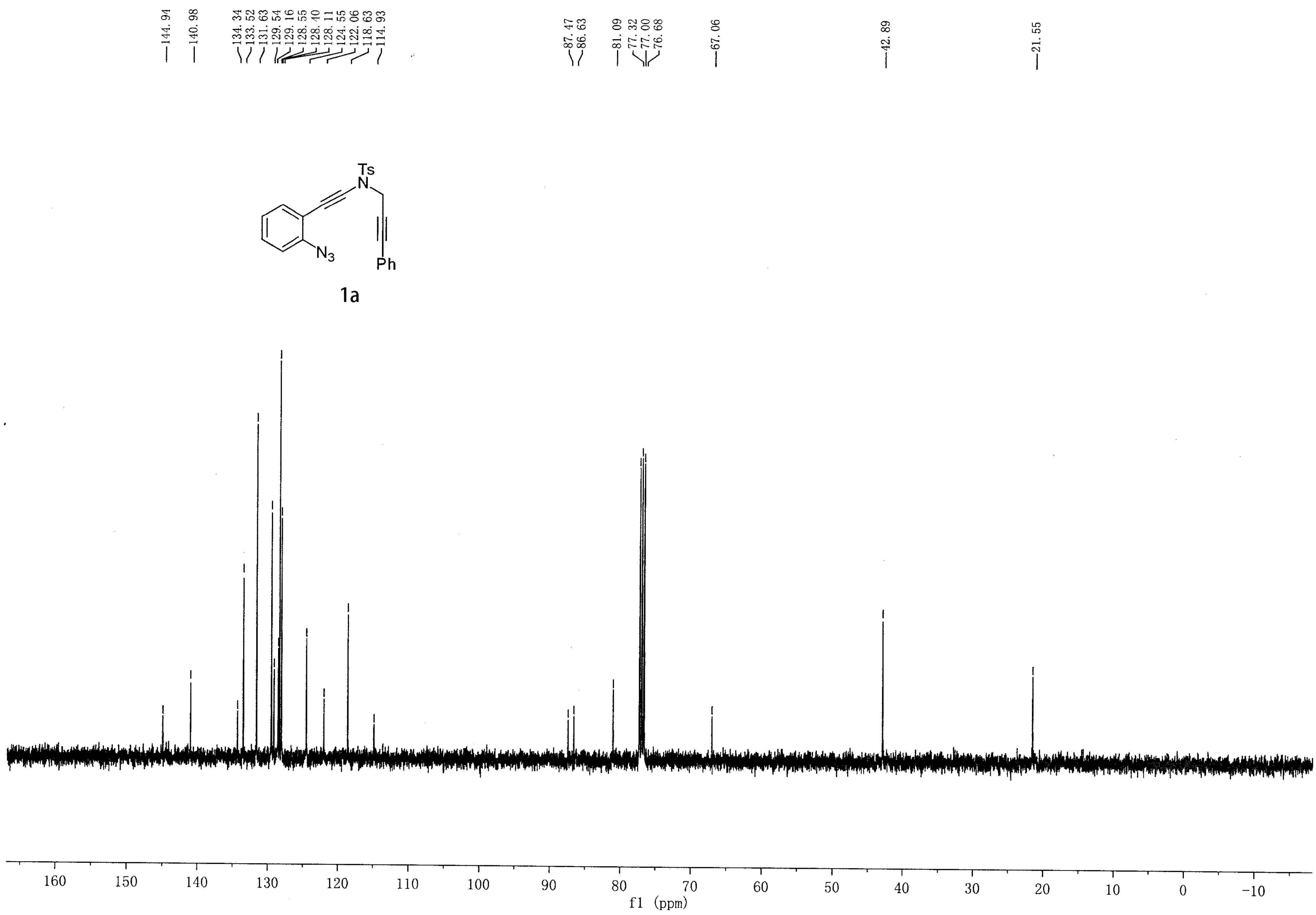

Supplementary Figure 1. ^{1}H and ^{13}C NMR spectra for 1a

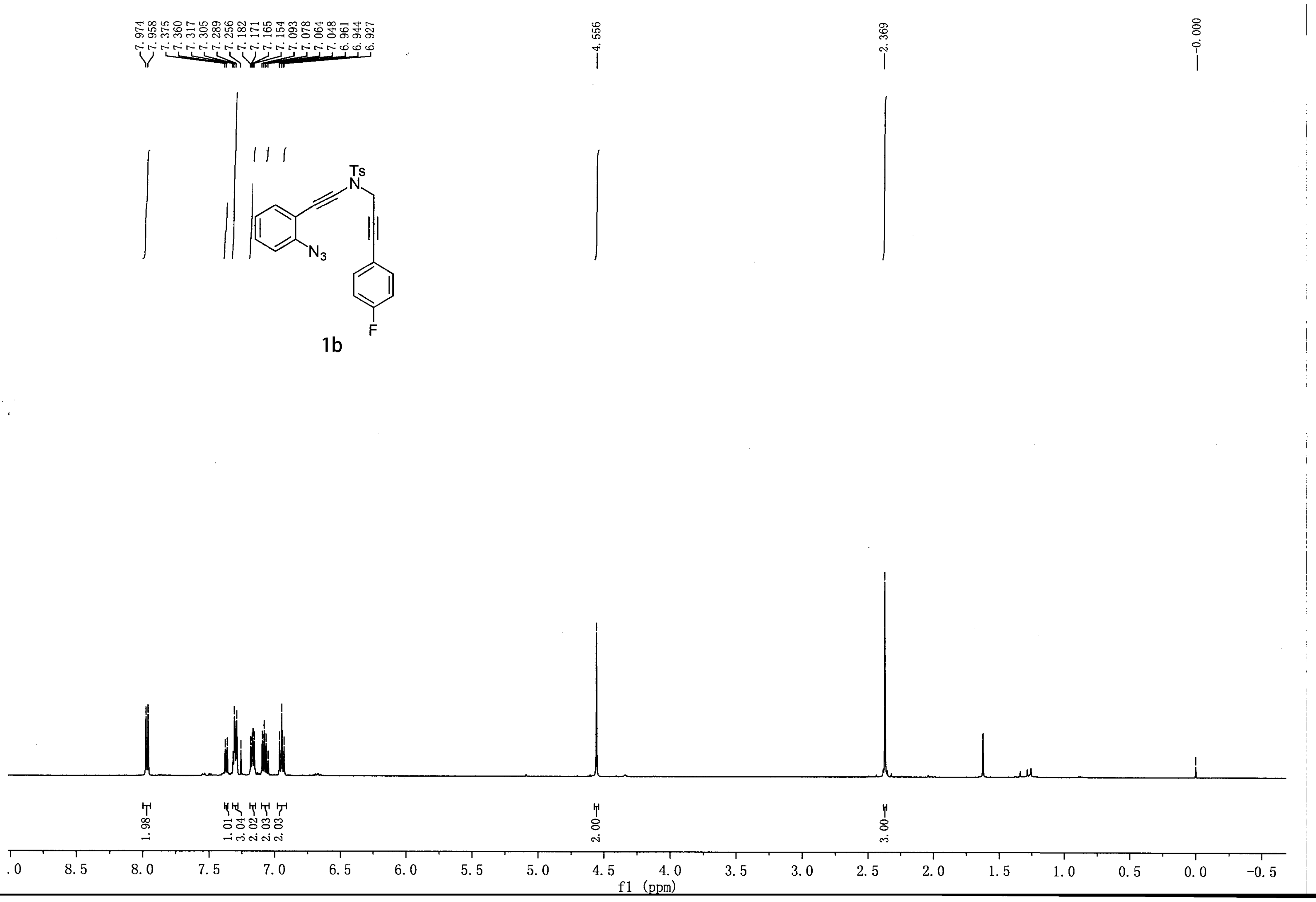

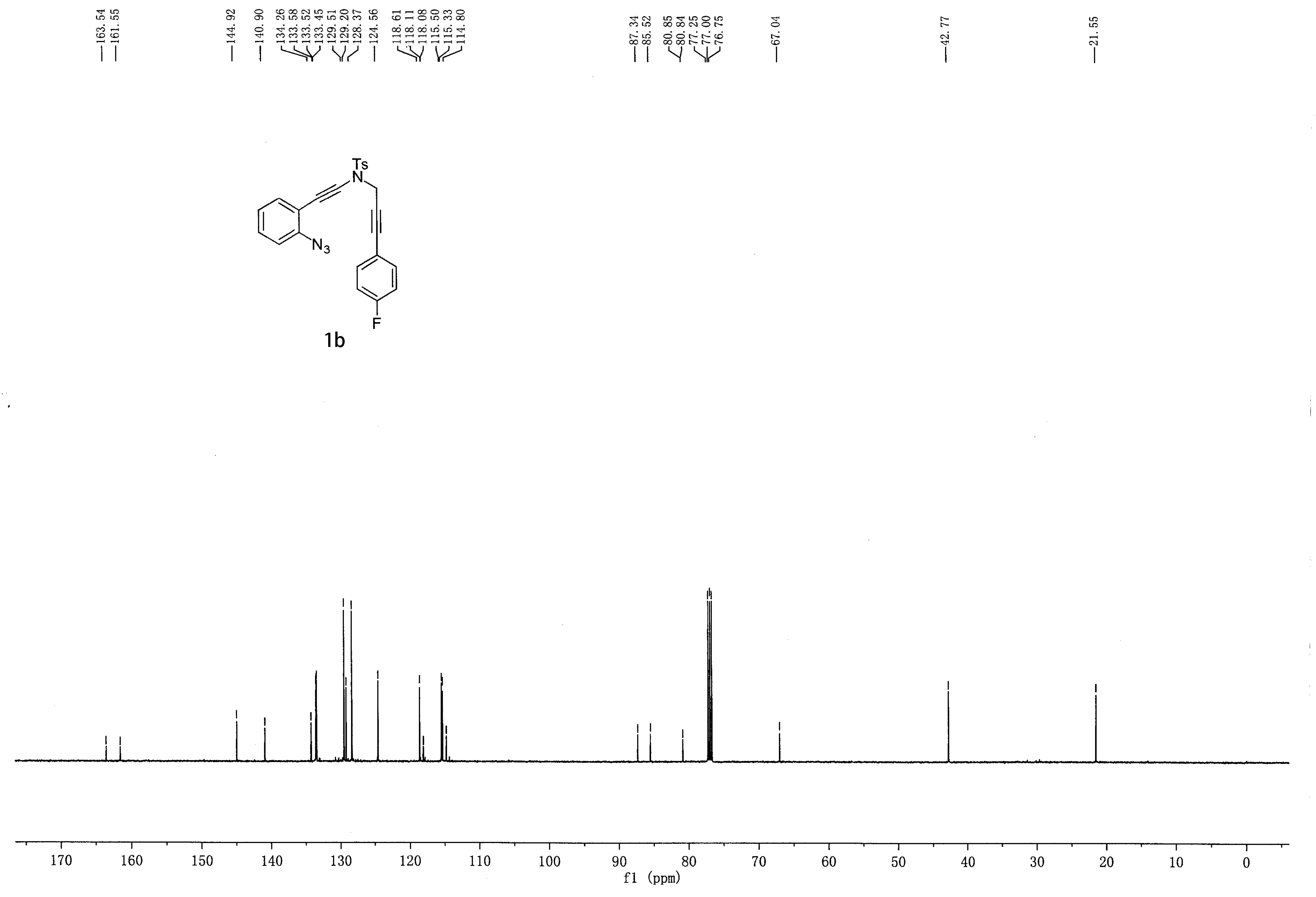

Supplementary Figure 2. ^{1}H and ^{13}C NMR spectra for 1b

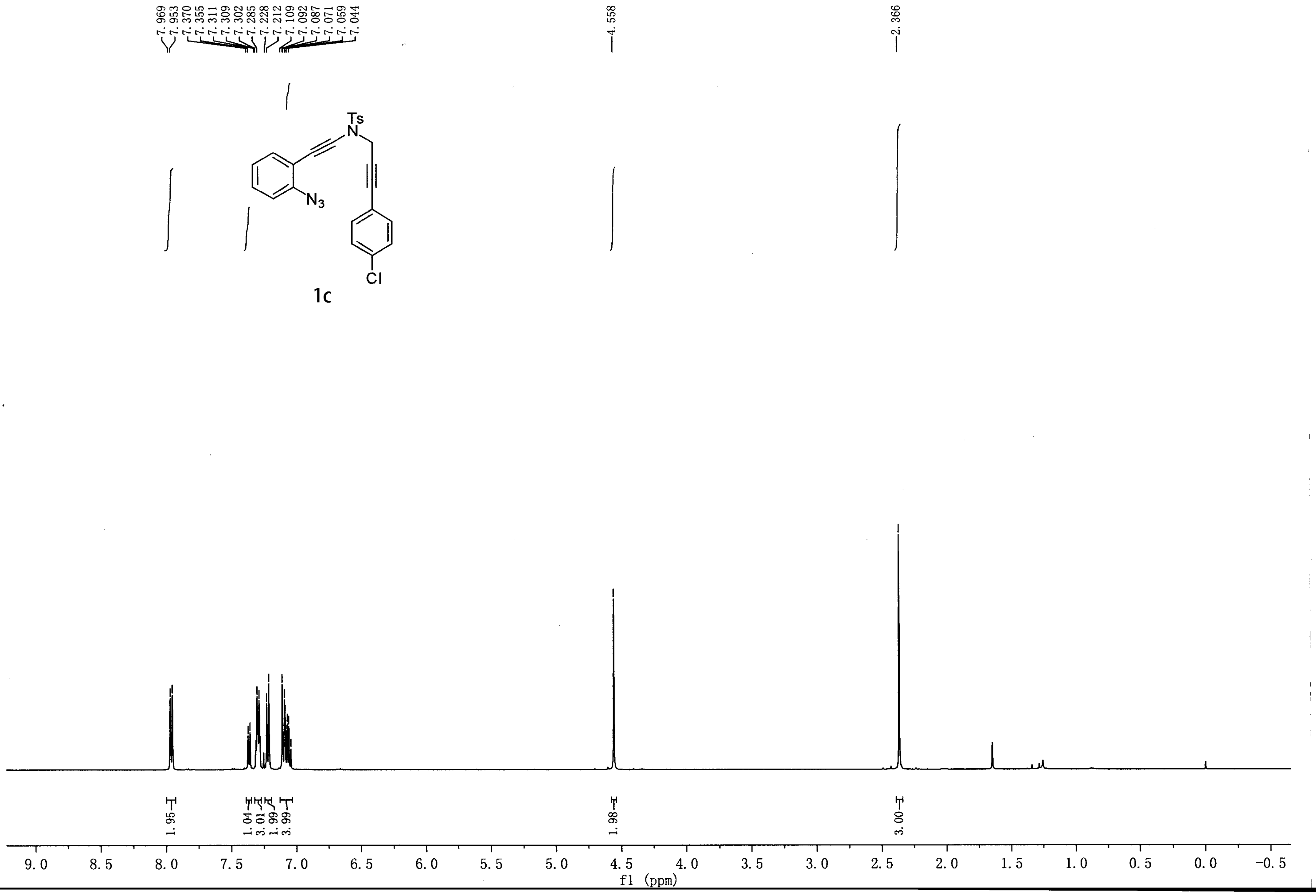

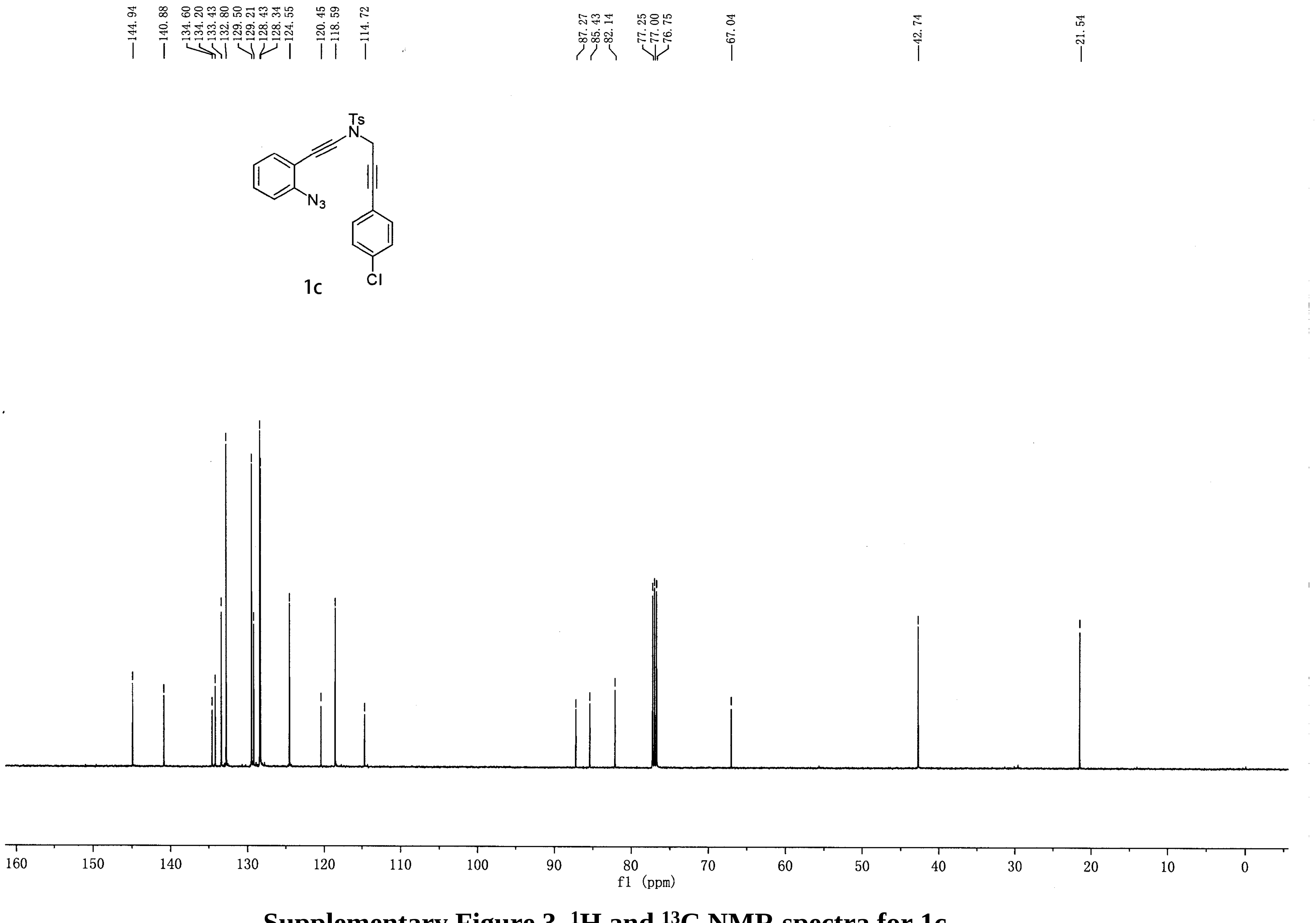

Supplementary Figure 3. ¹H and ¹³C NMR spectra for 1c

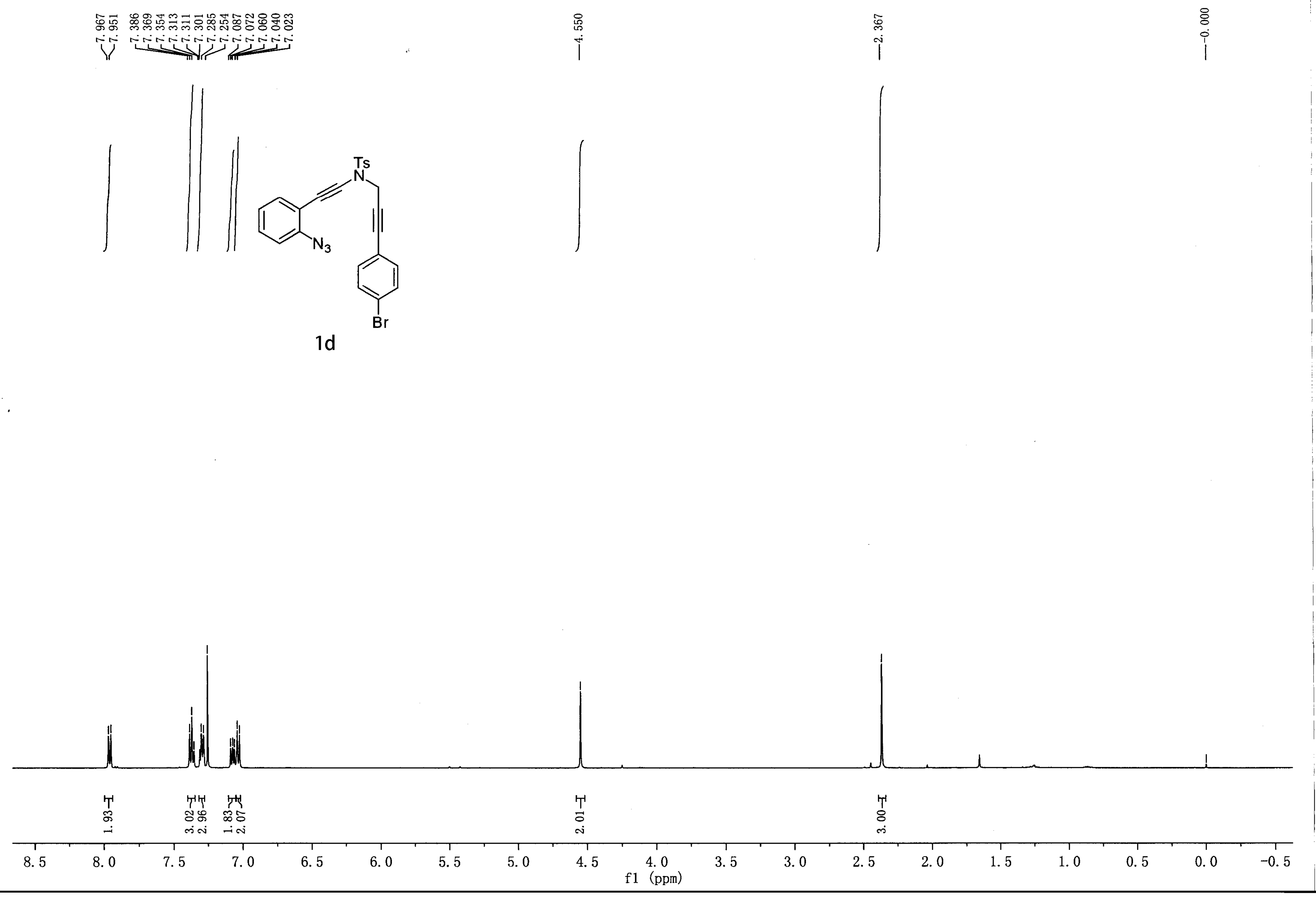

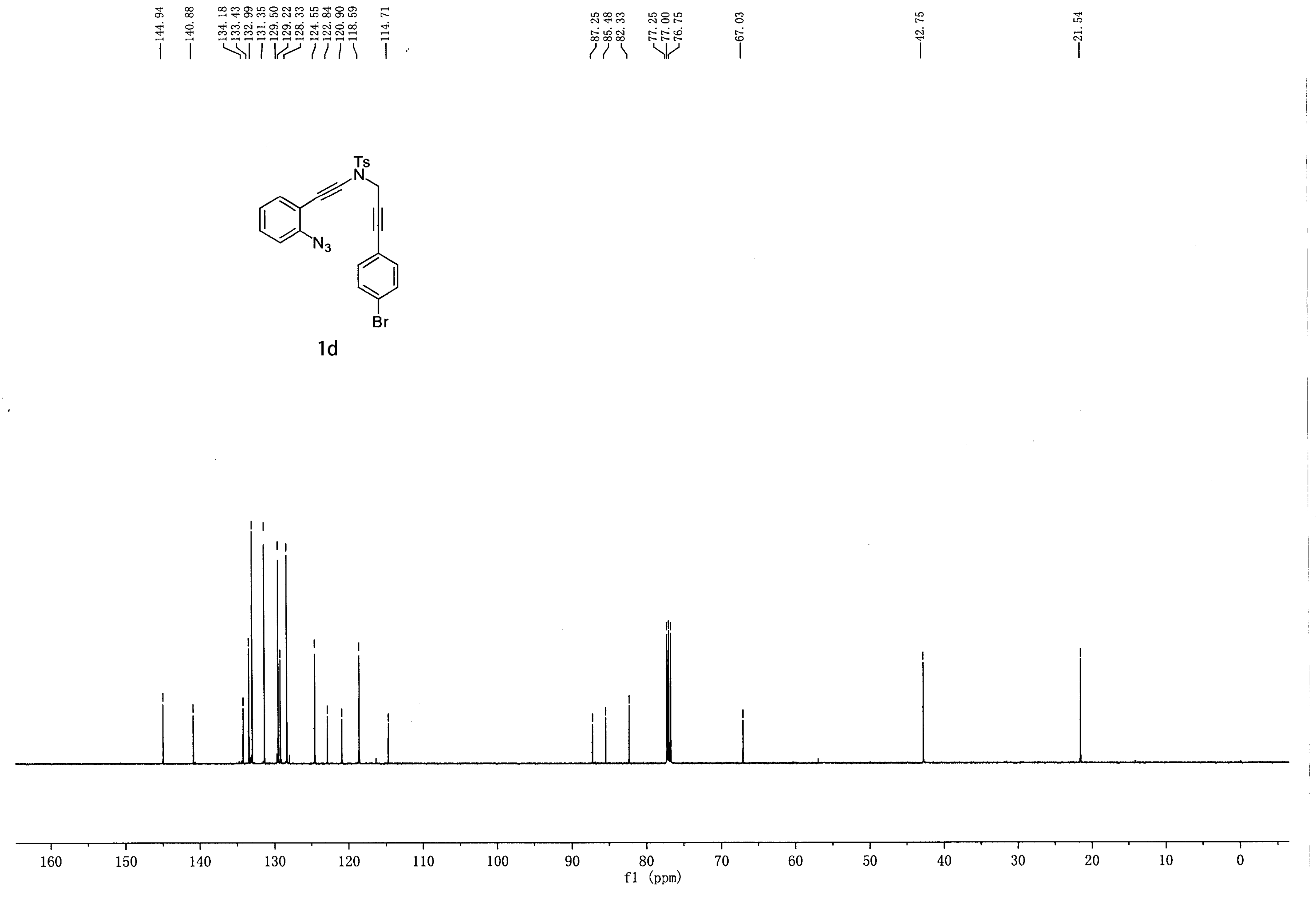

Supplementary Figure 4. ^{1}H and ^{13}C NMR spectra for 1d

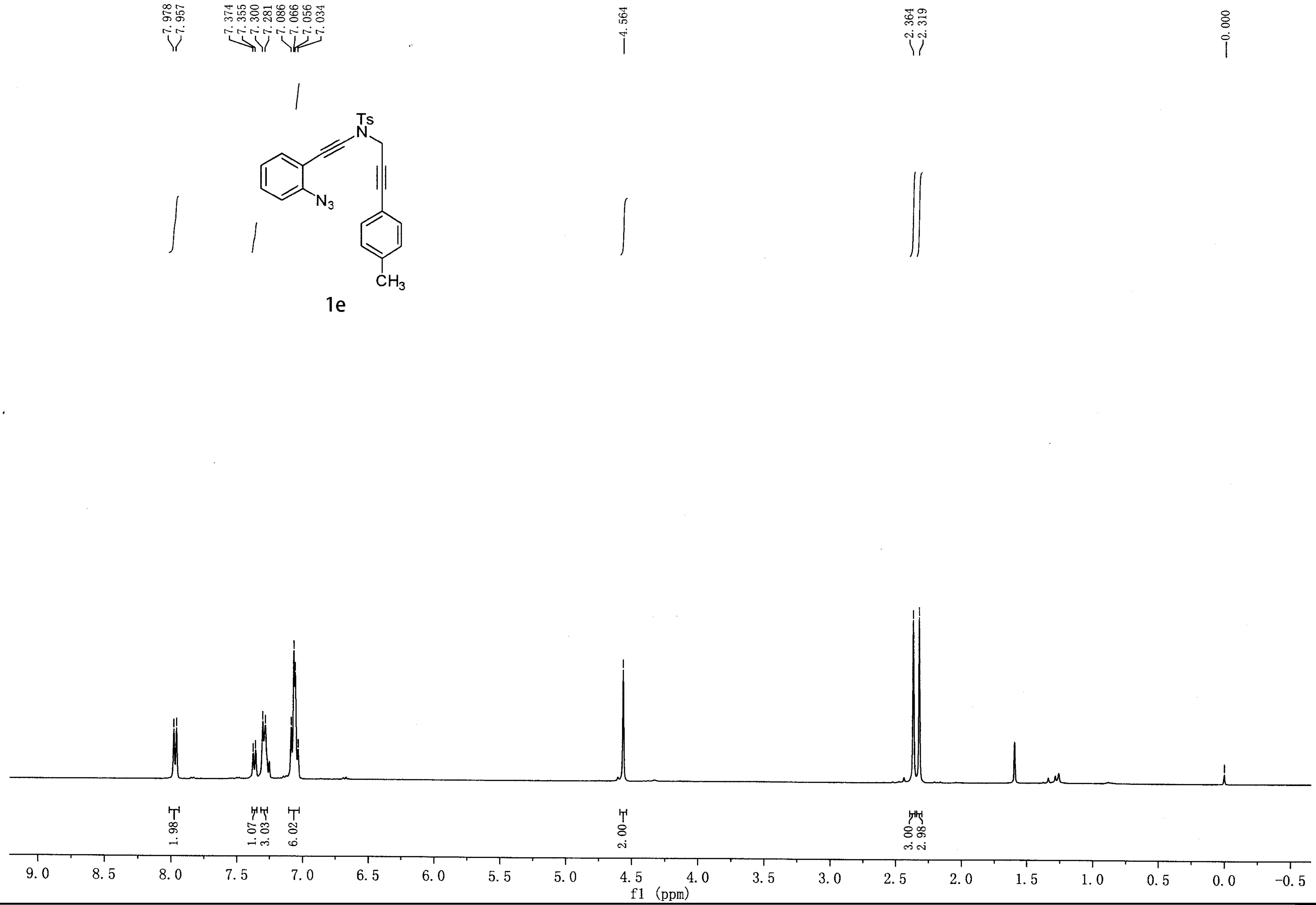

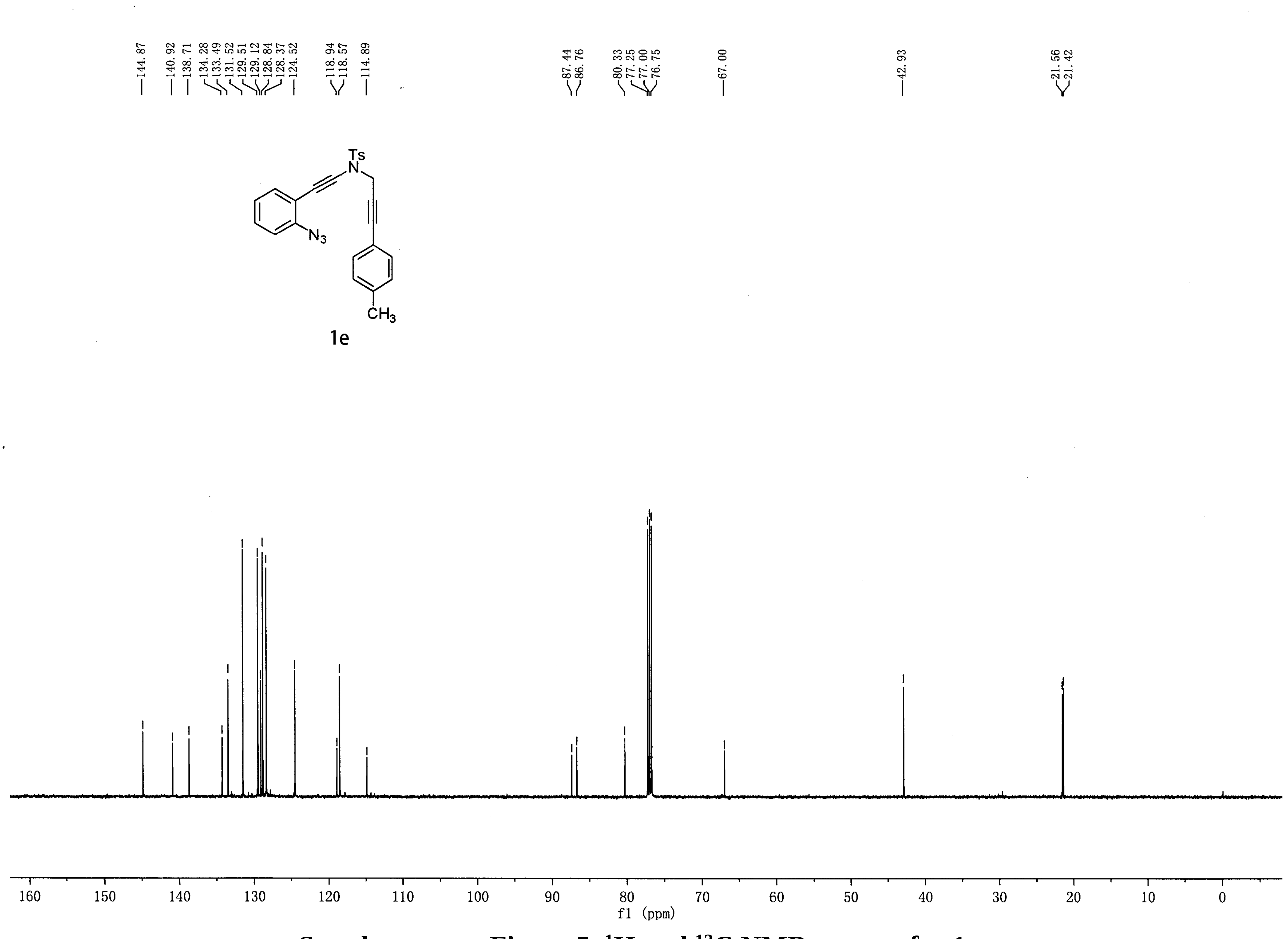

Supplementary Figure 5. ^{1}H and ^{13}C NMR spectra for 1e

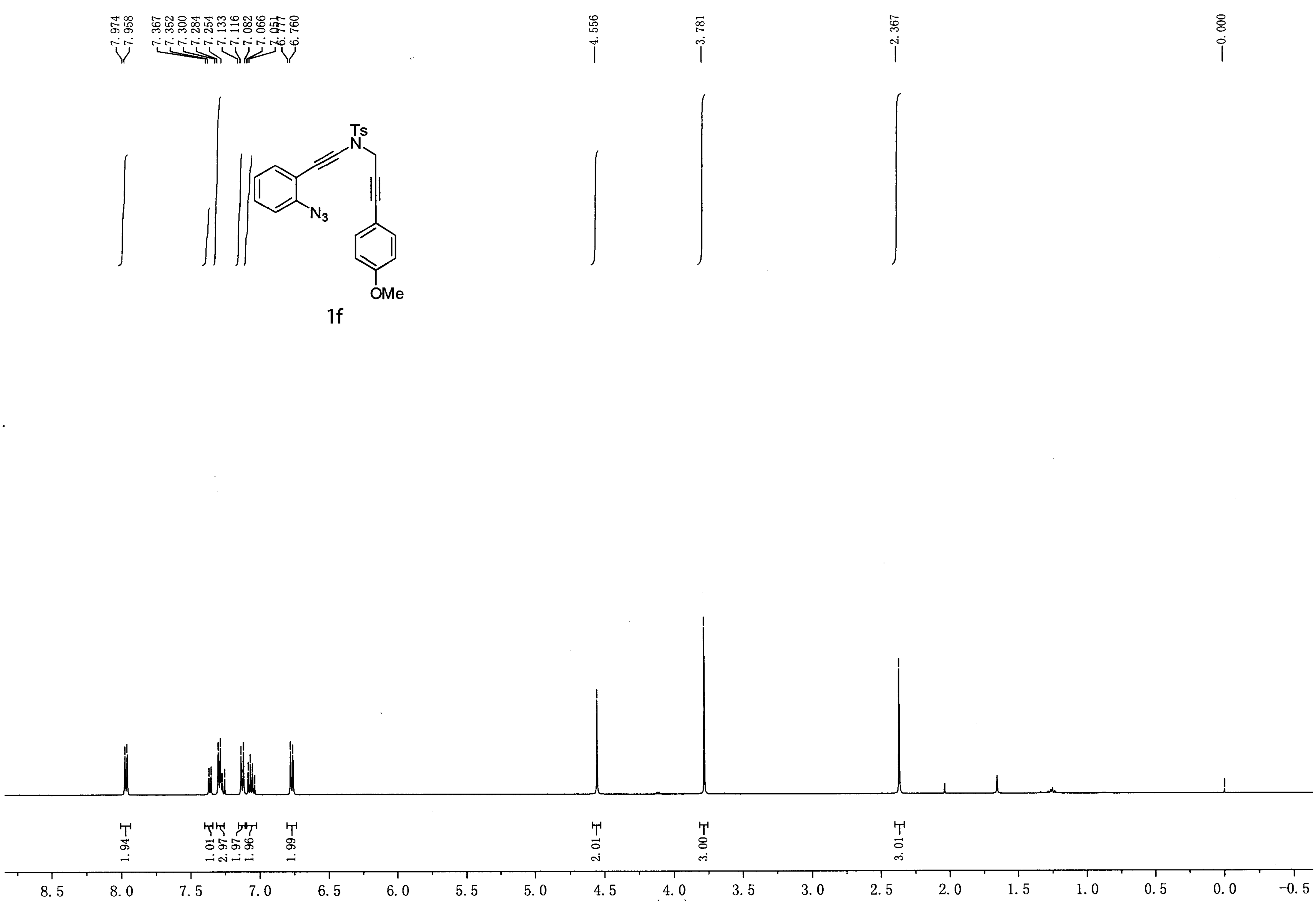

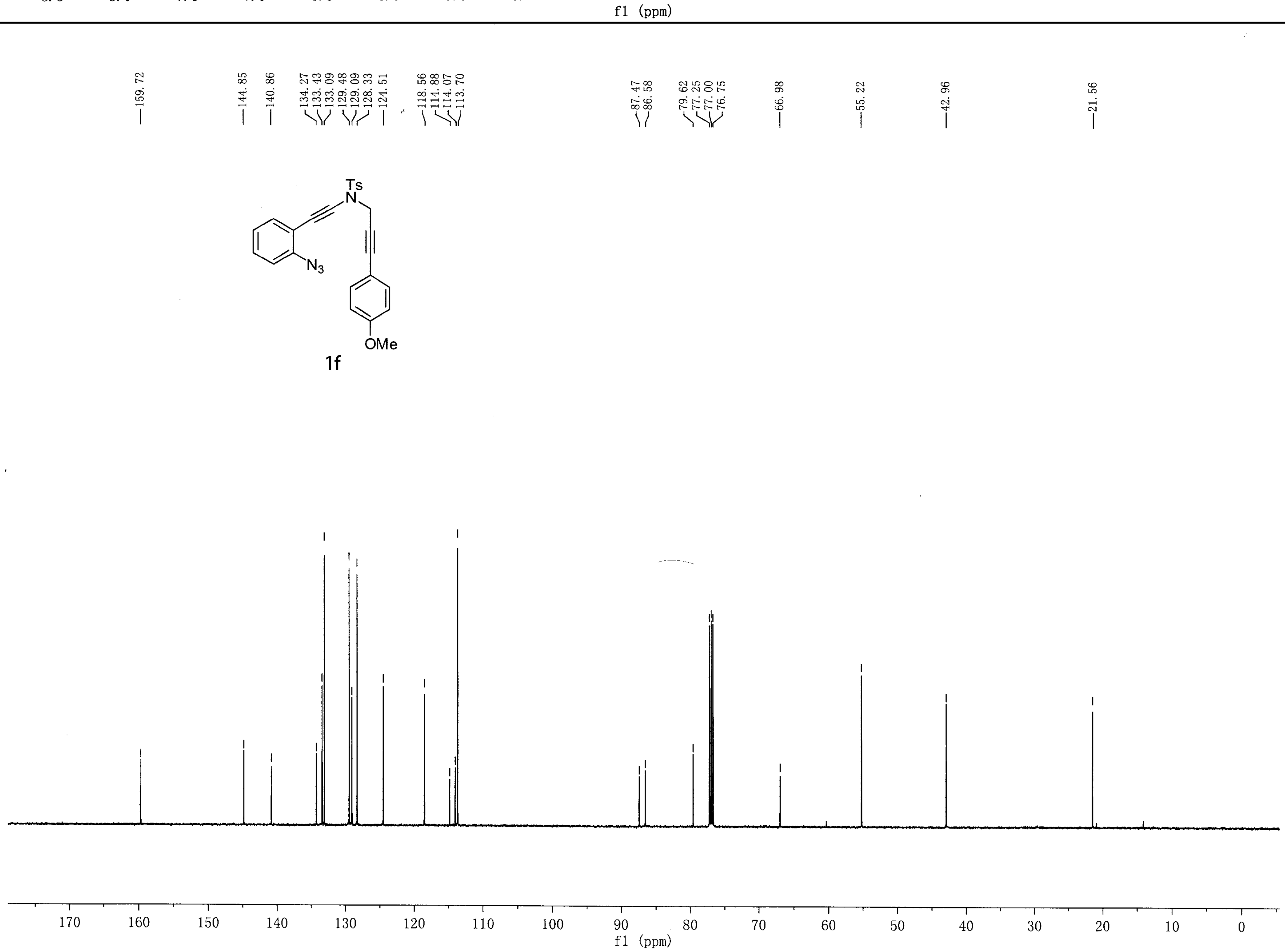

Supplementary Figure 6. ^{1}H and ^{13}C NMR spectra for 1f

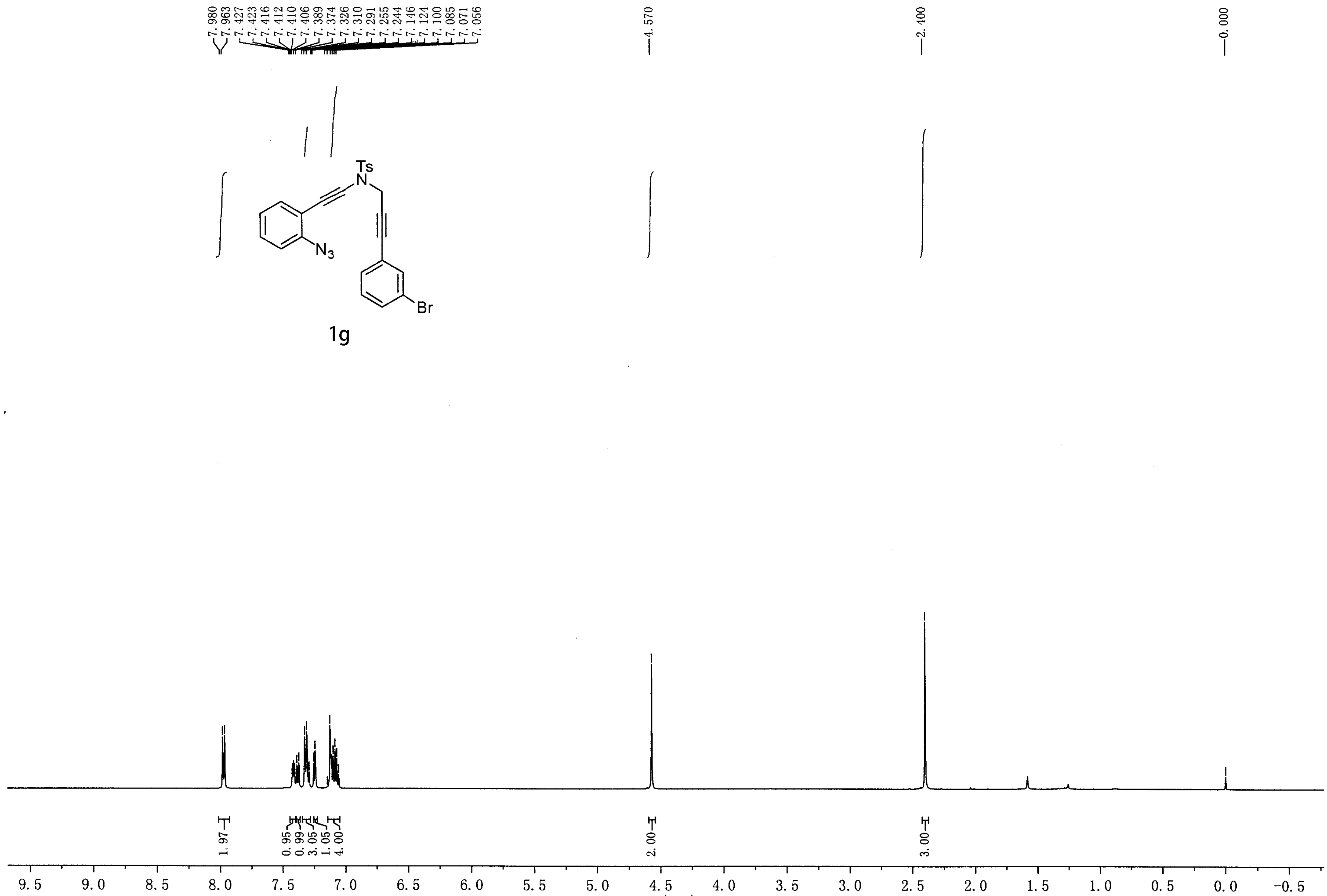

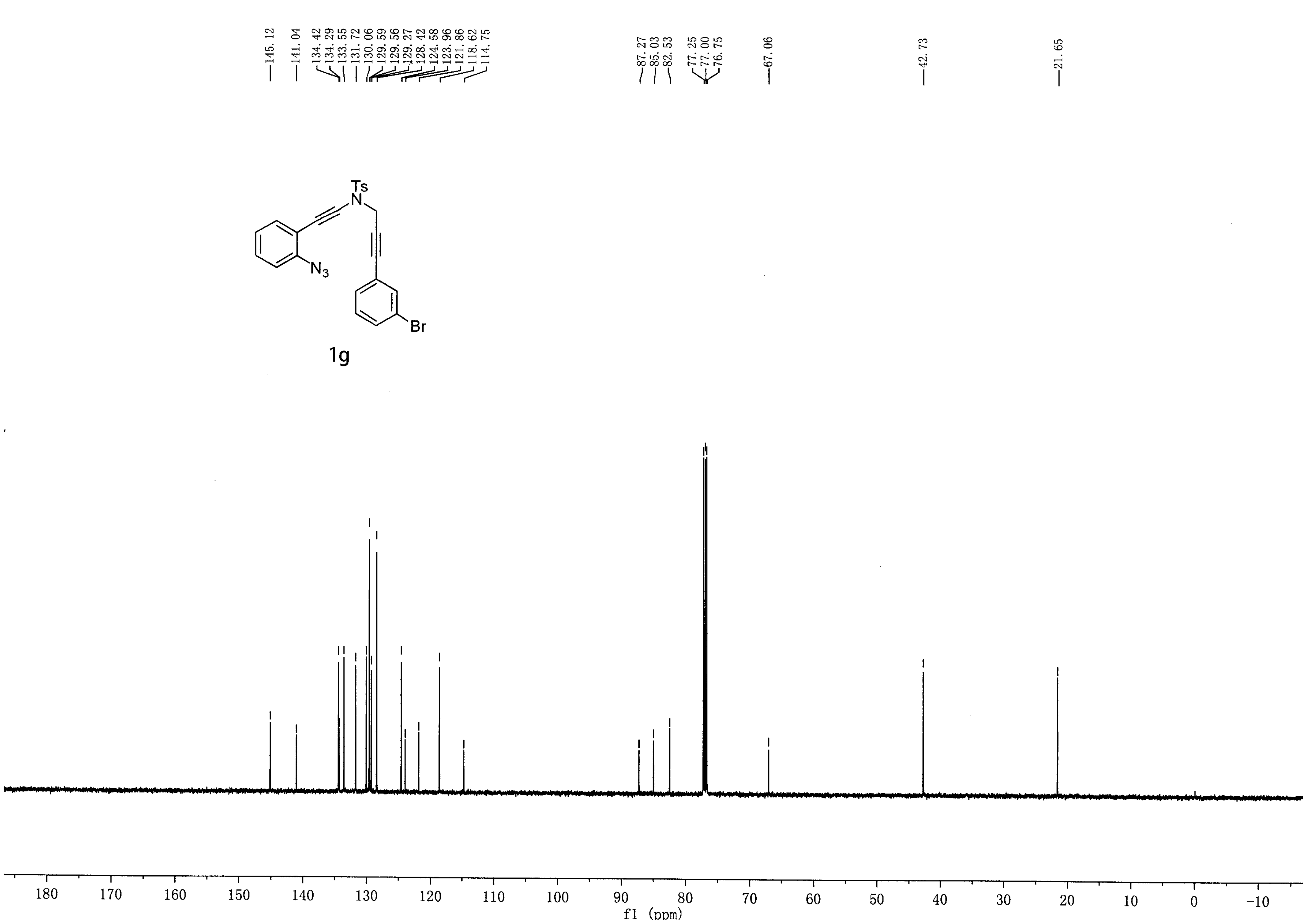

Supplementary Figure 7. ^{1}H and ^{13}C NMR spectra for 1g

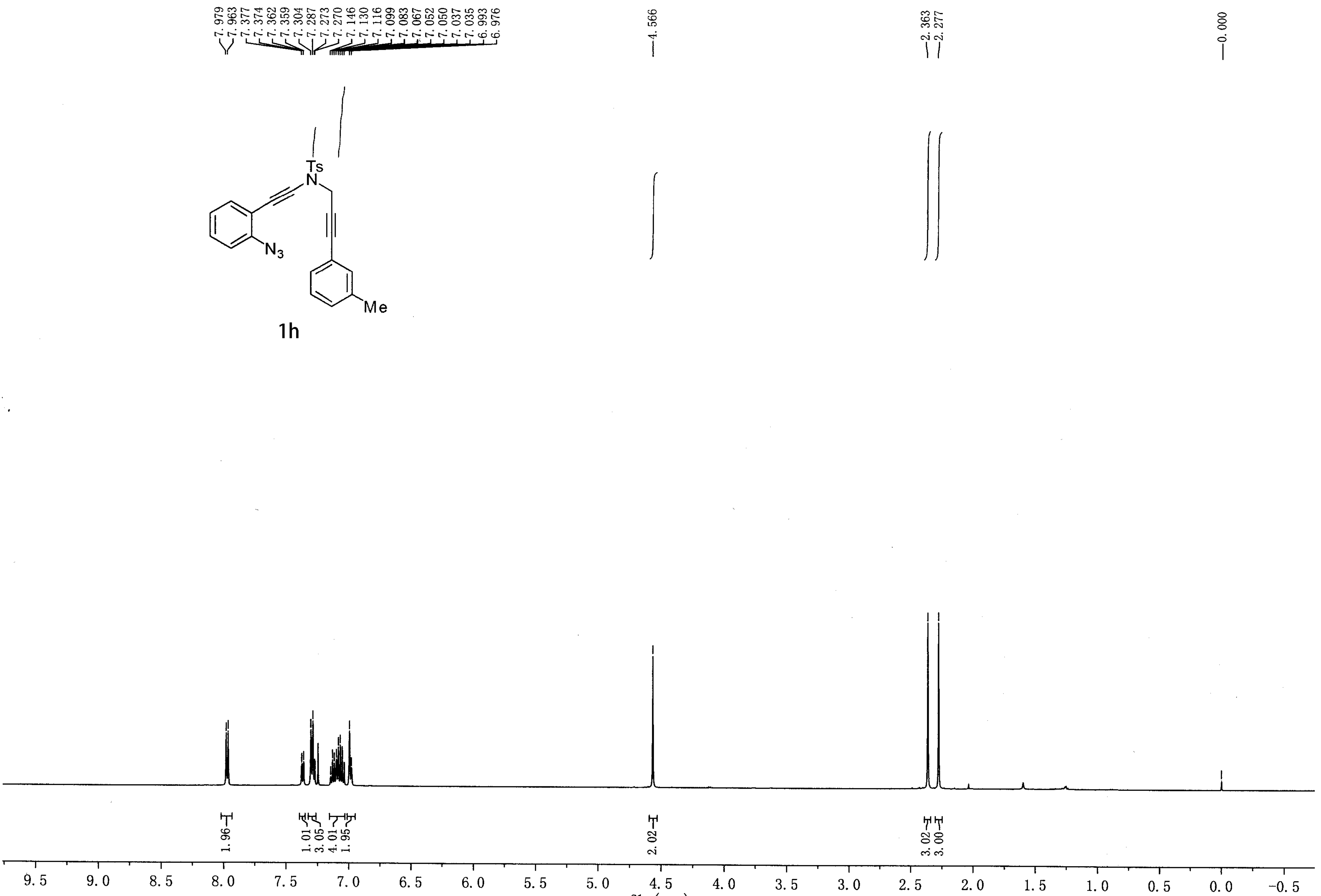

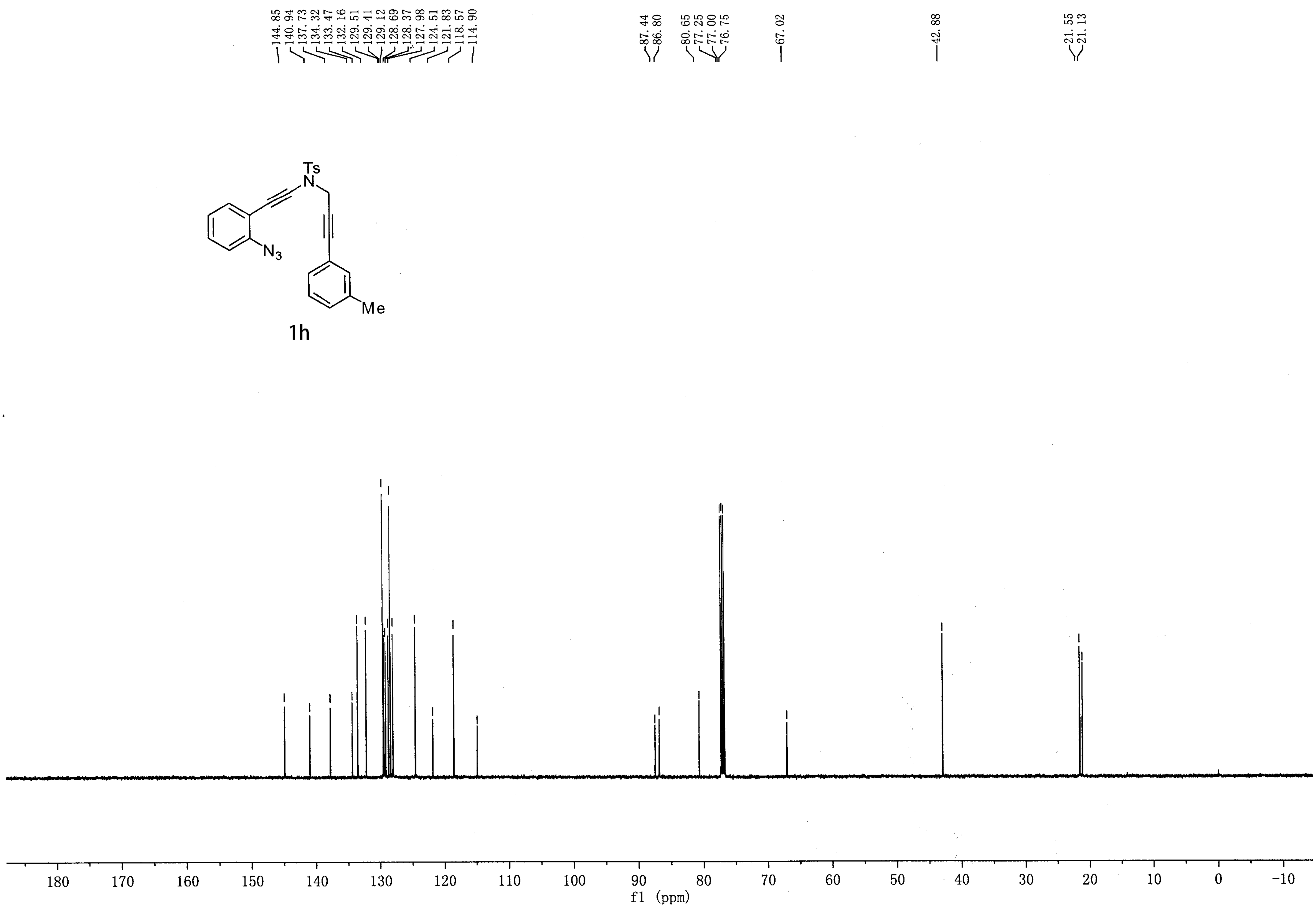

Supplementary Figure 8. ^{1}H and ^{13}C NMR spectra for 1h

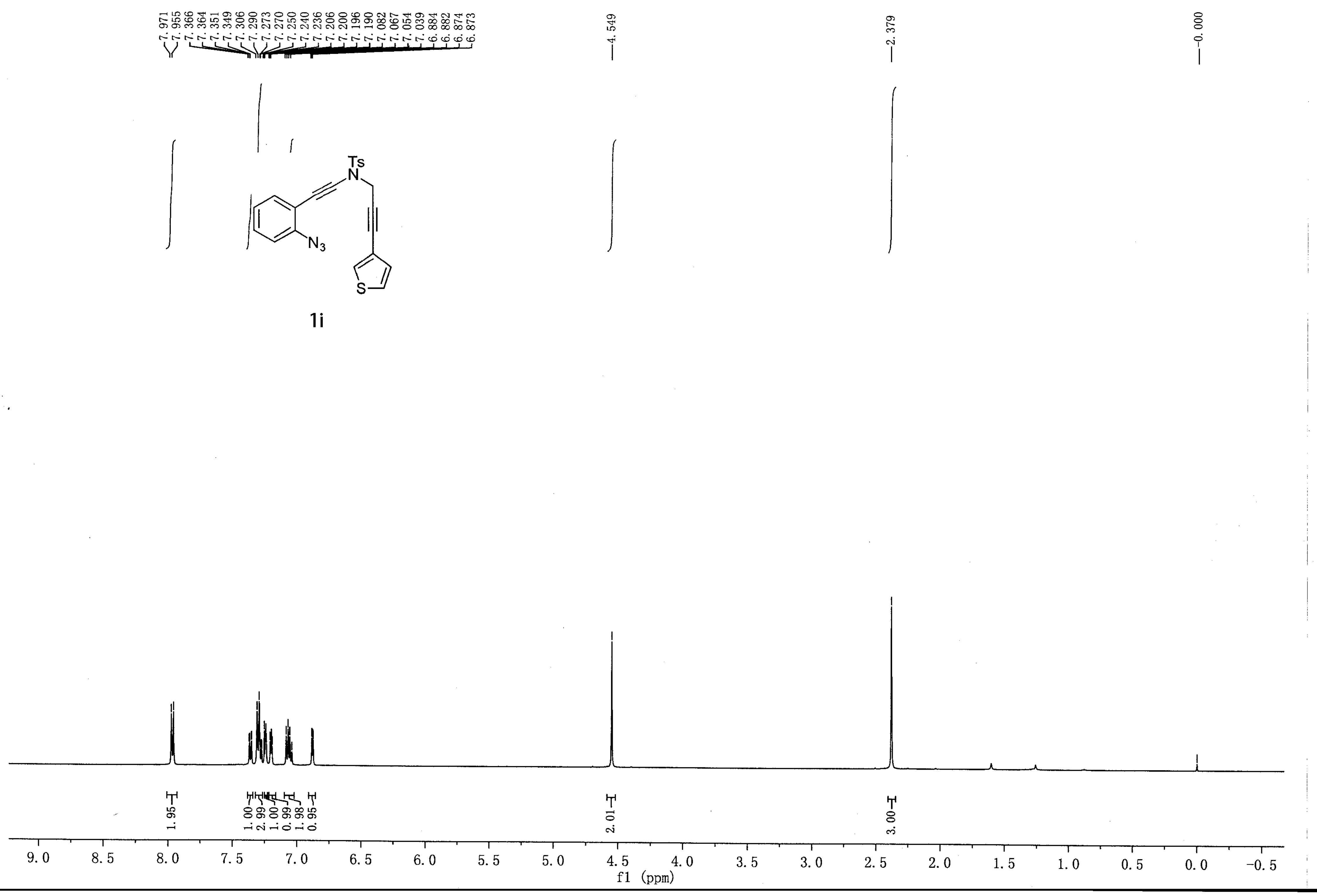

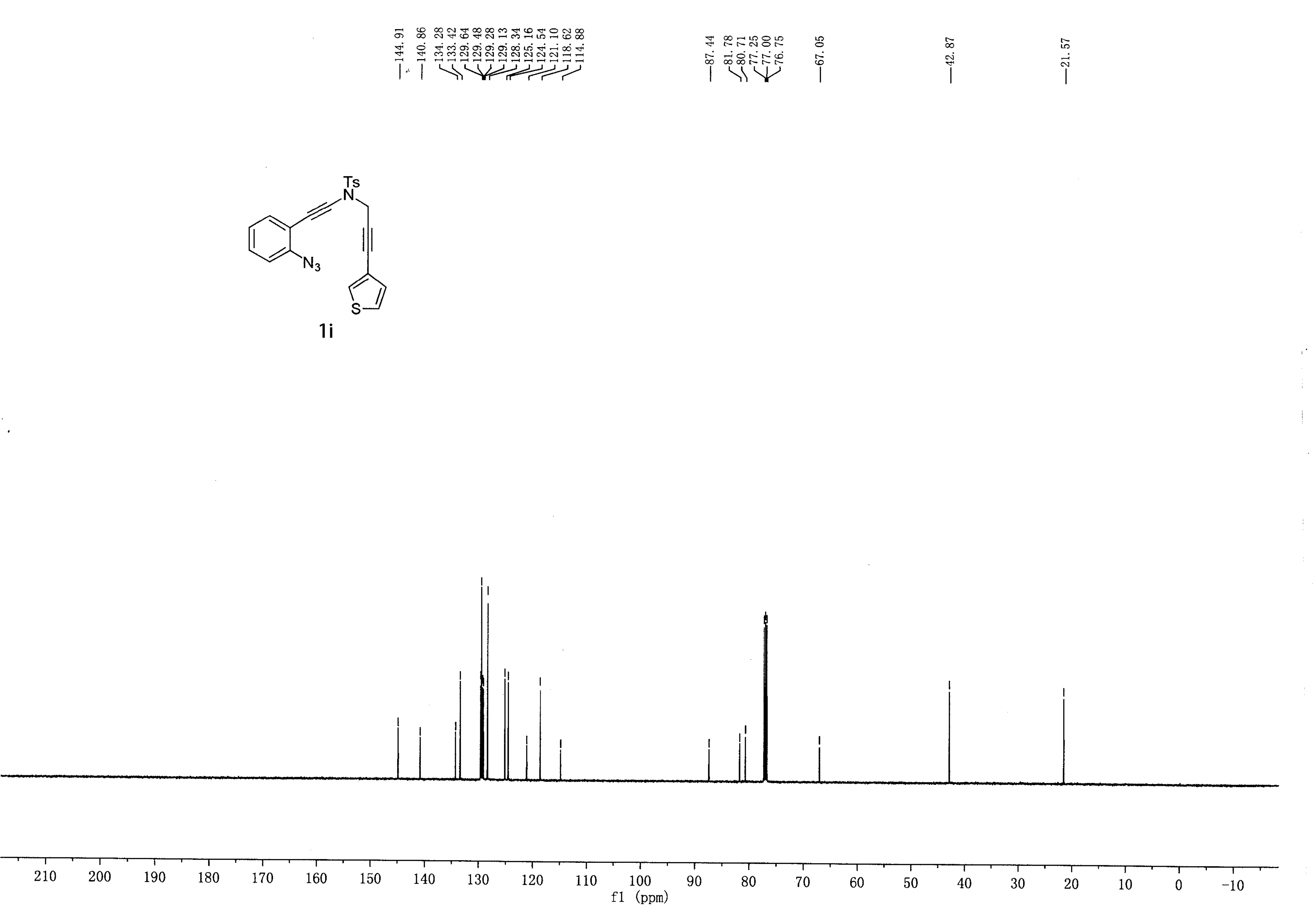

Supplementary Figure 9. ^{1}H and ^{13}C NMR spectra for 1i

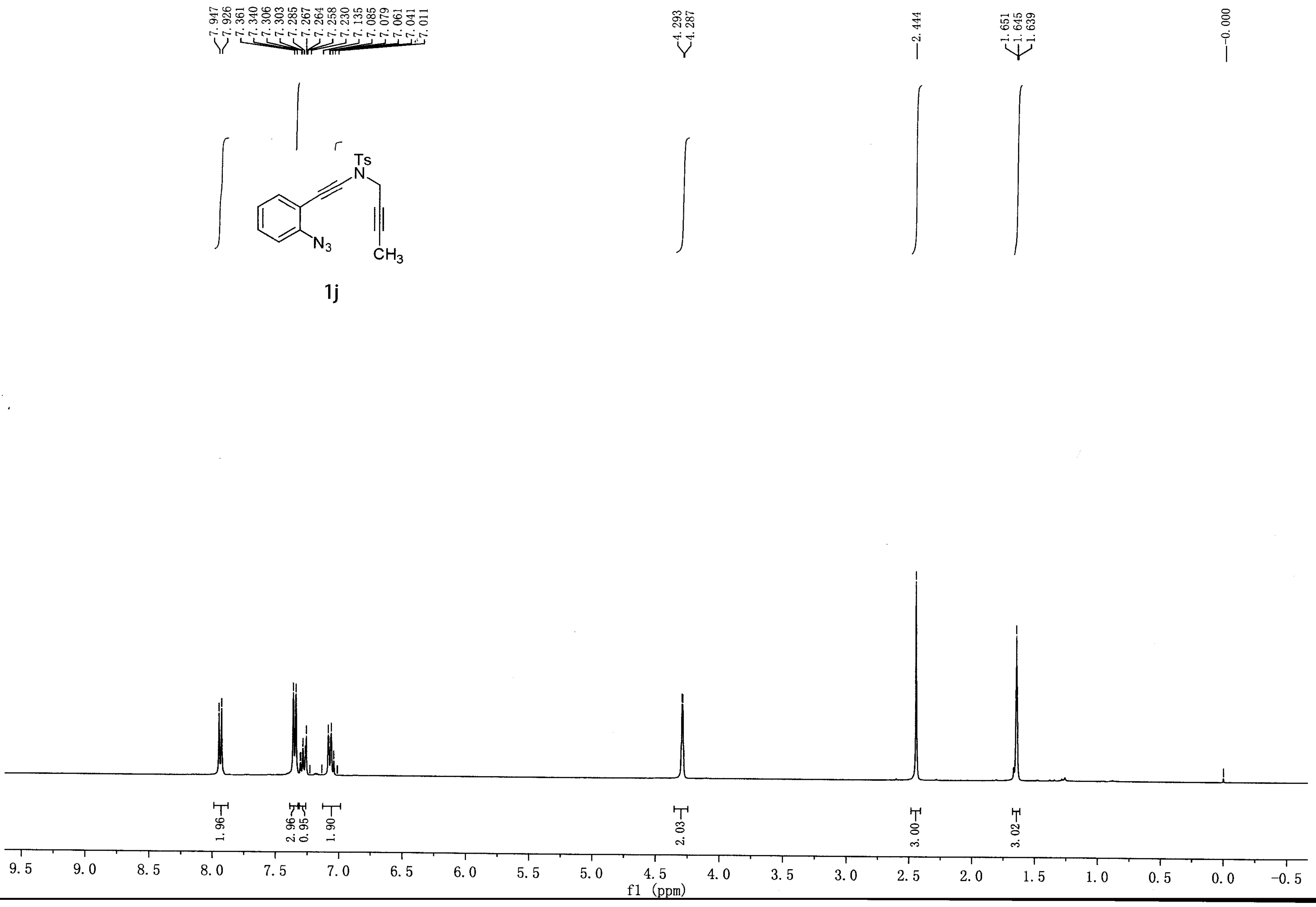

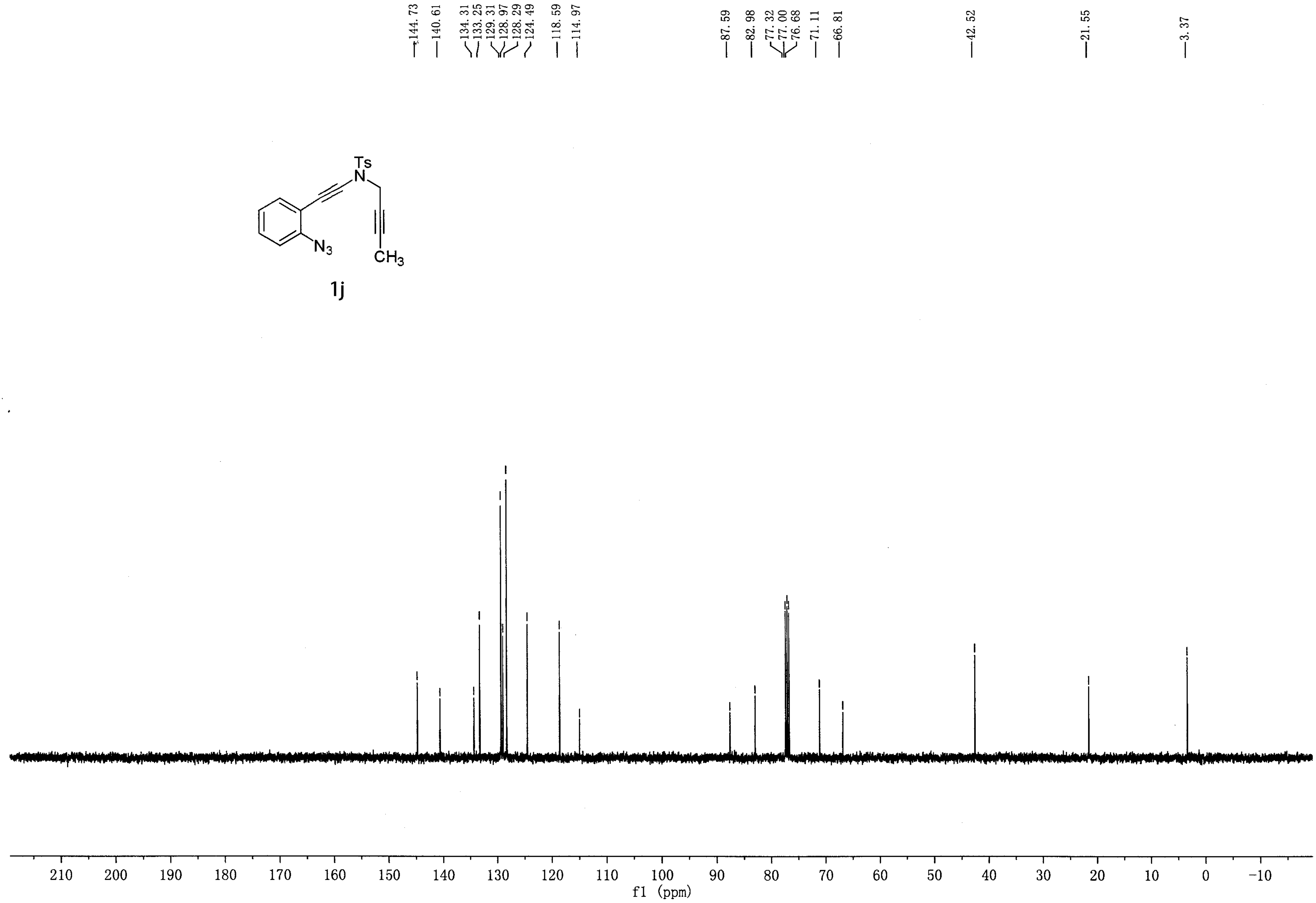

Supplementary Figure 10. ^{1}H and ^{13}C NMR spectra for 1j

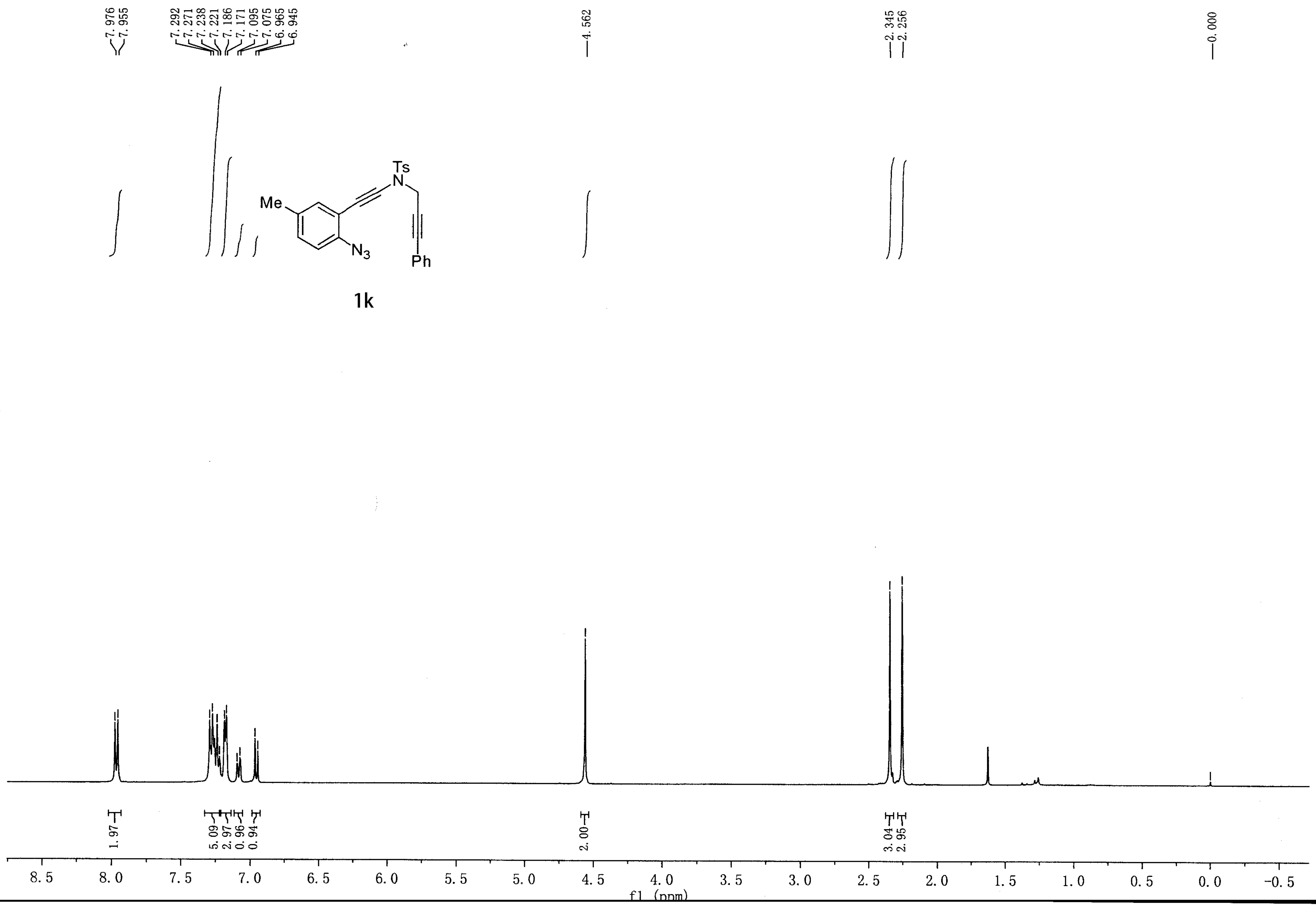

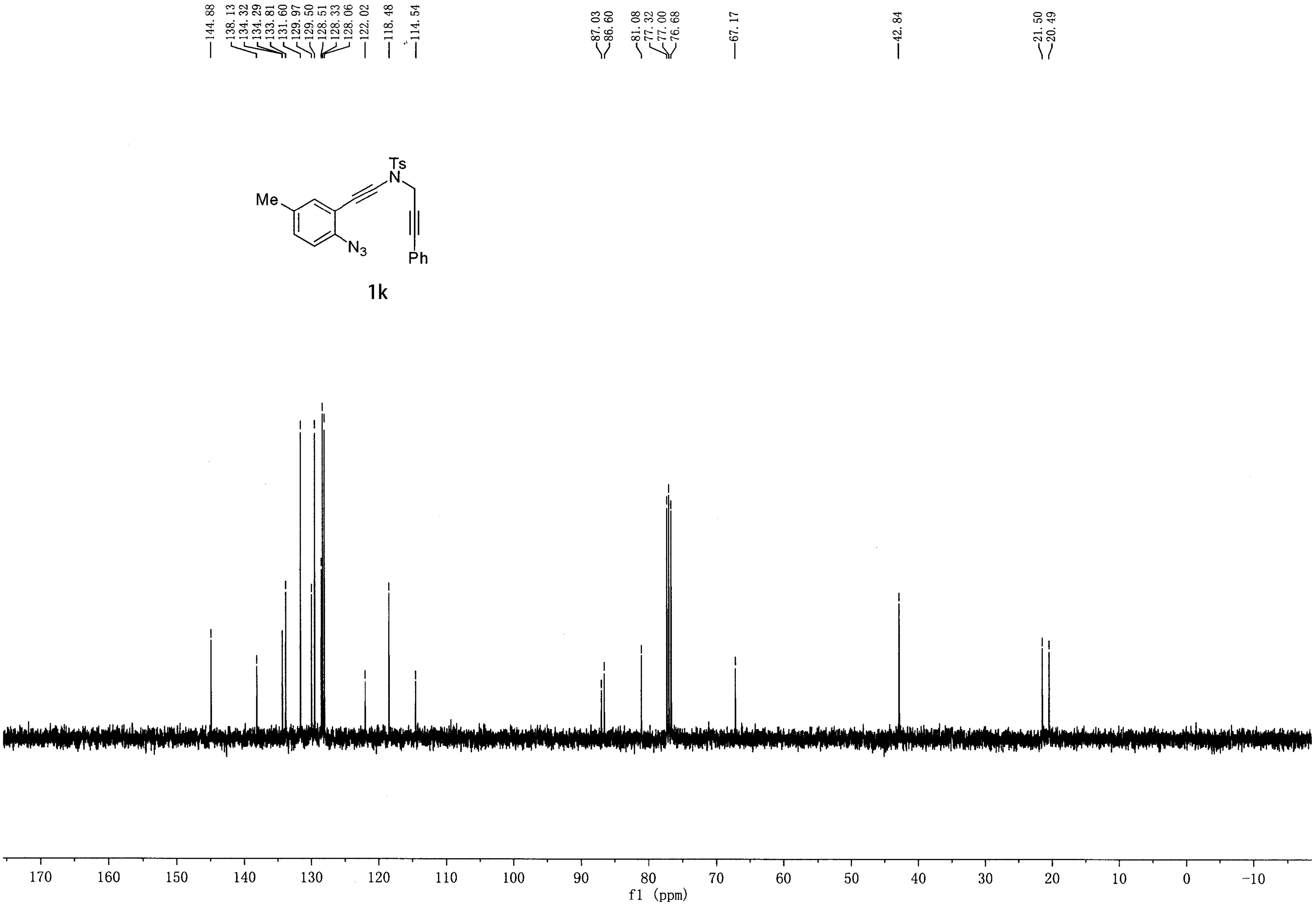

Supplementary Figure 11. ^{1}H and ^{13}C NMR spectra for 1k

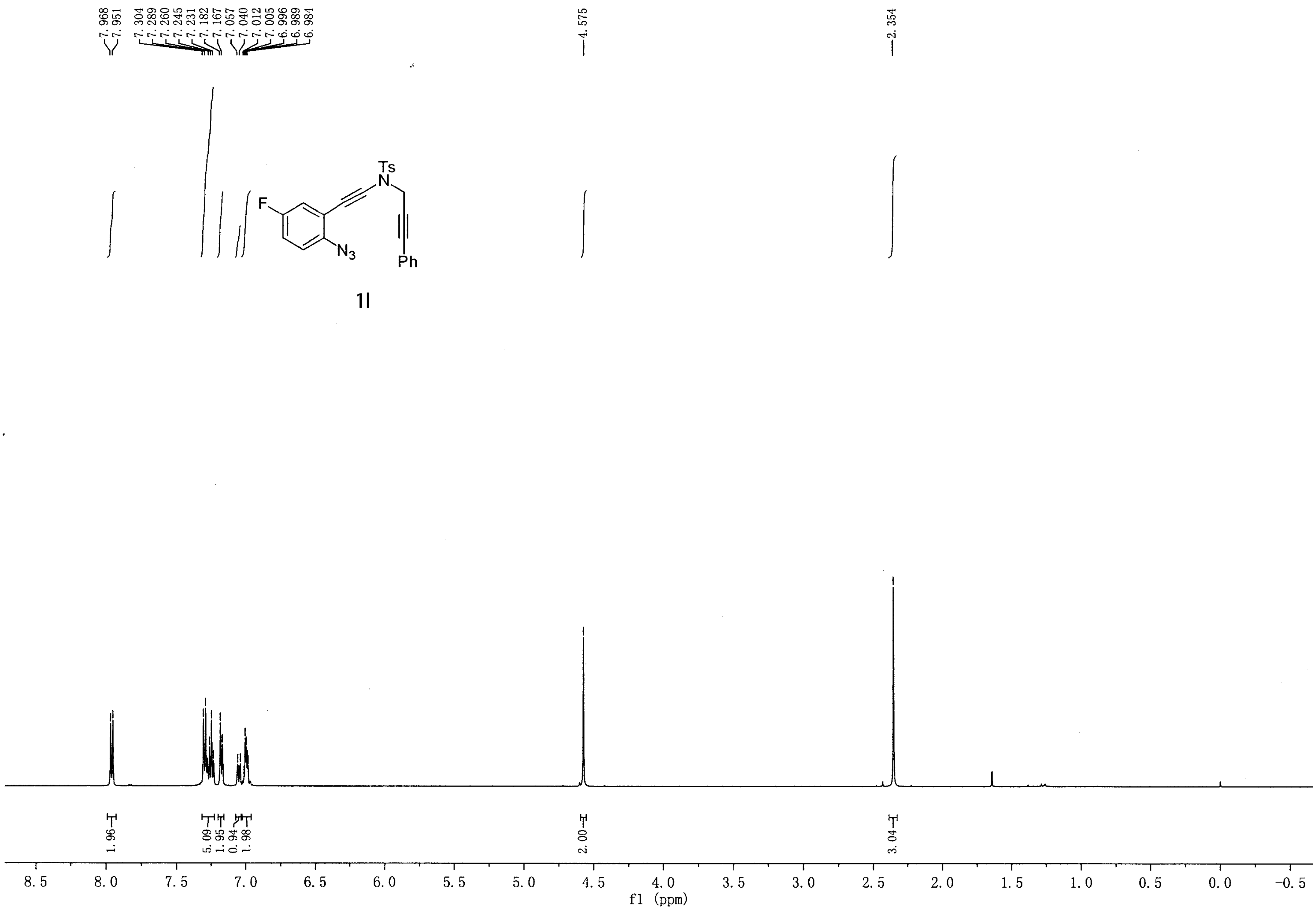

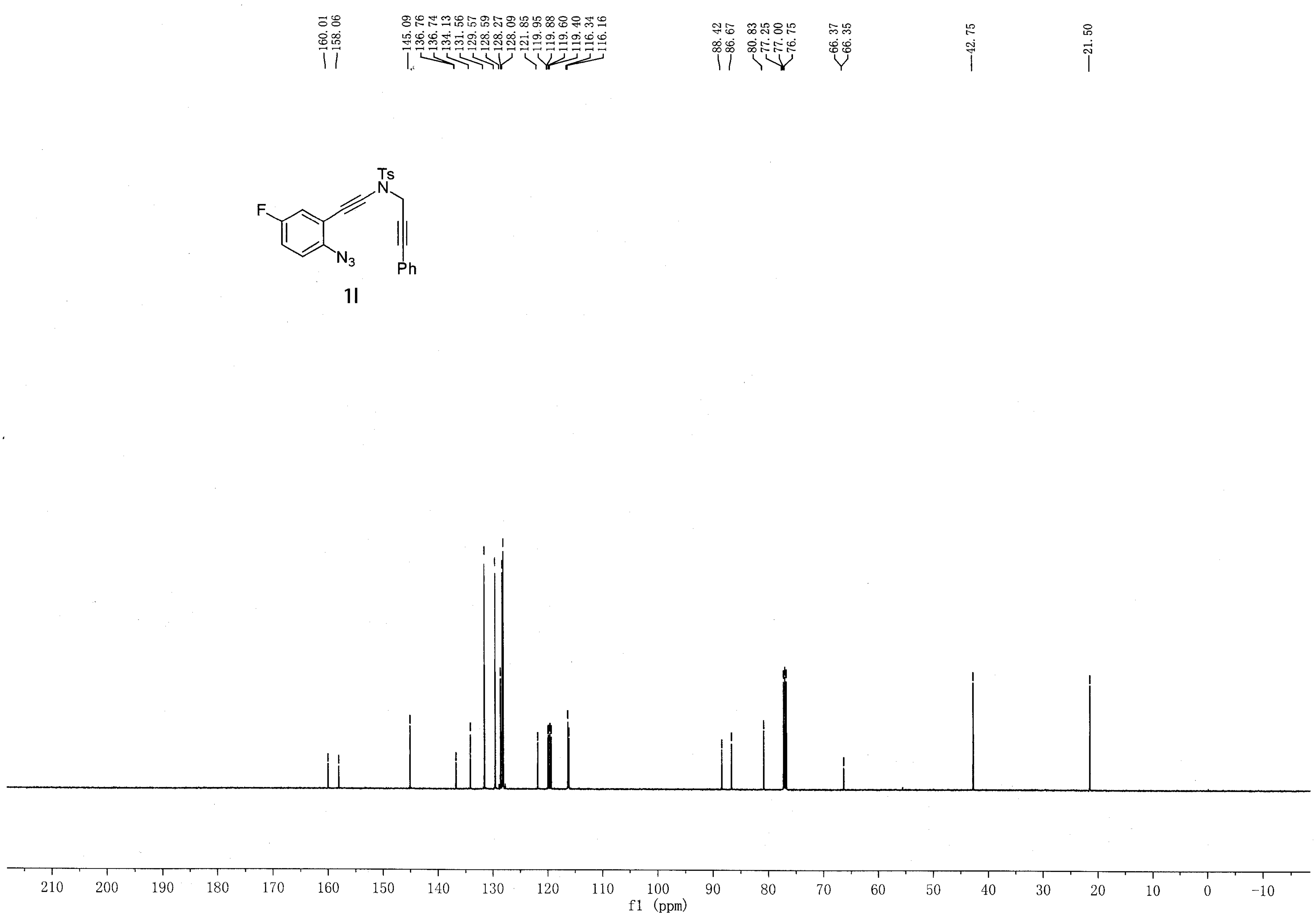

Supplementary Figure 12. ^{1}H and ^{13}C NMR spectra for 1l

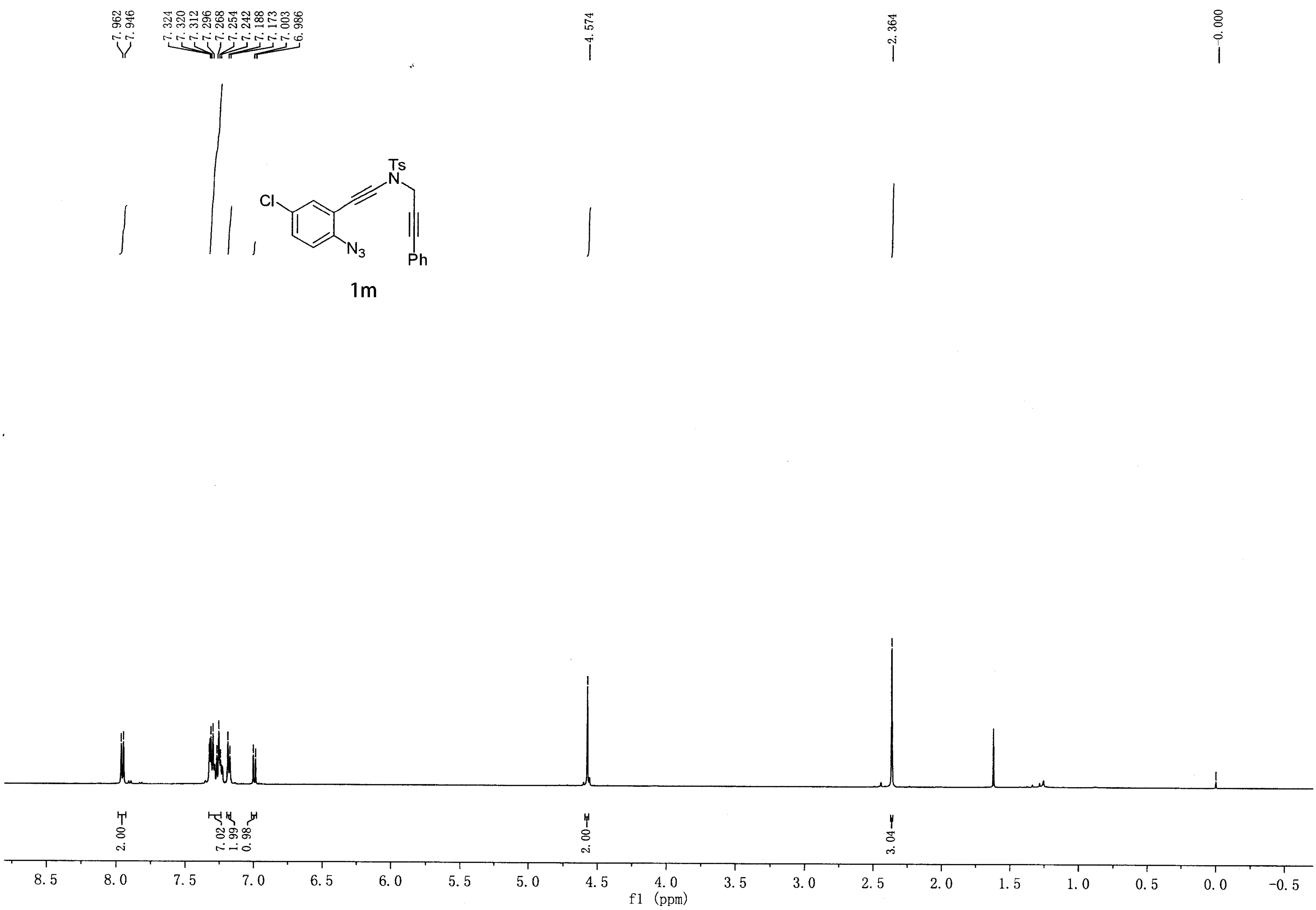

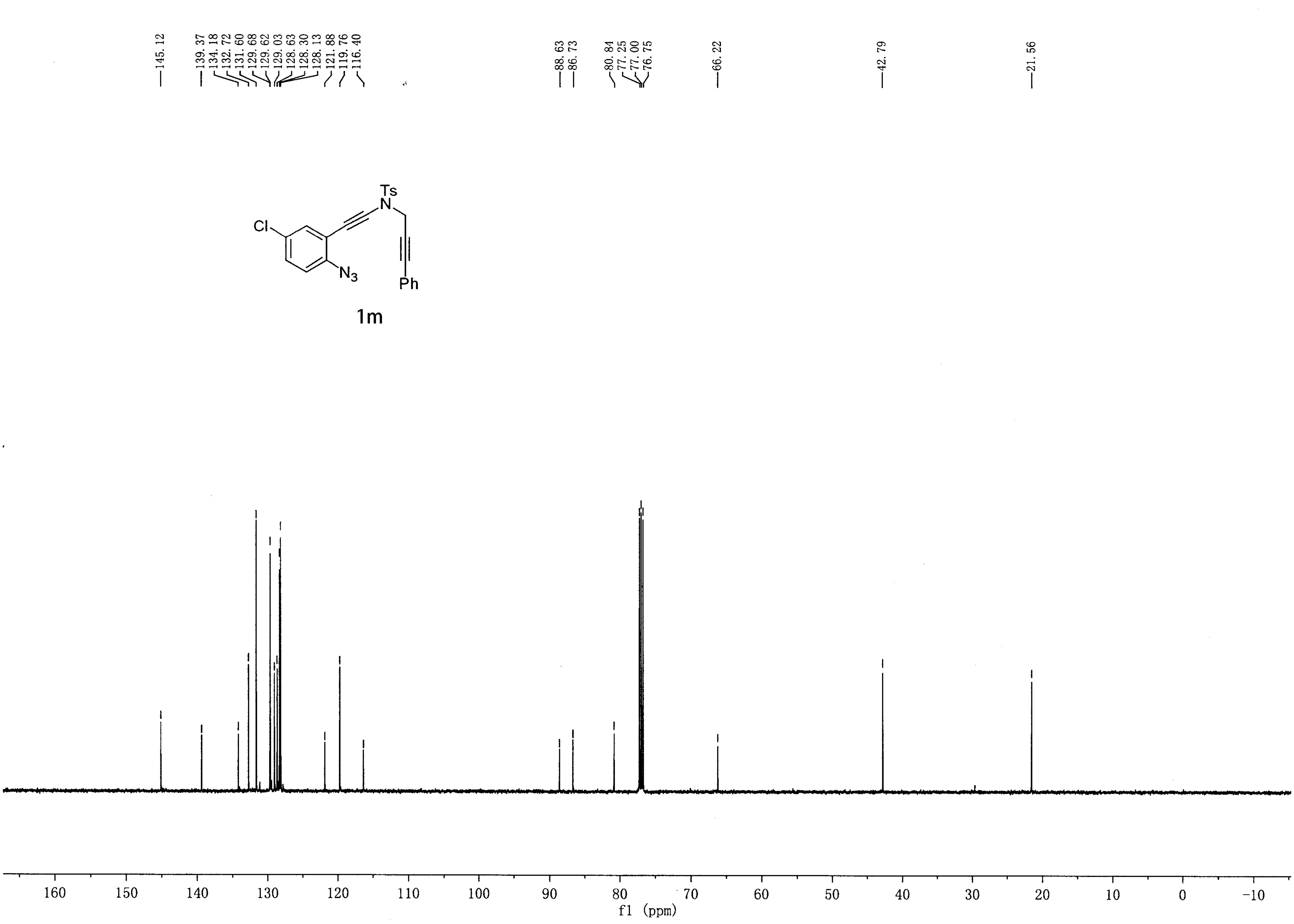

Supplementary Figure 13. ^{1}H and ^{13}C NMR spectra for 1m

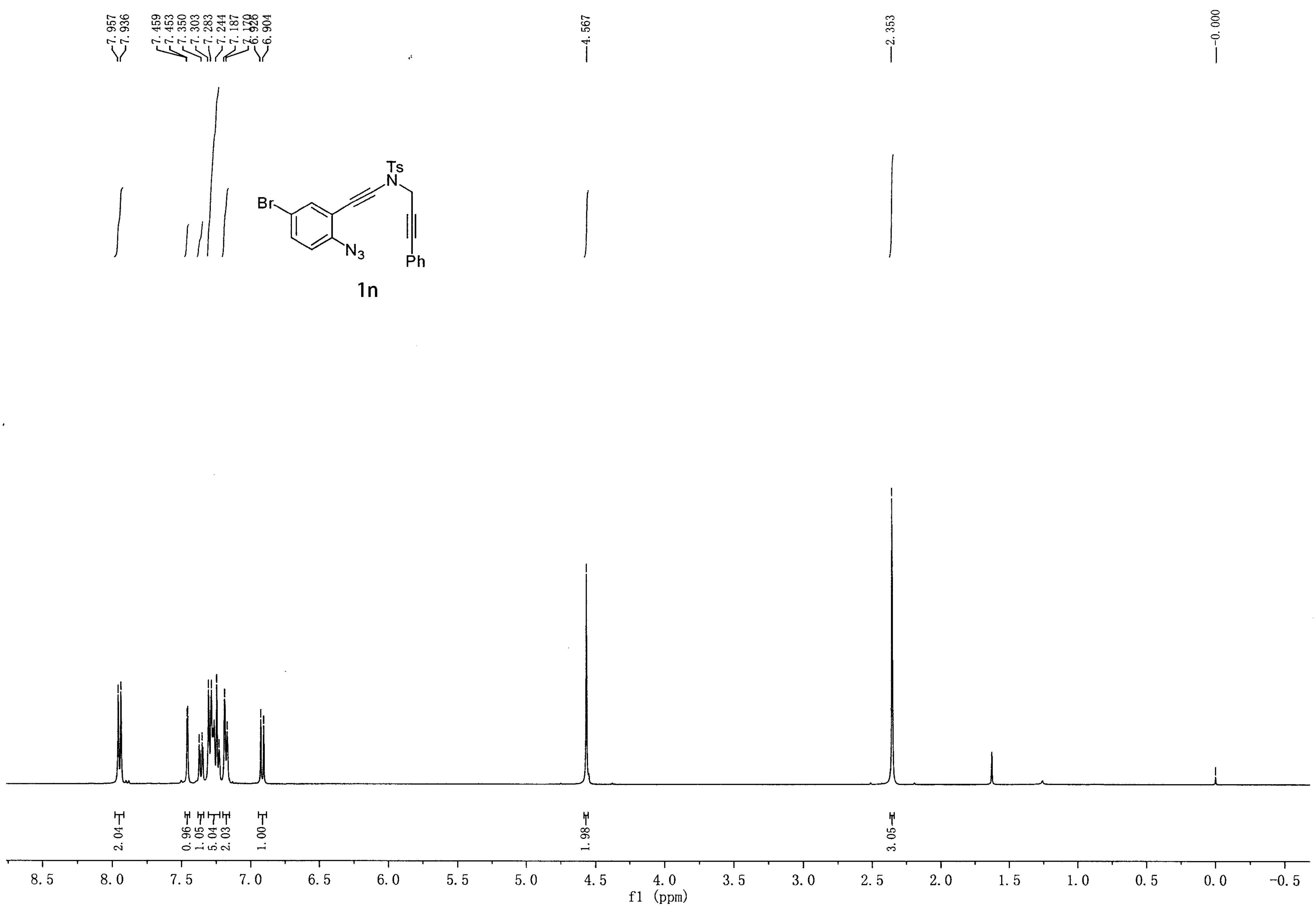

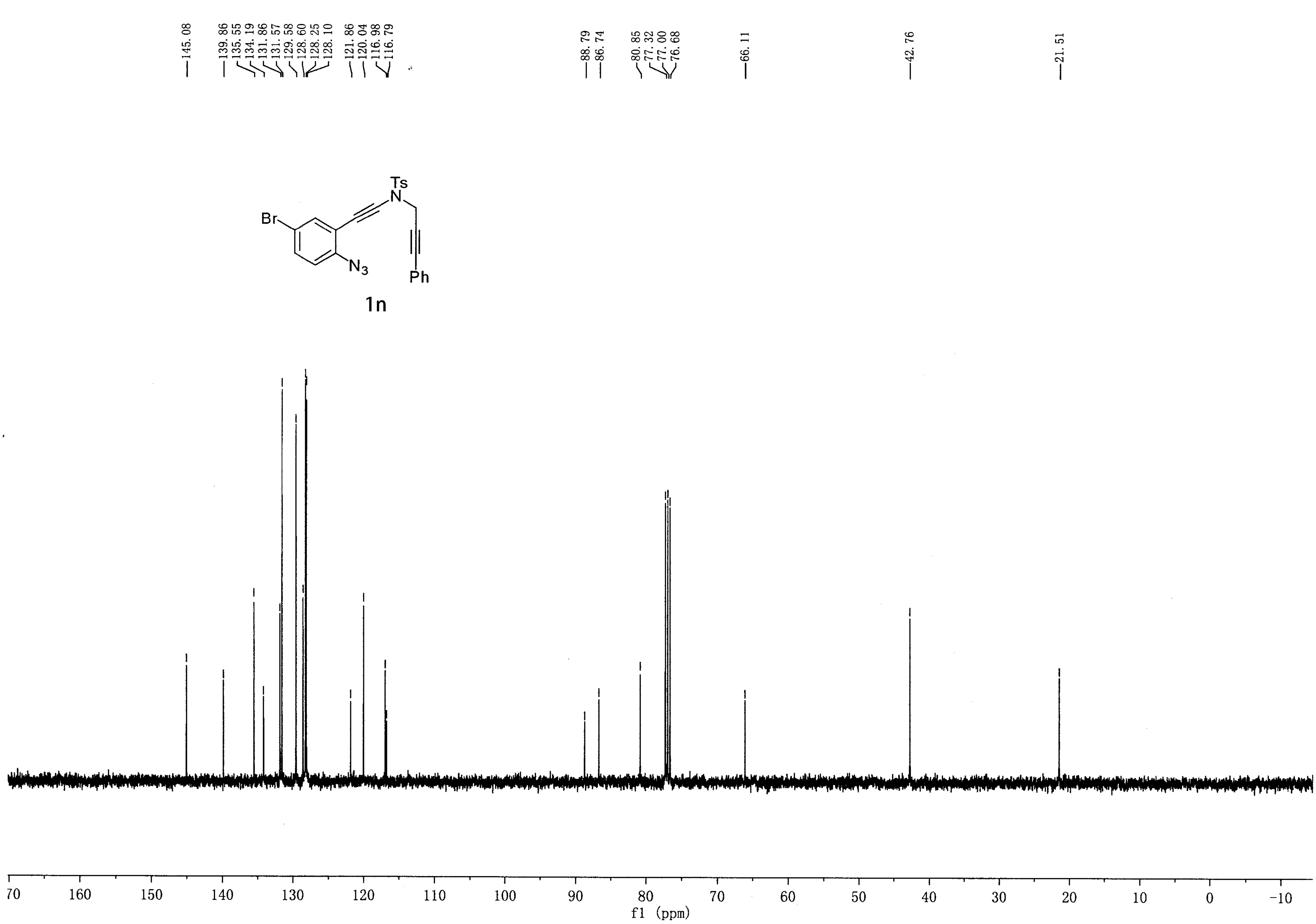

Supplementary Figure 14. ^{1}H and ^{13}C NMR spectra for 1n

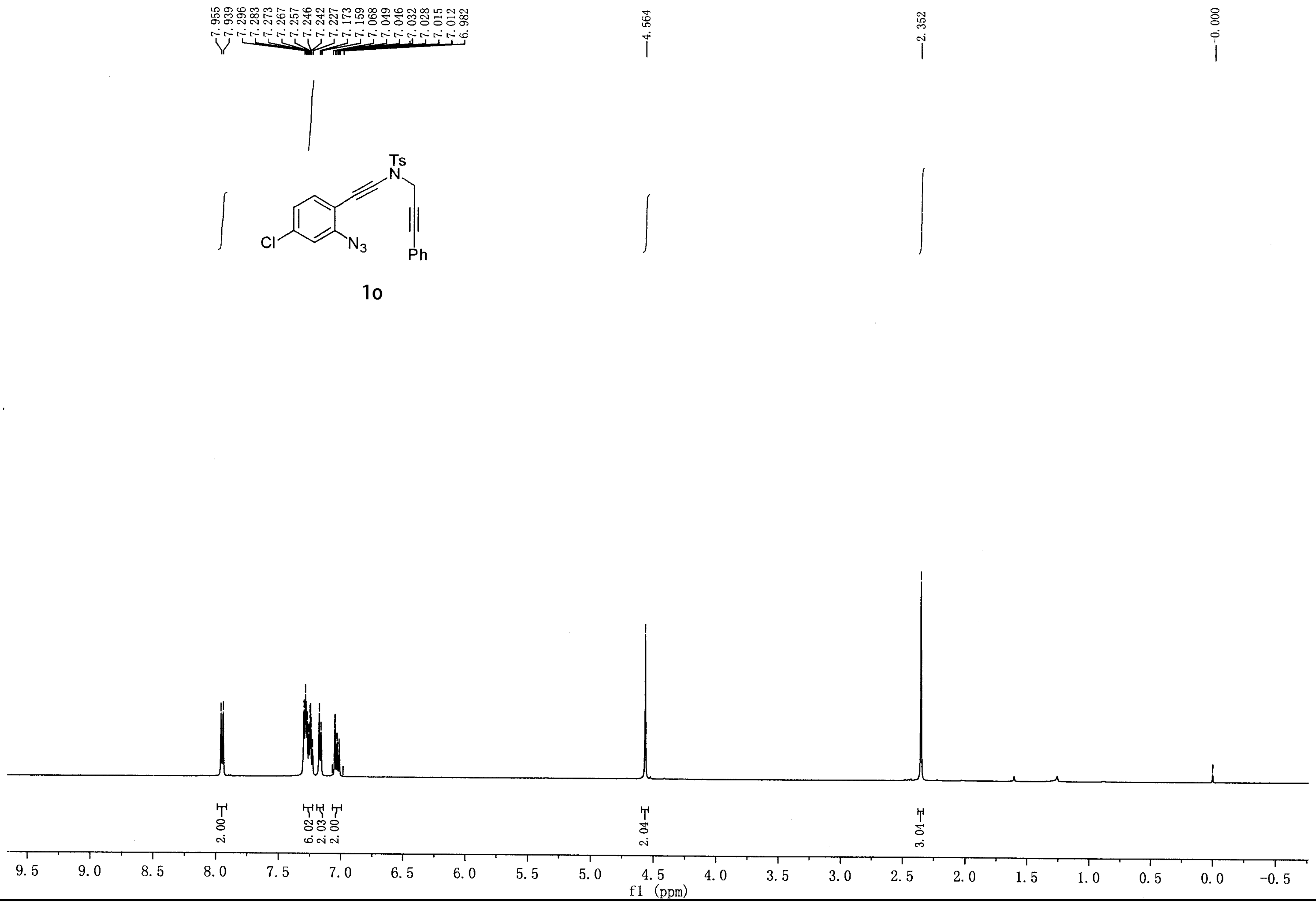

1o

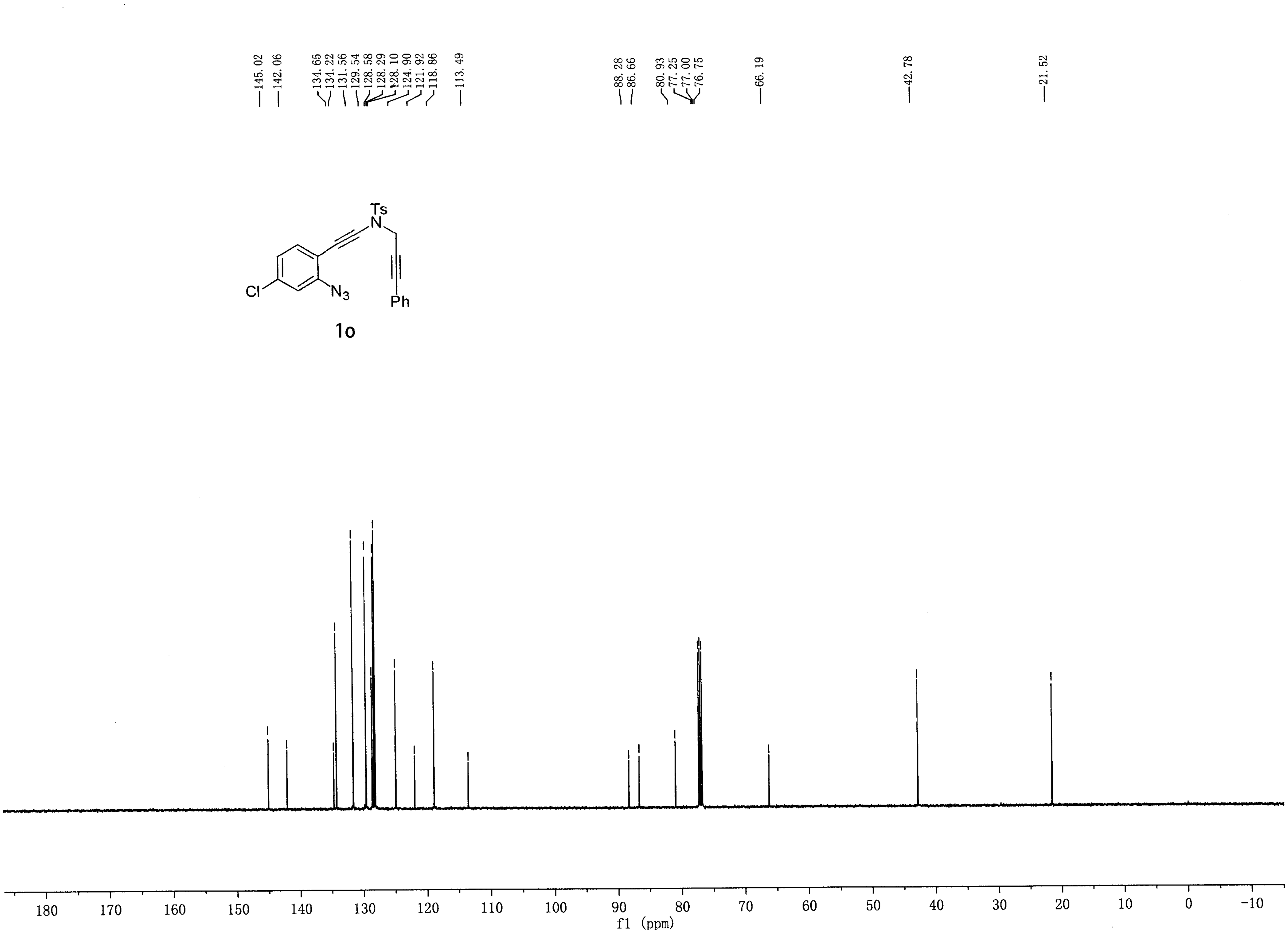

1o

Supplementary Figure 15. ^{1}H and ^{13}C NMR spectra for 1o

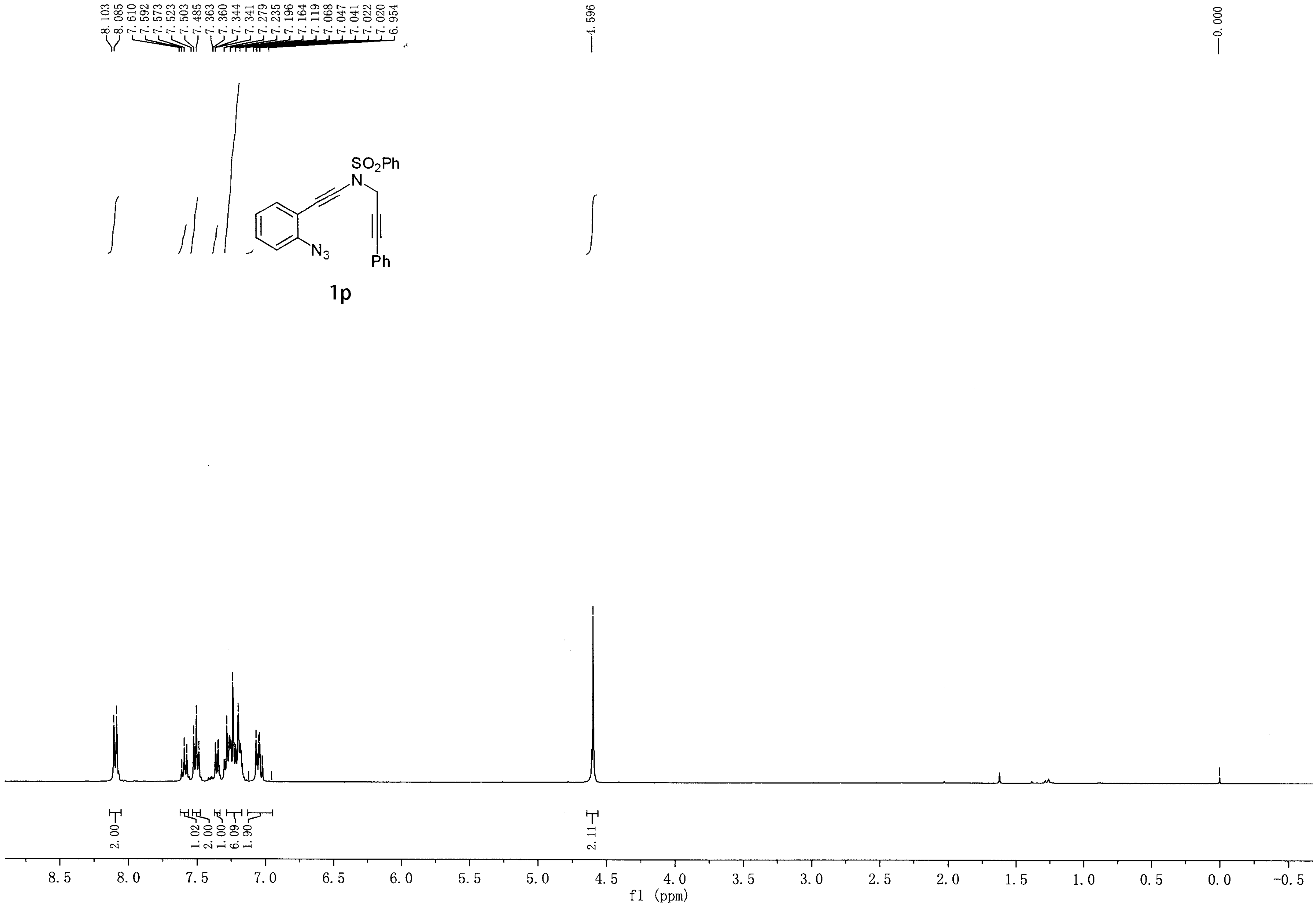

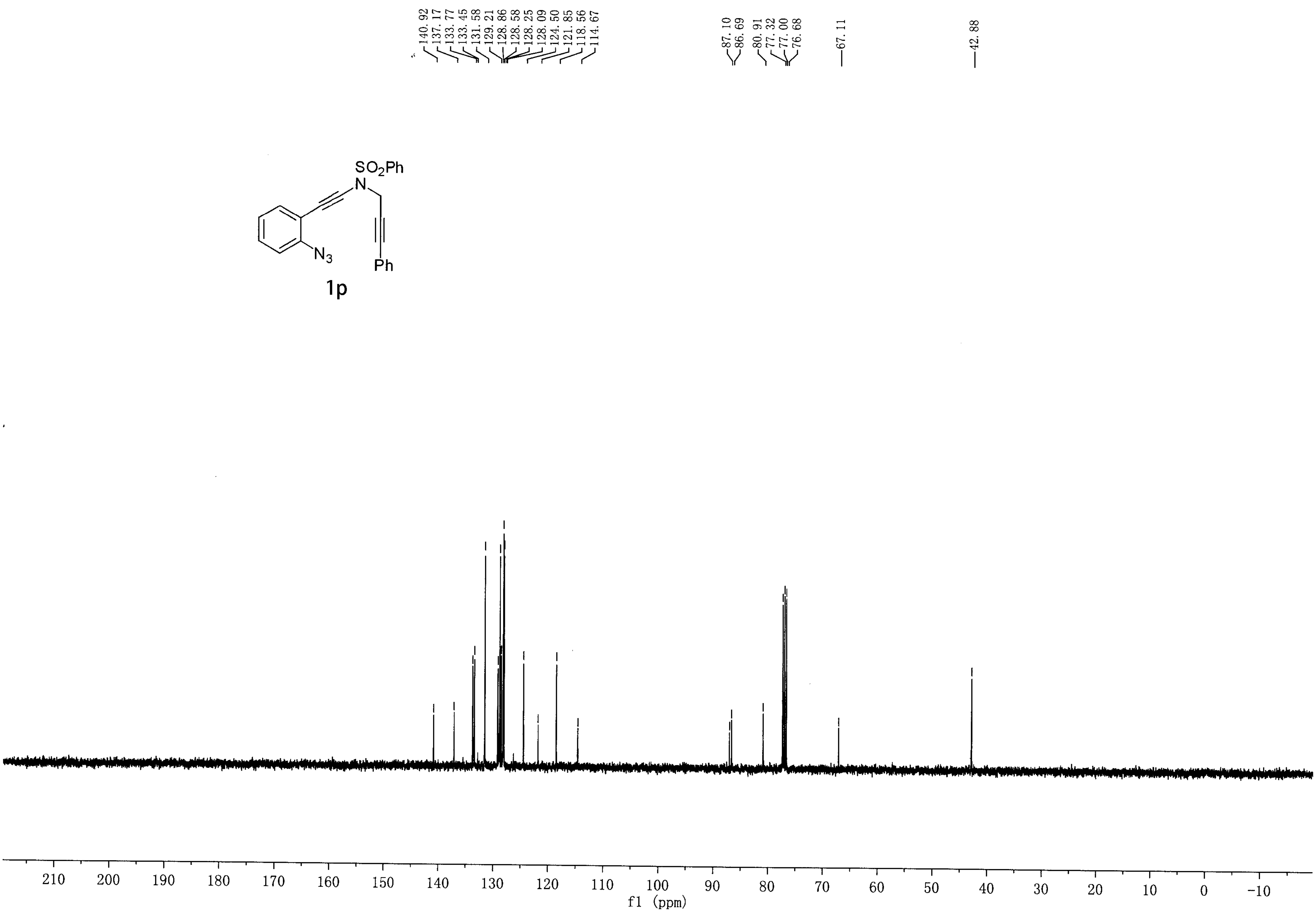

Supplementary Figure 16. ^{1}H and ^{13}C NMR spectra for 1p

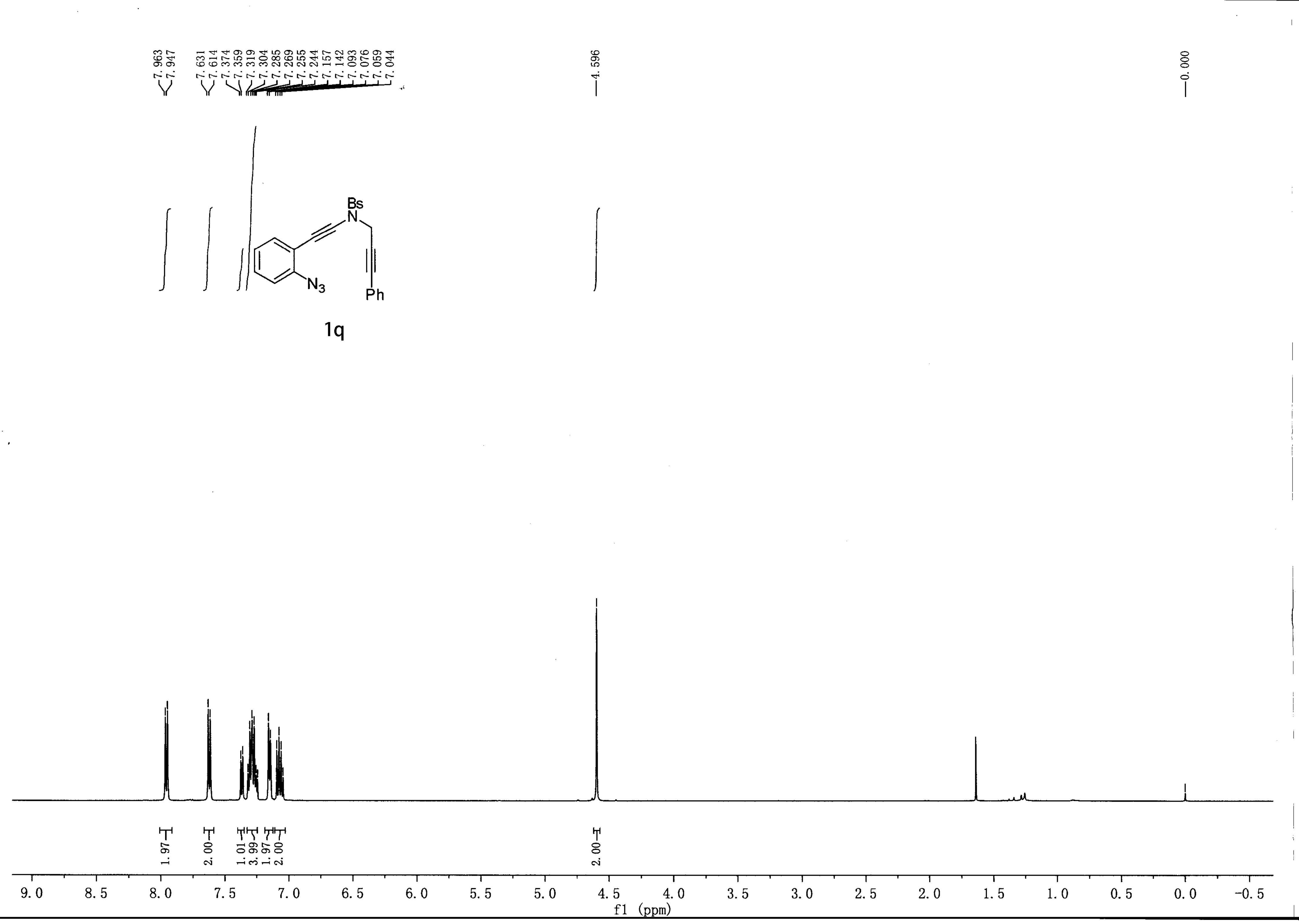

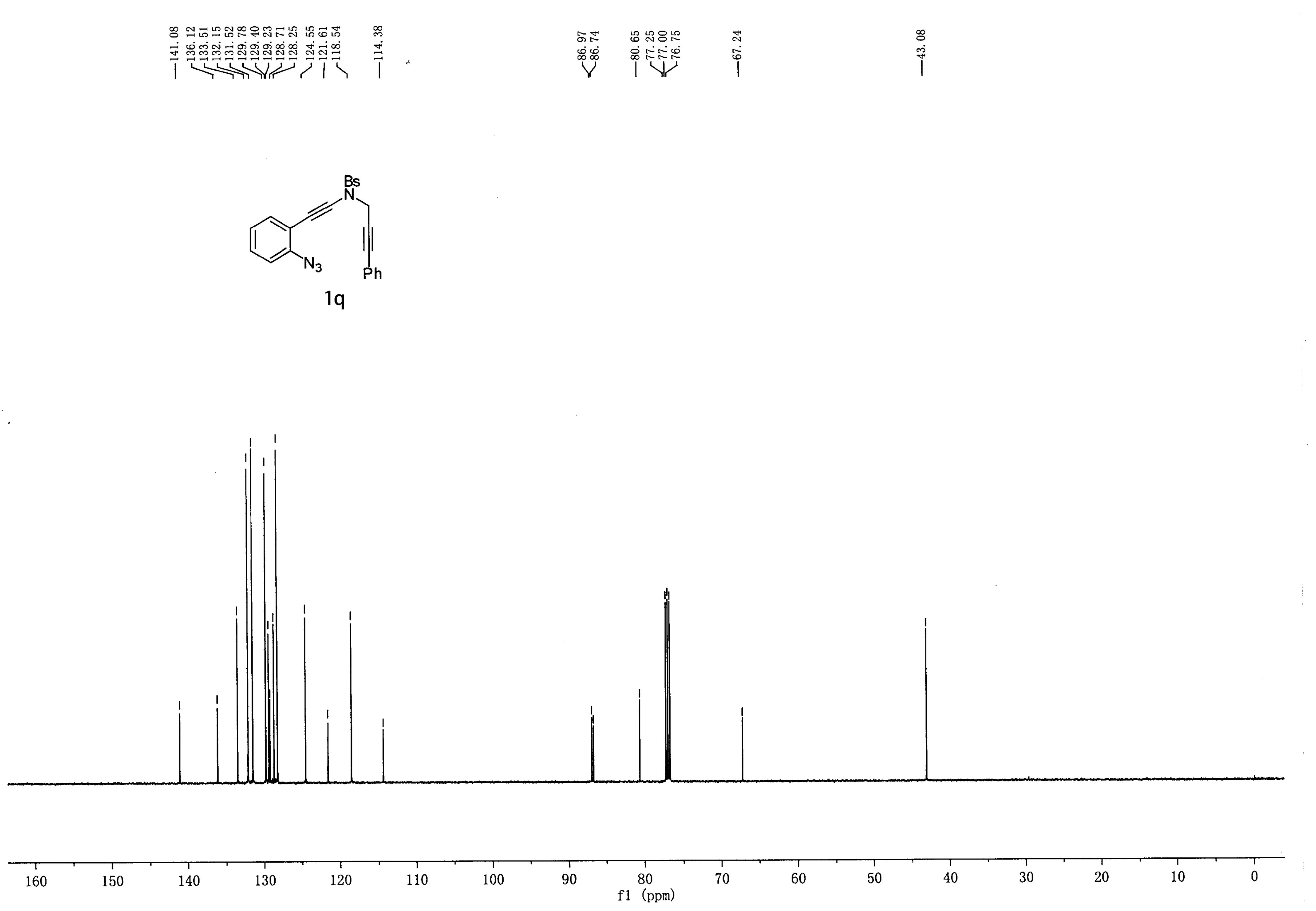

Supplementary Figure 17. ^{1}H and ^{13}C NMR spectra for 1q

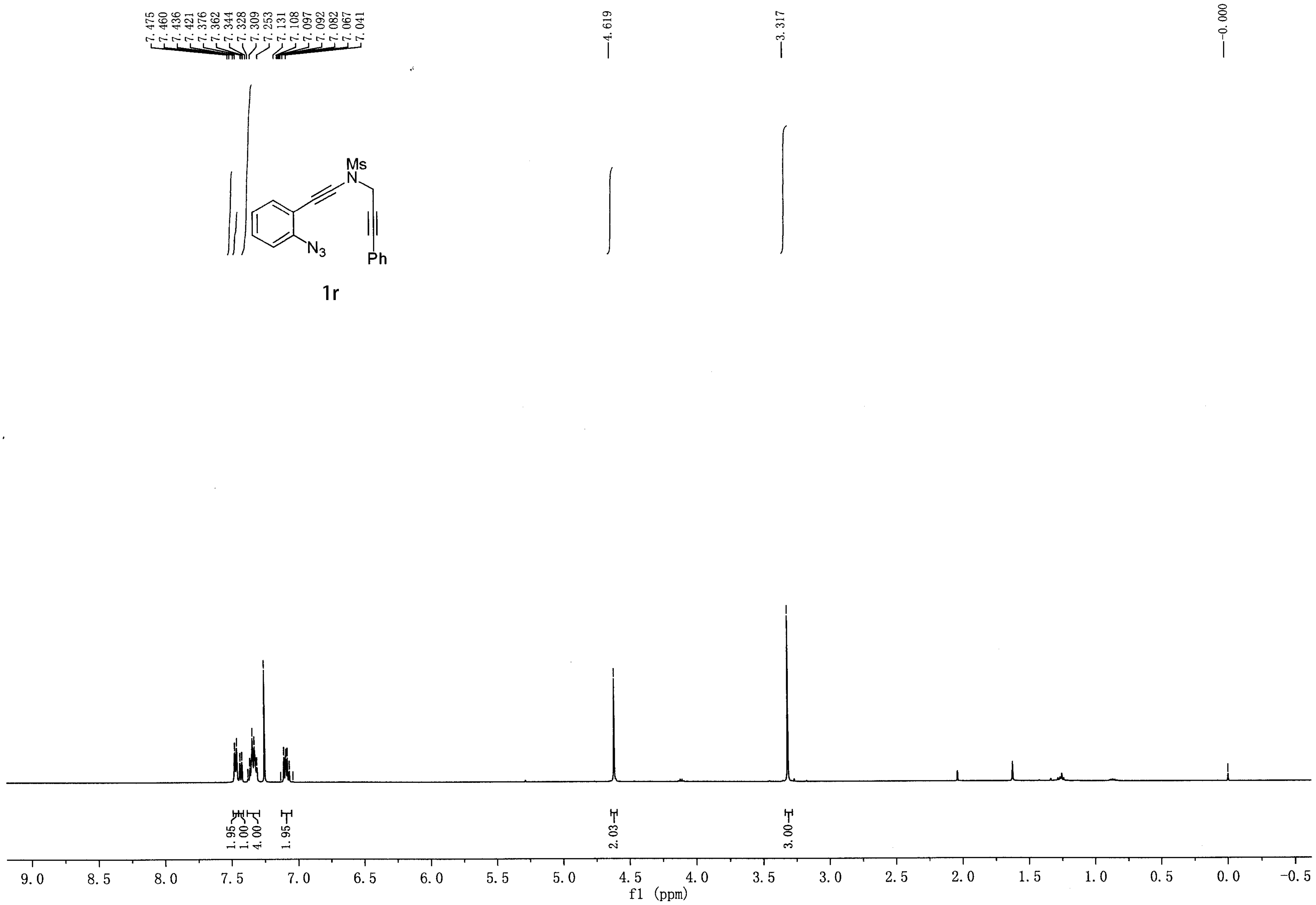

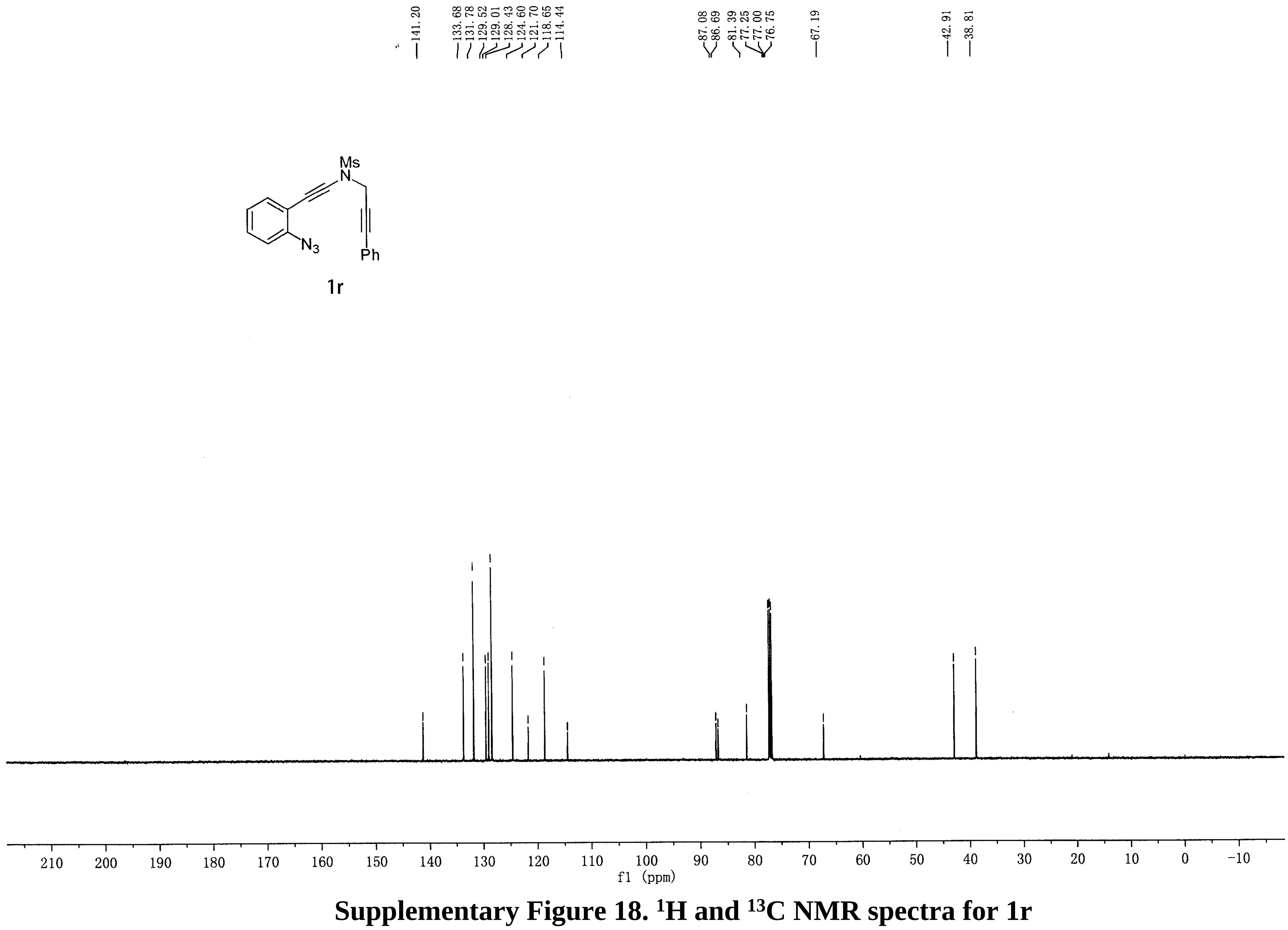

Supplementary Figure 18. ^{1}H and ^{13}C NMR spectra for 1r

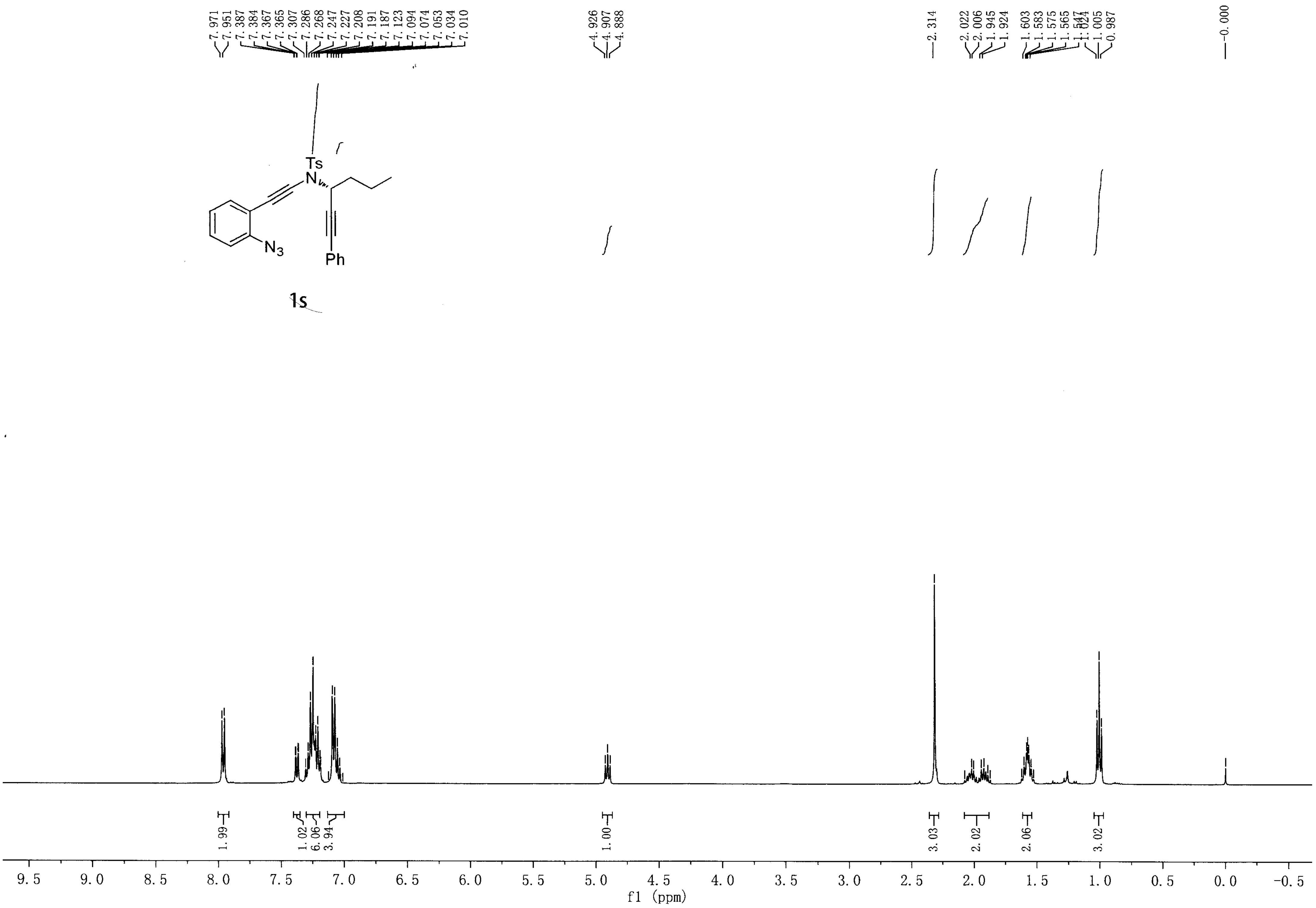

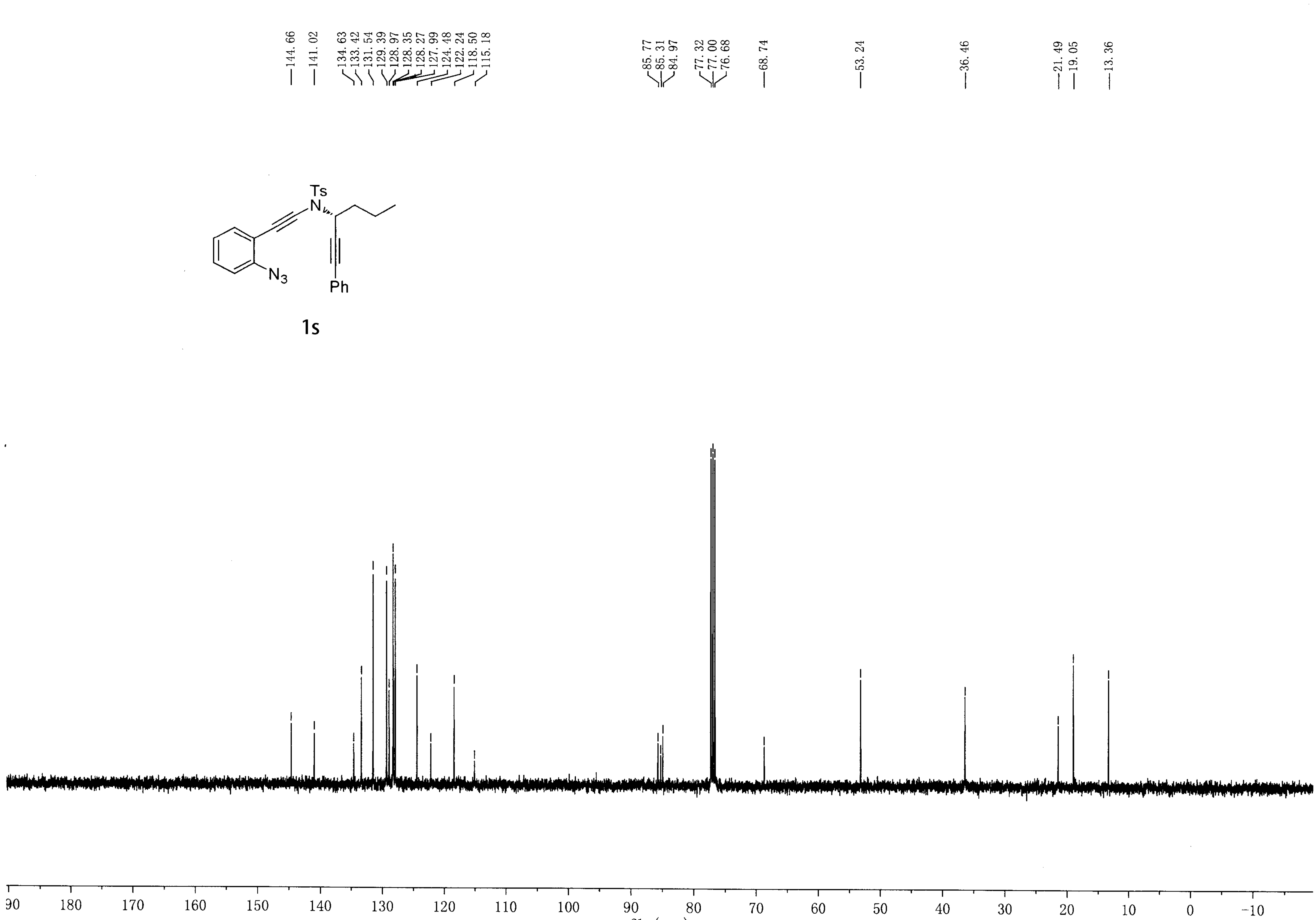

Supplementary Figure 19. ^{1}H and ^{13}C NMR spectra for 1s

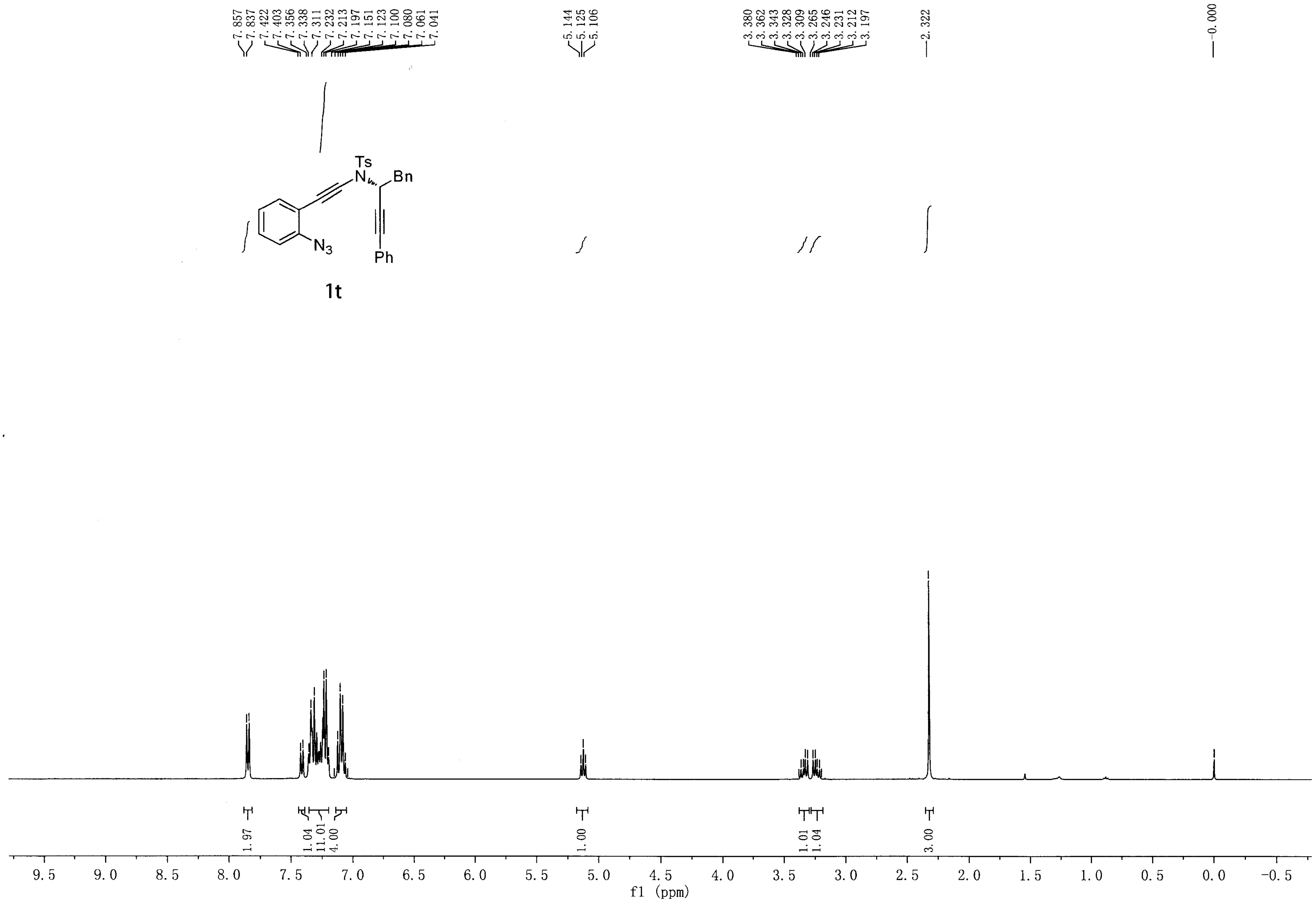

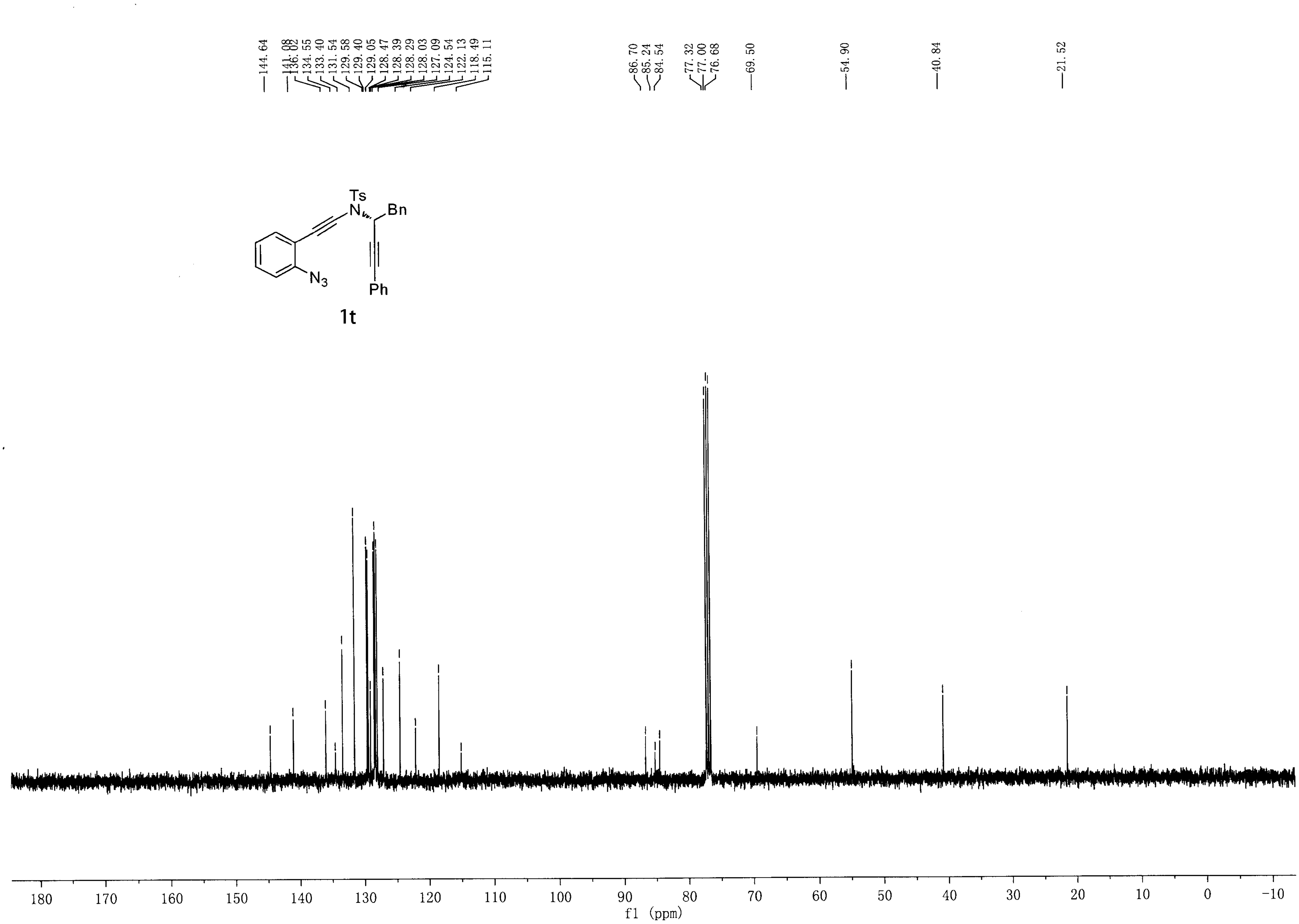

Supplementary Figure 20. ^{1}H and ^{13}C NMR spectra for 1t

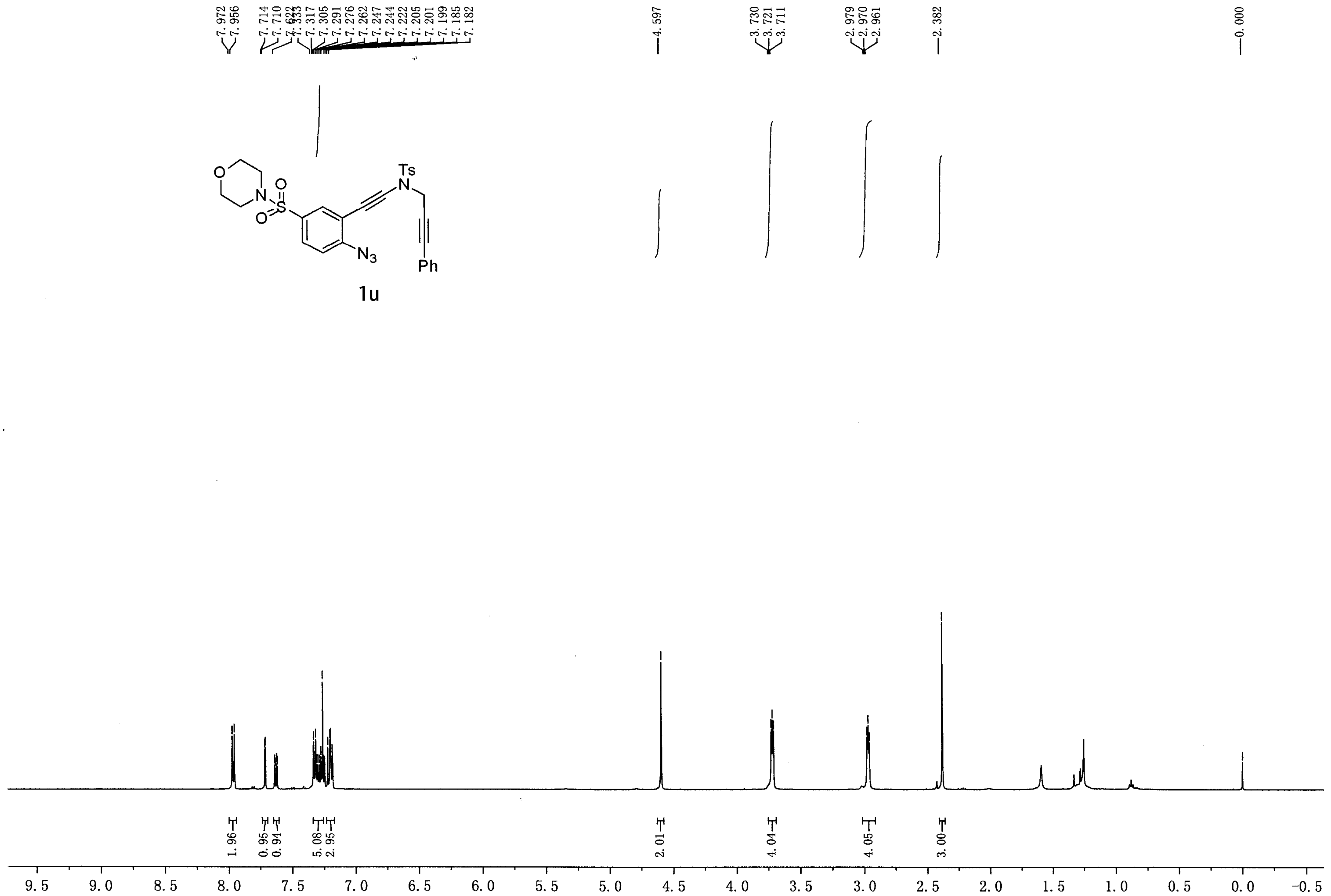

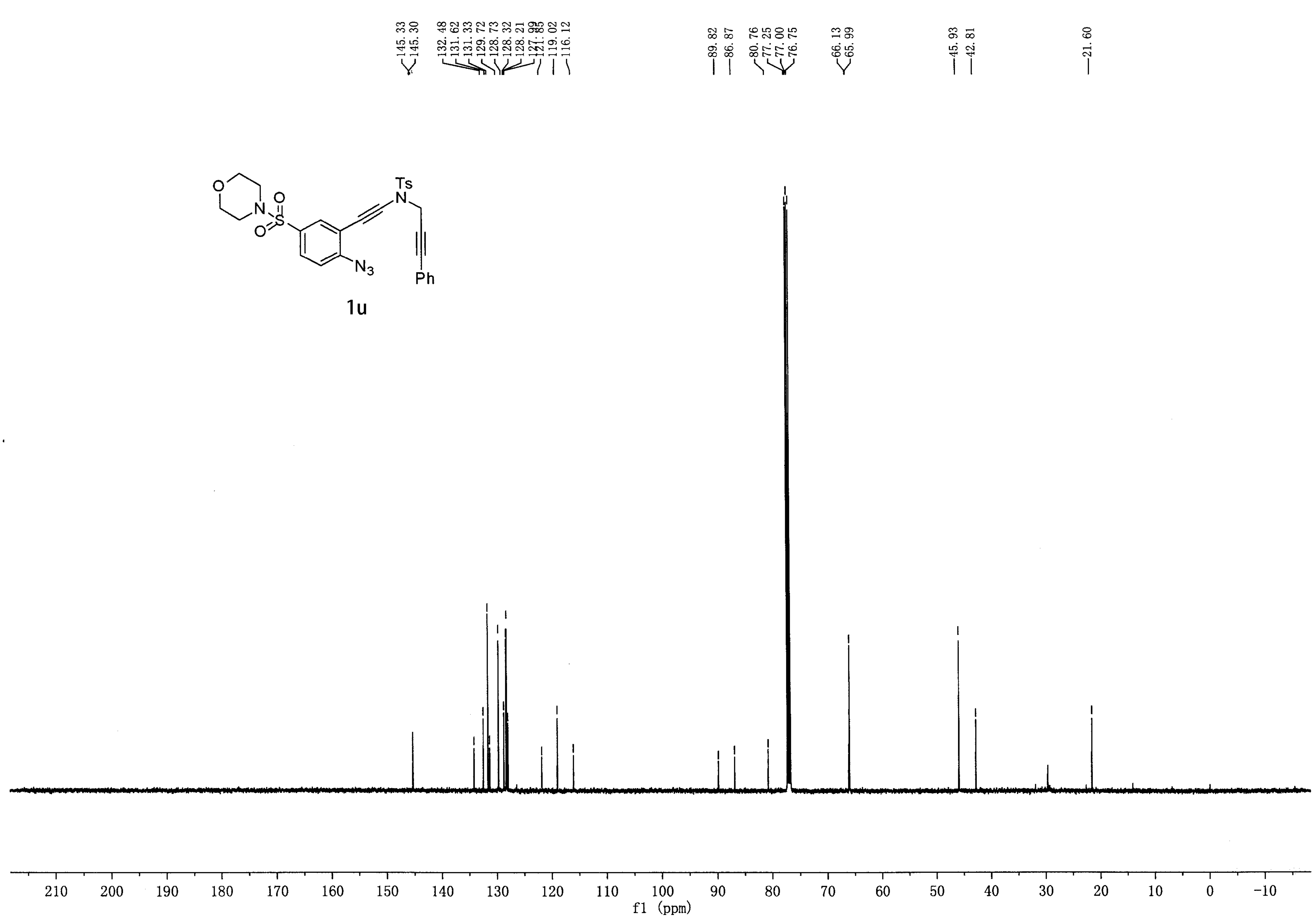

Supplementary Figure 21. ^{1}H and ^{13}C NMR spectra for 1u

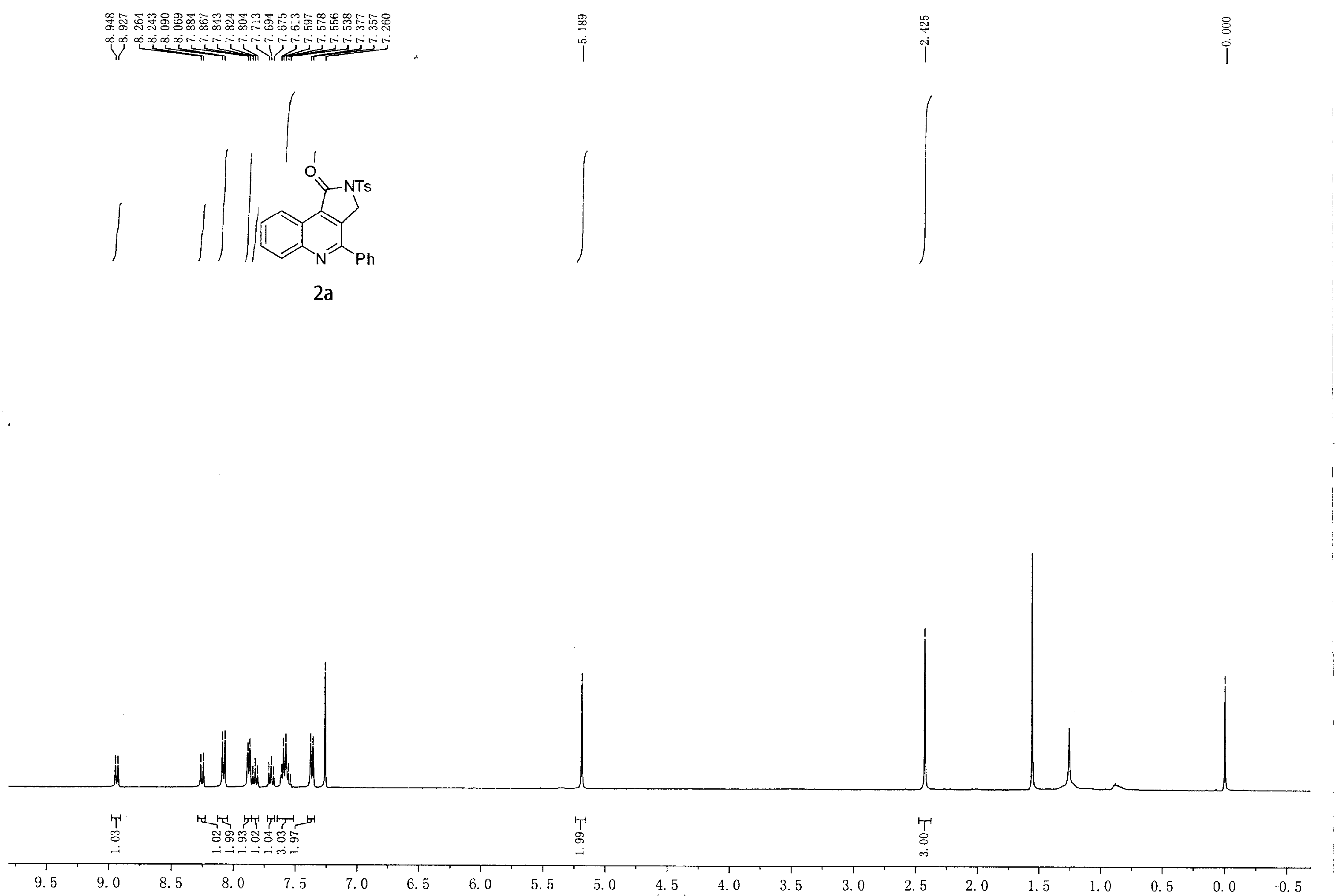

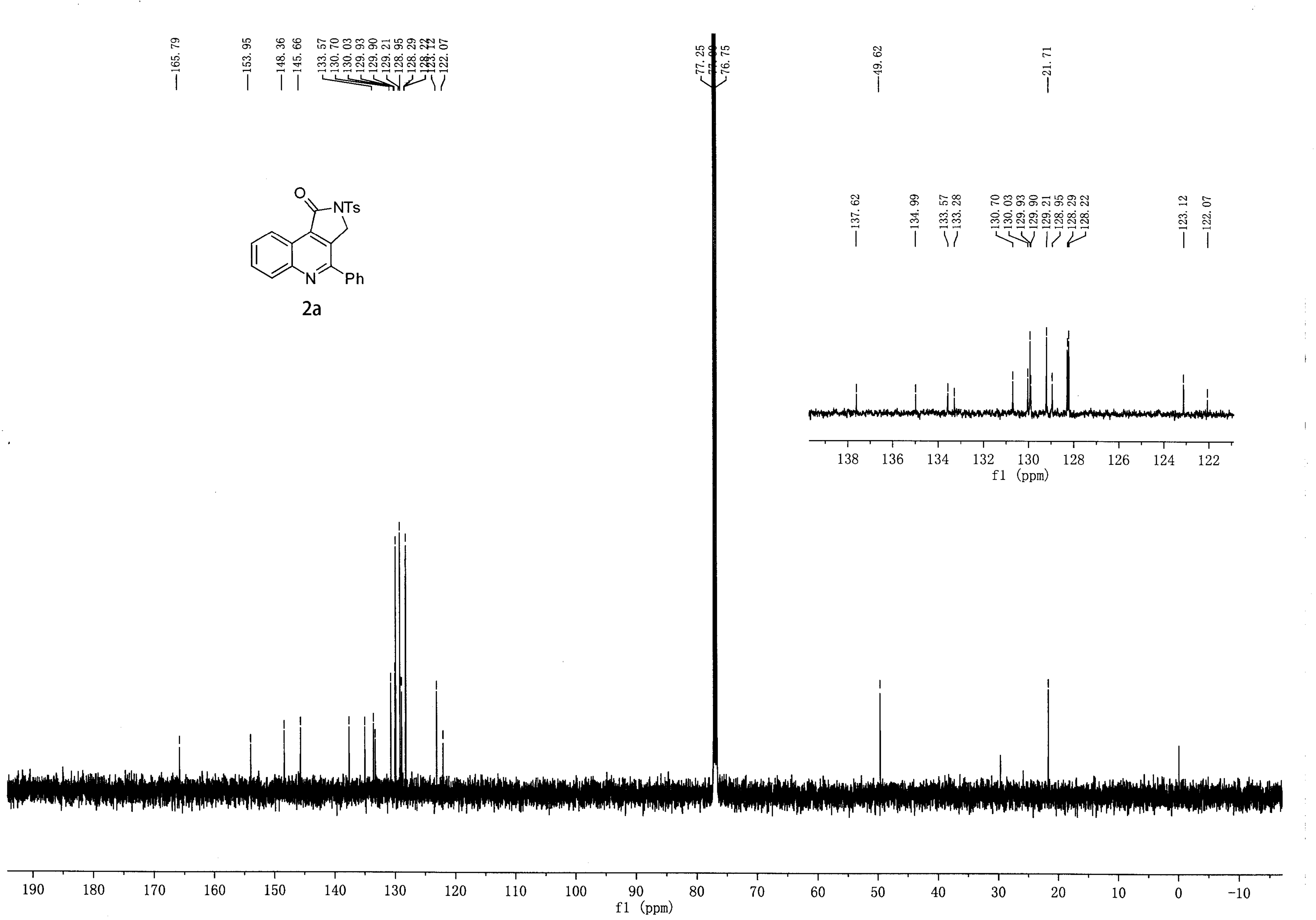

Supplementary Figure 22. ^{1}H and ^{13}C NMR spectra for 2a

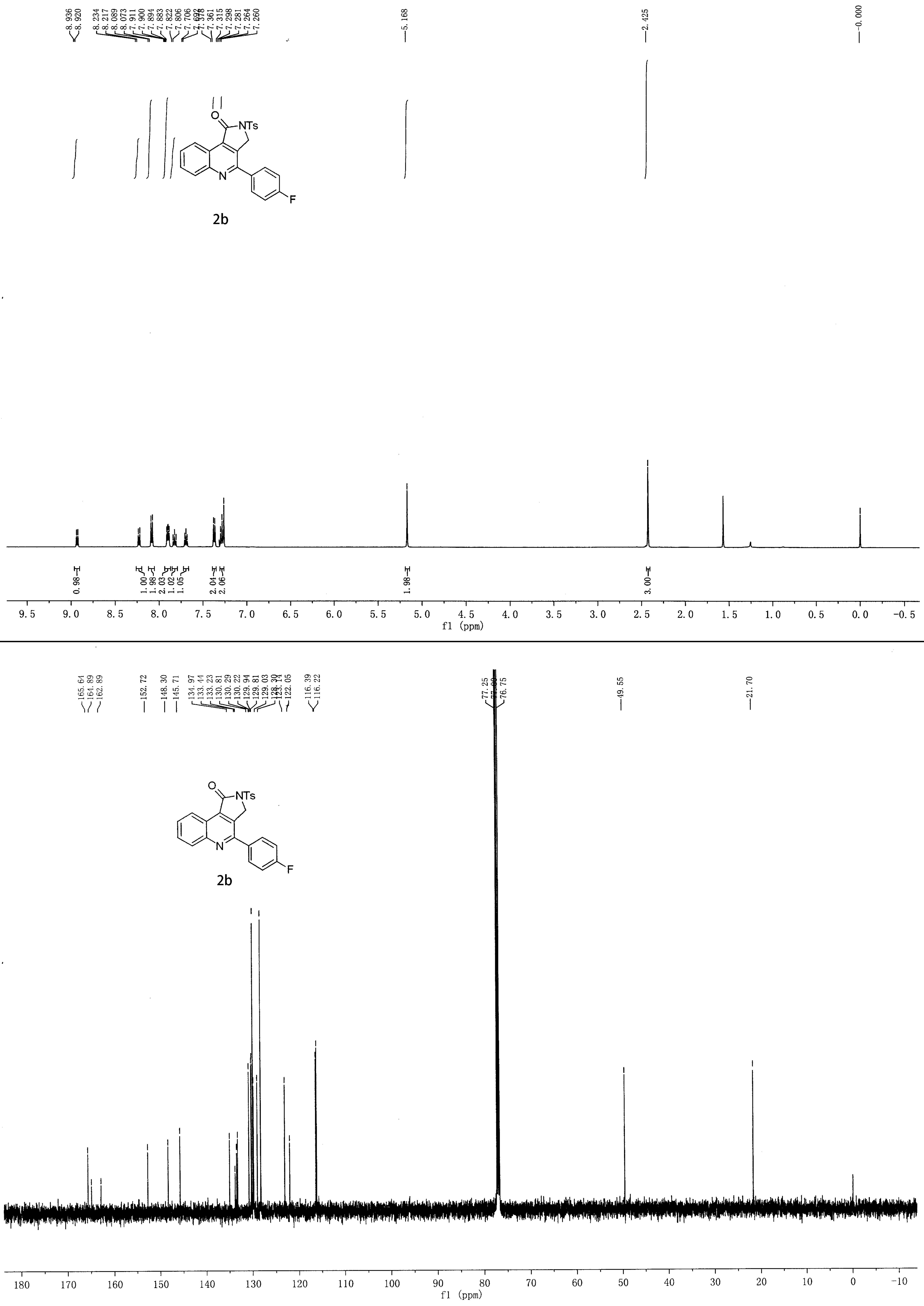

Supplementary Figure 23. ^{1}H and ^{13}C NMR spectra for 2b

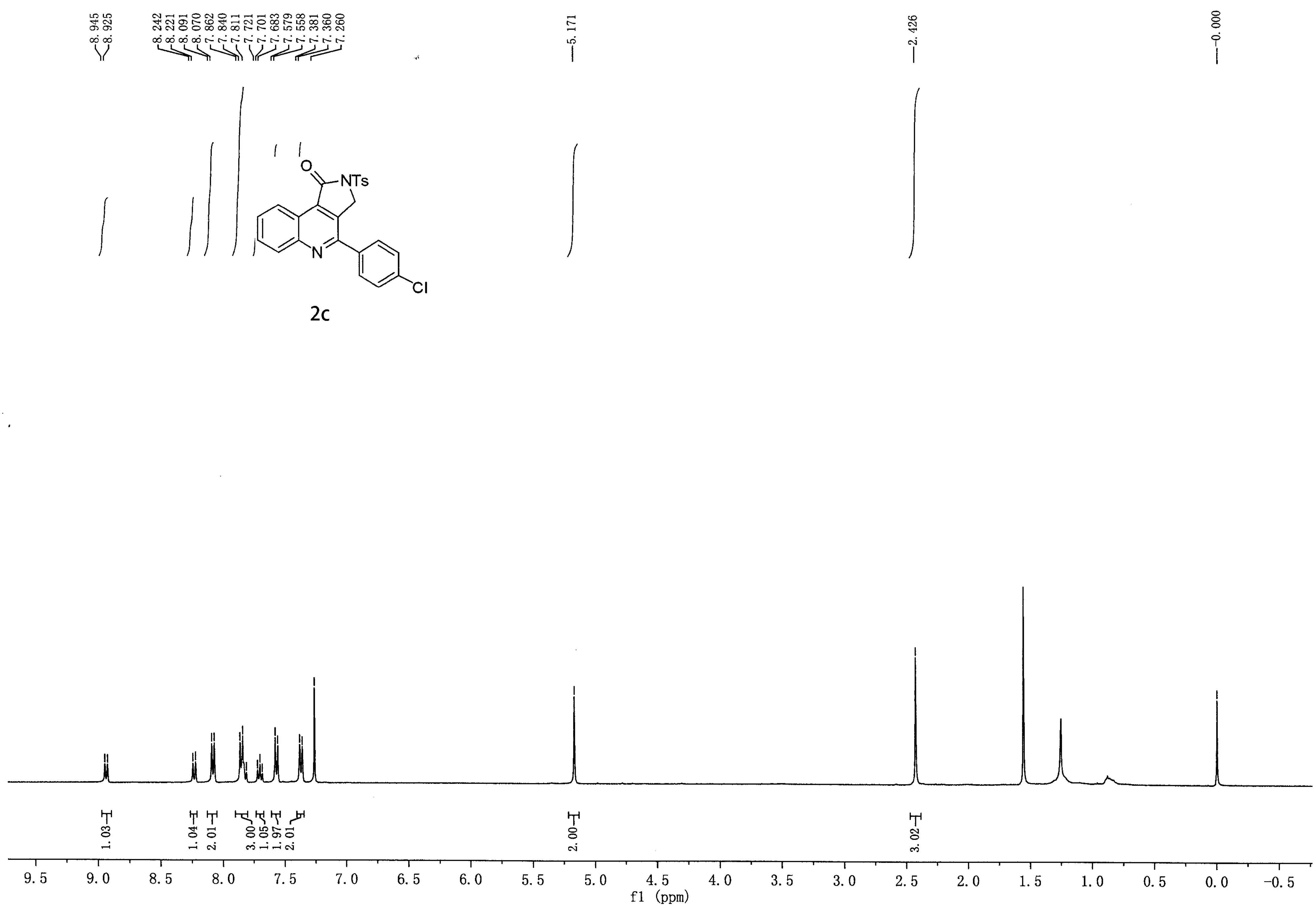

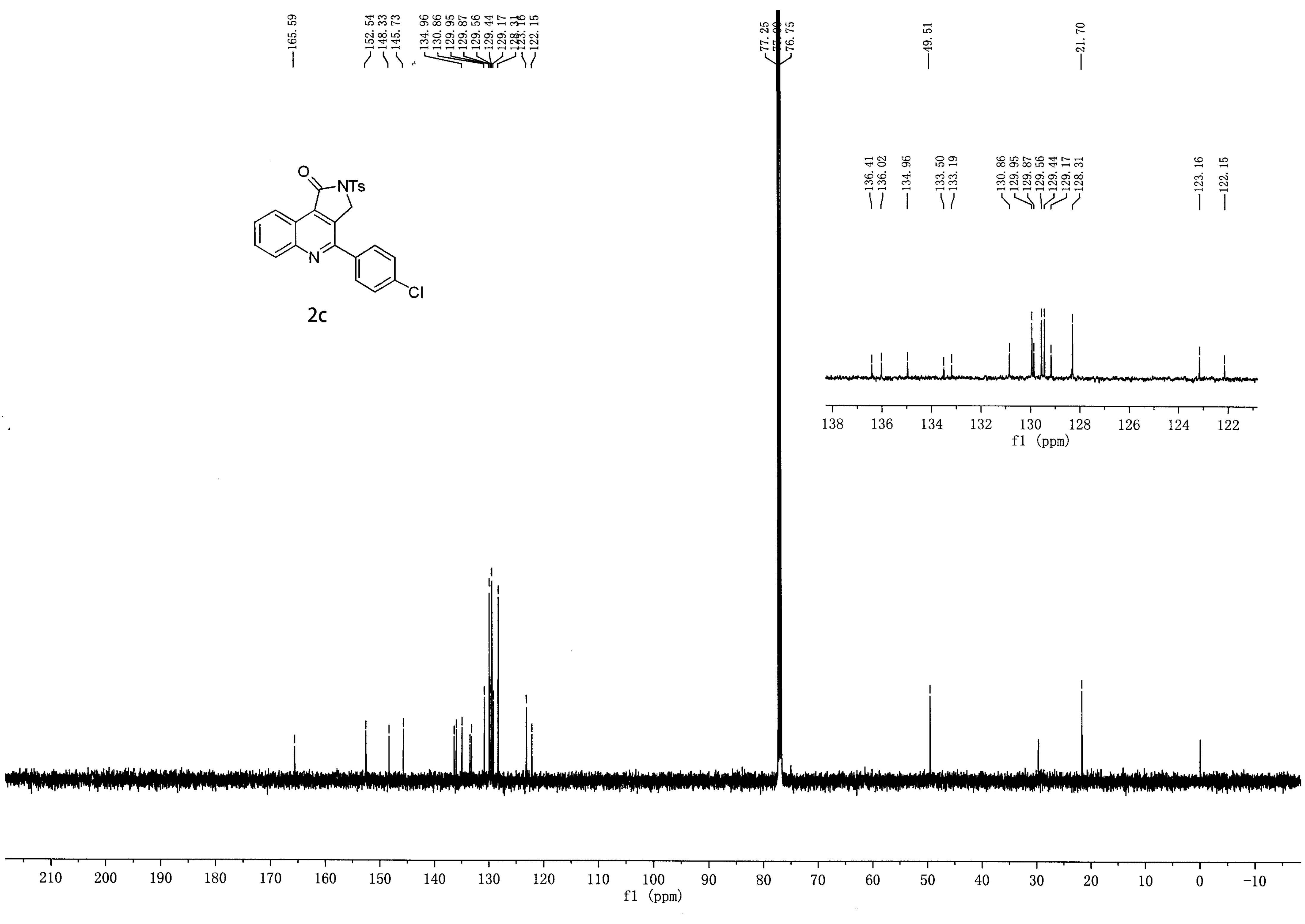

Supplementary Figure 24. ^{1}H and ^{13}C NMR spectra for 2c

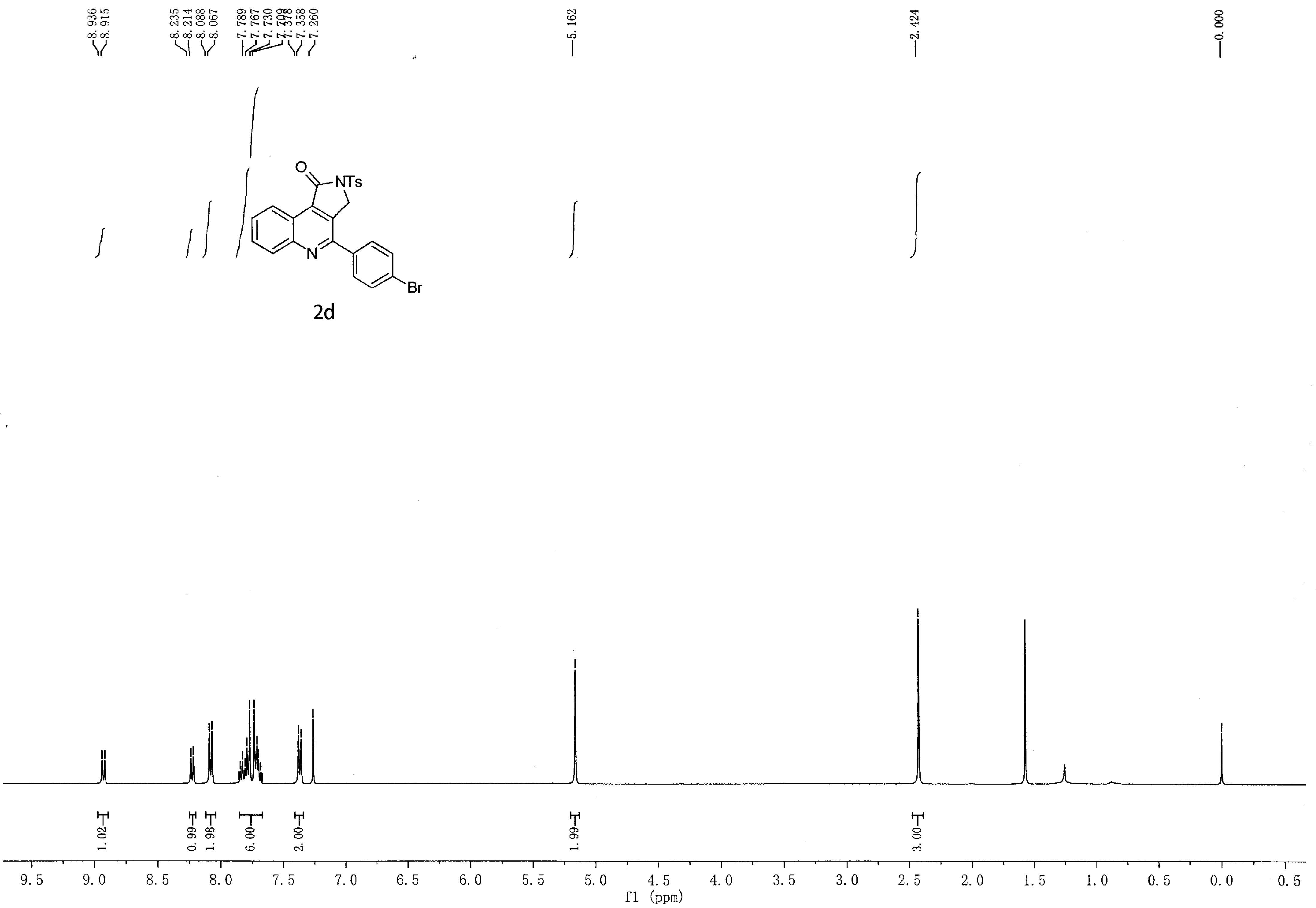

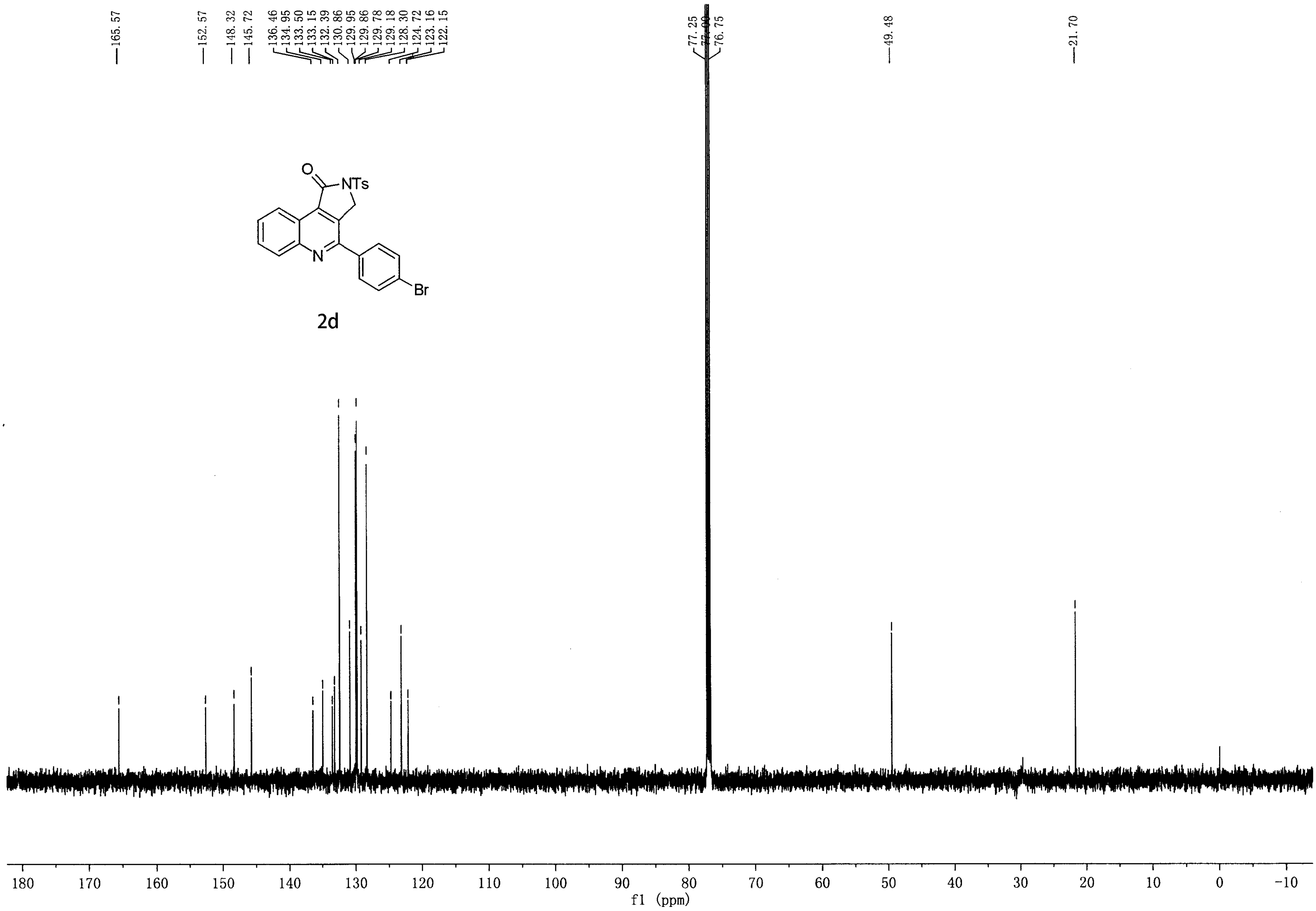

Supplementary Figure 25. ^{1}H and ^{13}C NMR spectra for 2d

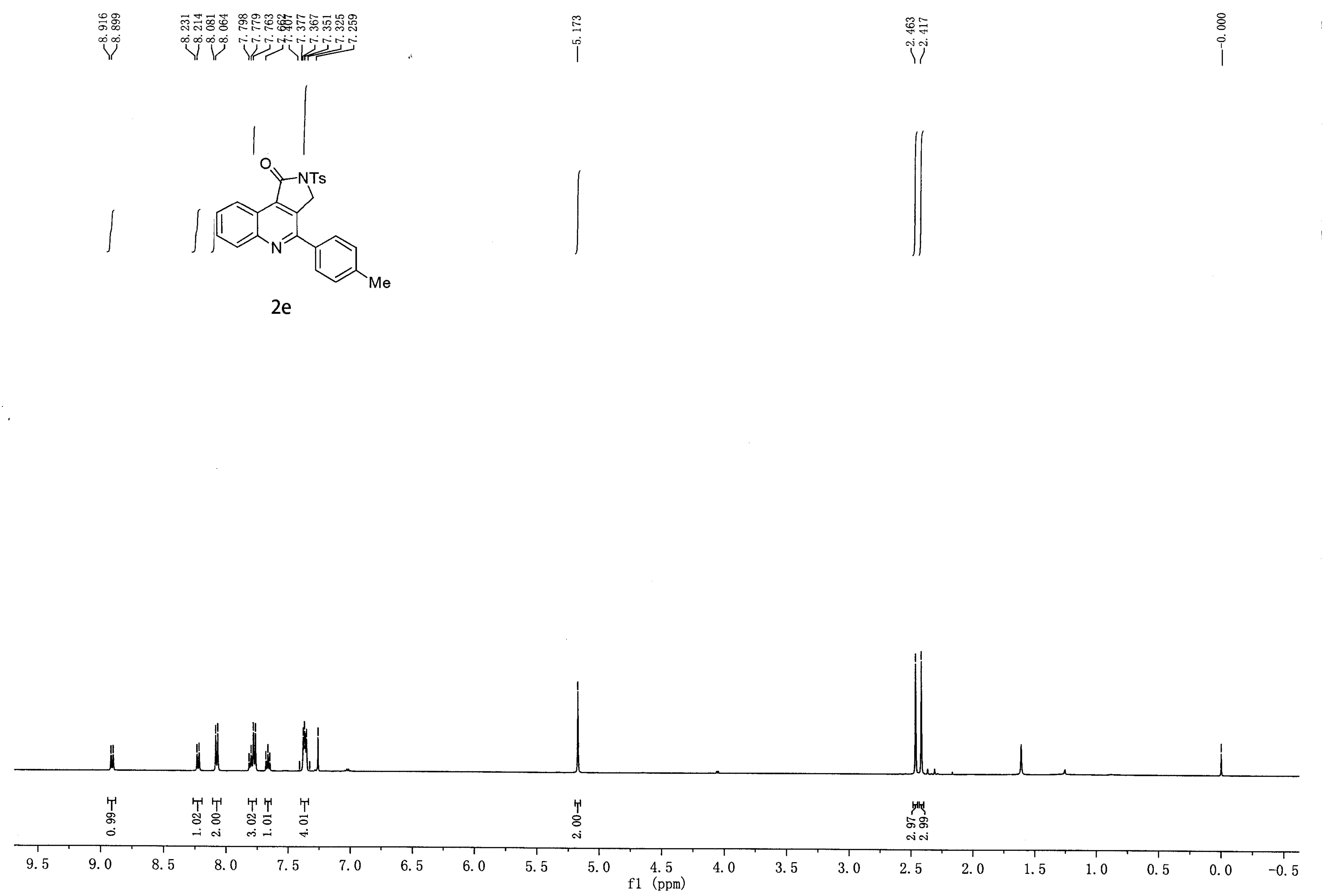

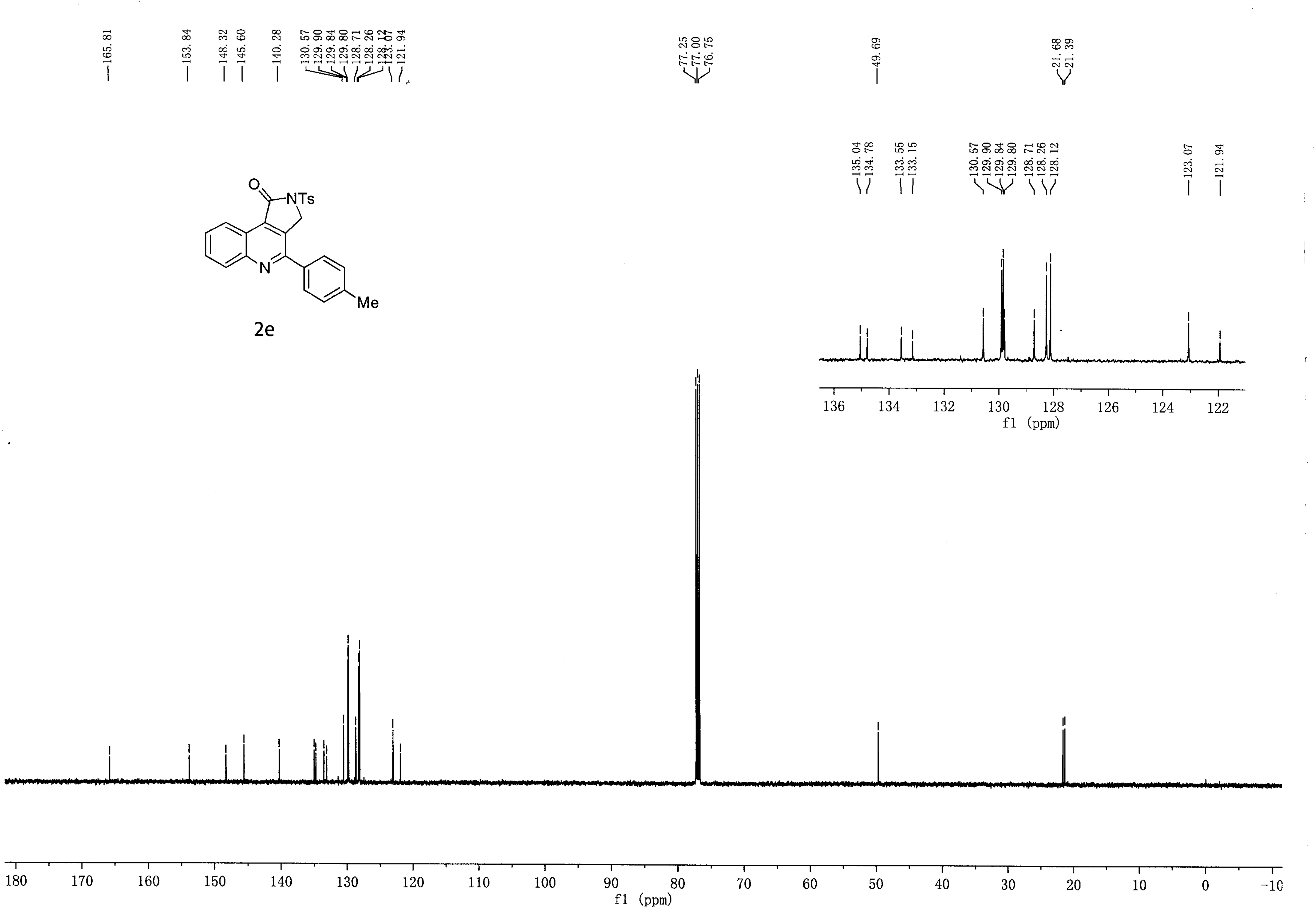

Supplementary Figure 26. ^{1}H and ^{13}C NMR spectra for 2e

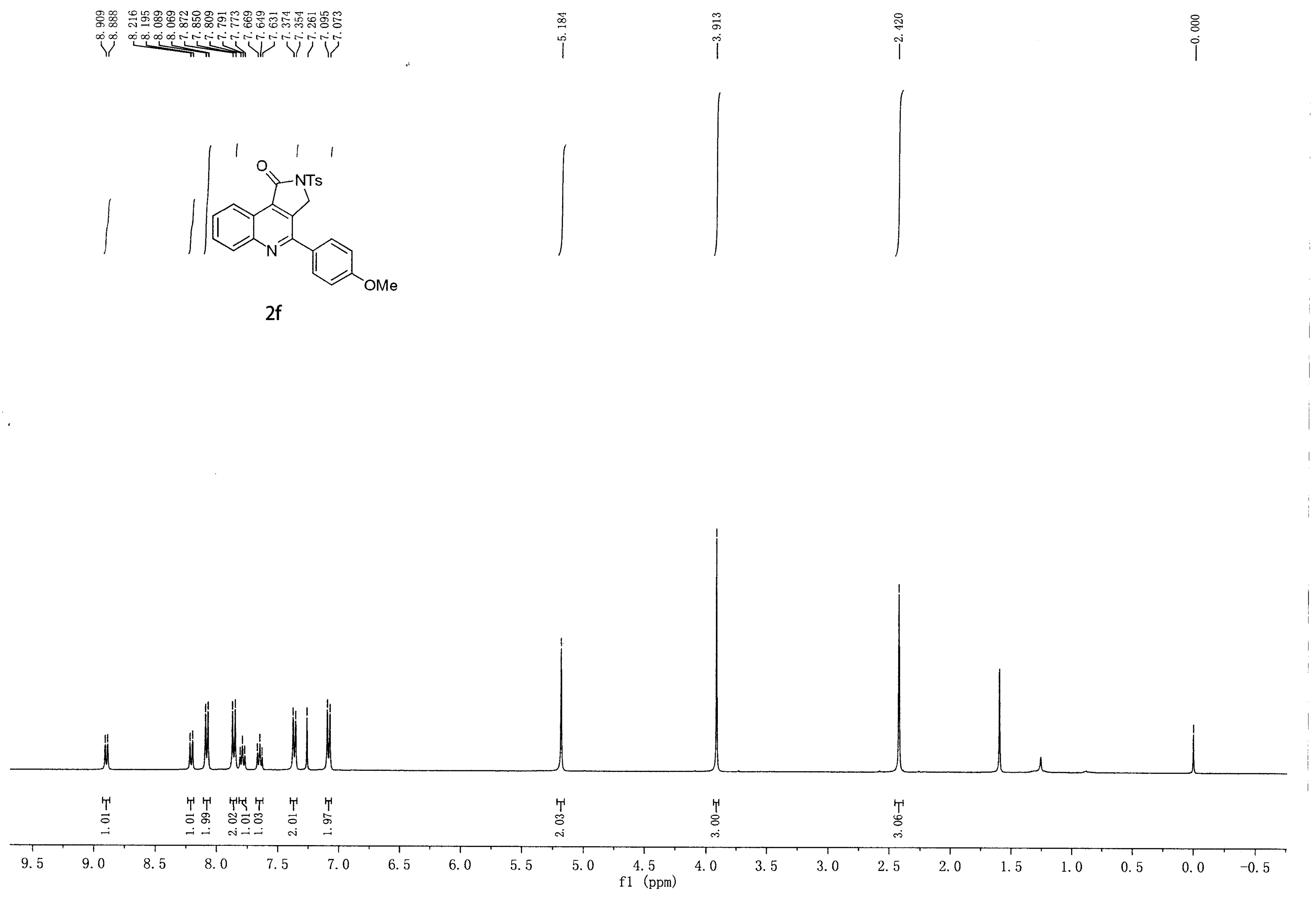

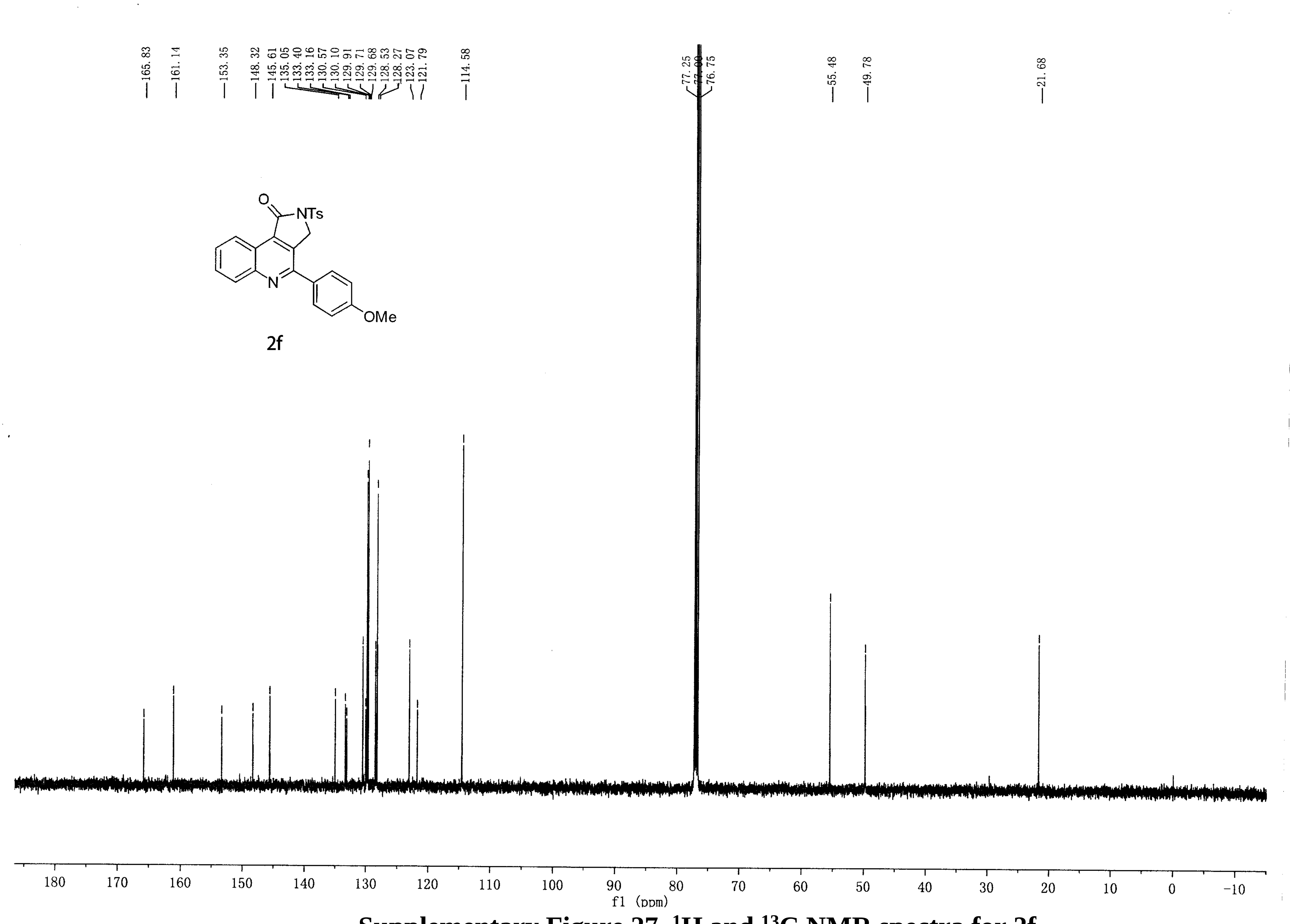

Supplementary Figure 27. ^1H and ^{13}C NMR spectra for 2f

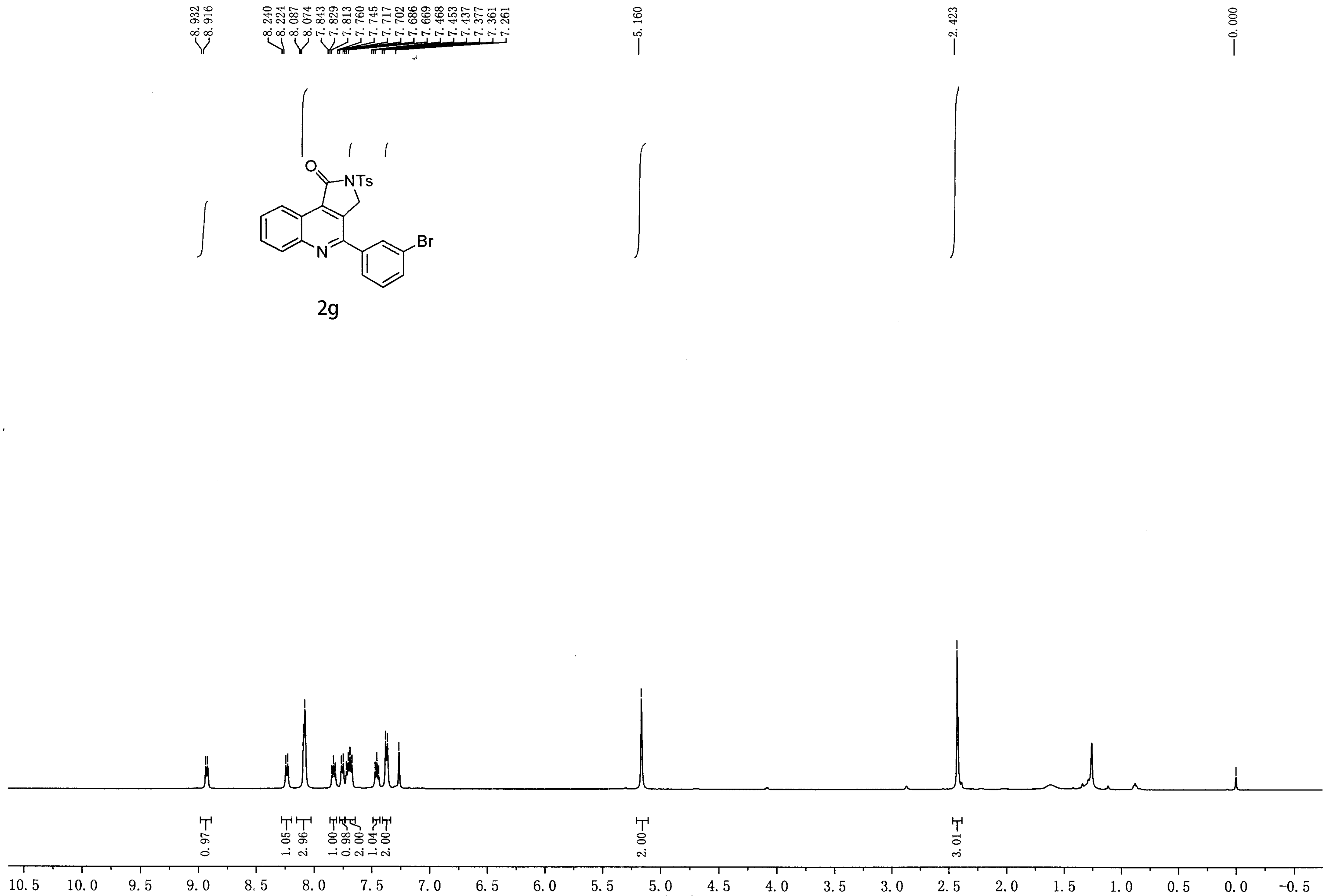

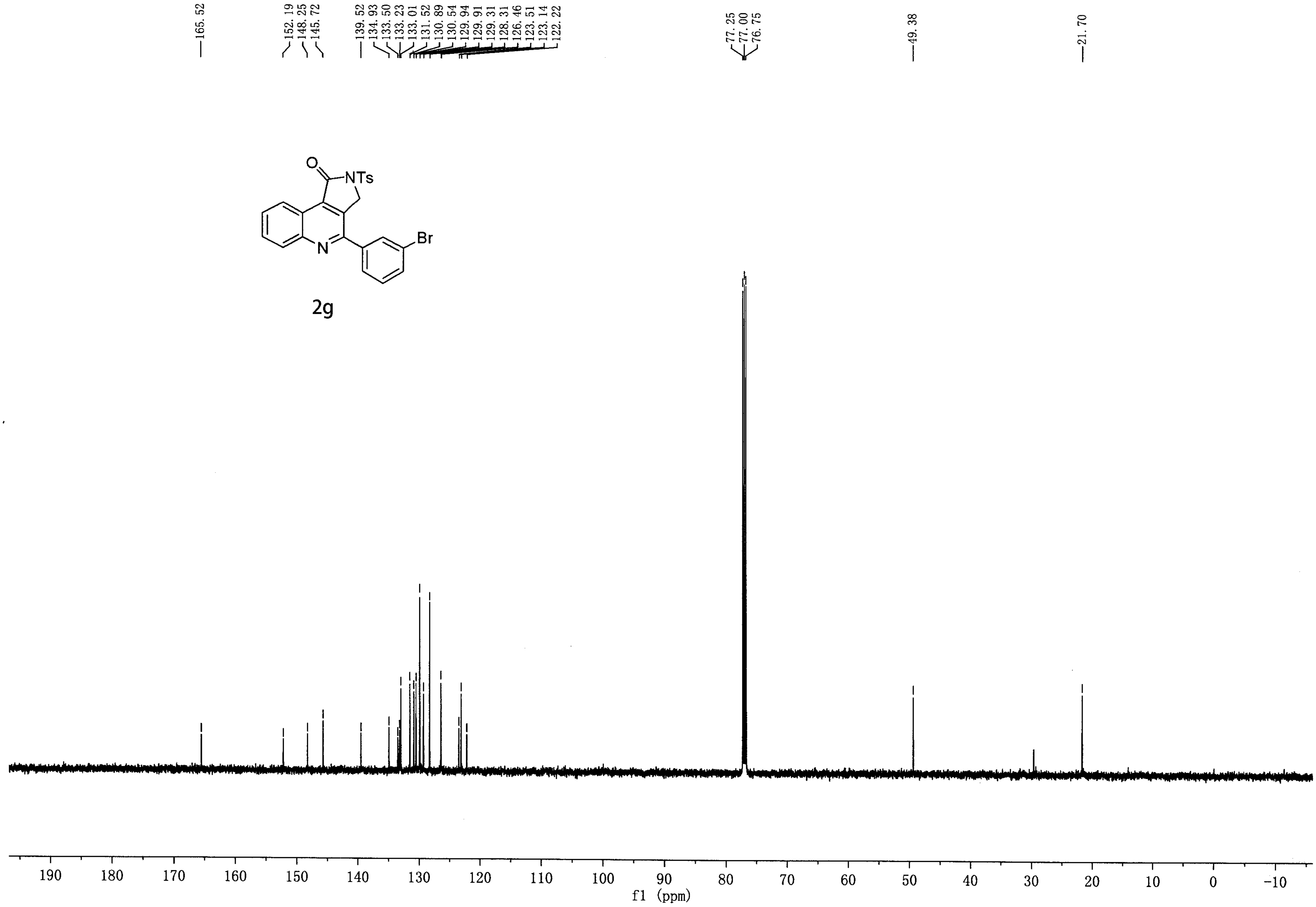

Supplementary Figure 28. ^{1}H and ^{13}C NMR spectra for 2g

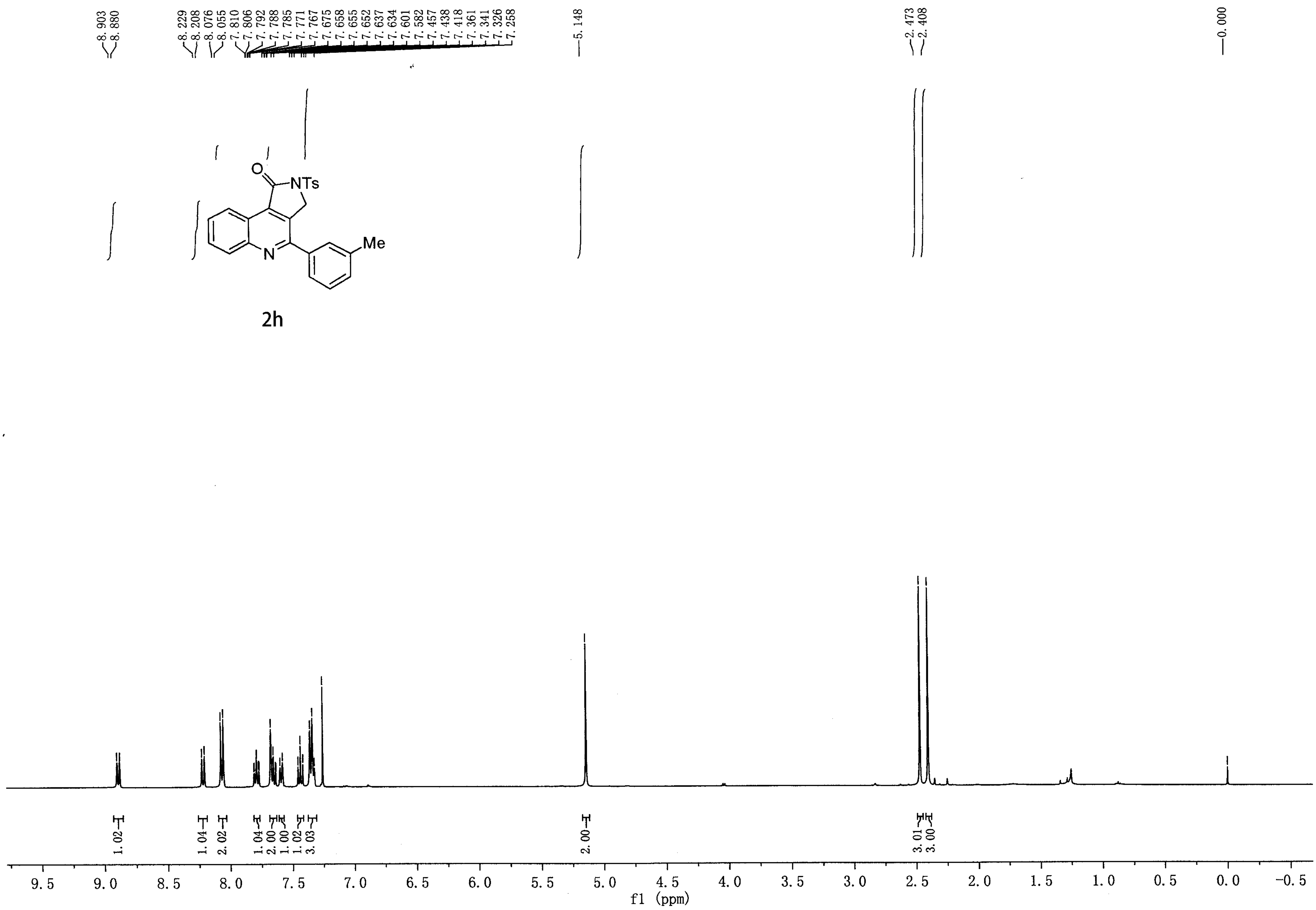

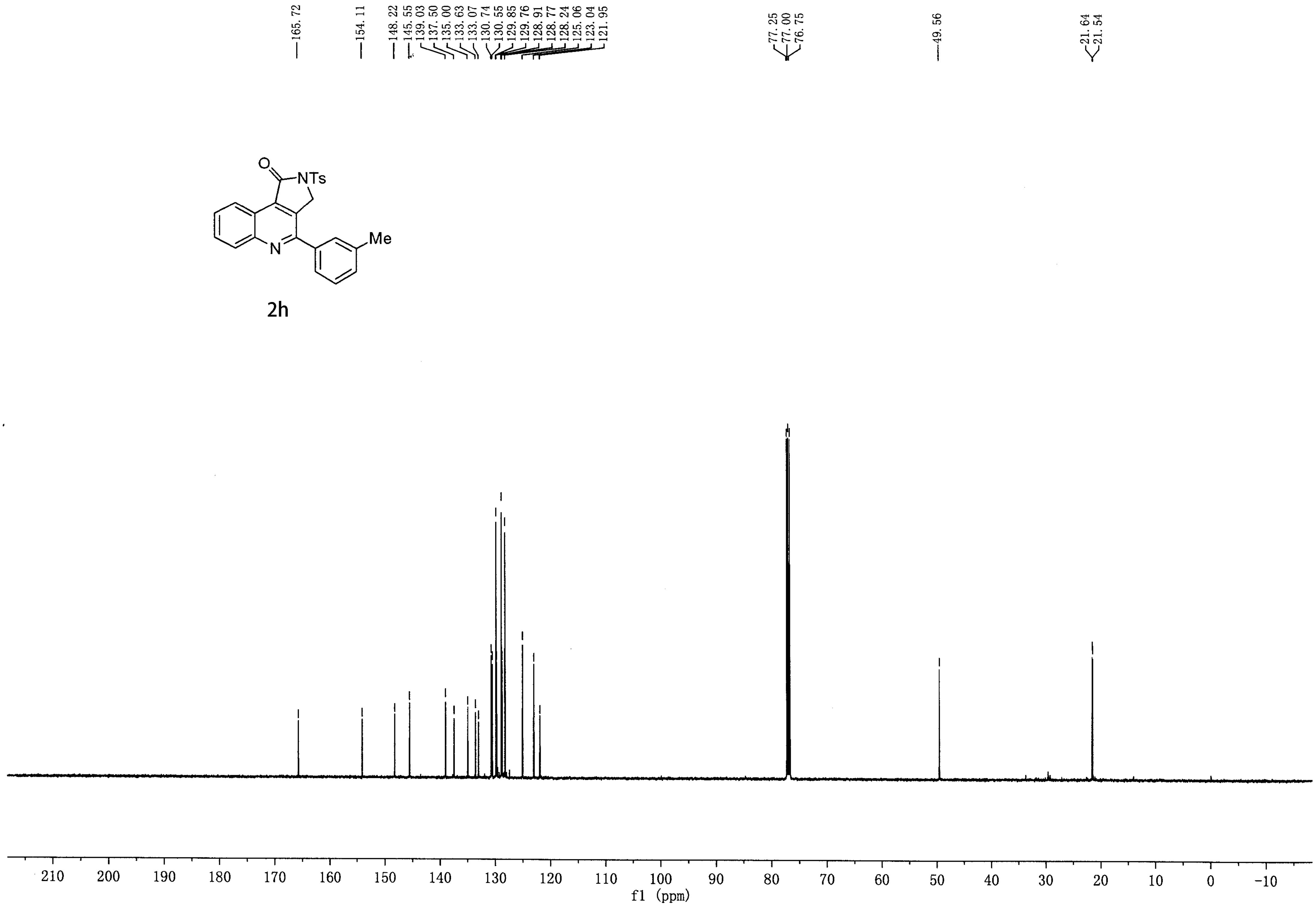

Supplementary Figure 29. ^{1}H and ^{13}C NMR spectra for 2h

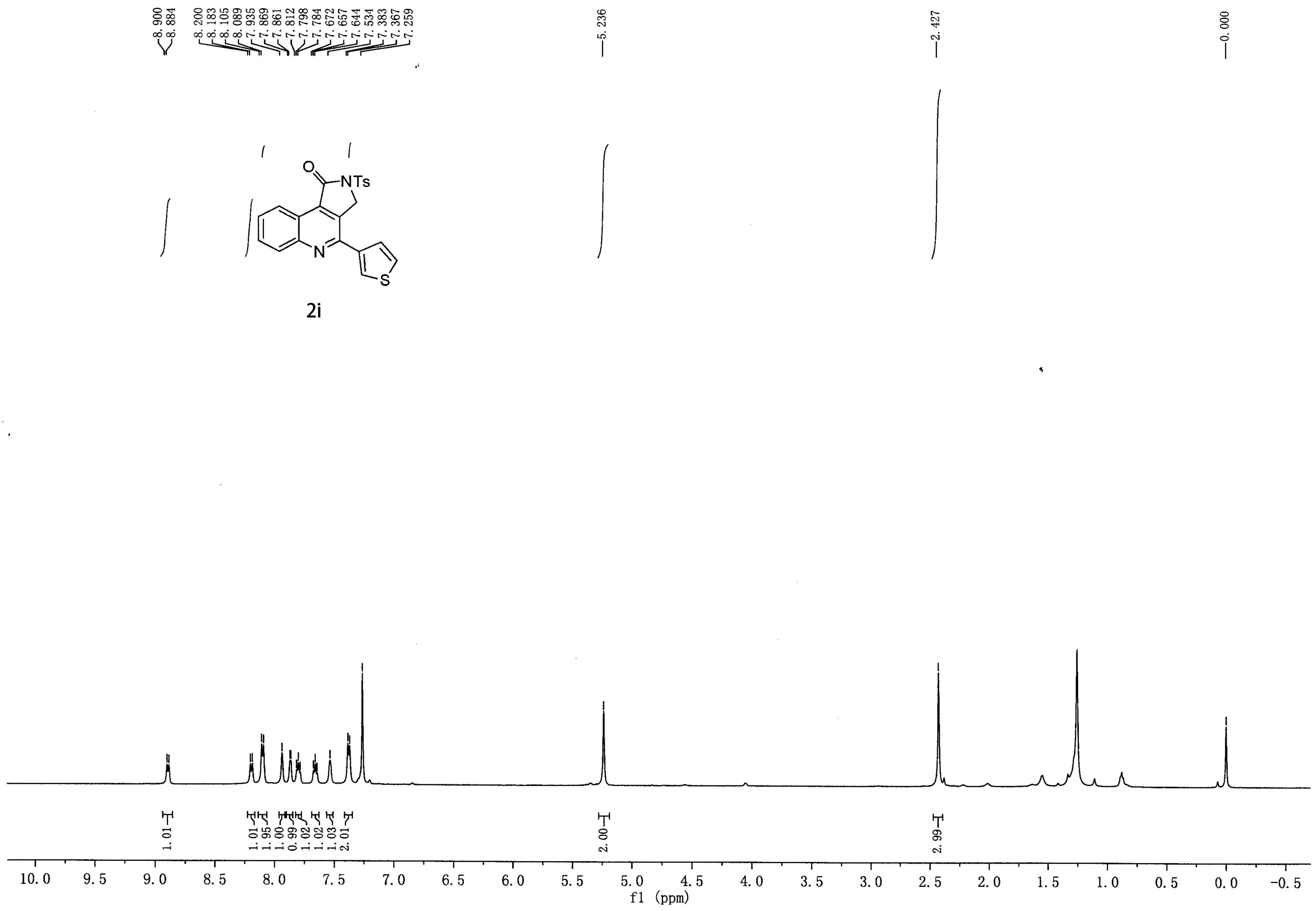

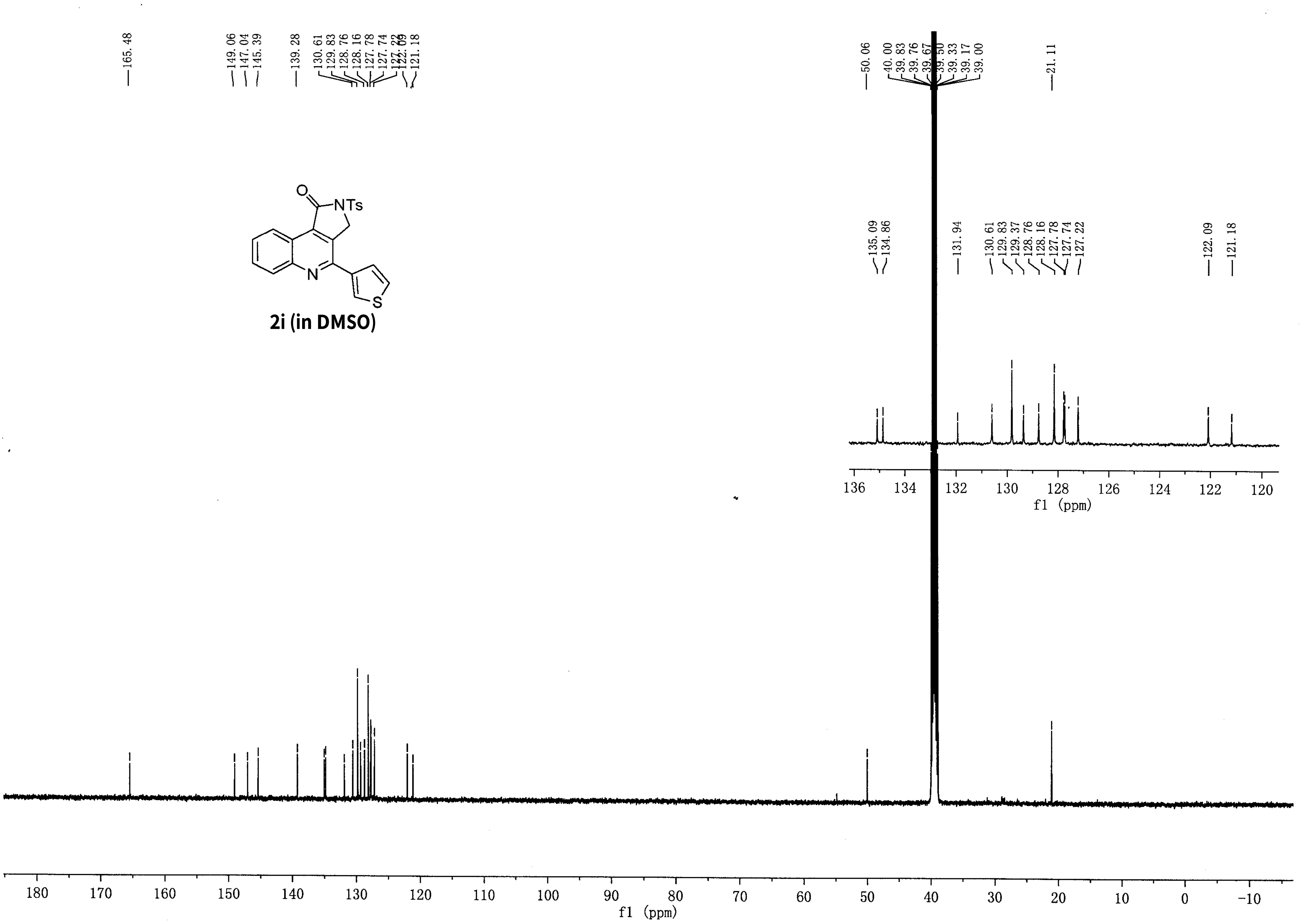

Supplementary Figure 30. ^{1}H and ^{13}C NMR spectra for 2i

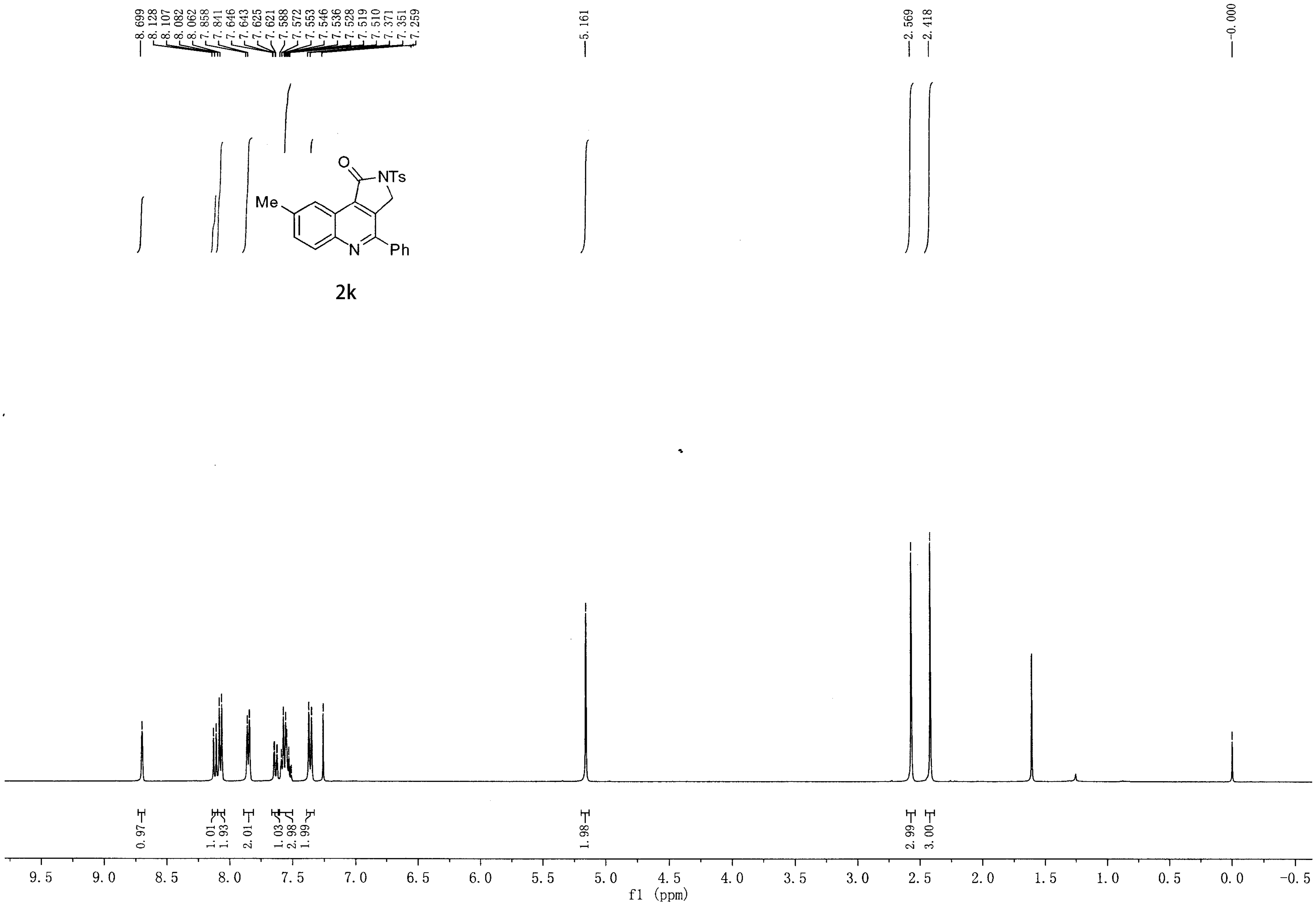

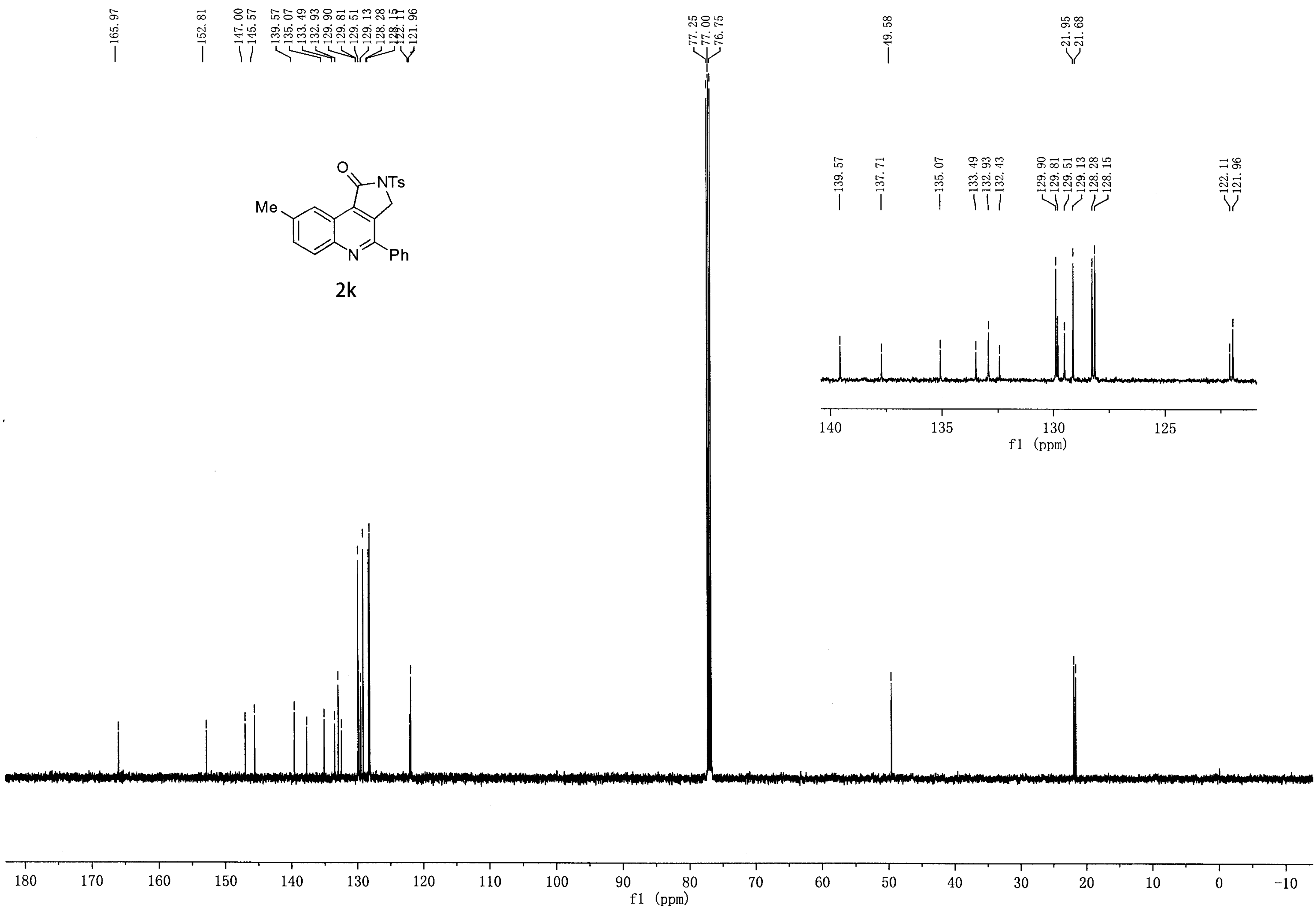

Supplementary Figure 31. ^{1}H and ^{13}C NMR spectra for 2k

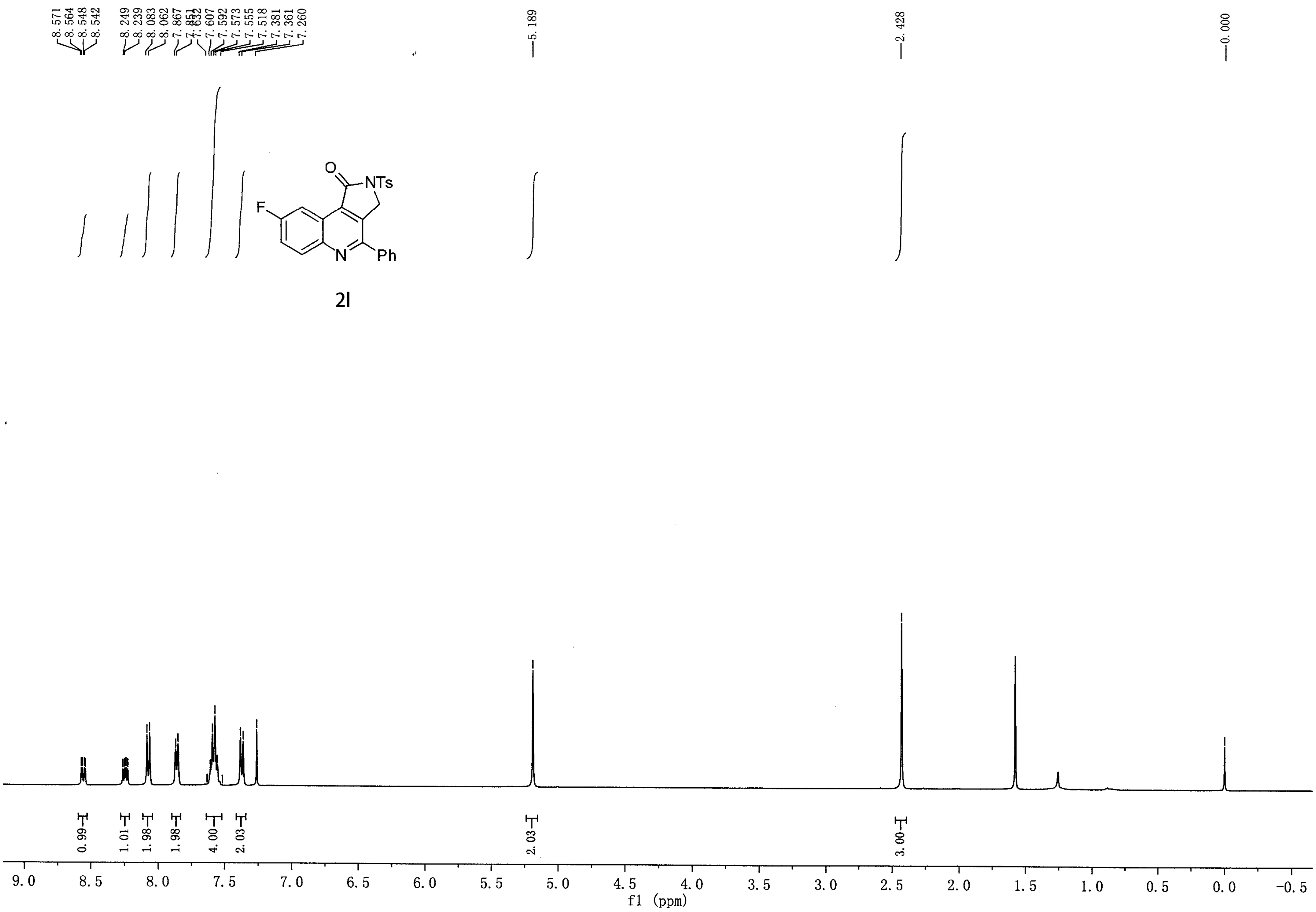

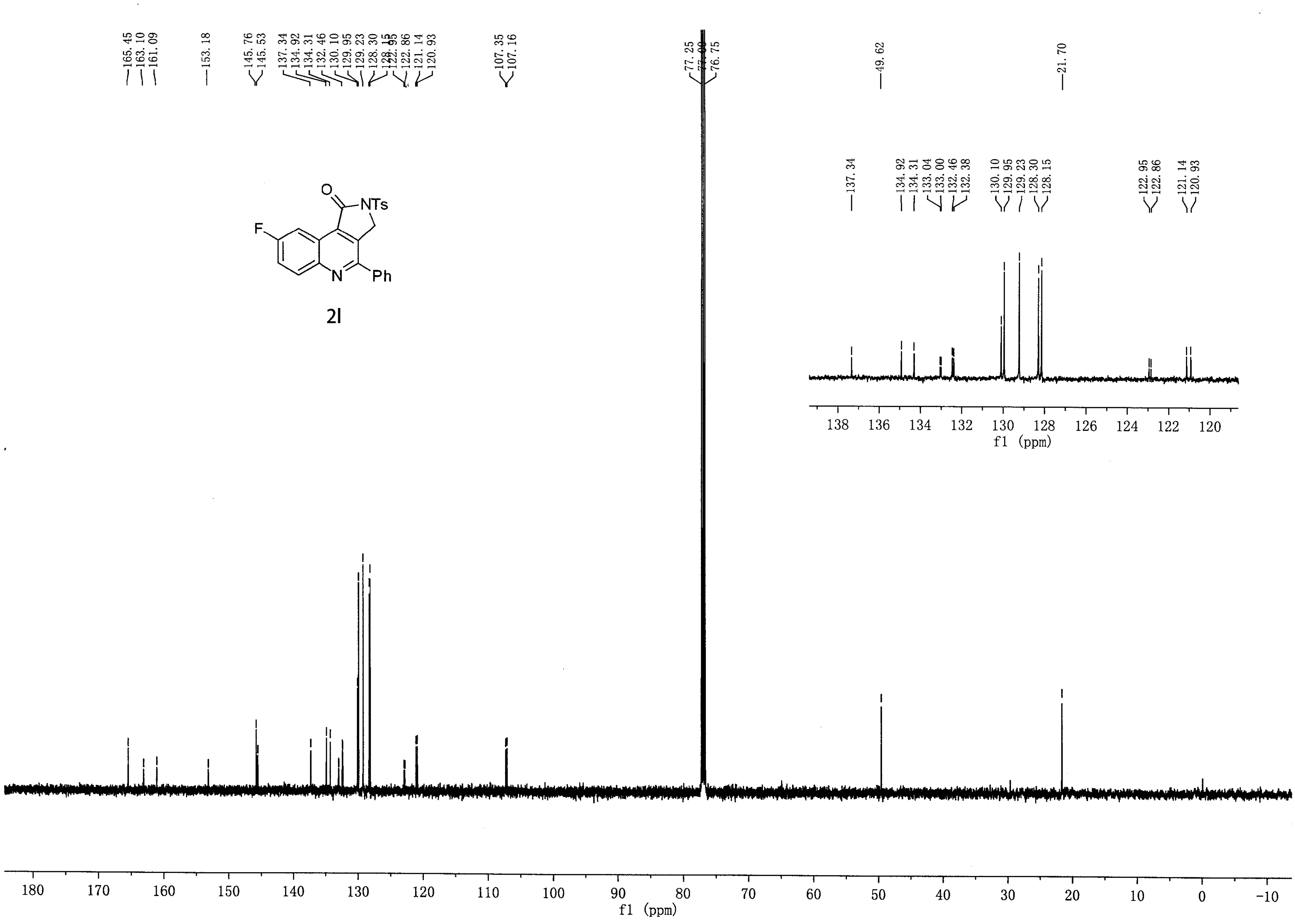

Supplementary Figure 32. ^{1}H and ^{13}C NMR spectra for 2l

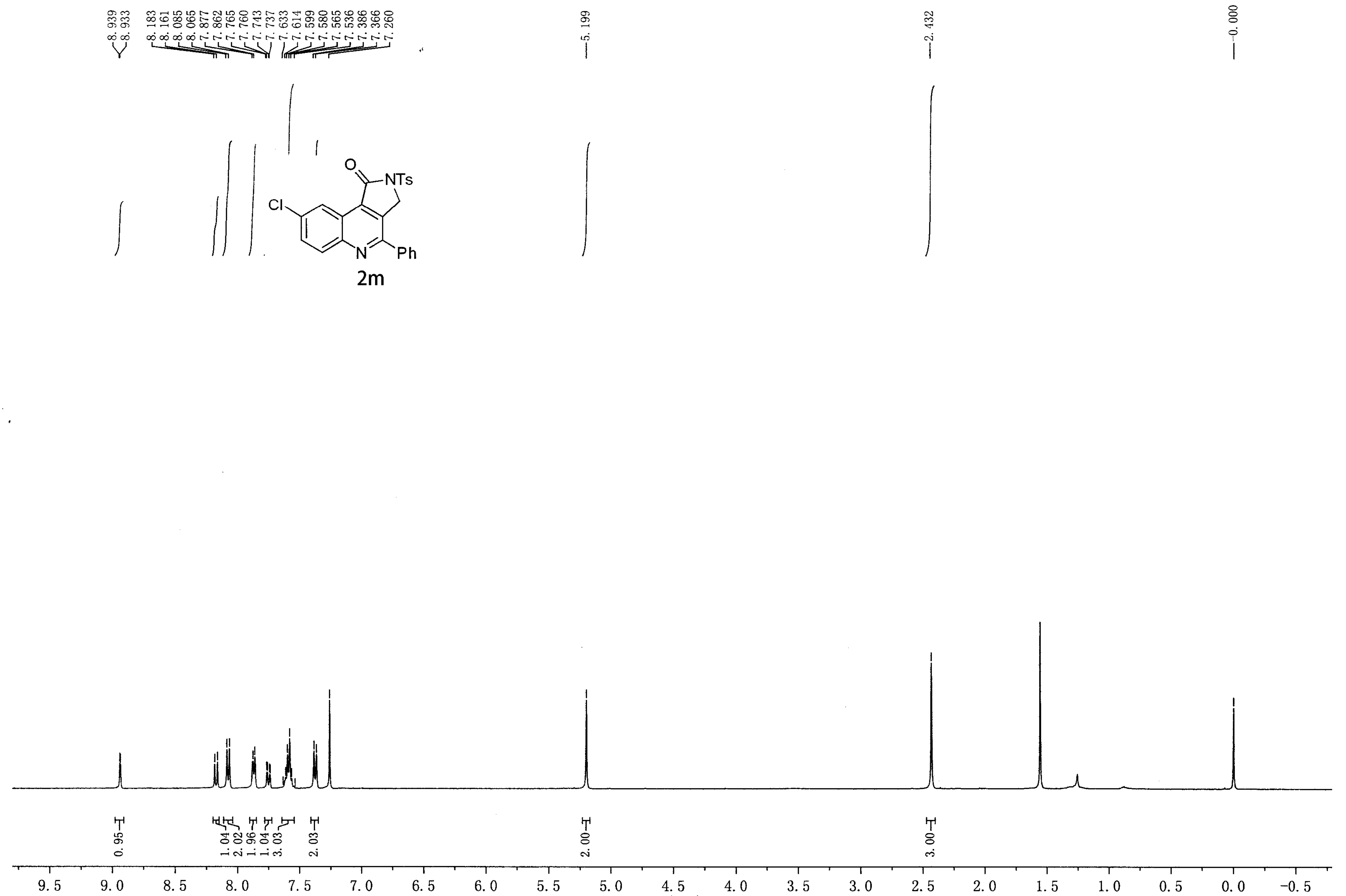

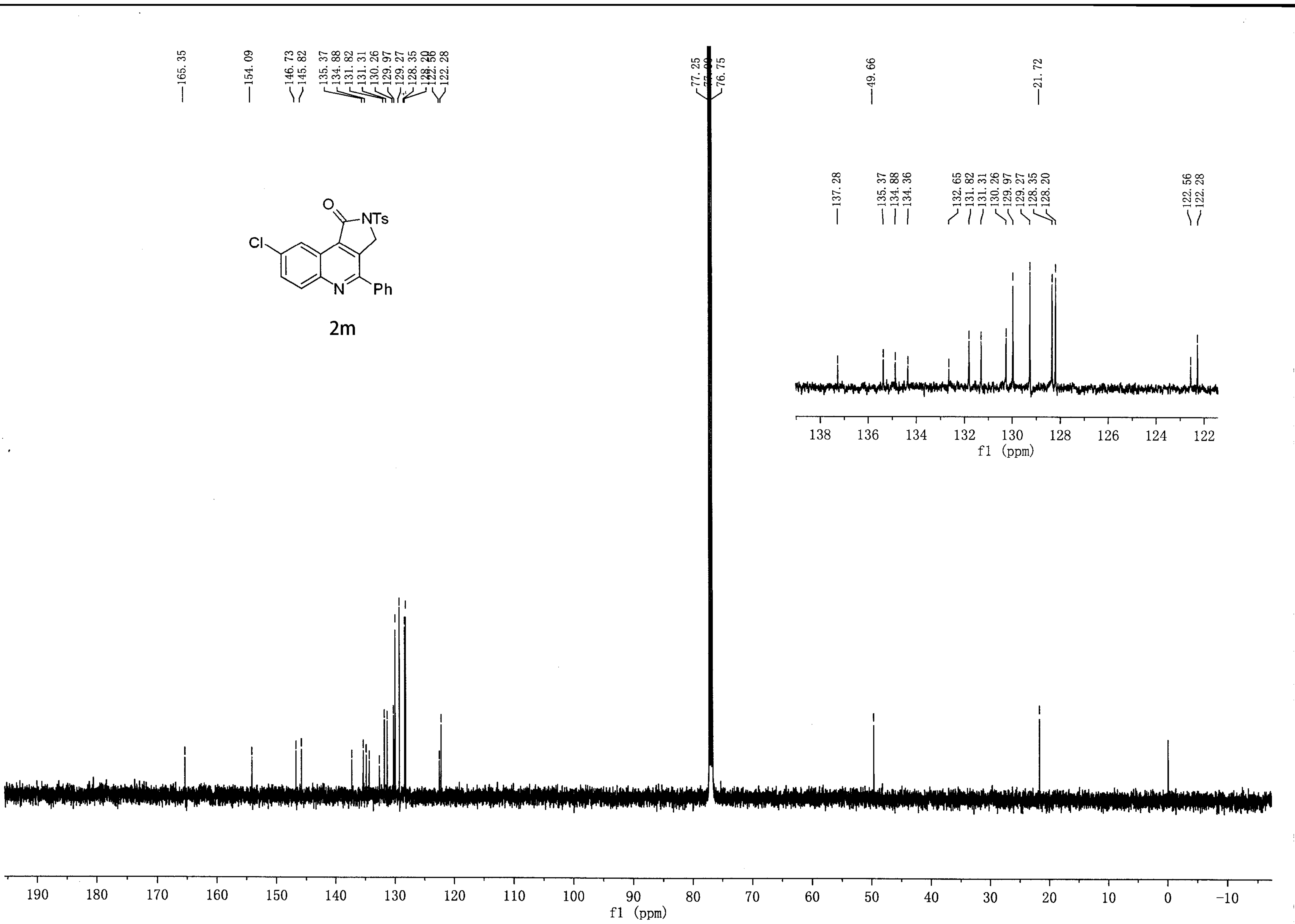

Supplementary Figure 33. ^{1}H and ^{13}C NMR spectra for 2m

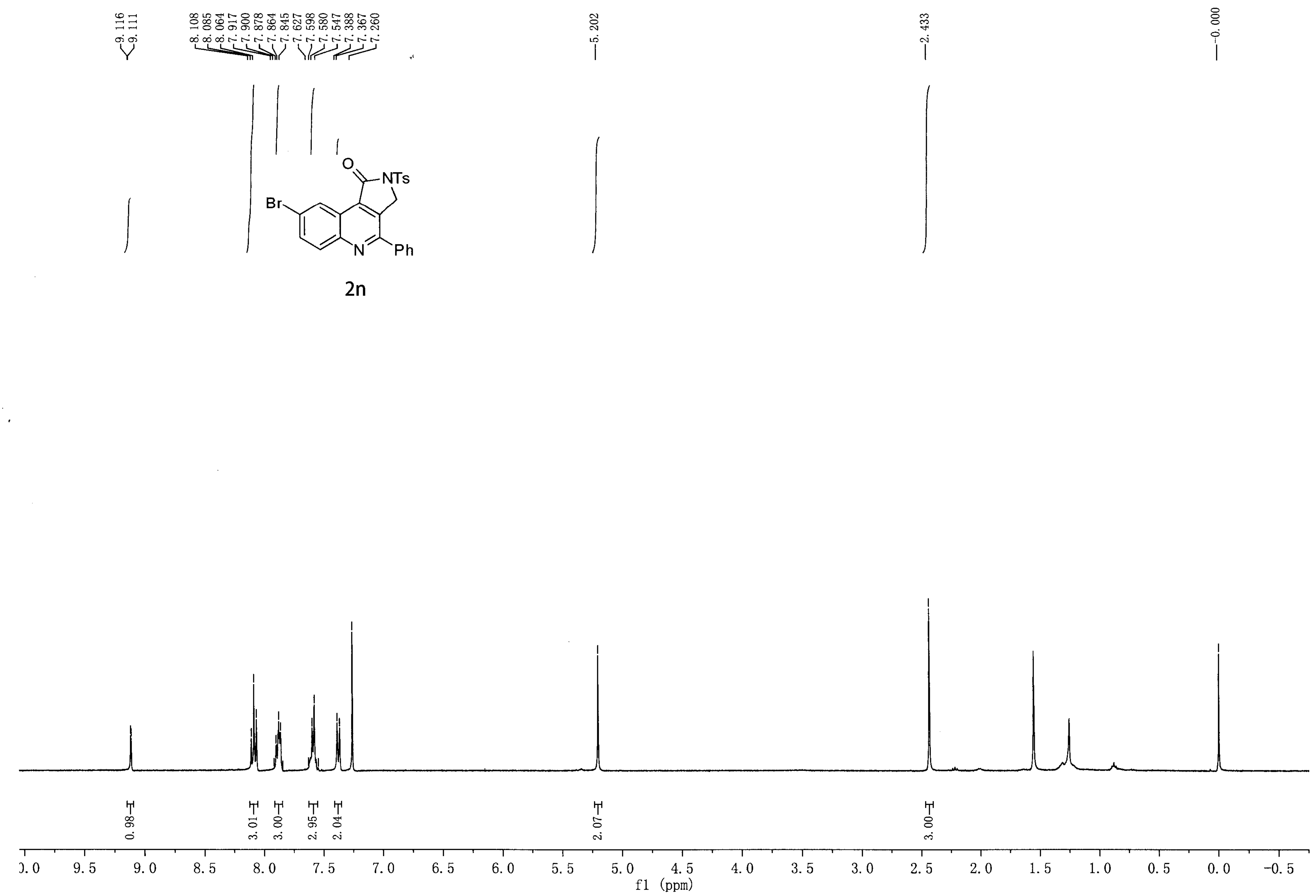

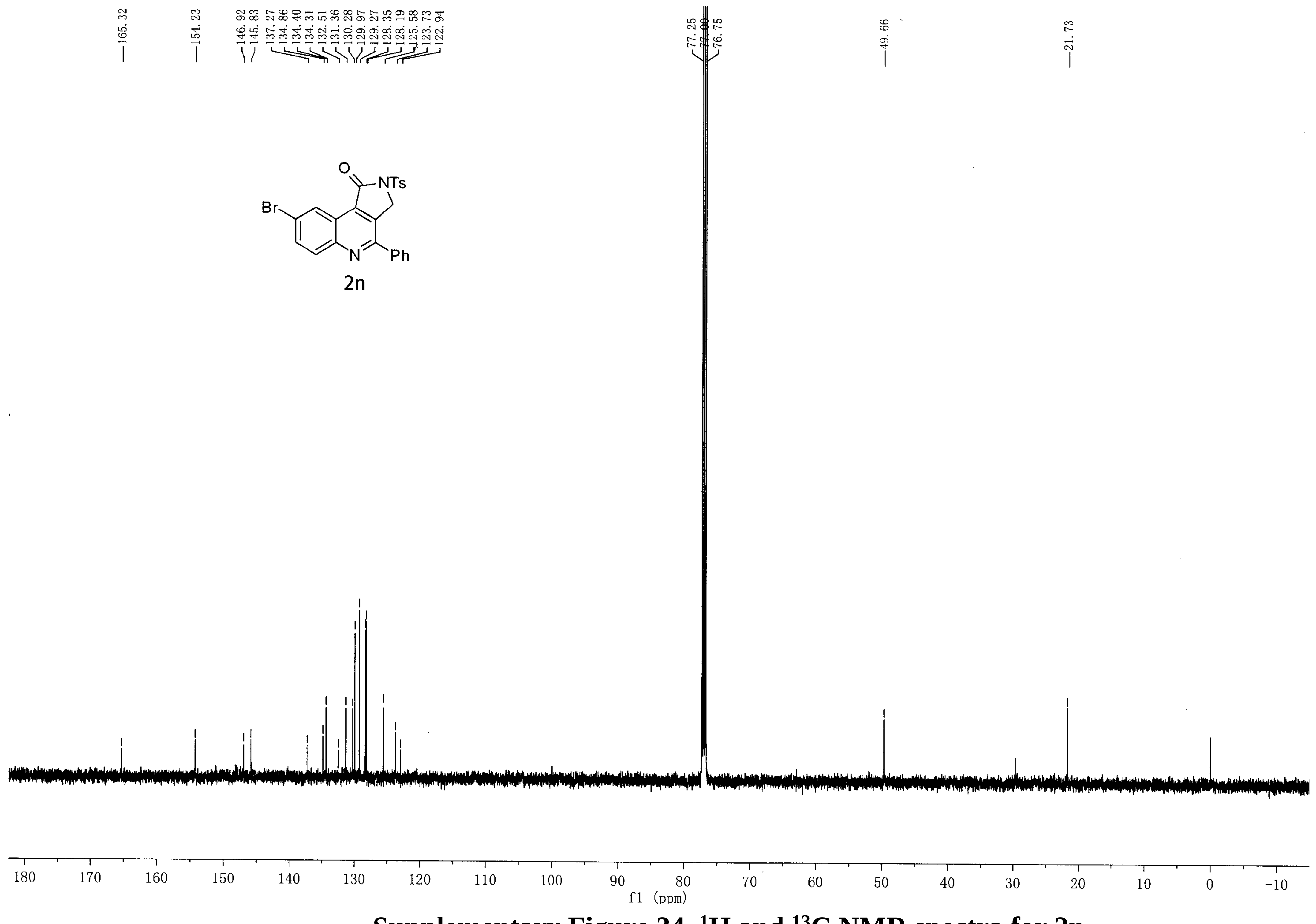

Supplementary Figure 34. ^{1}H and ^{13}C NMR spectra for 2n

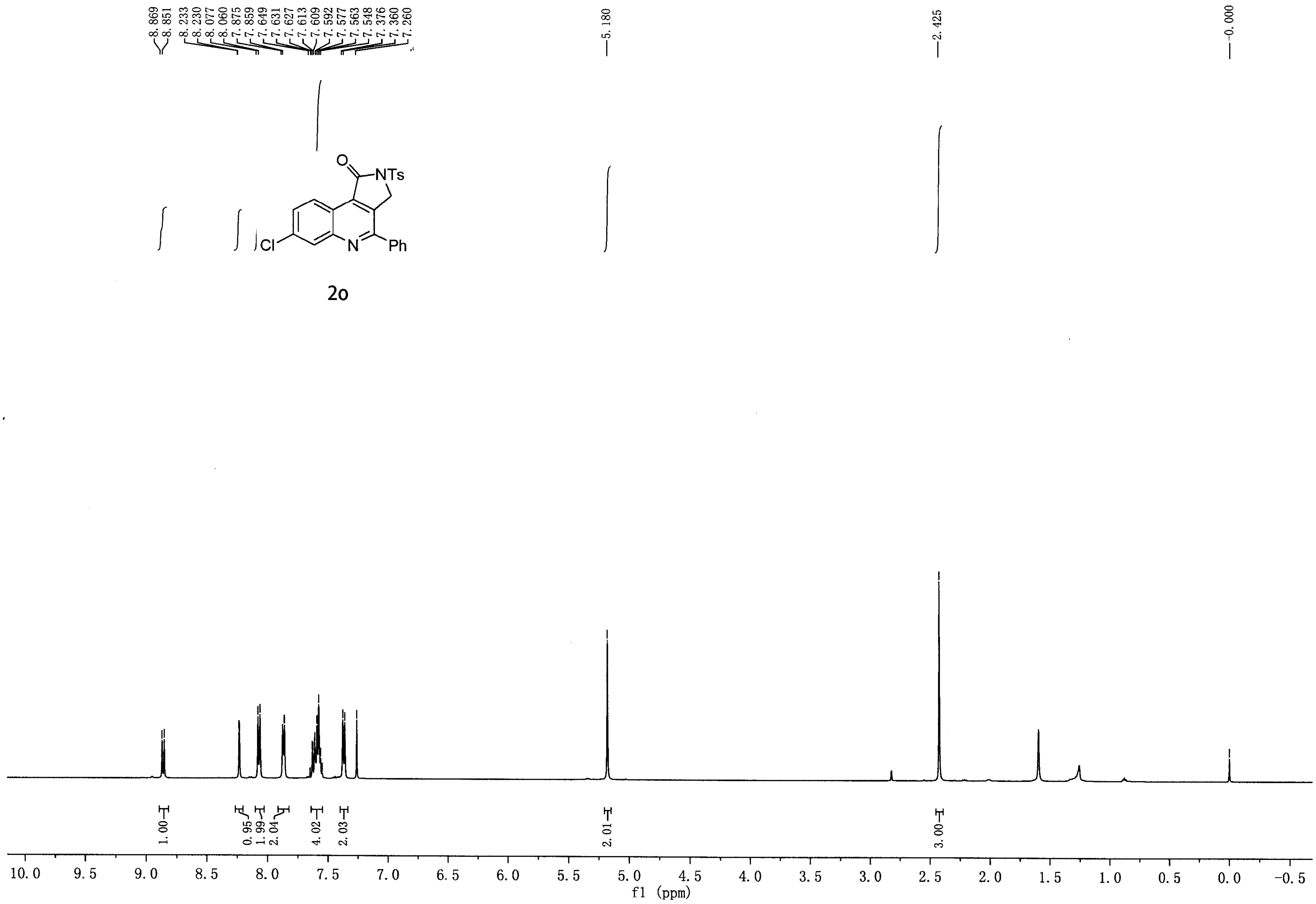

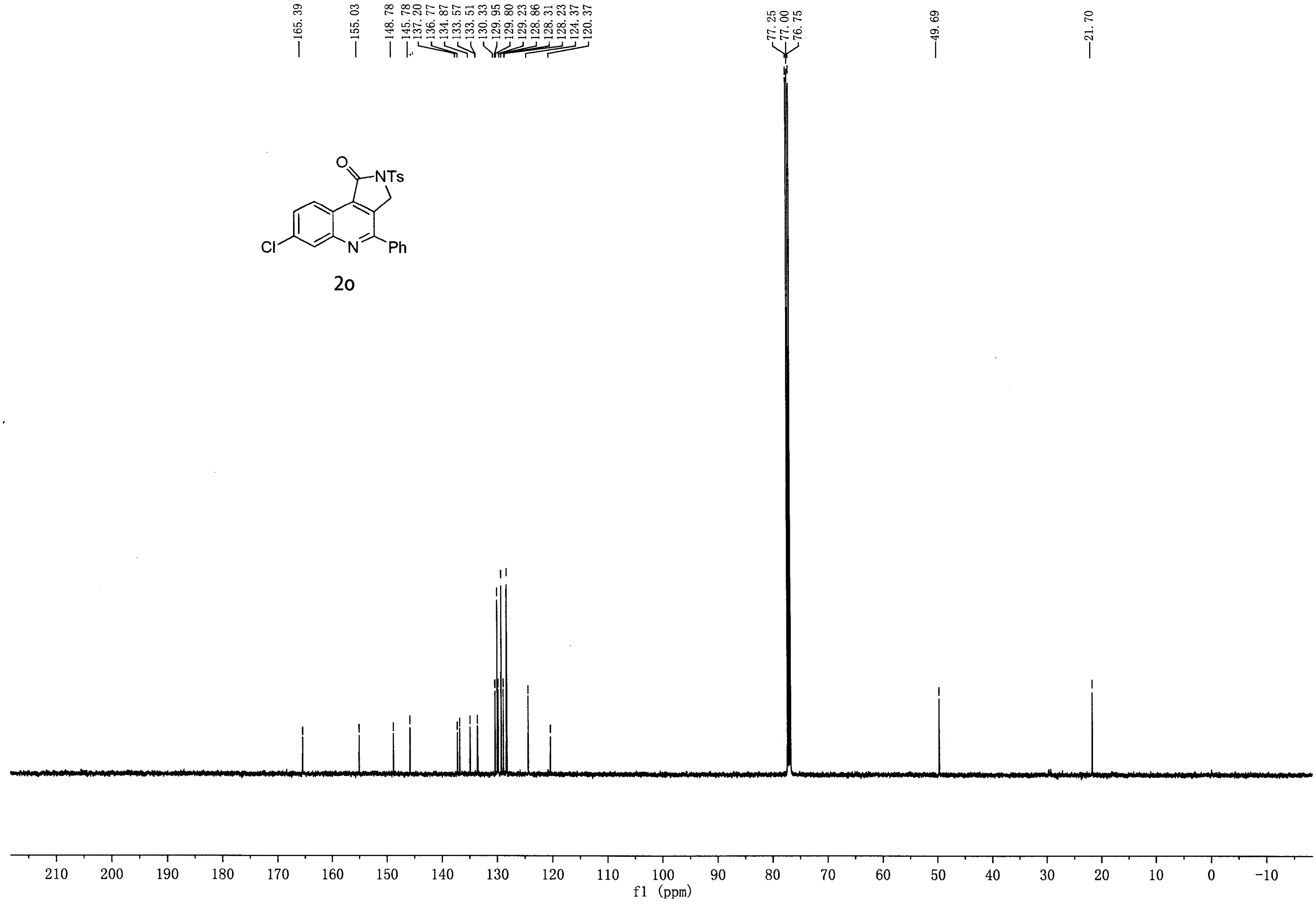

Supplementary Figure 35. ^{1}H and ^{13}C NMR spectra for 2o

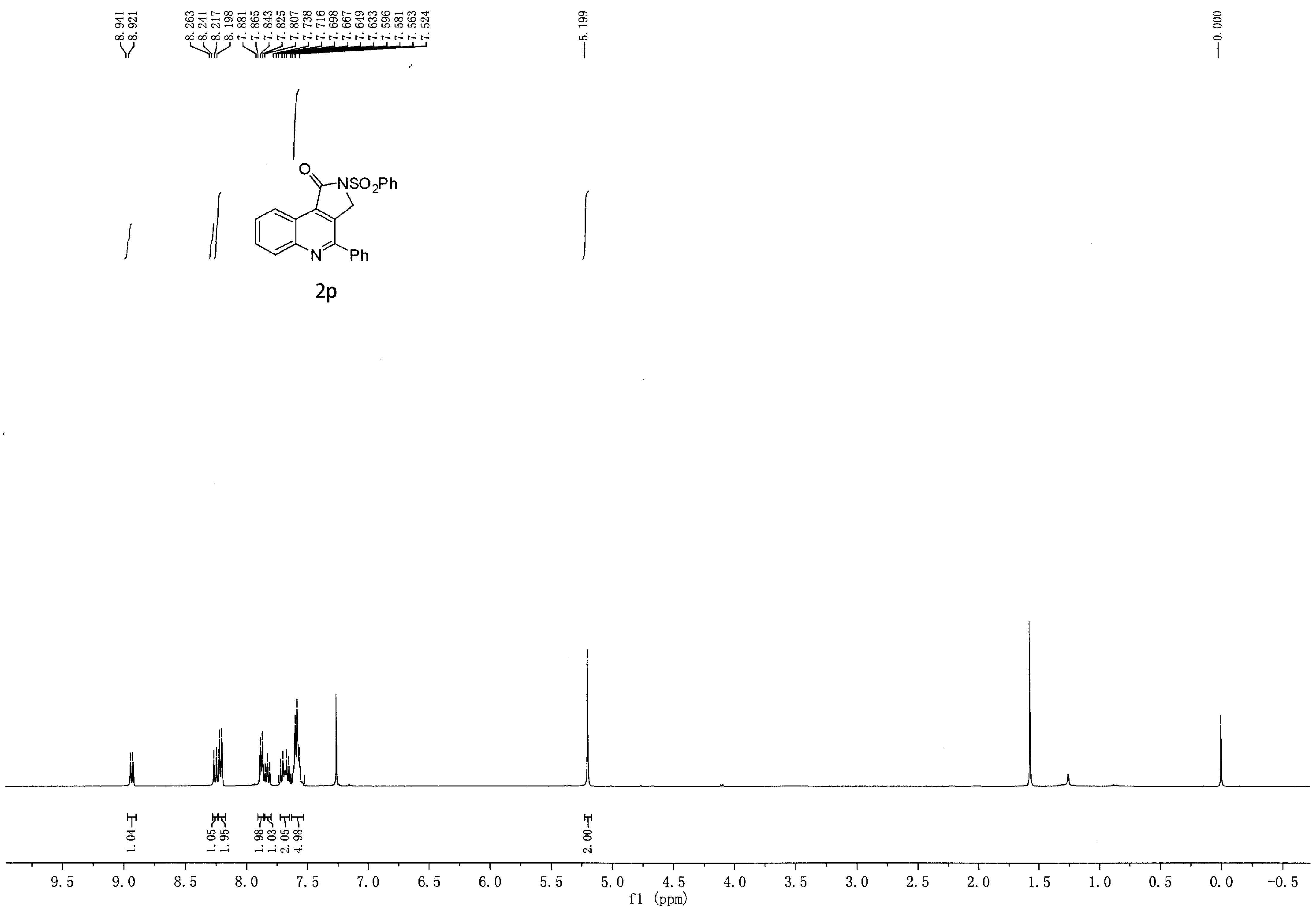

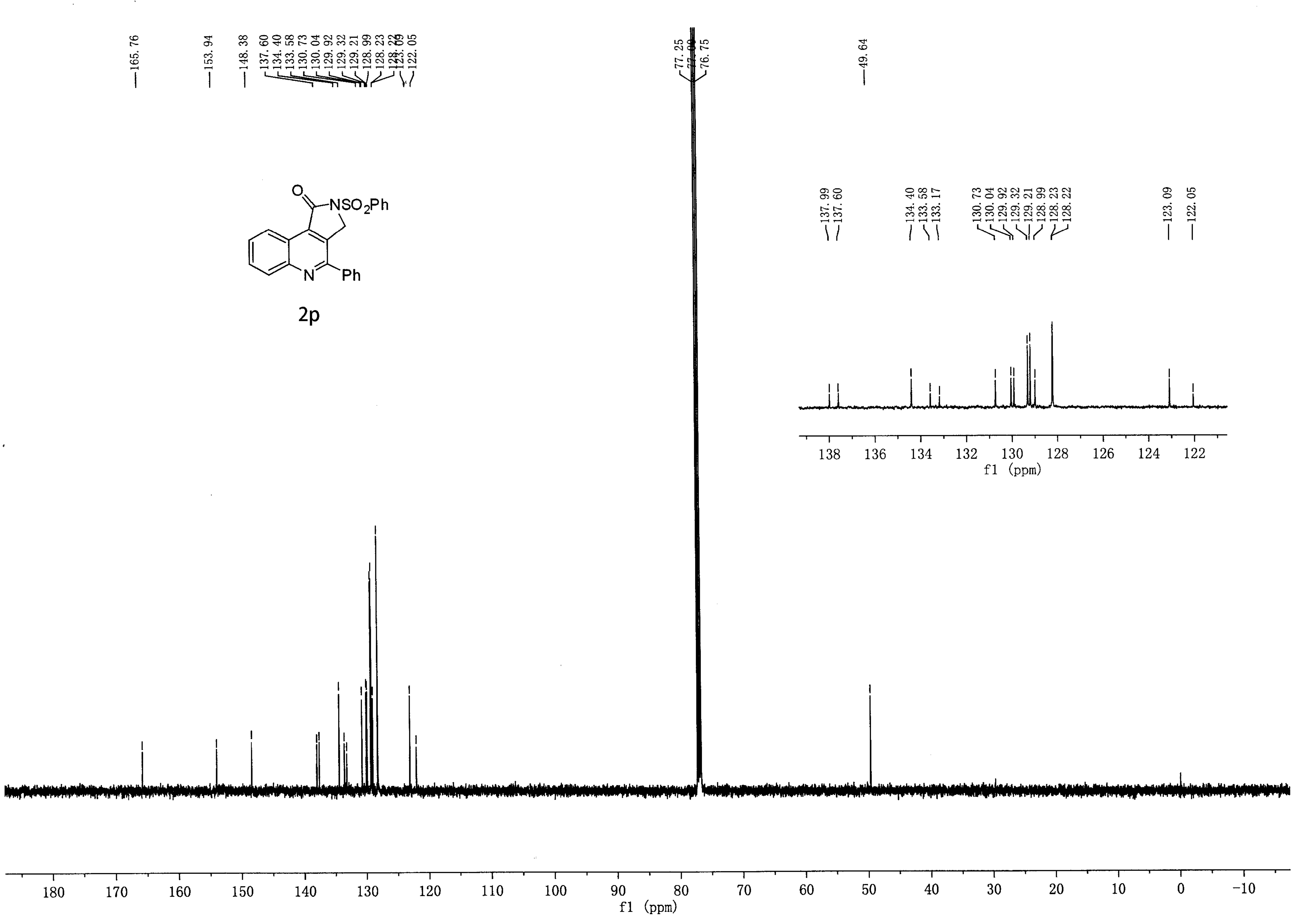

Supplementary Figure 36. ^{1}H and ^{13}C NMR spectra for 2p

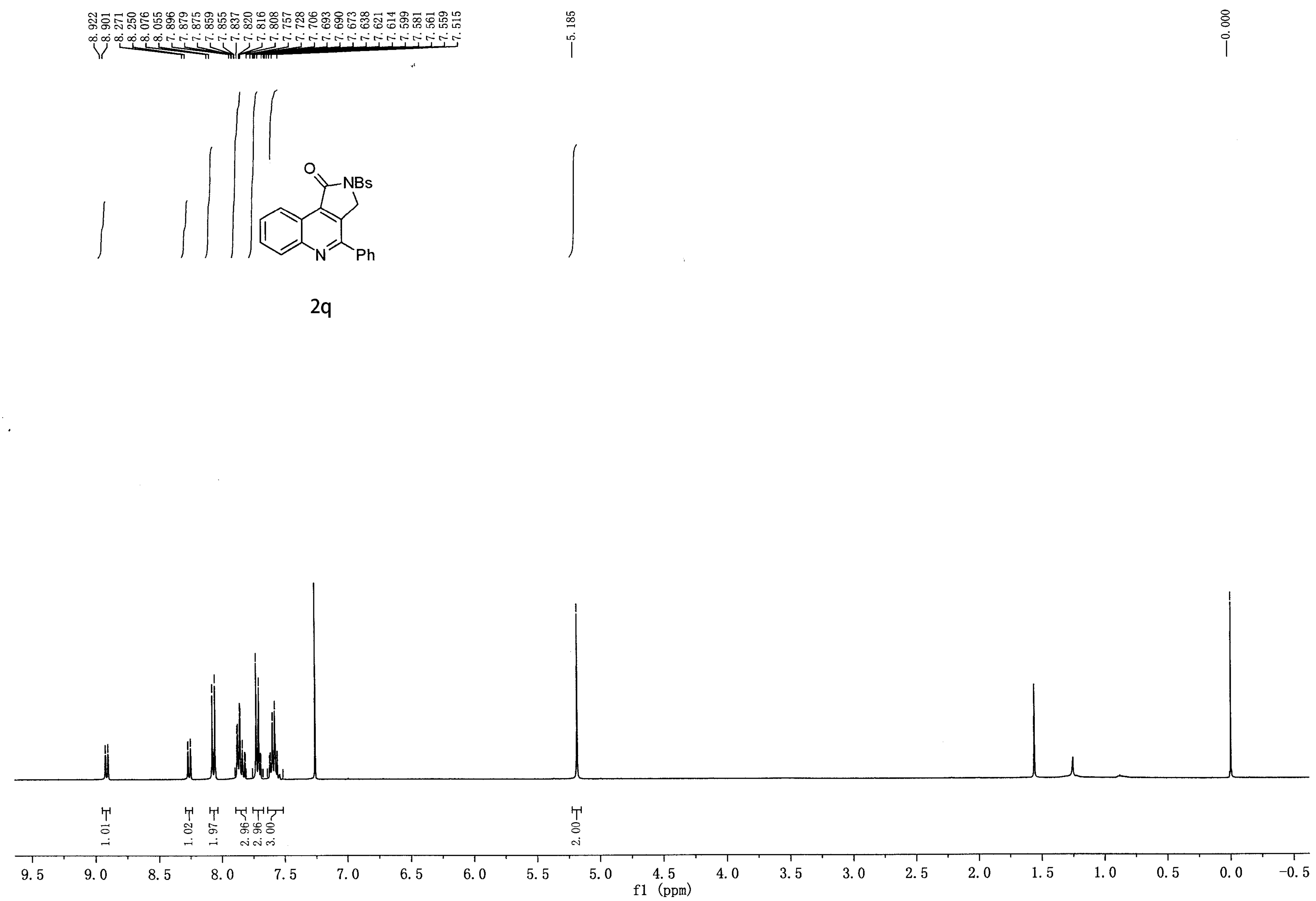

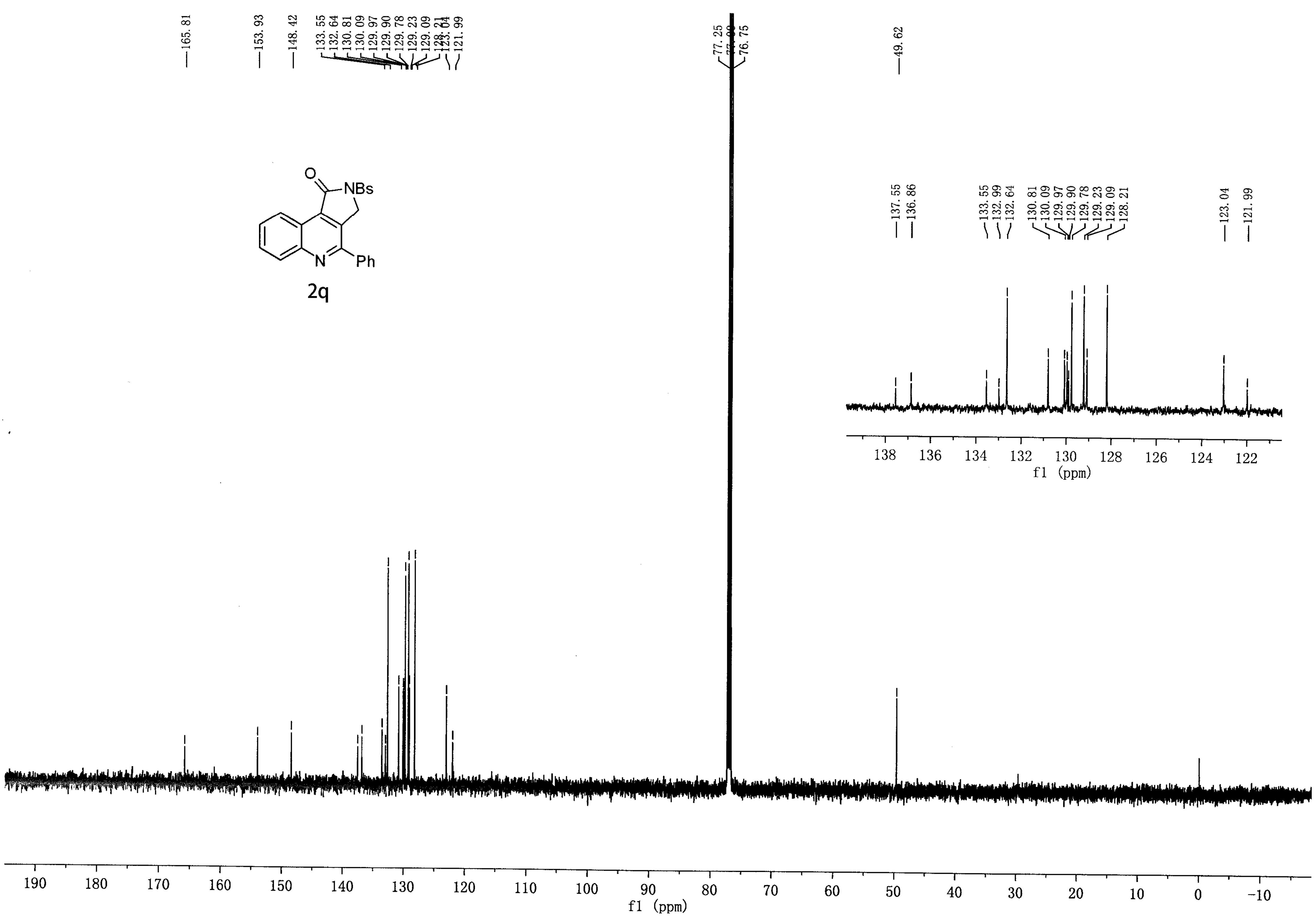

Supplementary Figure 37. ^{1}H and ^{13}C NMR spectra for 2q

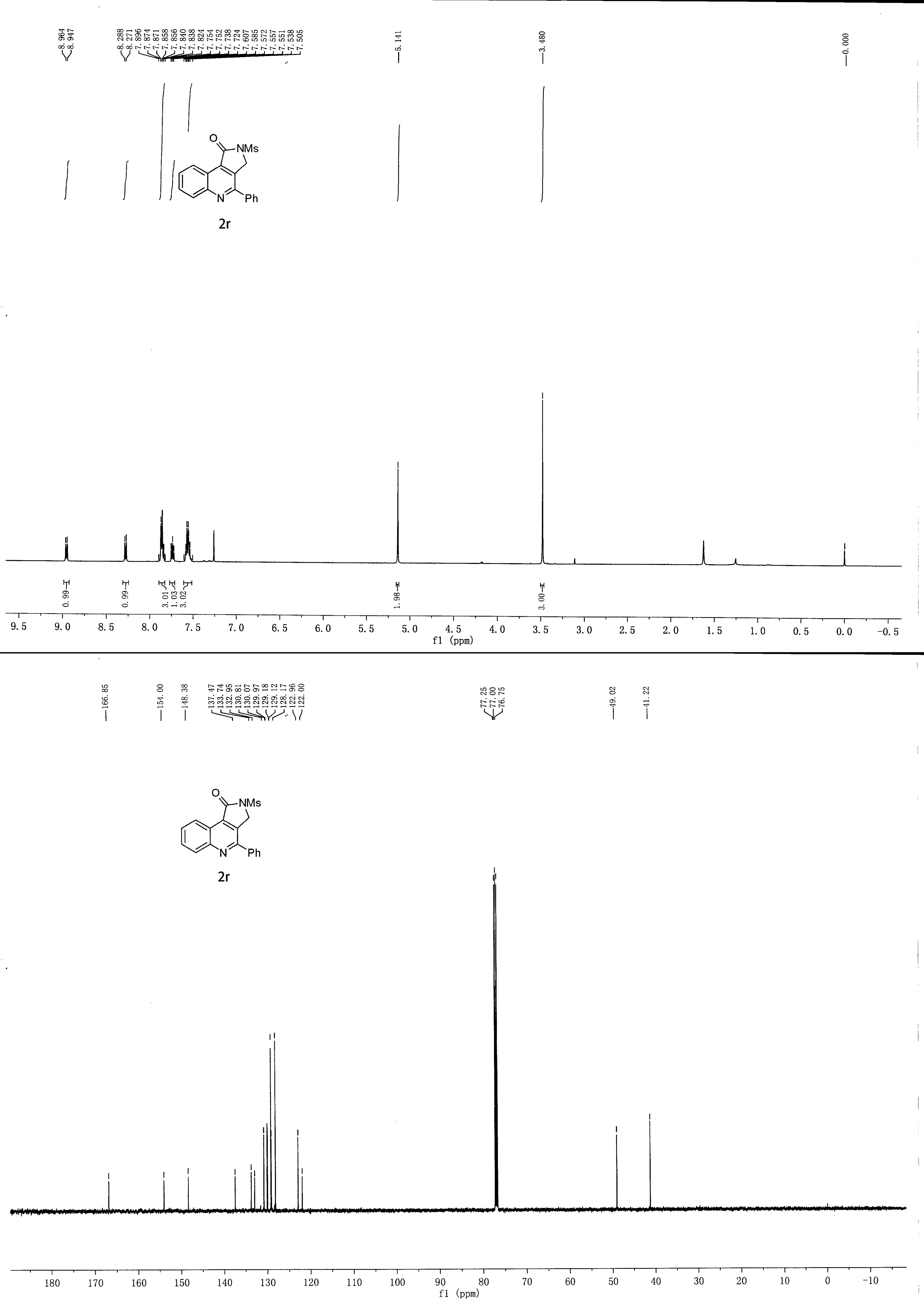

Supplementary Figure 38. ^{1}H and ^{13}C NMR spectra for 2r

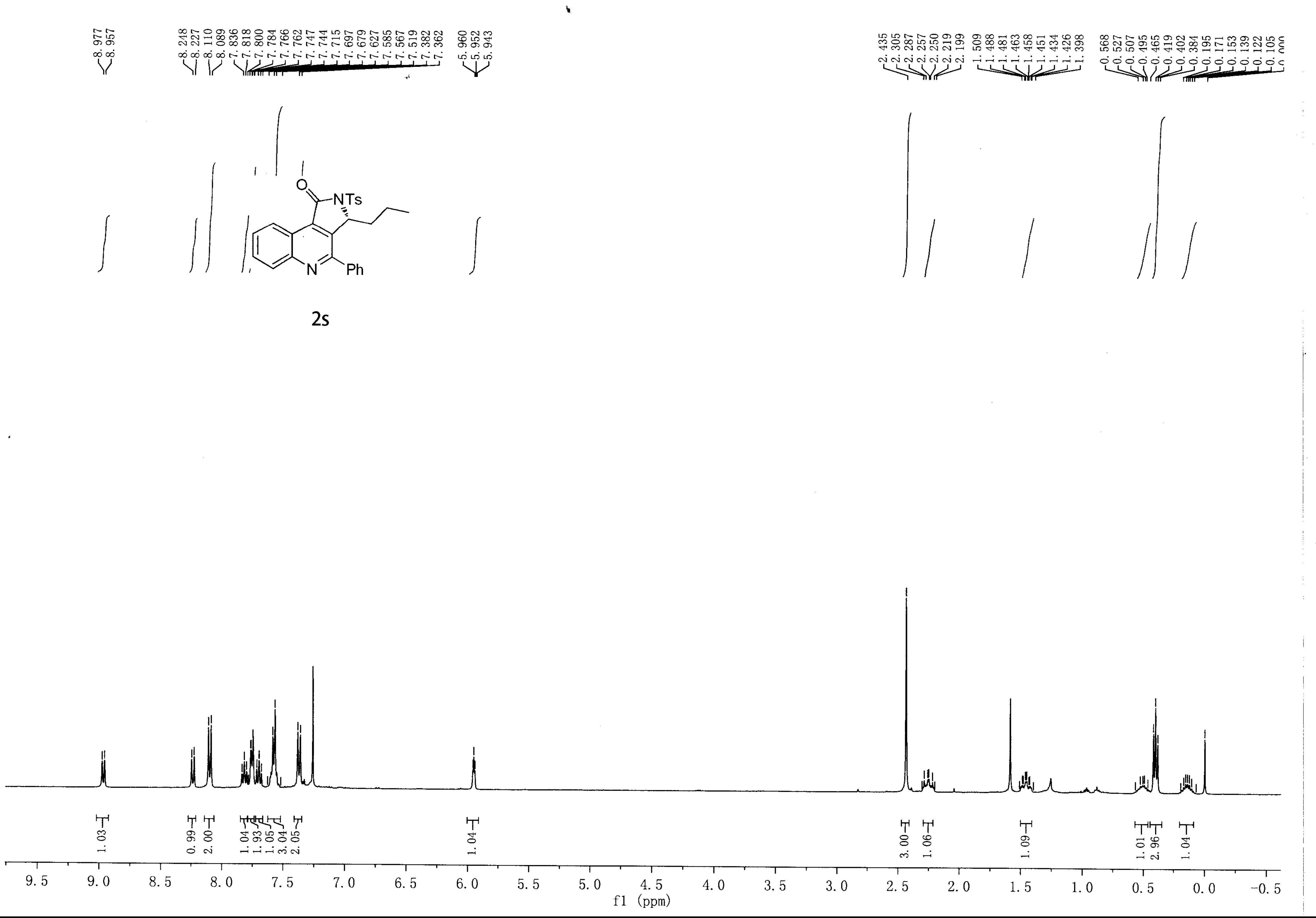

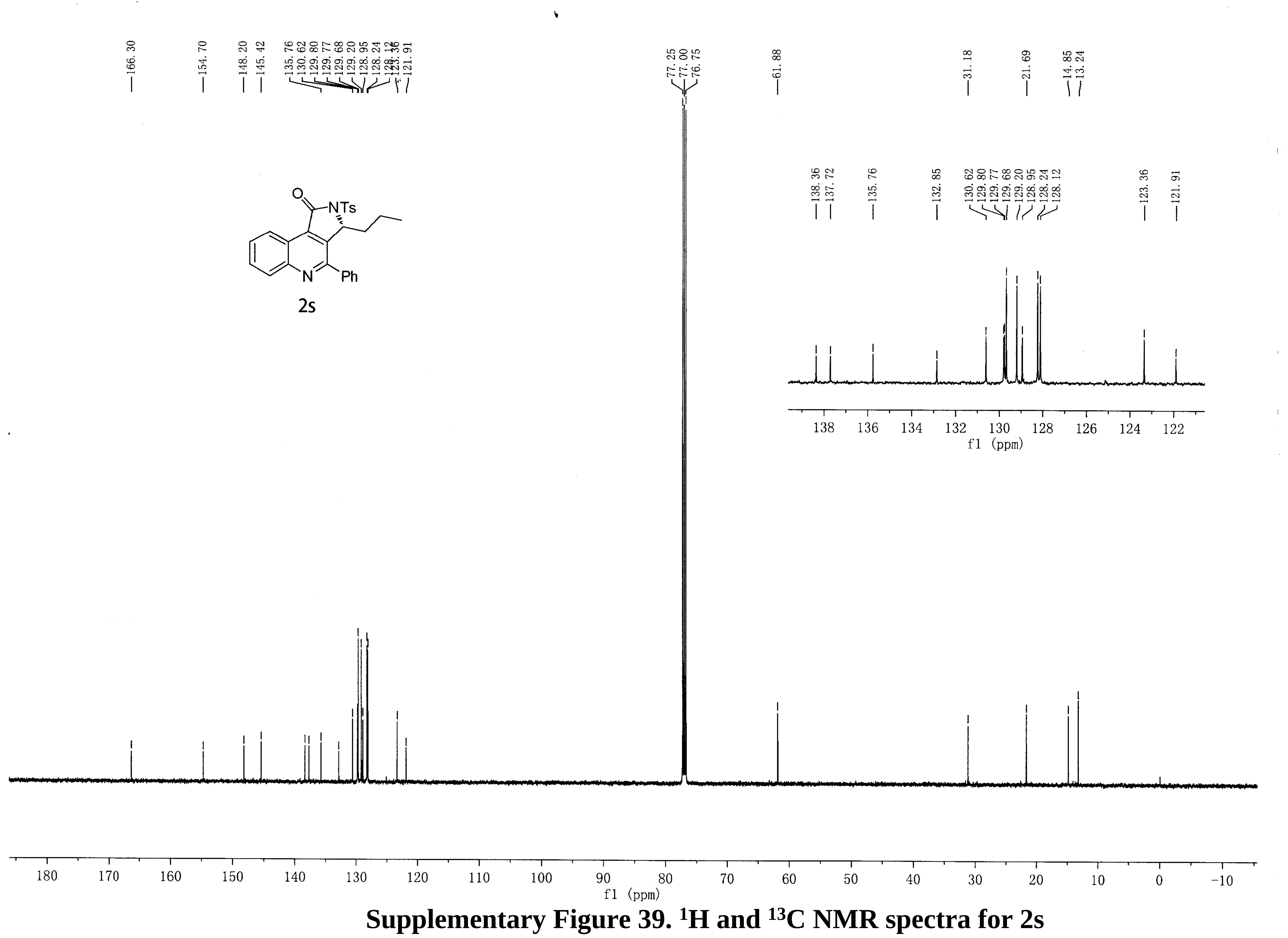

Supplementary Figure 39. ^{1}H and ^{13}C NMR spectra for 2s

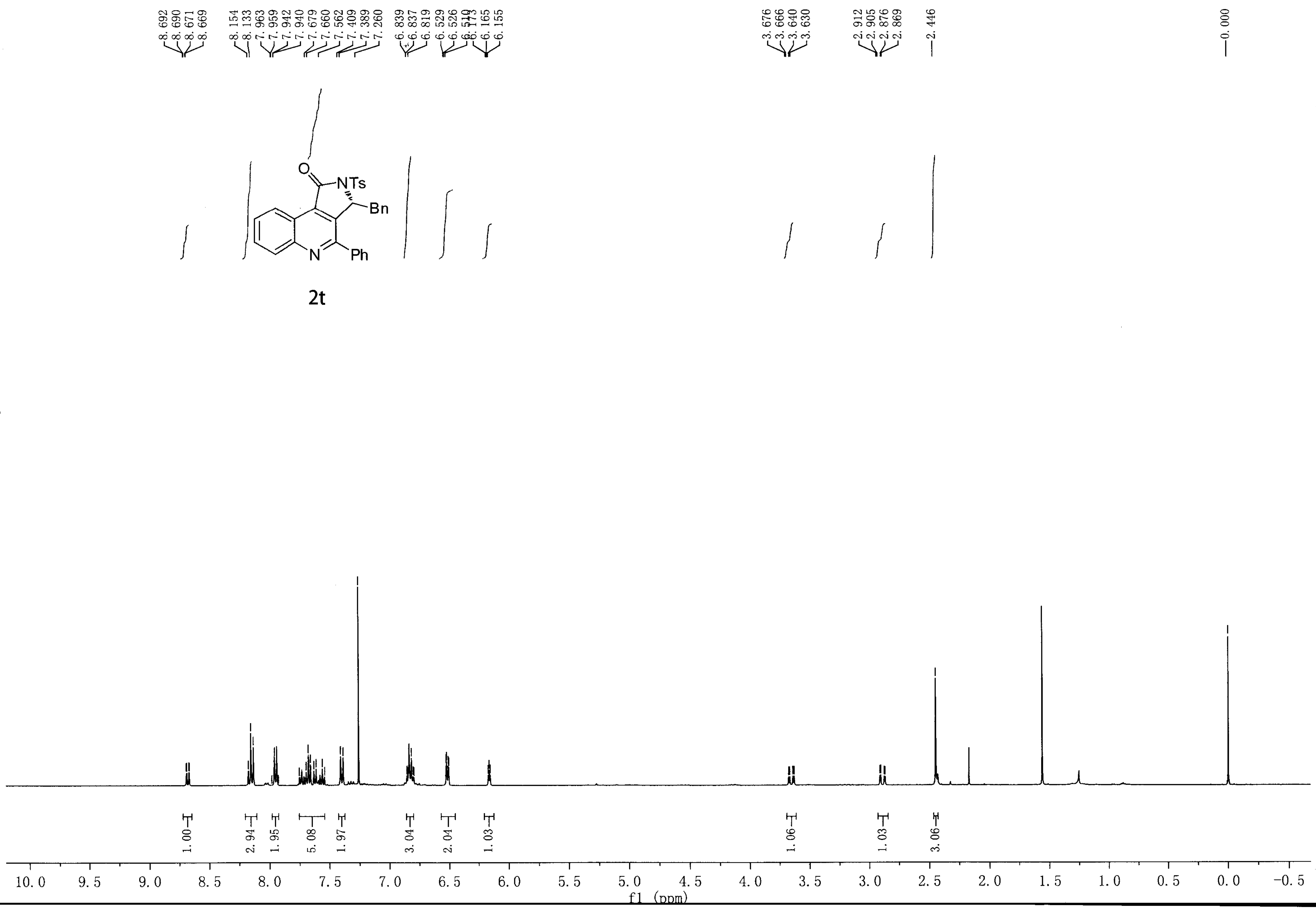

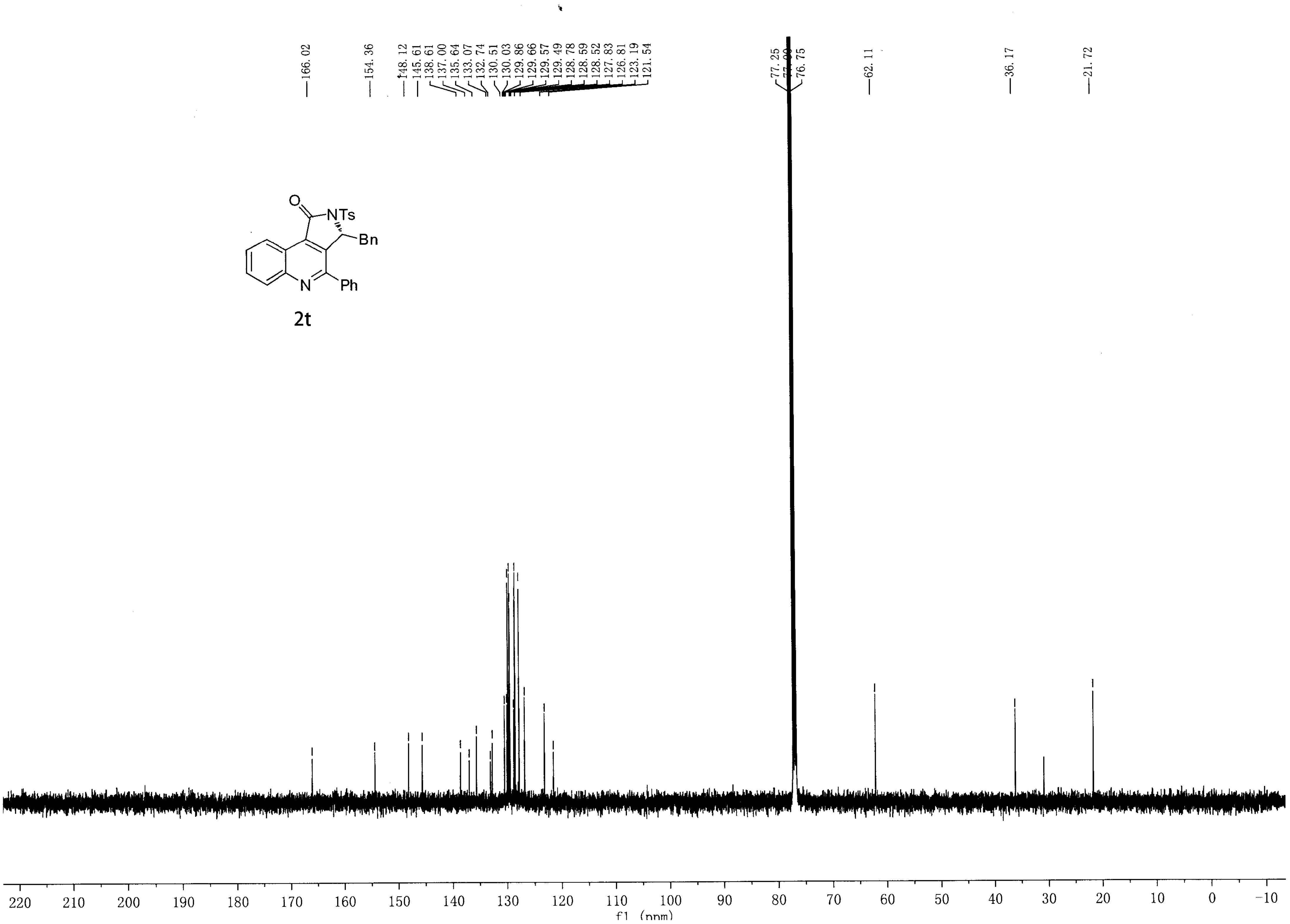

Supplementary Figure 40. ^{1}H and ^{13}C NMR spectra for 2t

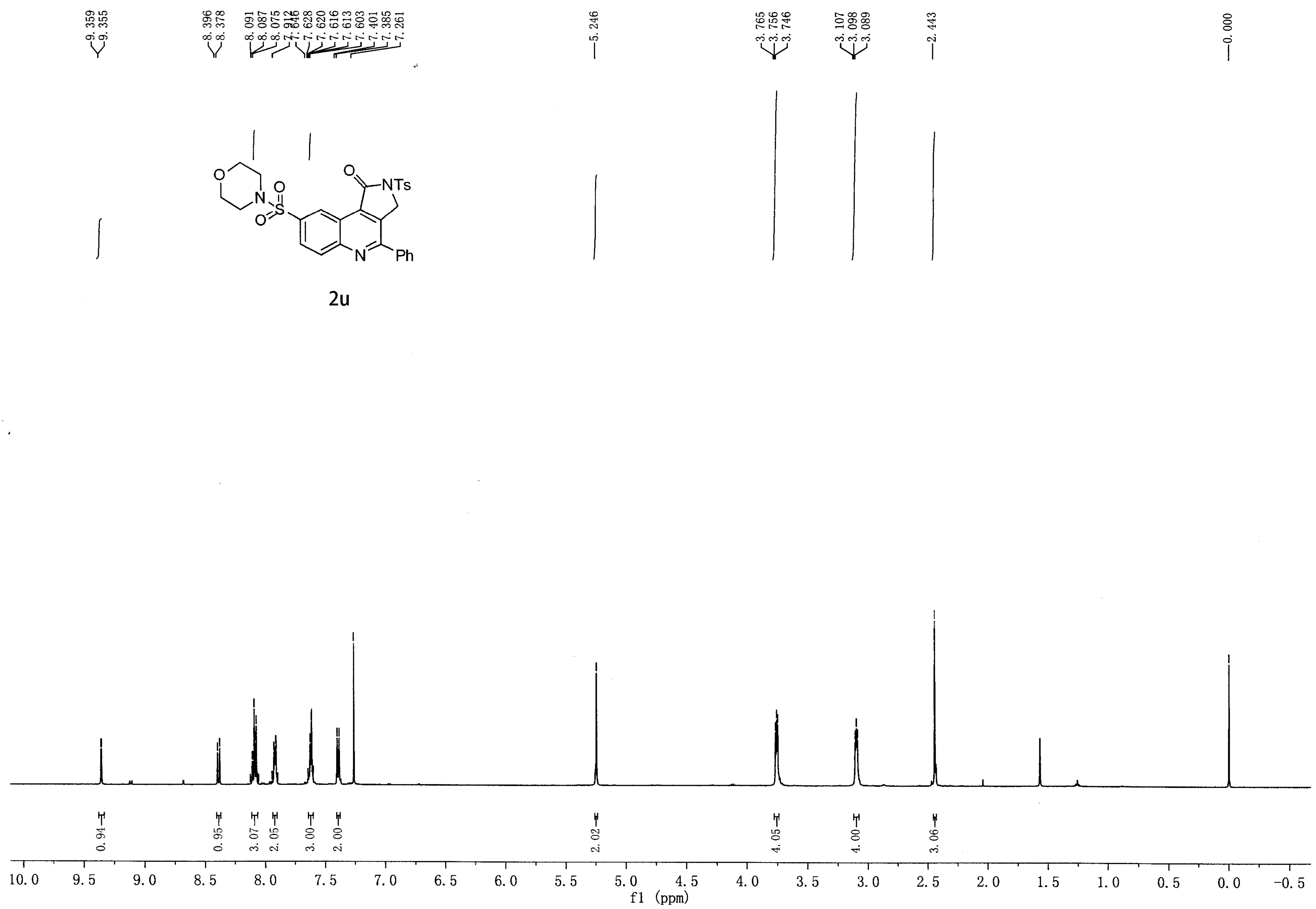

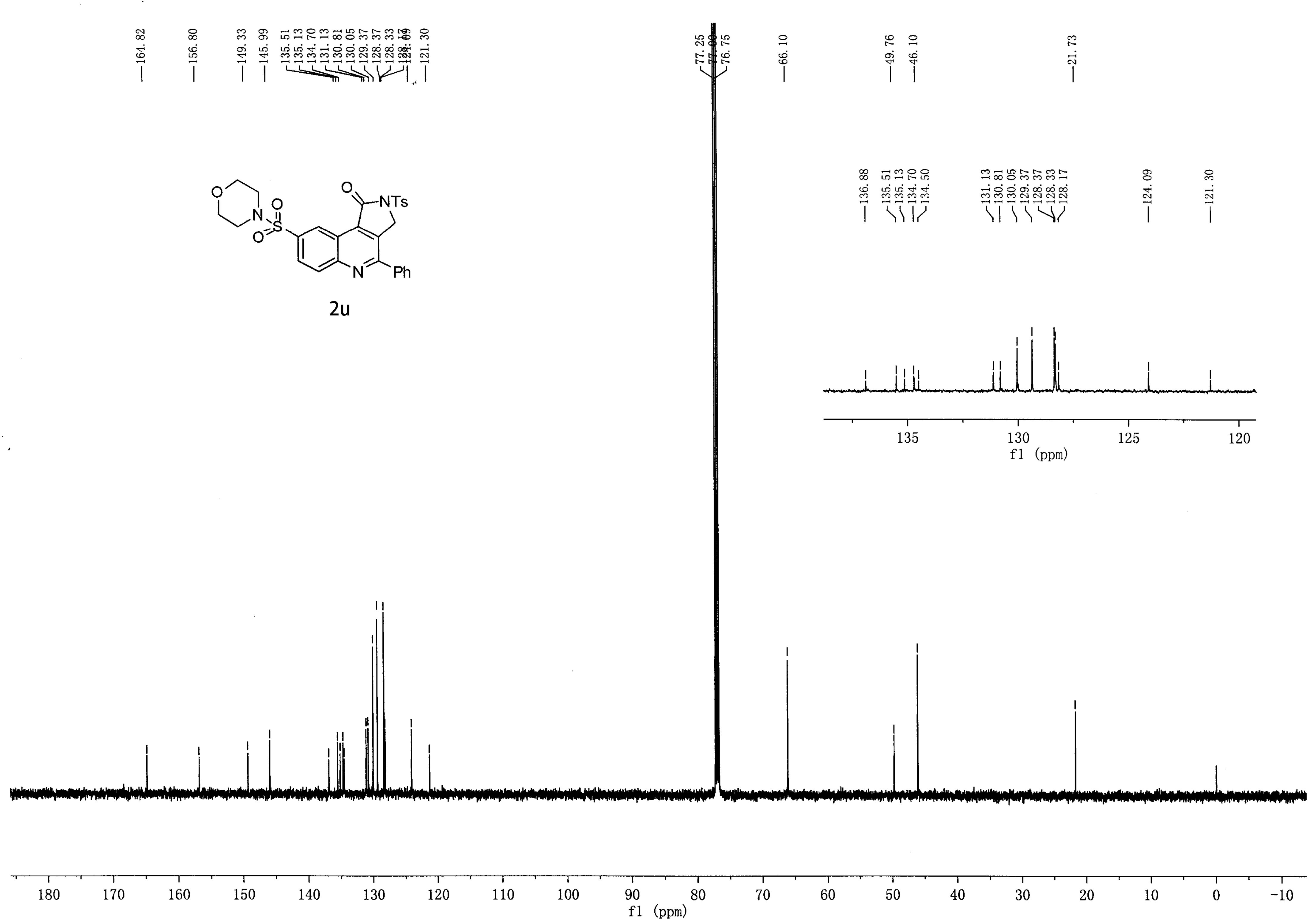

Supplementary Figure 41. ^{1}H and ^{13}C NMR spectra for 2u

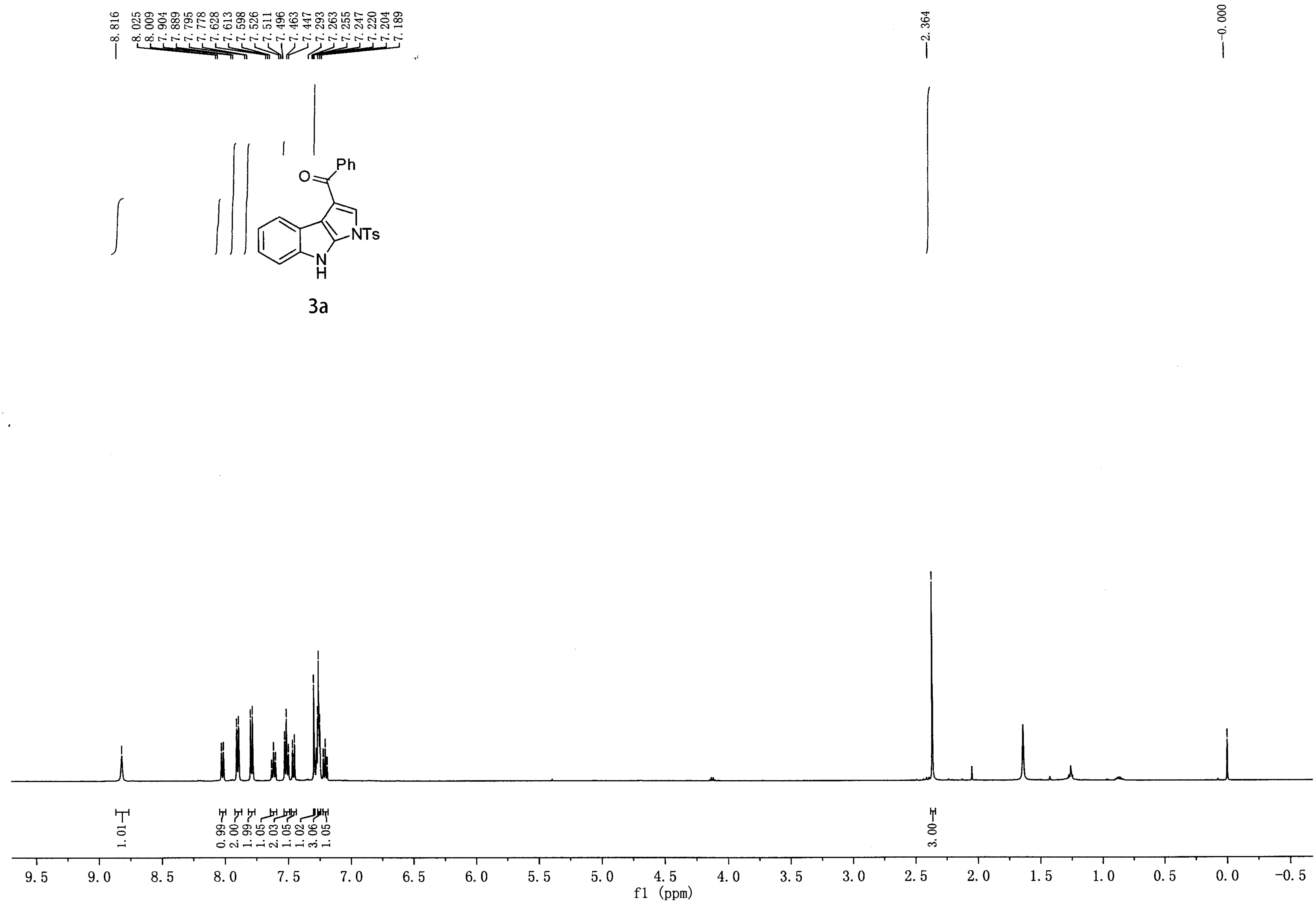

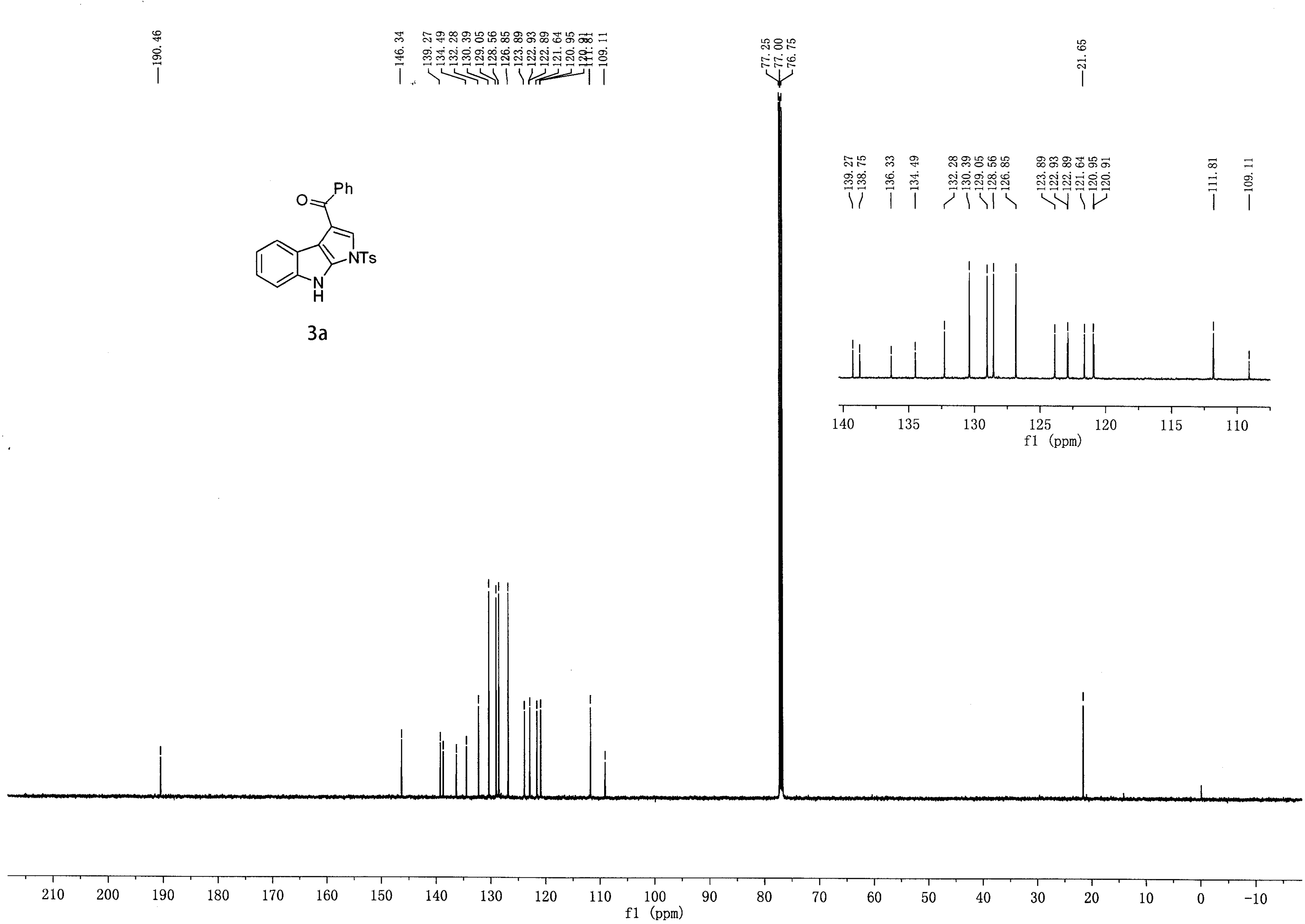

Supplementary Figure 42. ^{1}H and ^{13}C NMR spectra for 3a

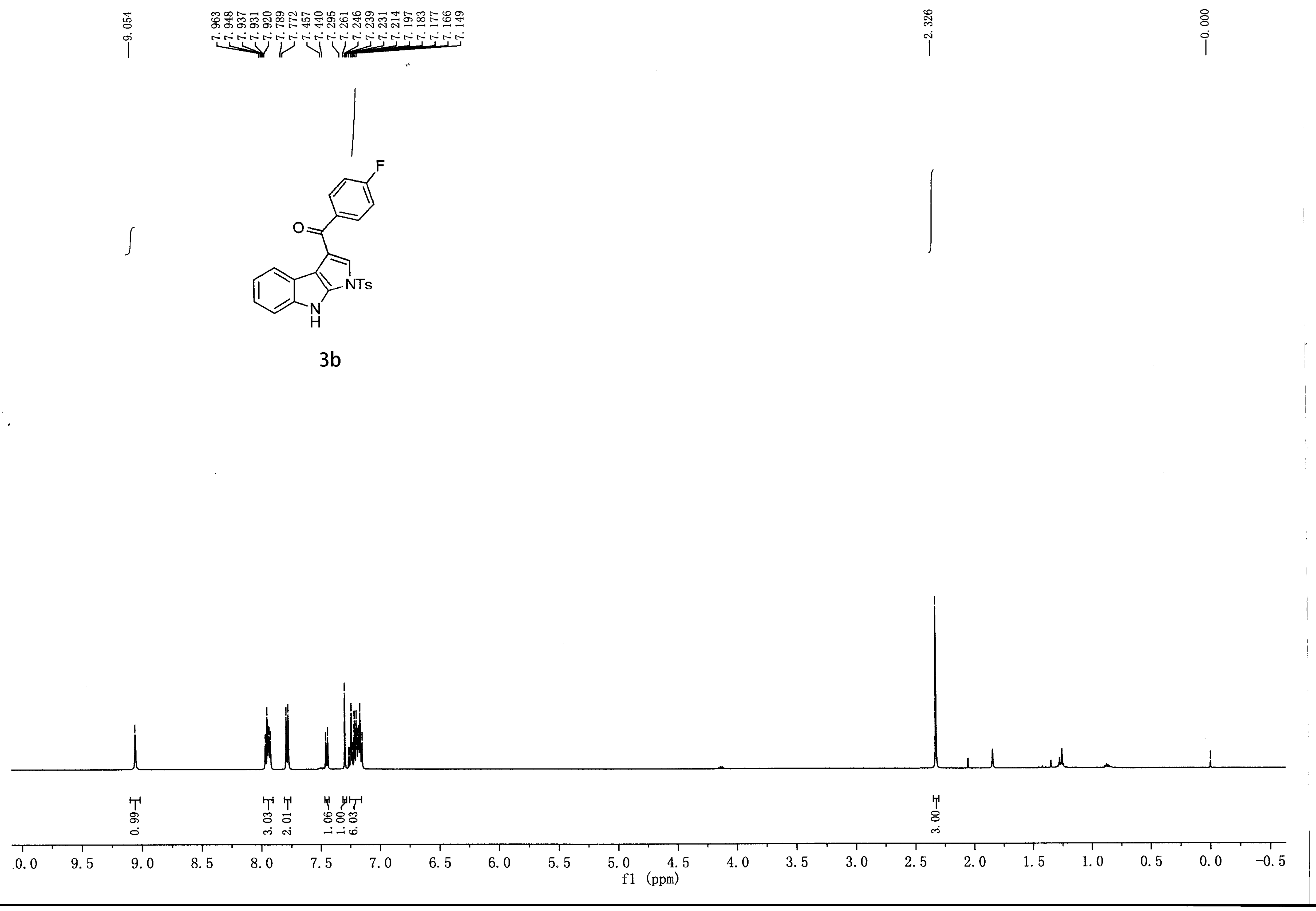

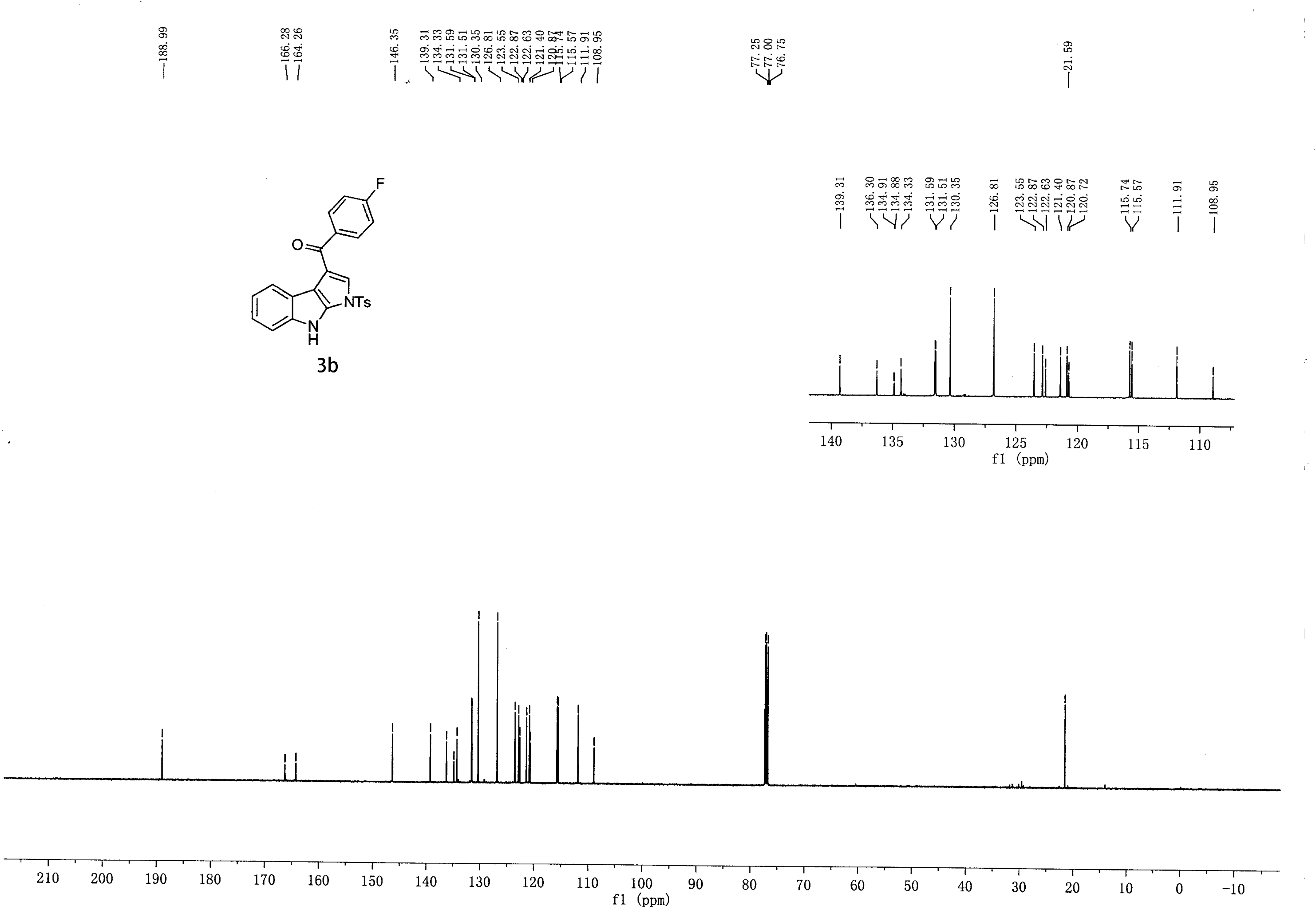

Supplementary Figure 43. ^{1}H and ^{13}C NMR spectra for 3b

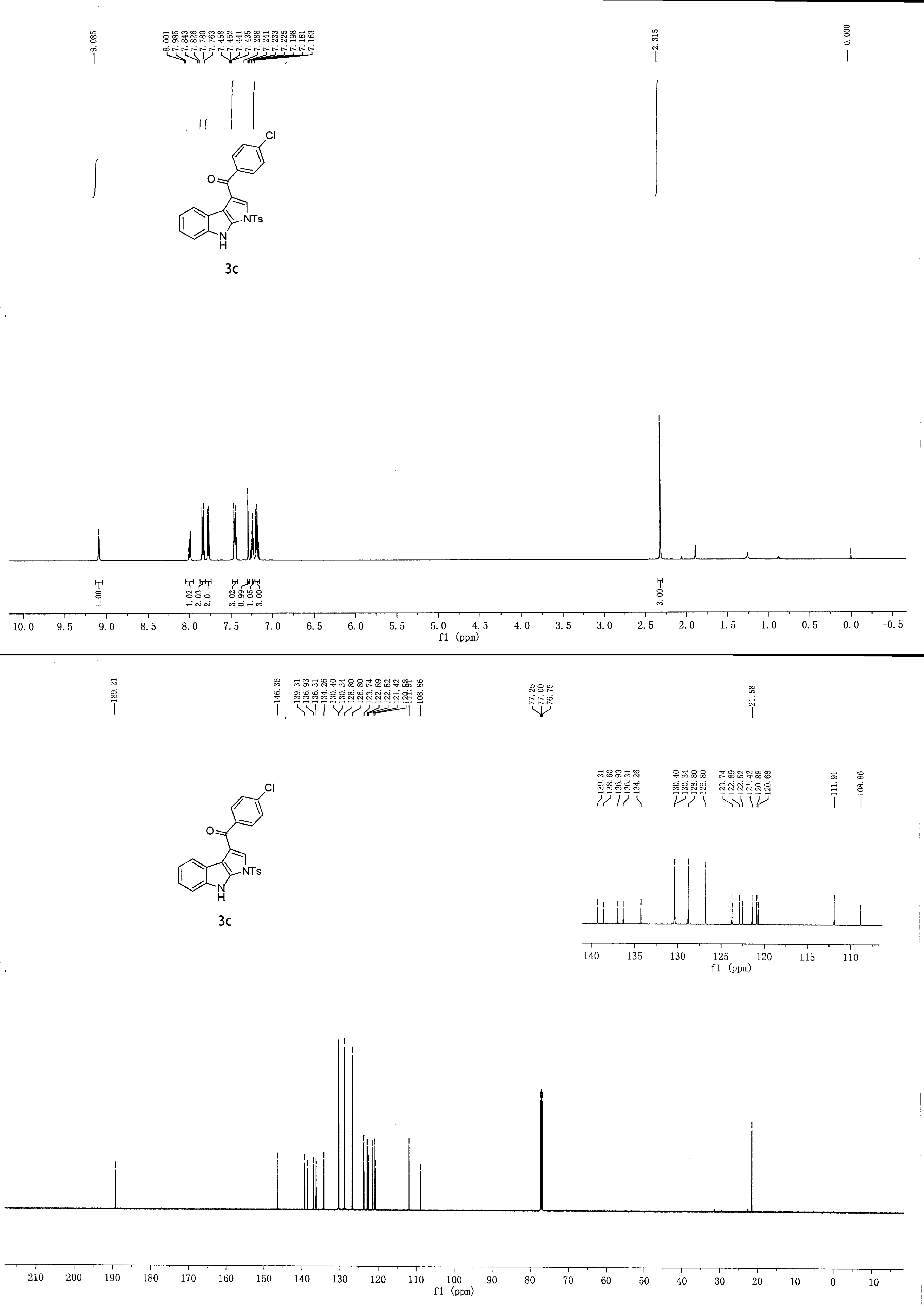

Supplementary Figure 44. ¹H and ¹³C NMR spectra for 3c

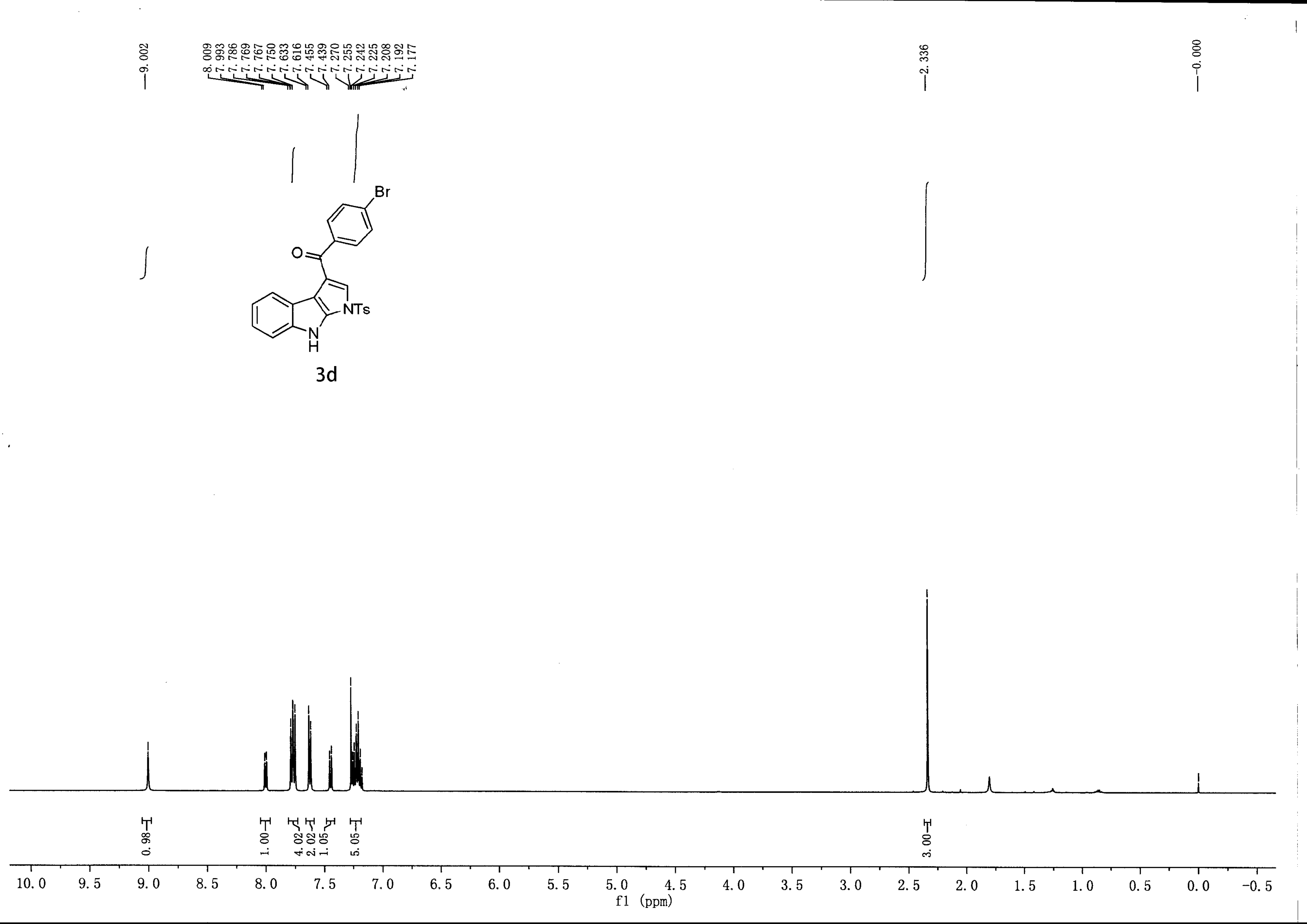

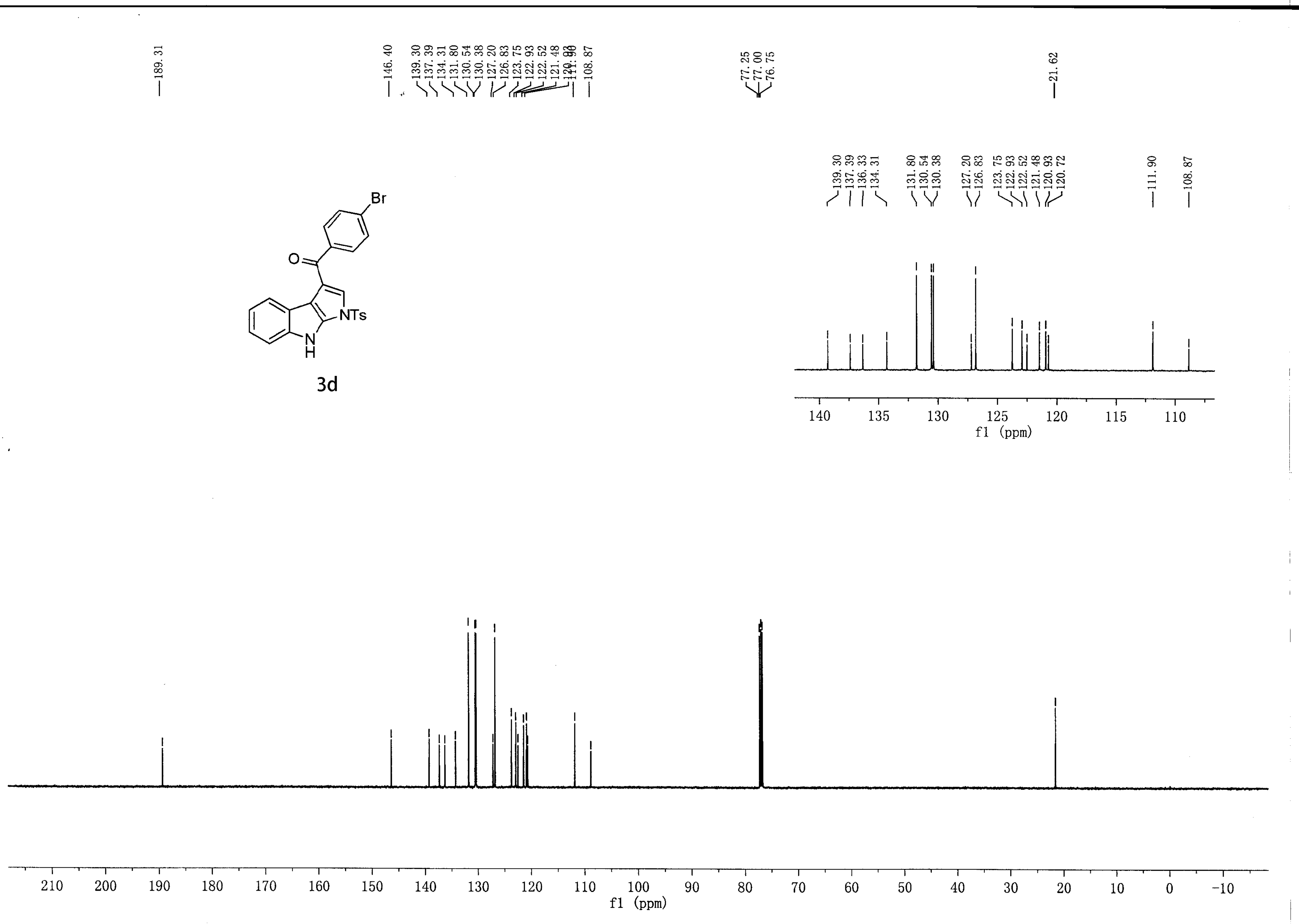

Supplementary Figure 45. ^{1}H and ^{13}C NMR spectra for 3d

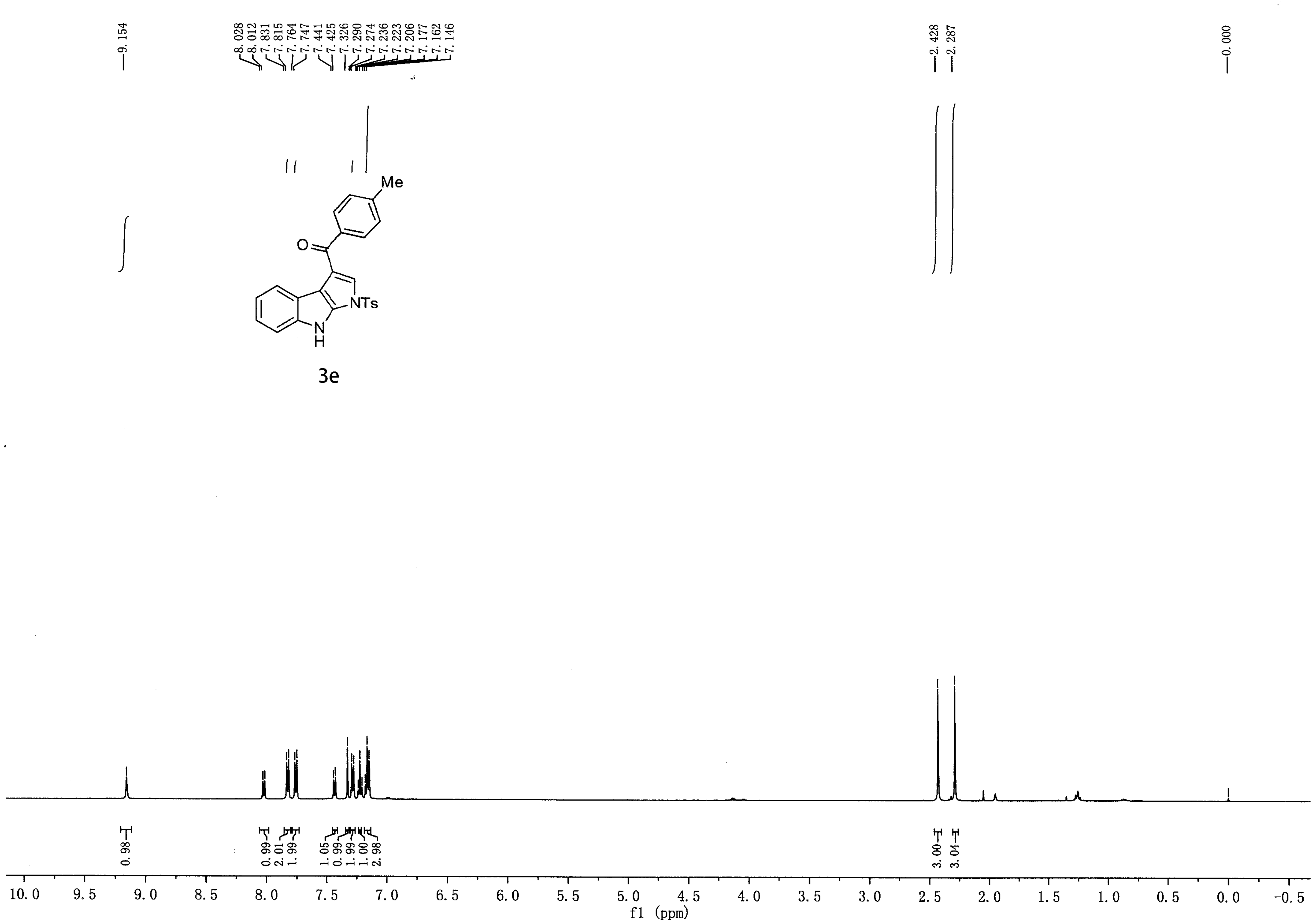

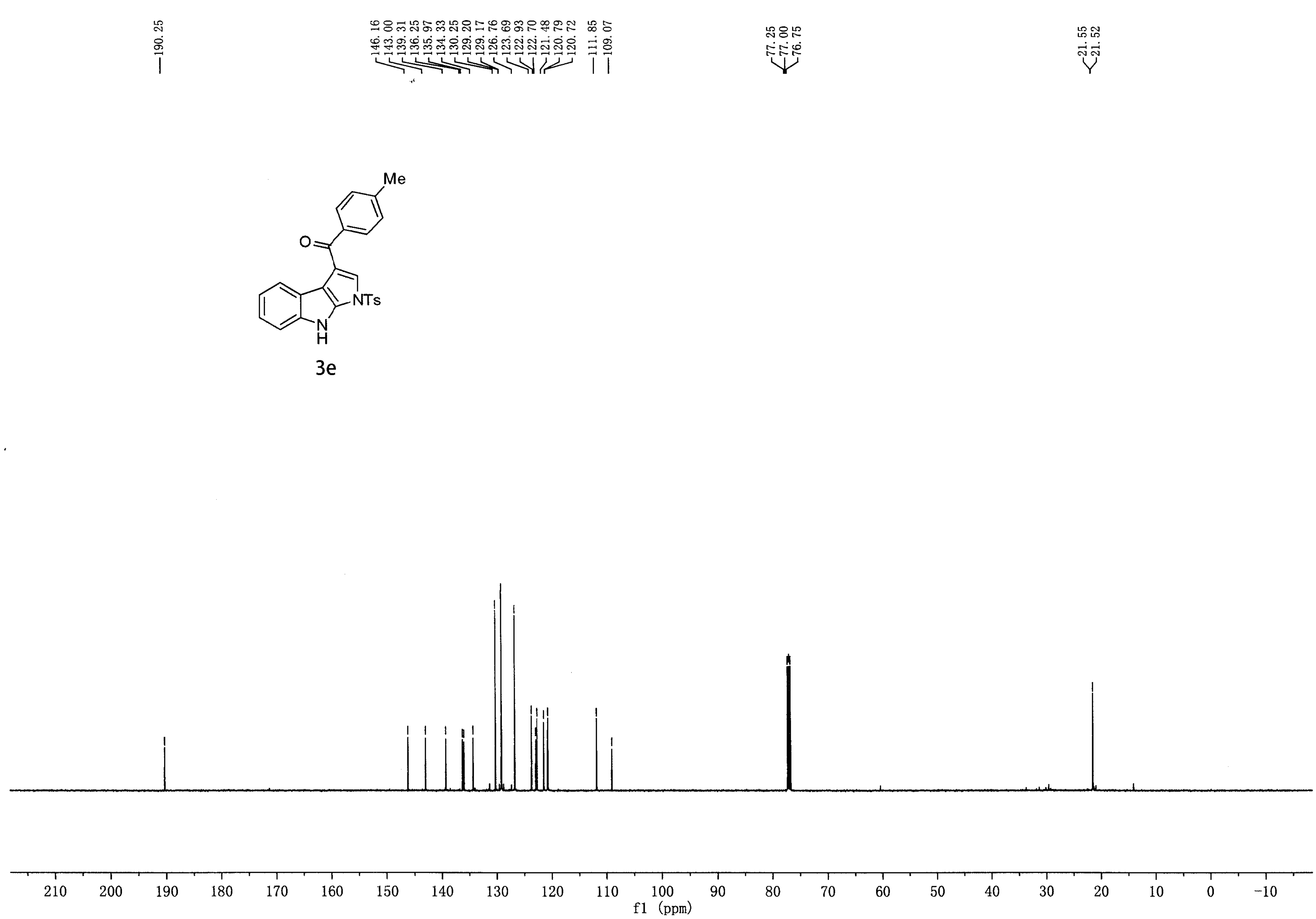

Supplementary Figure 46. ^{1}H and ^{13}C NMR spectra for 3e

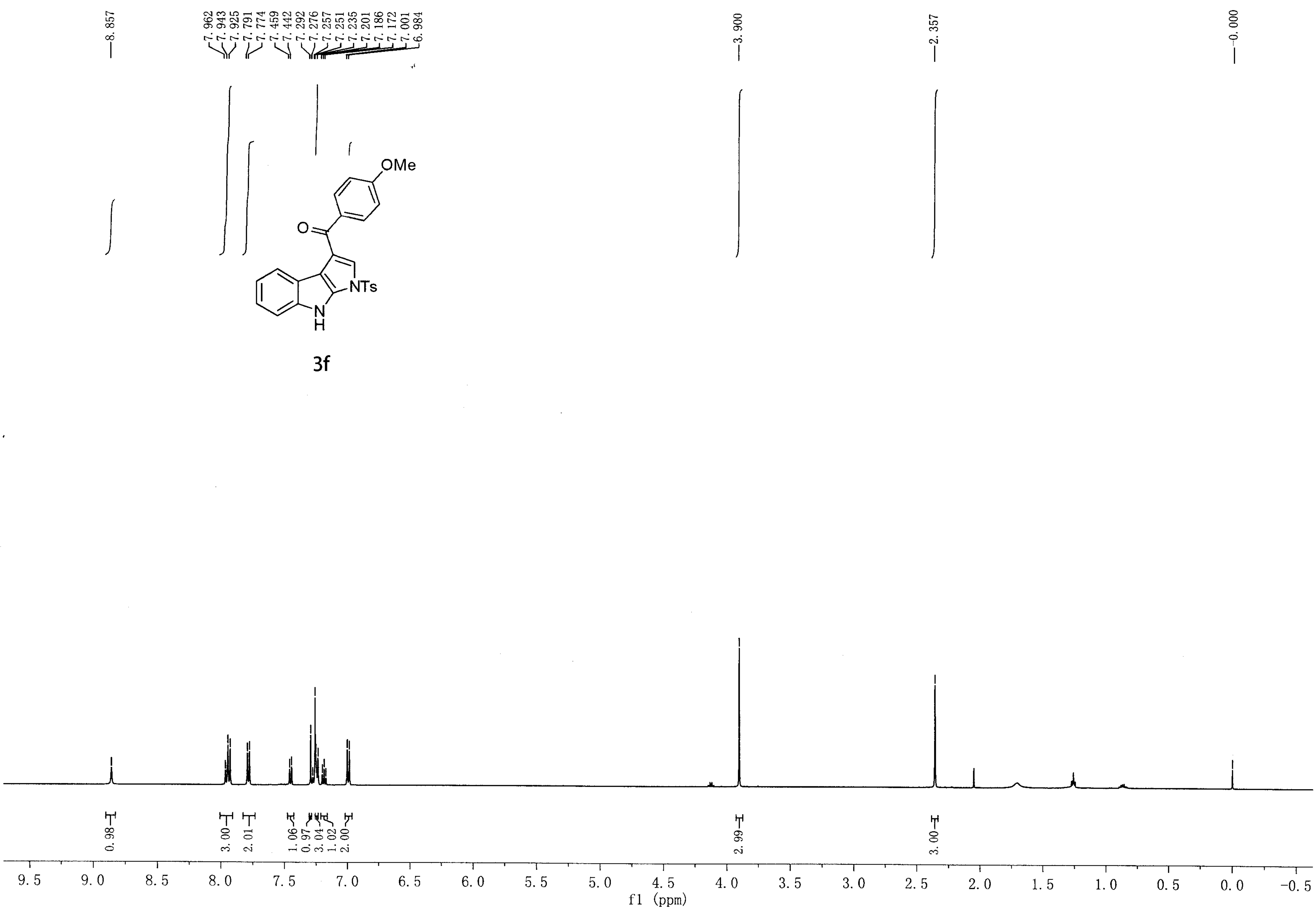

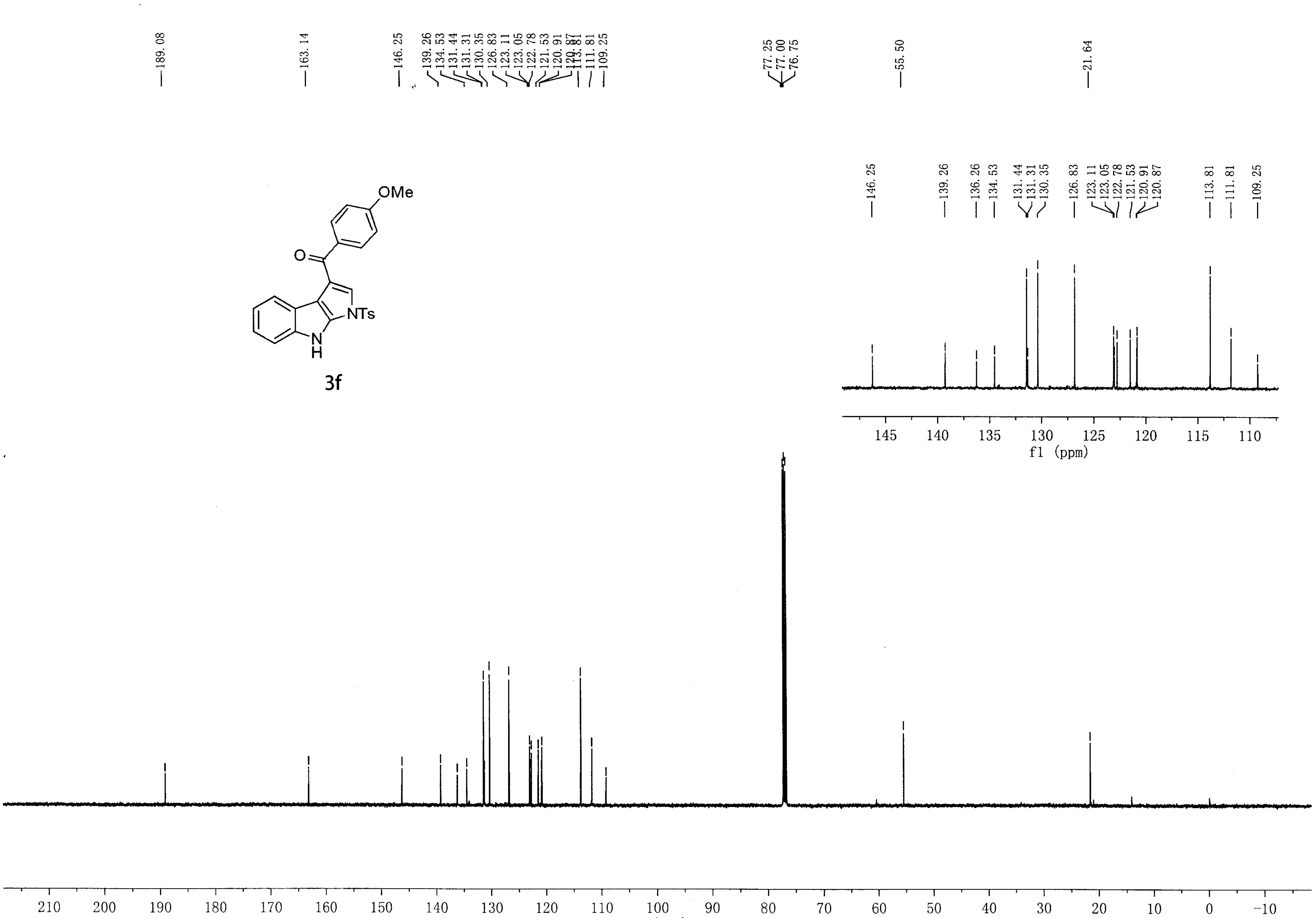

Supplementary Figure 47. ^{1}H and ^{13}C NMR spectra for 3f

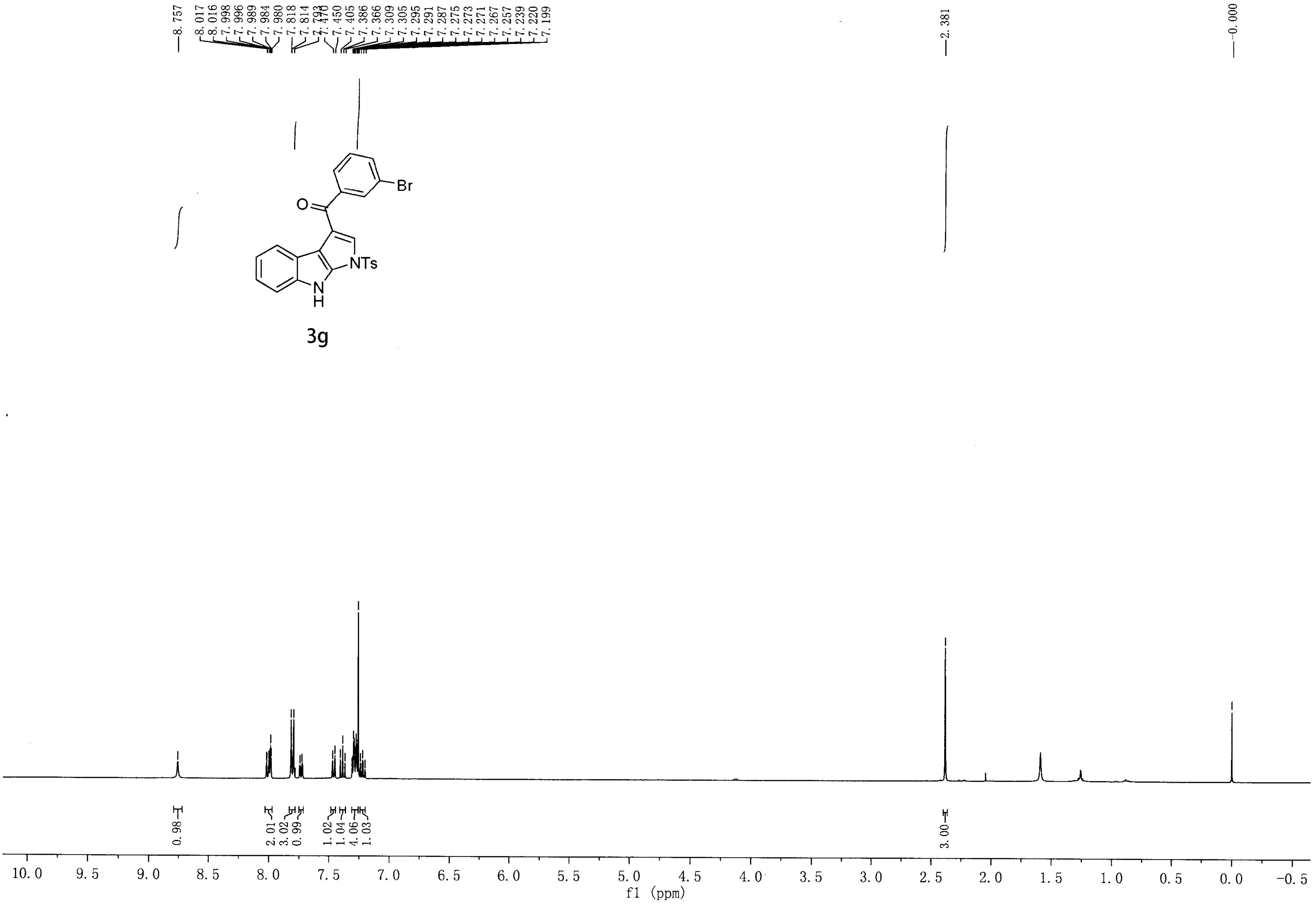

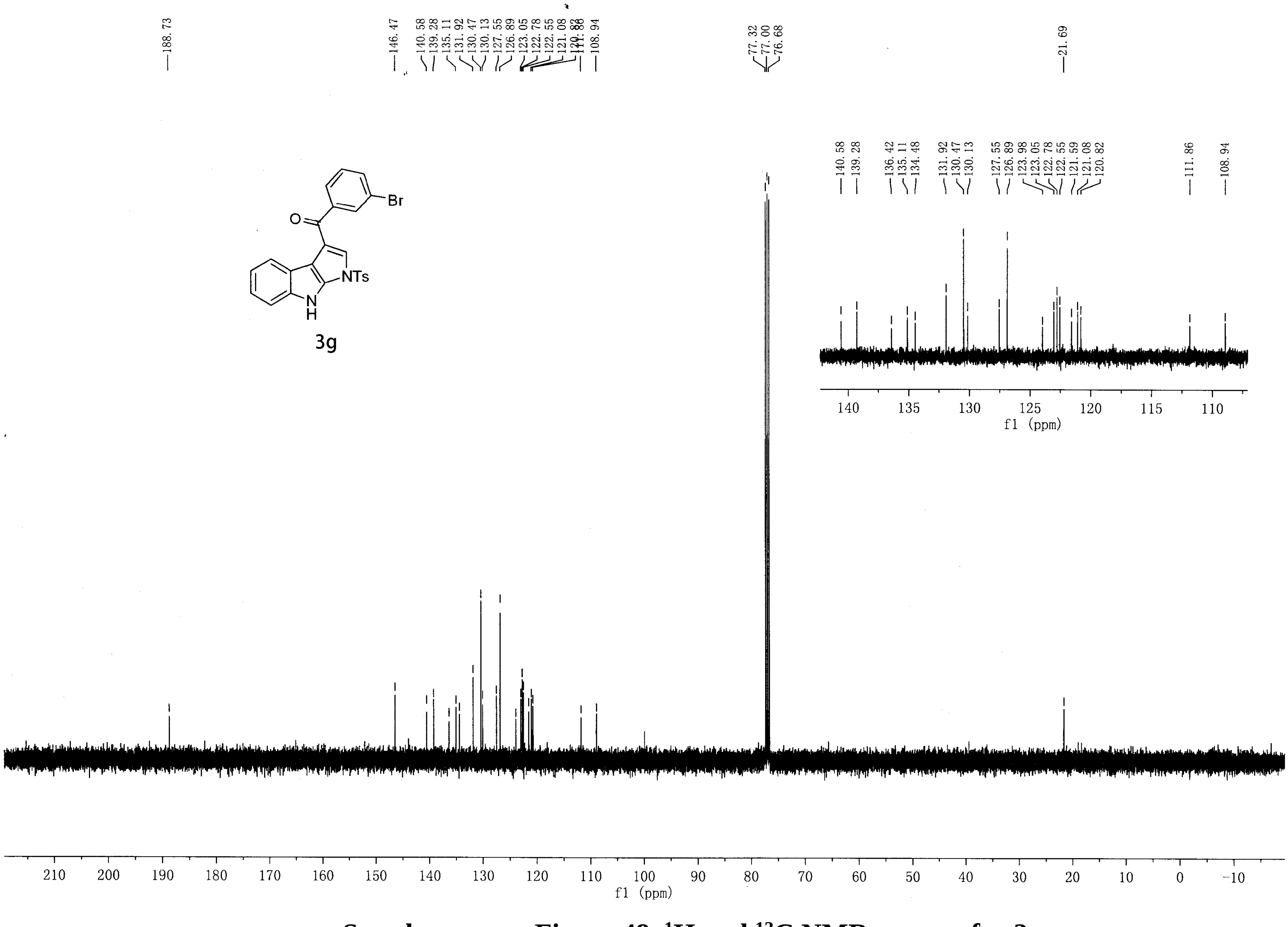

Supplementary Figure 48. ^{1}H and ^{13}C NMR spectra for 3g

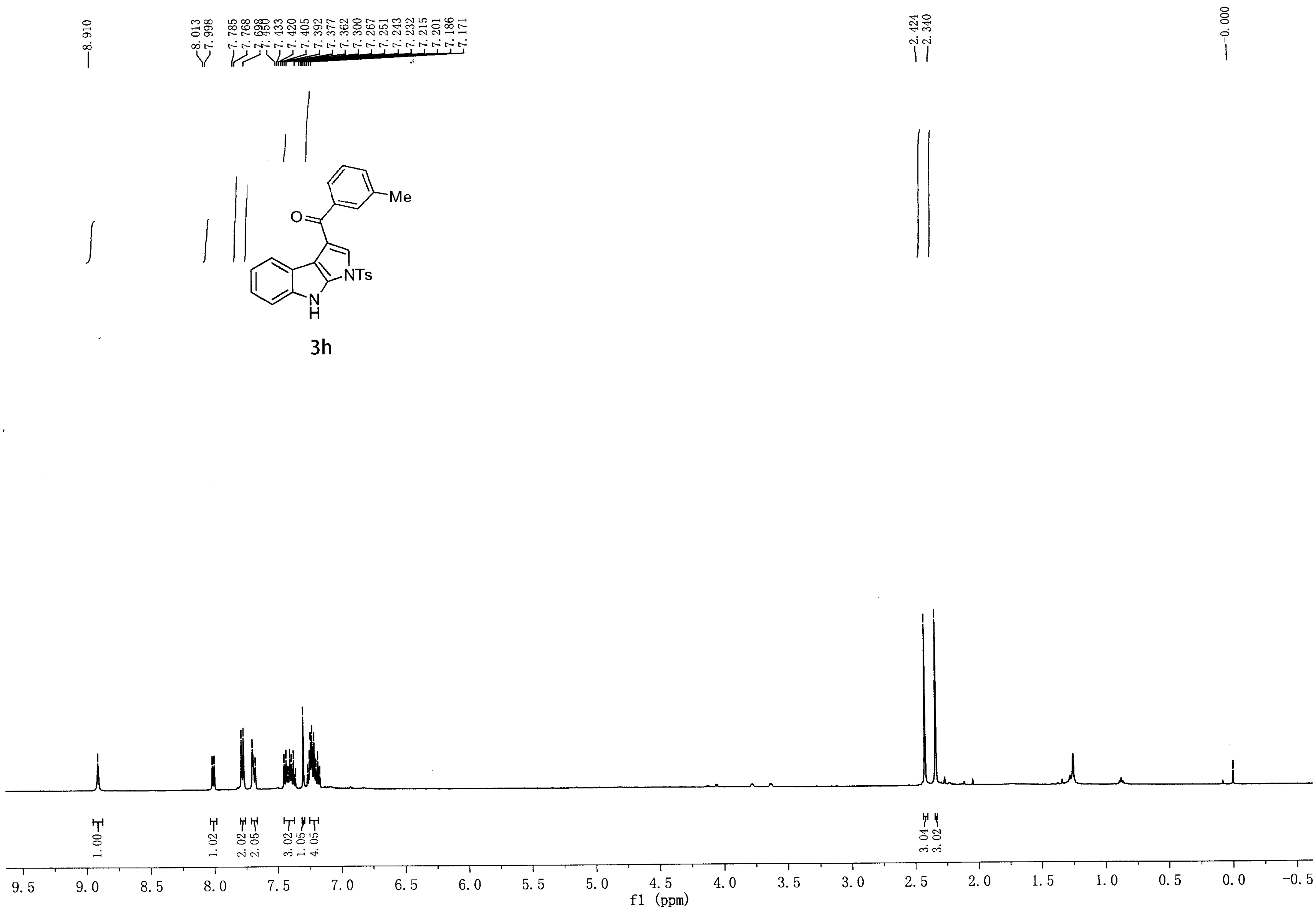

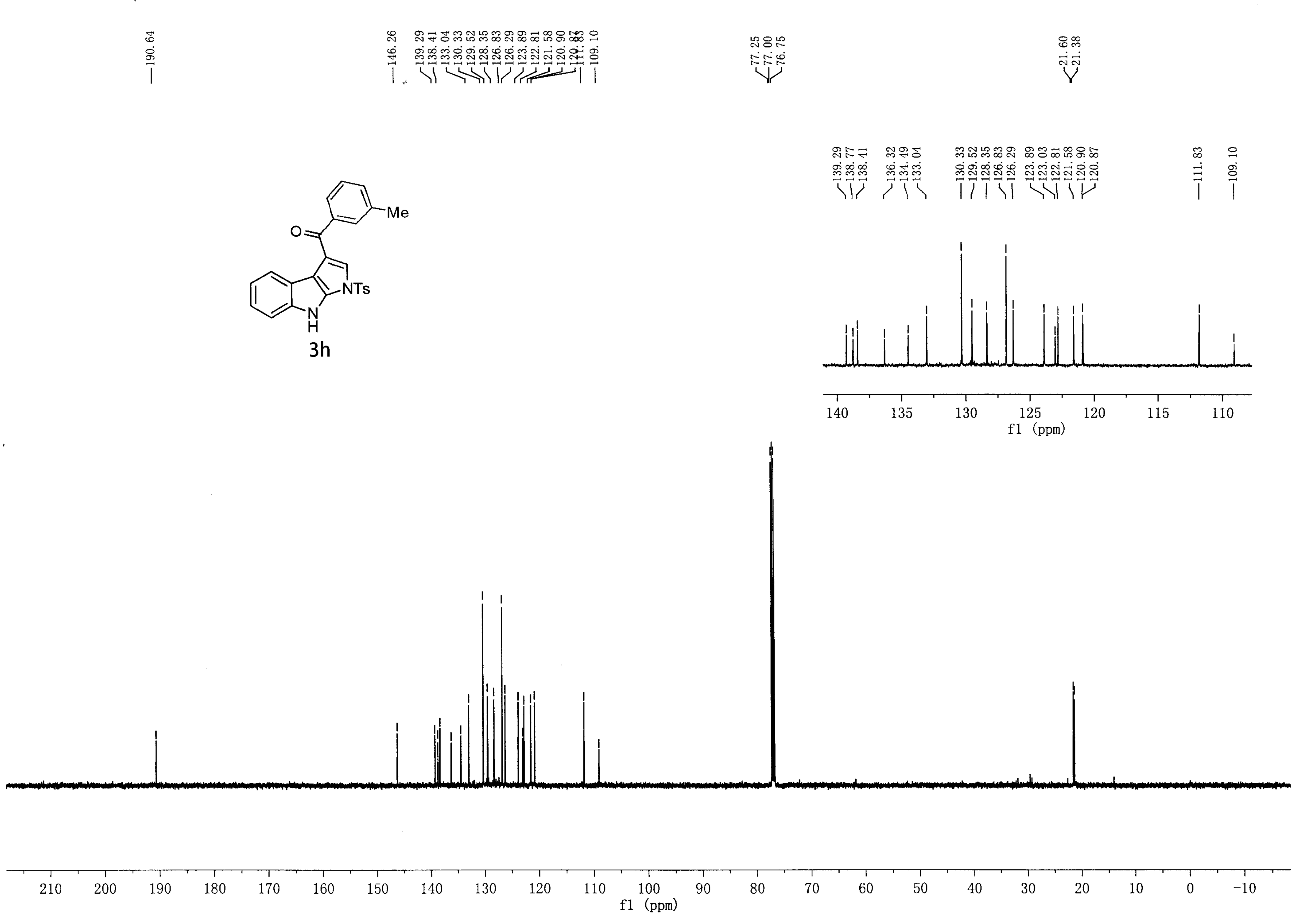

Supplementary Figure 49. ^{1}H and ^{13}C NMR spectra for 3h

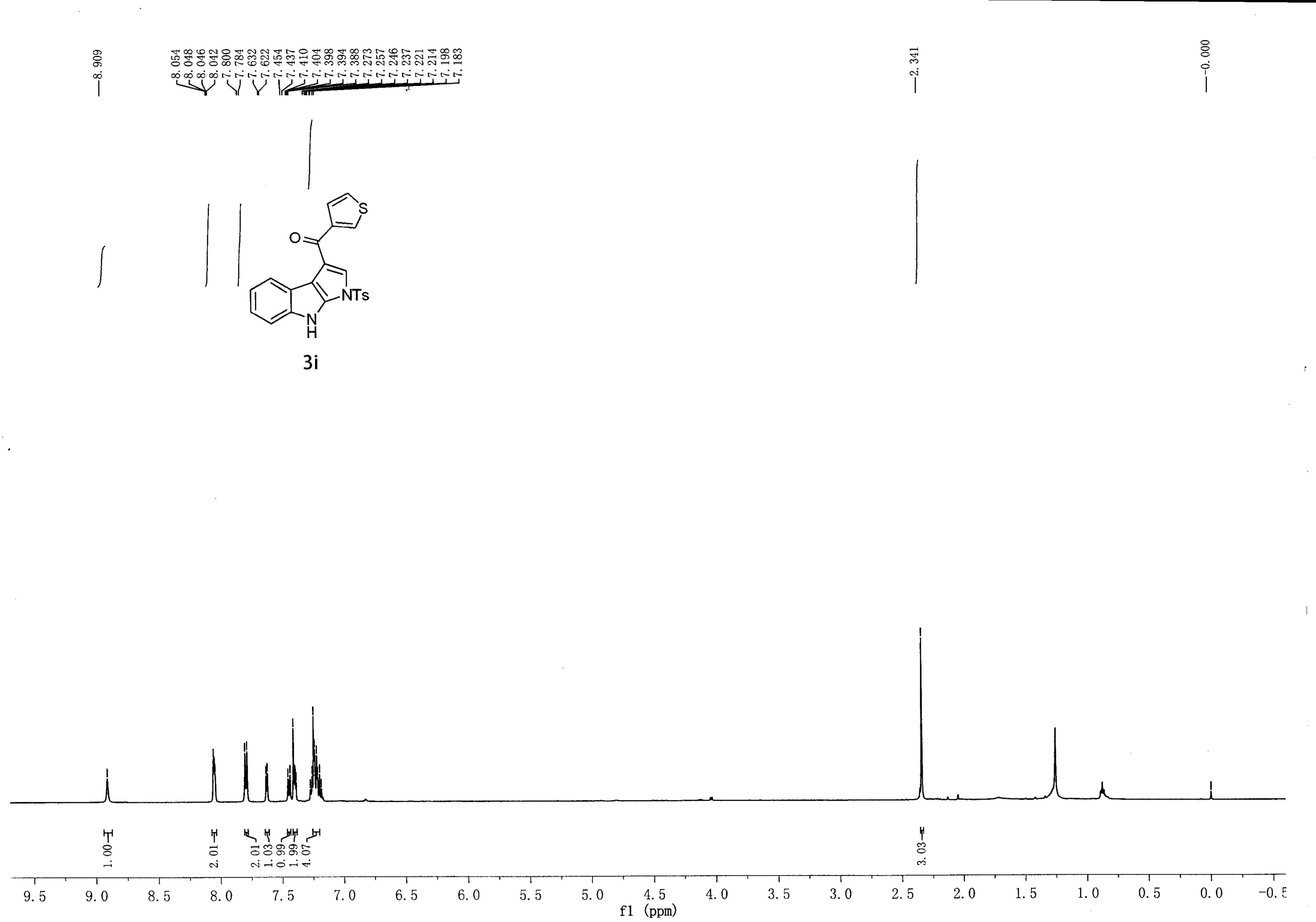

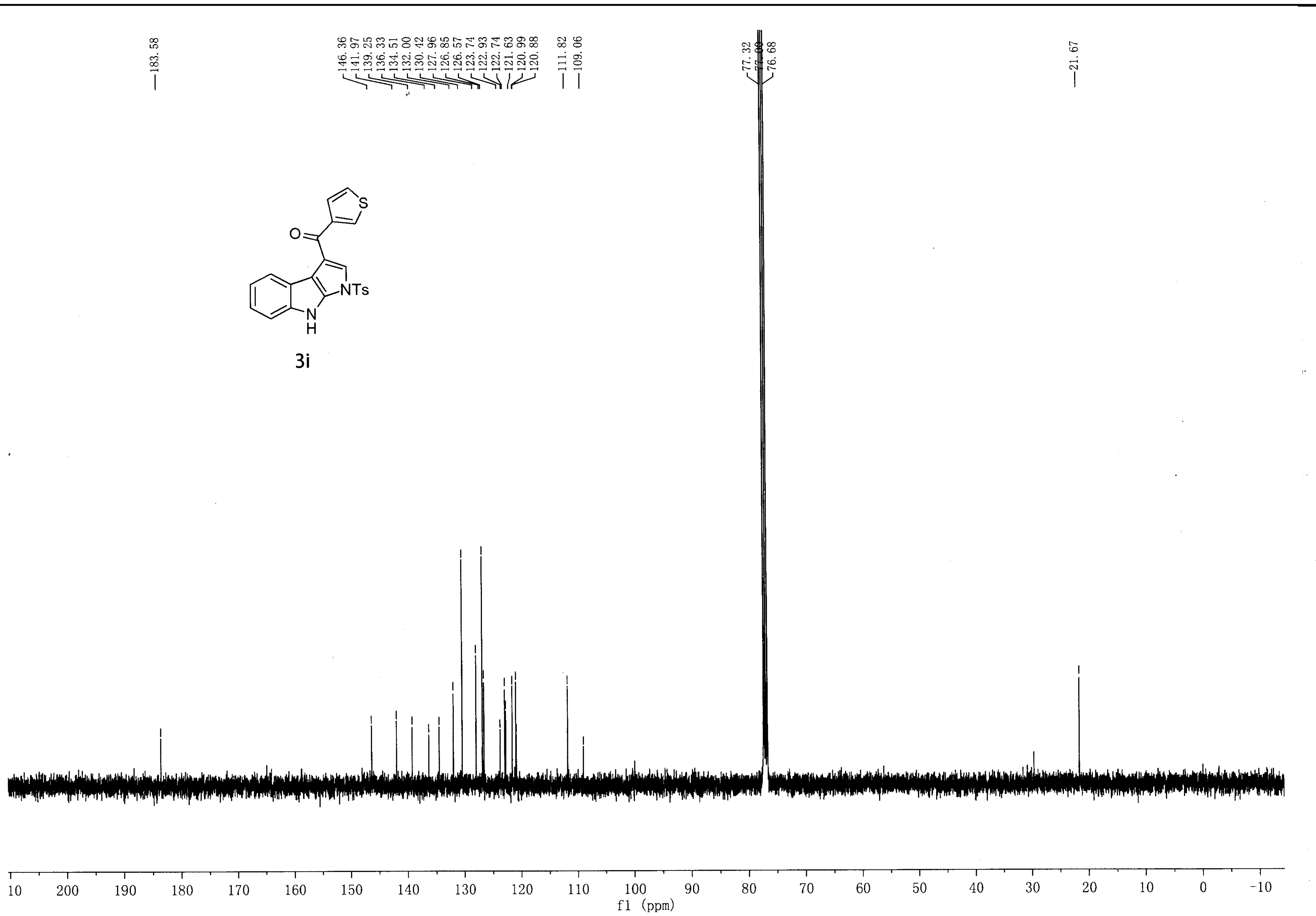

Supplementary Figure 50. ^{1}H **and** ^{13}C **NMR spectra for 3i**

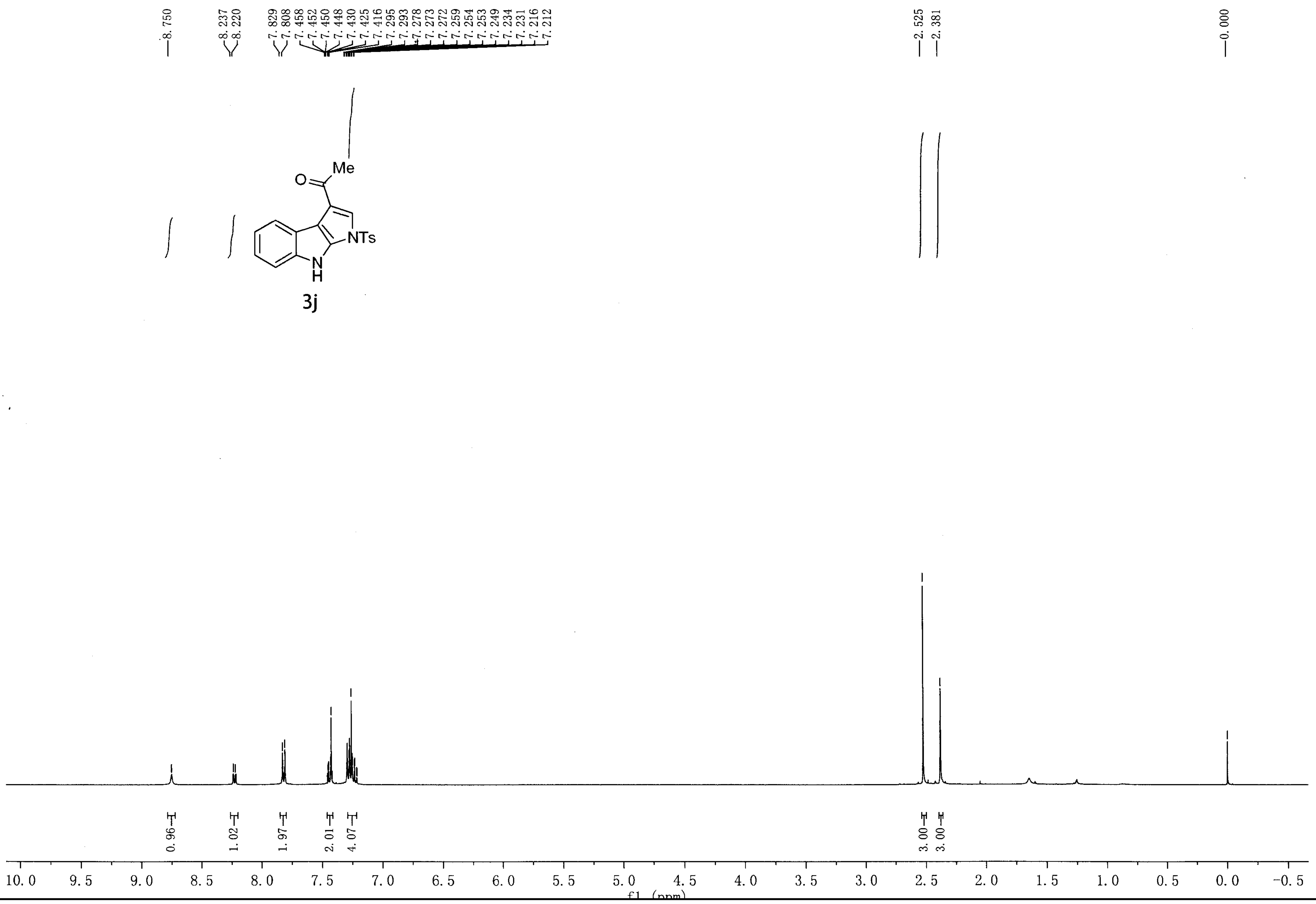

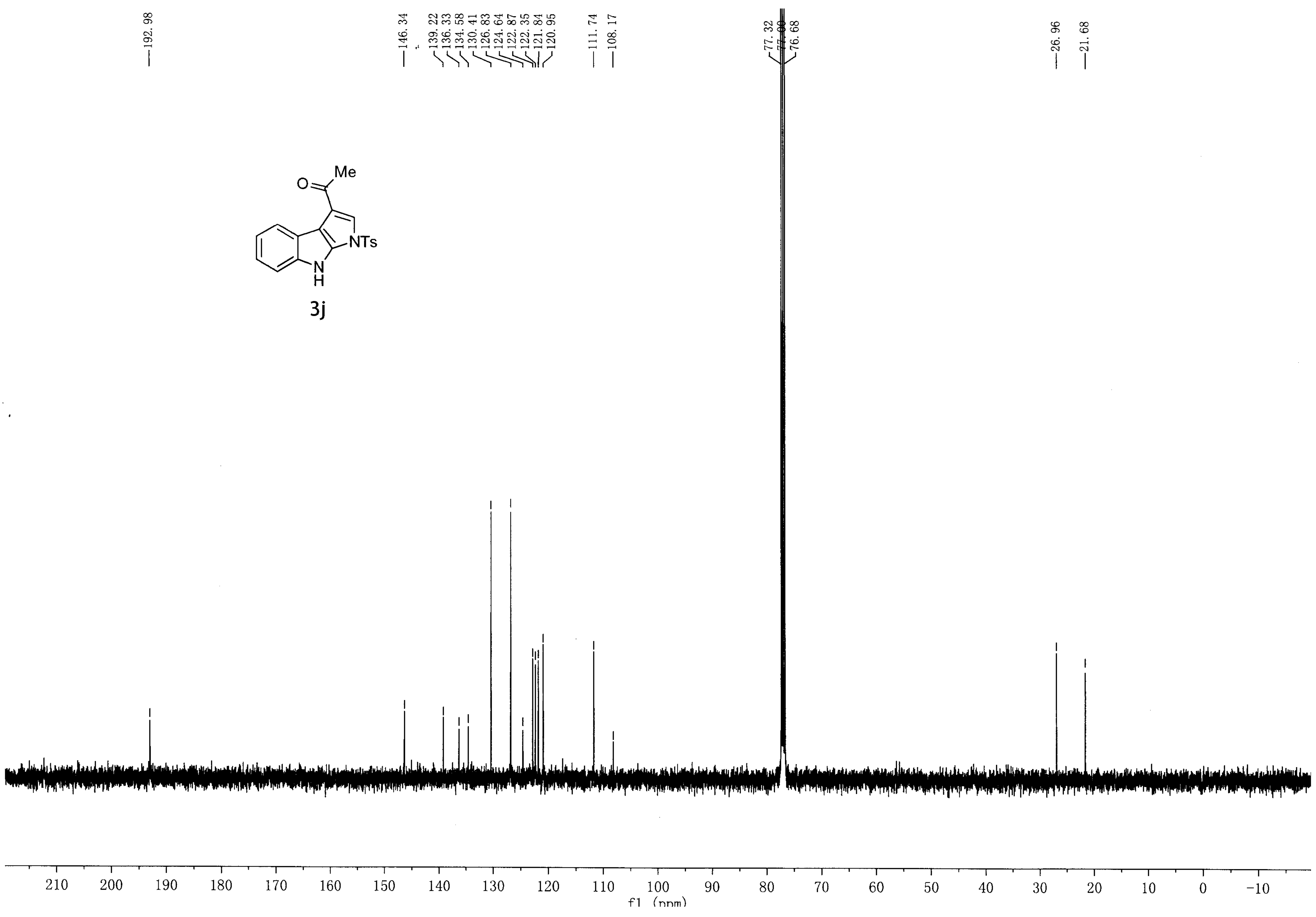

Supplementary Figure 51. ^{1}H and ^{13}C NMR spectra for 3j

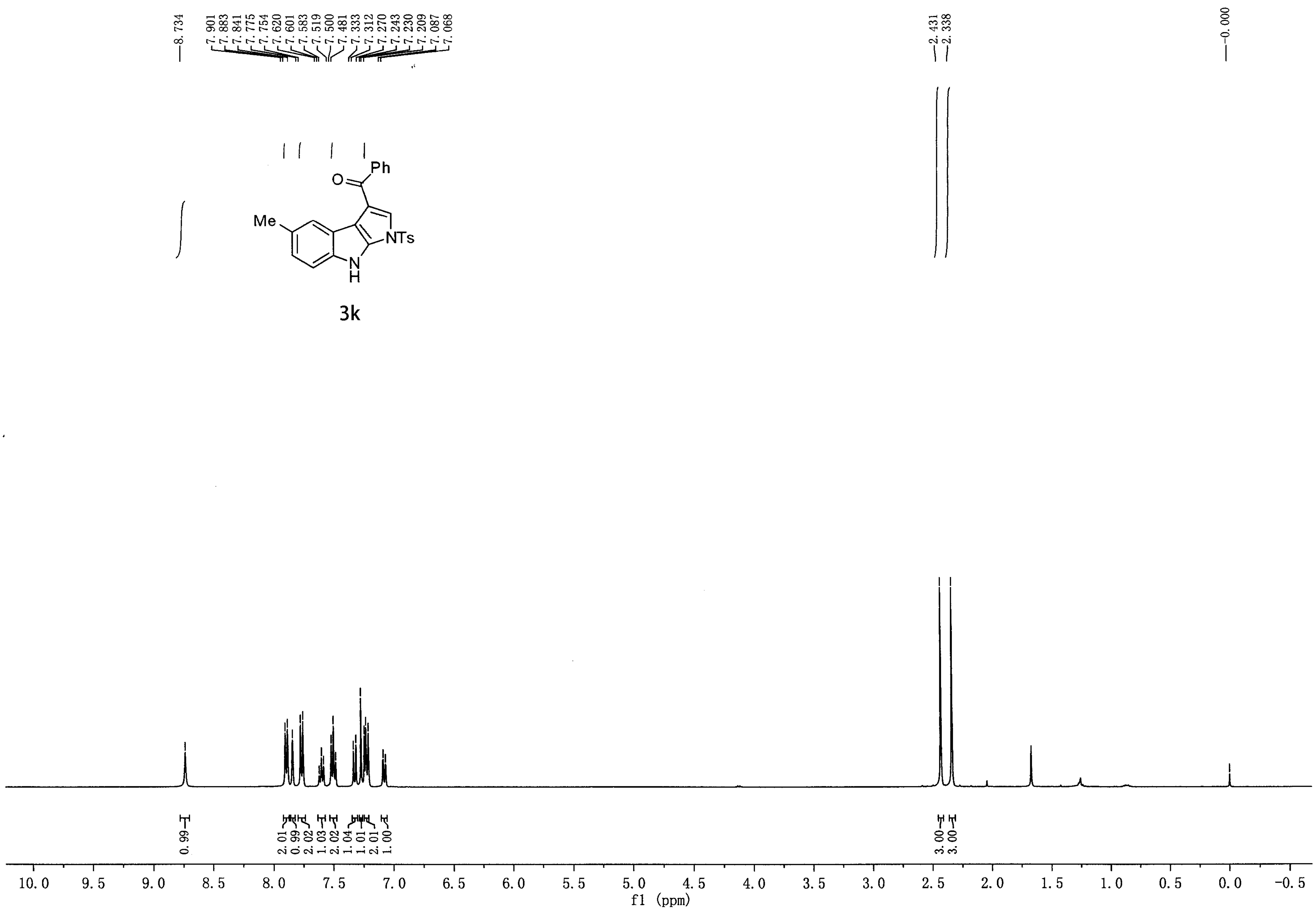

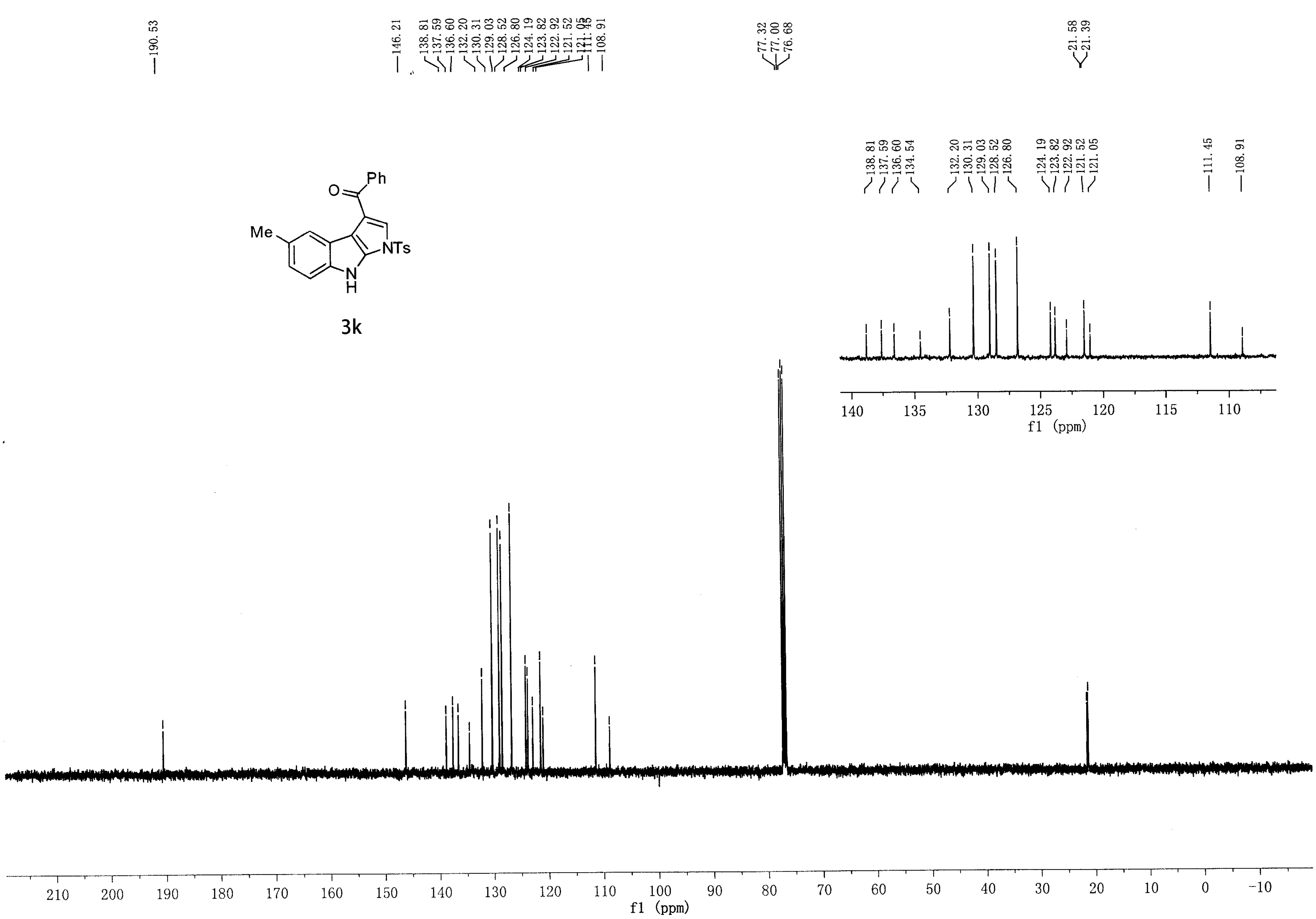

Supplementary Figure 52. ^{1}H and ^{13}C NMR spectra for 3k

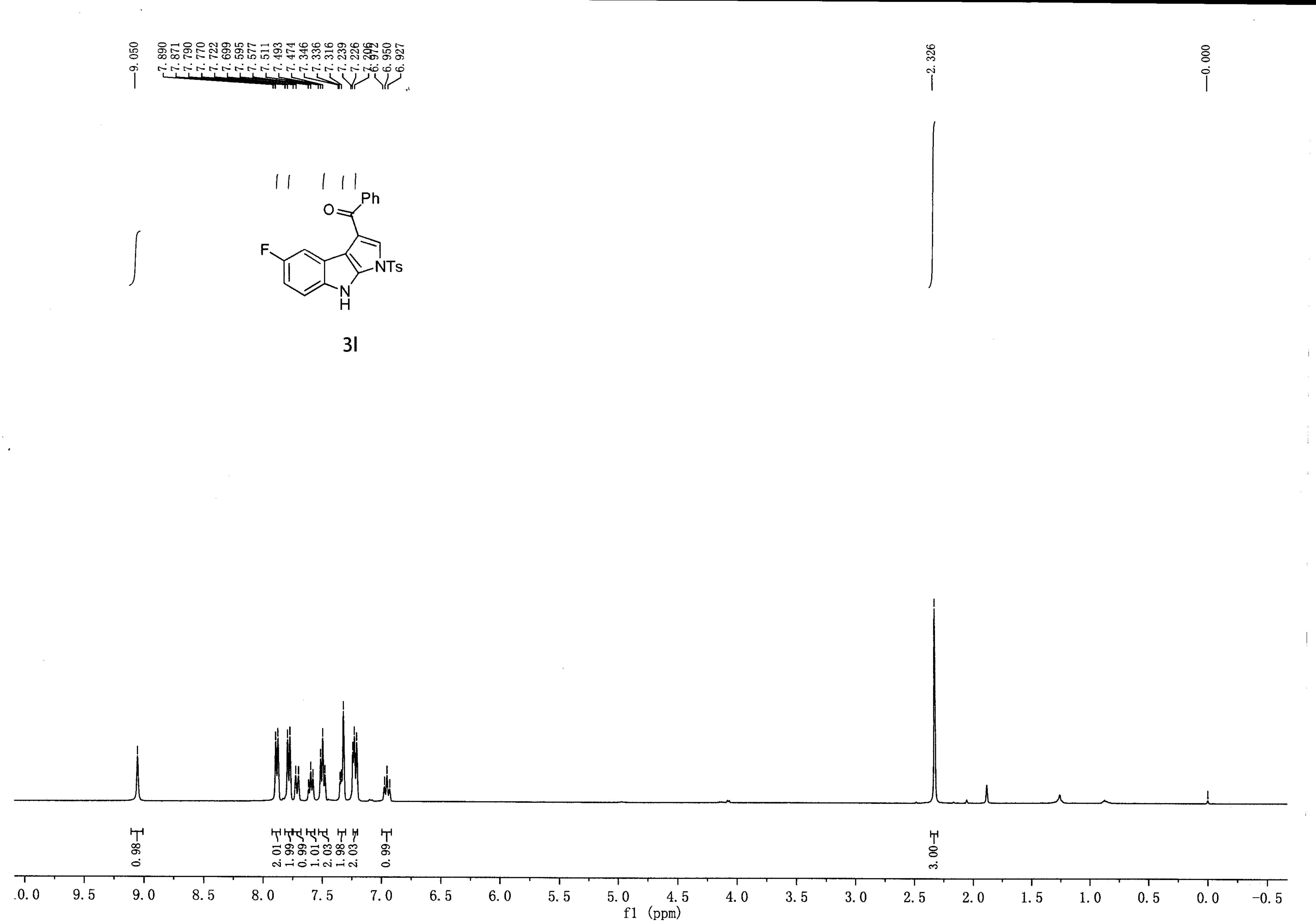

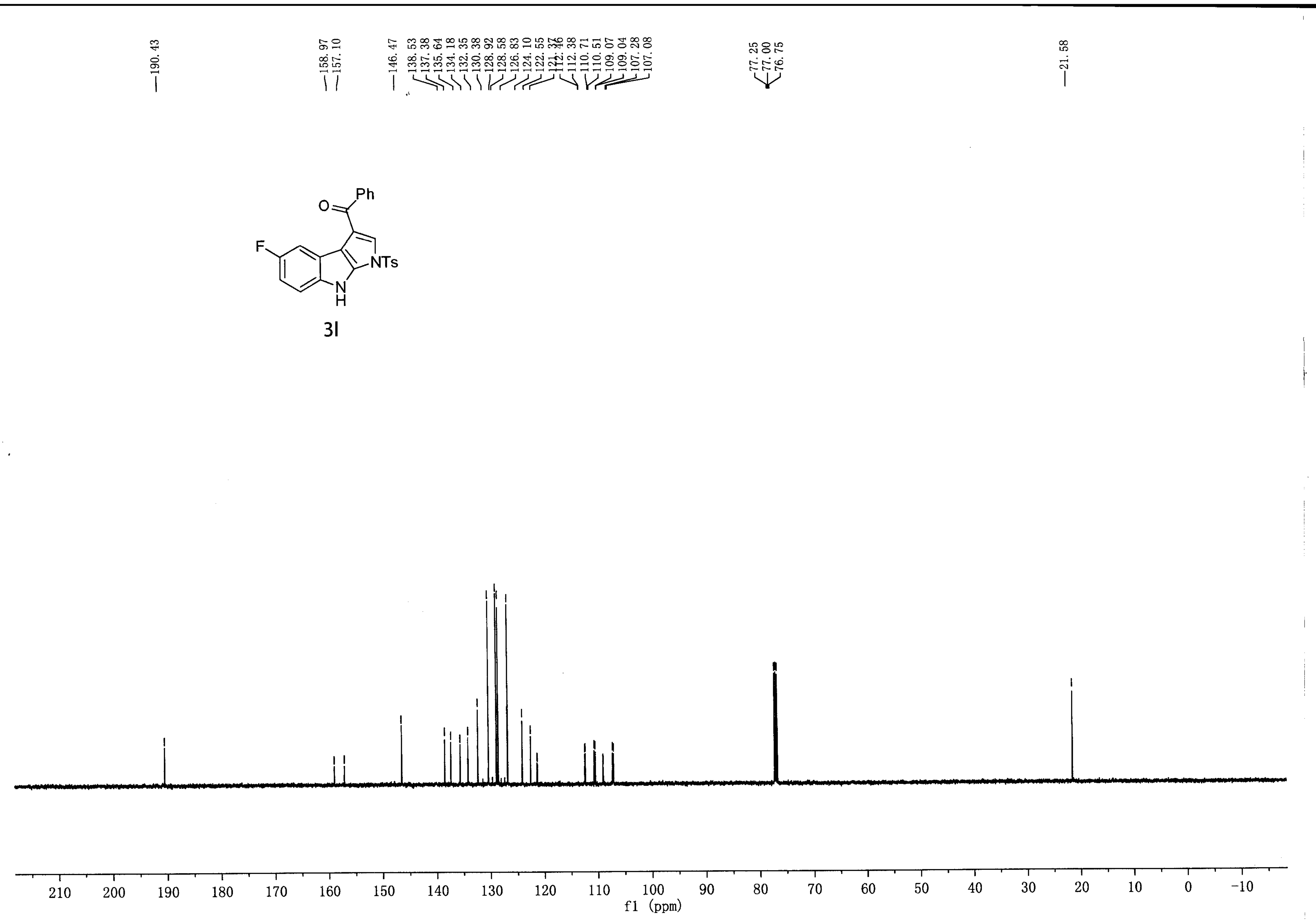

Supplementary Figure 53. ^{1}H and ^{13}C NMR spectra for 3l

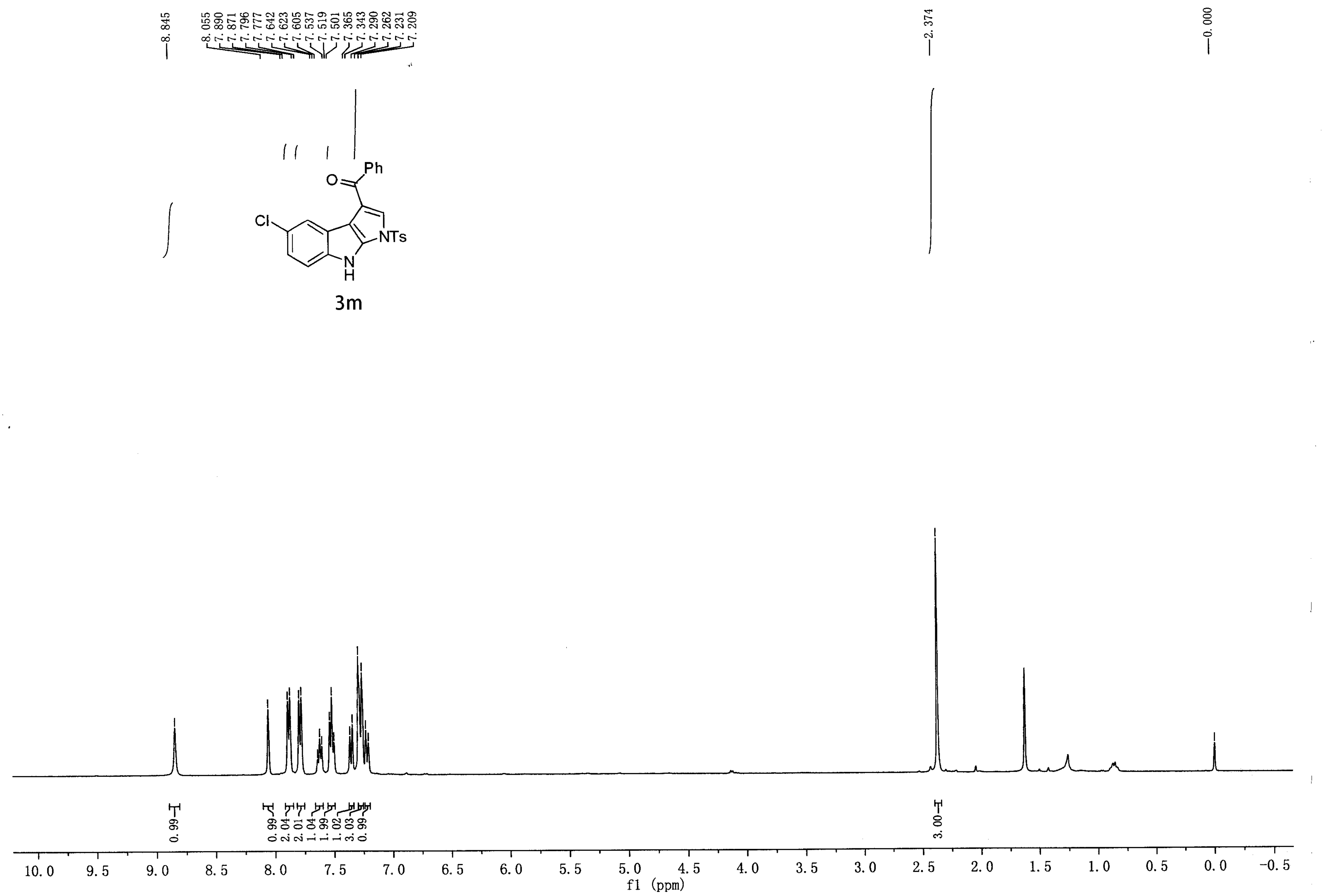

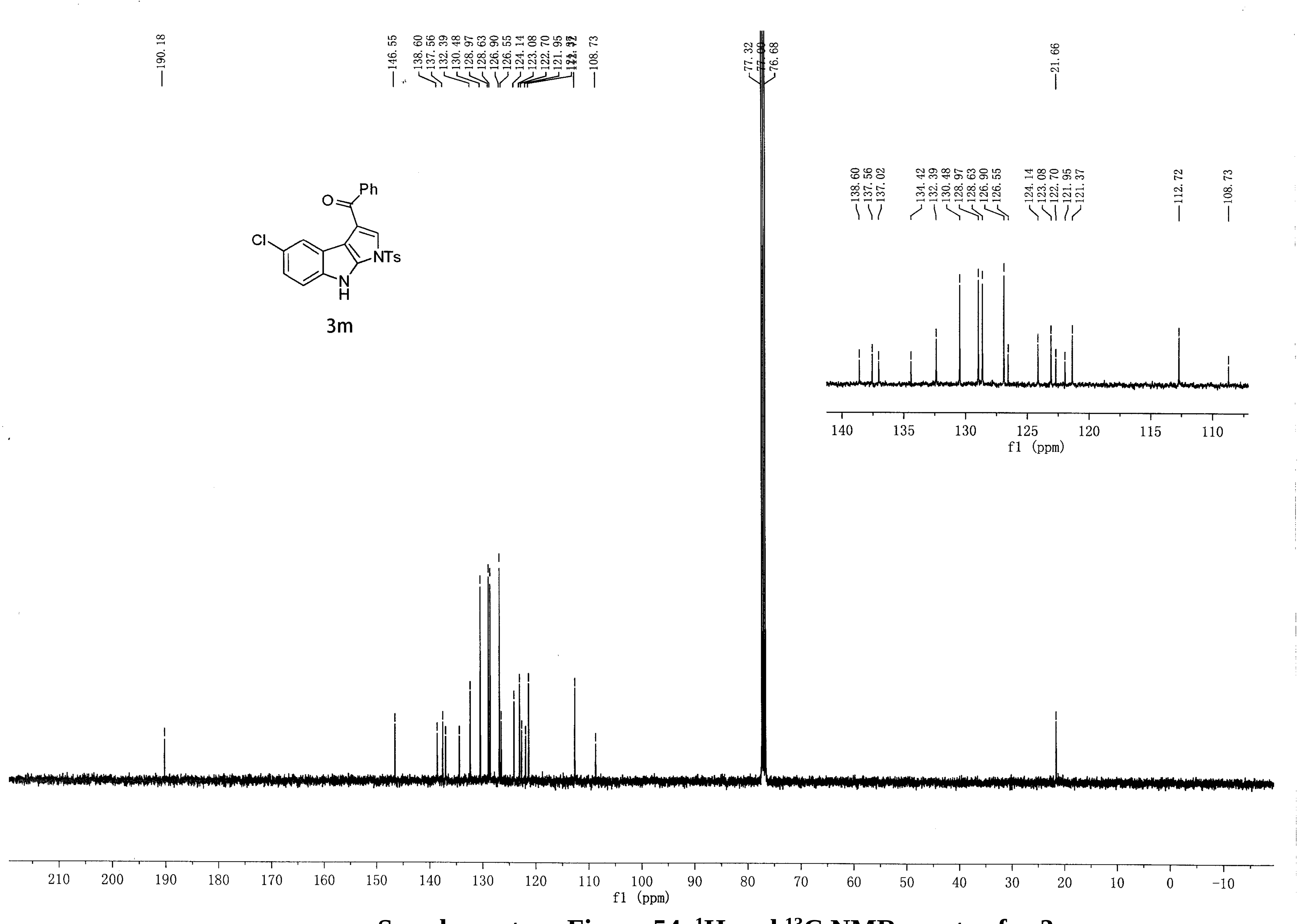

Supplementary Figure 54. ^{1}H and ^{13}C NMR spectra for 3m

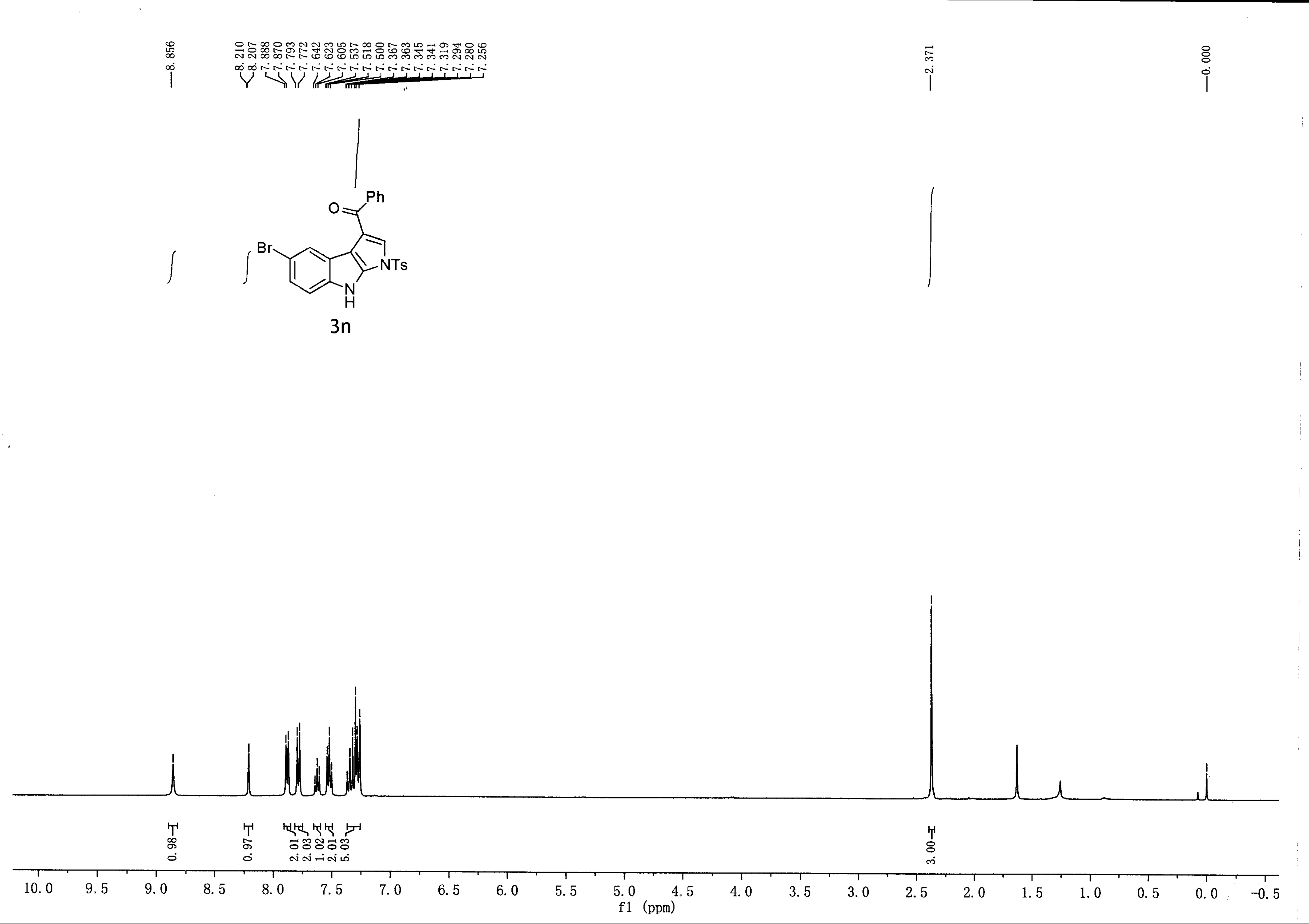

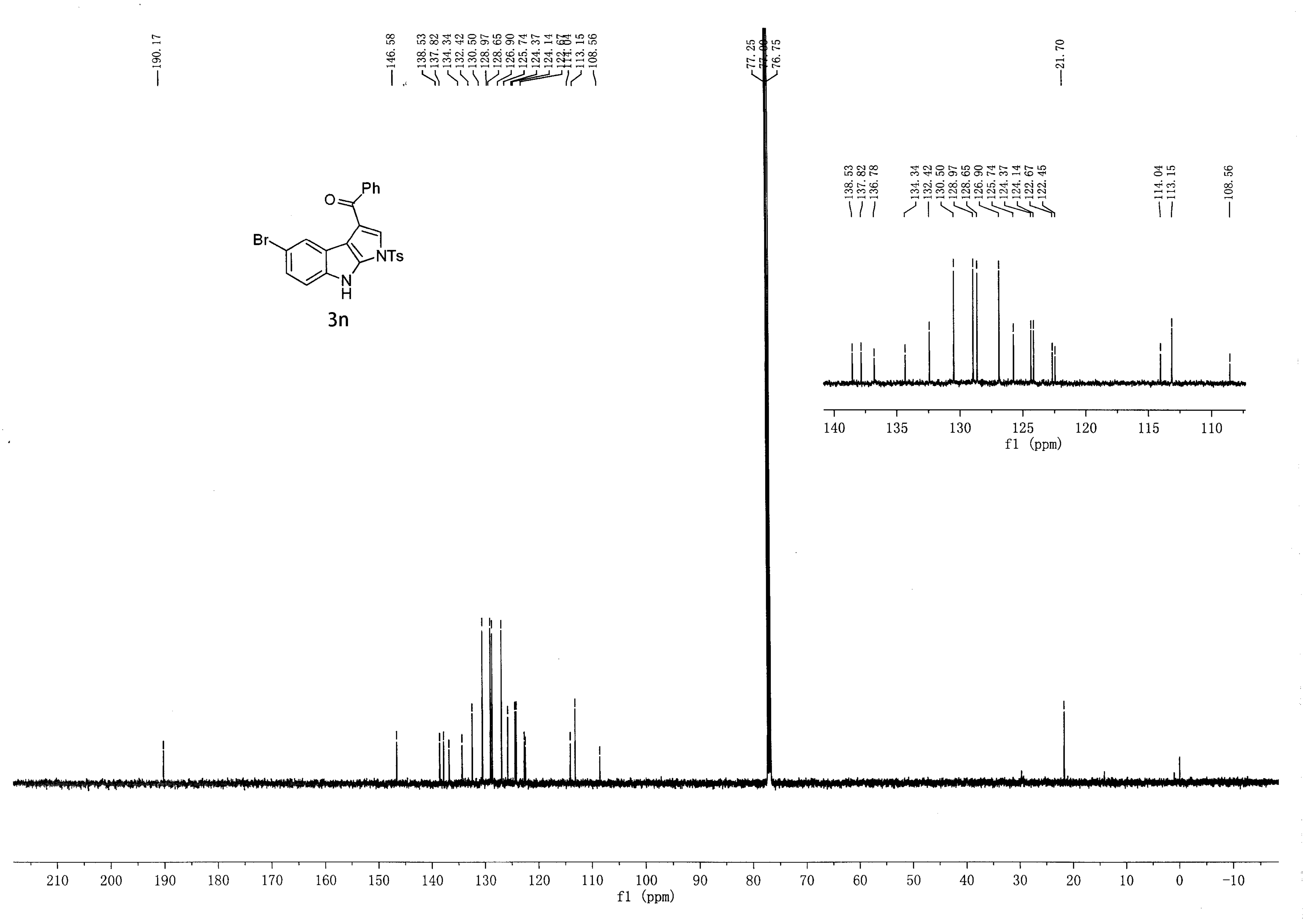

Supplementary Figure 55. ^{1}H and ^{13}C NMR spectra for 3n

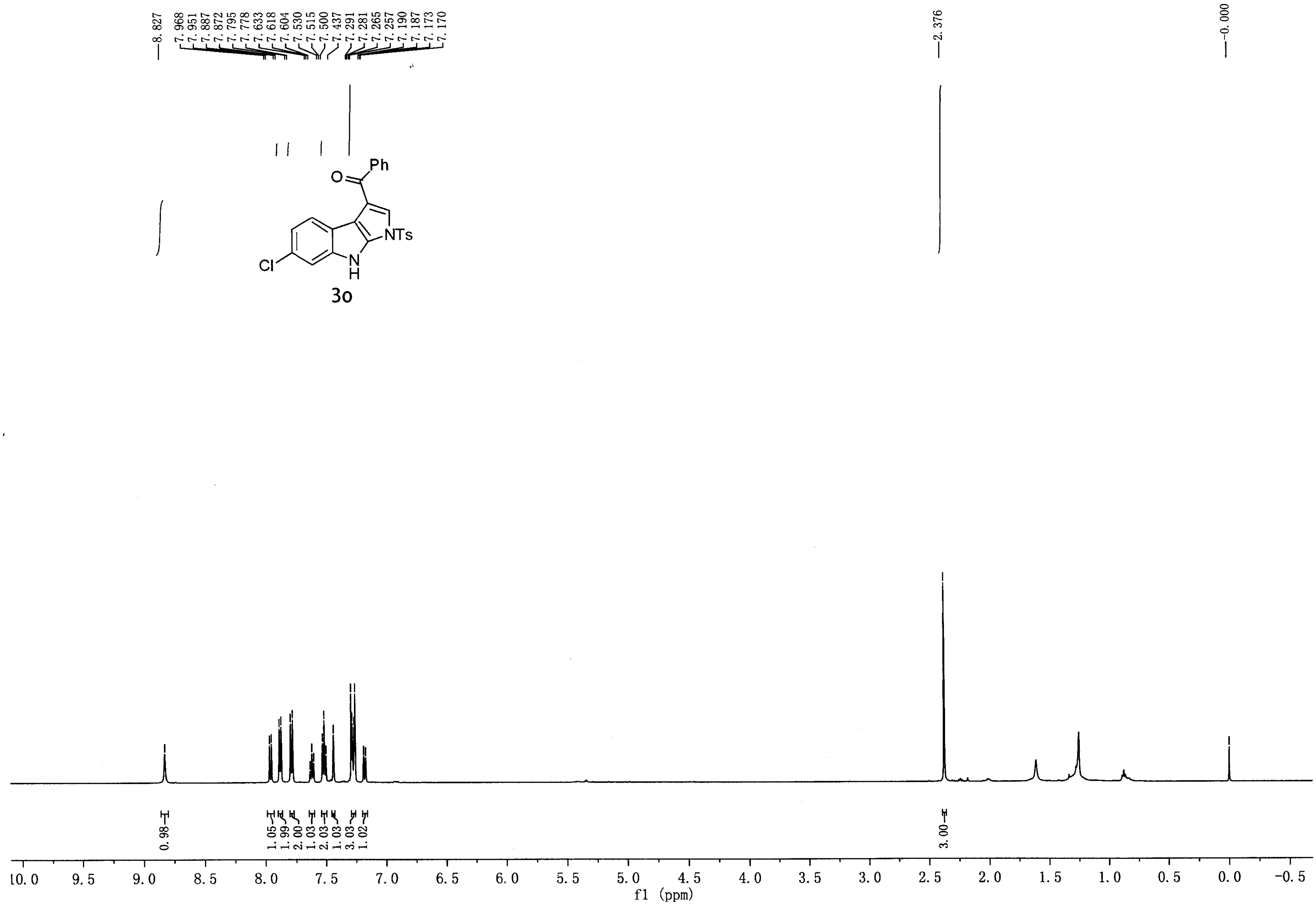

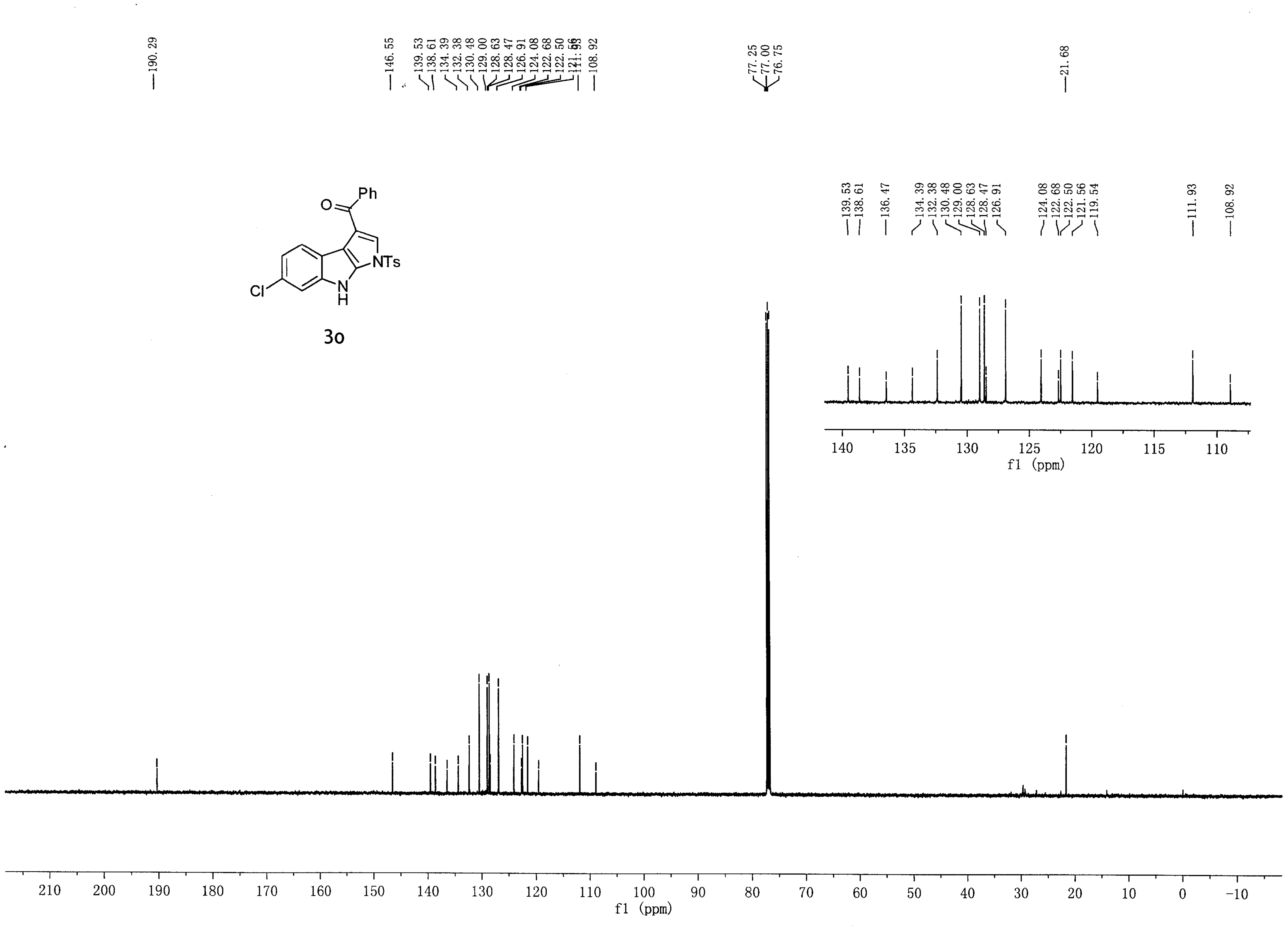

Supplementary Figure 56. ^{1}H and ^{13}C NMR spectra for 3o

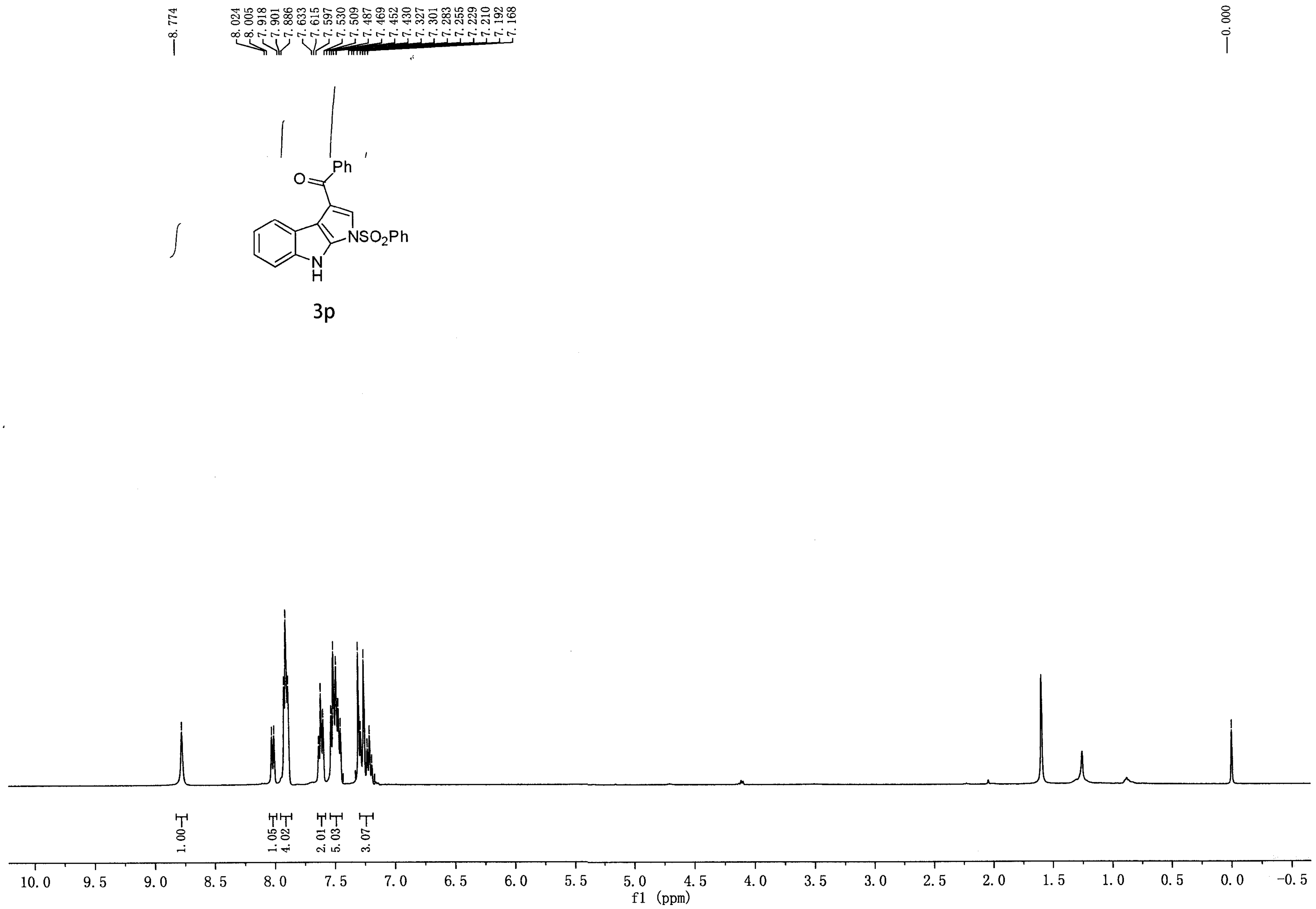

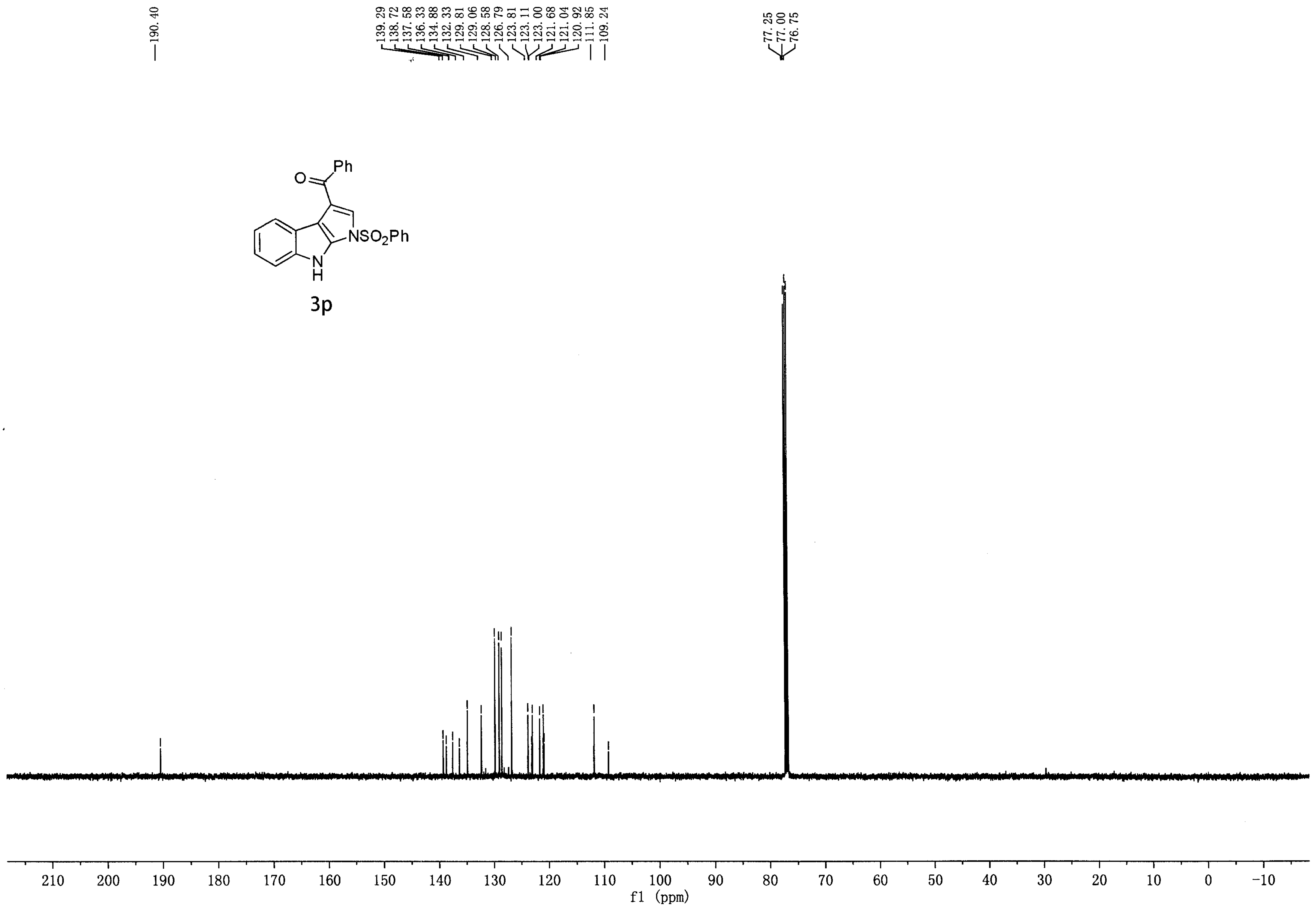

Supplementary Figure 57. ^{1}H and ^{13}C NMR spectra for 3p

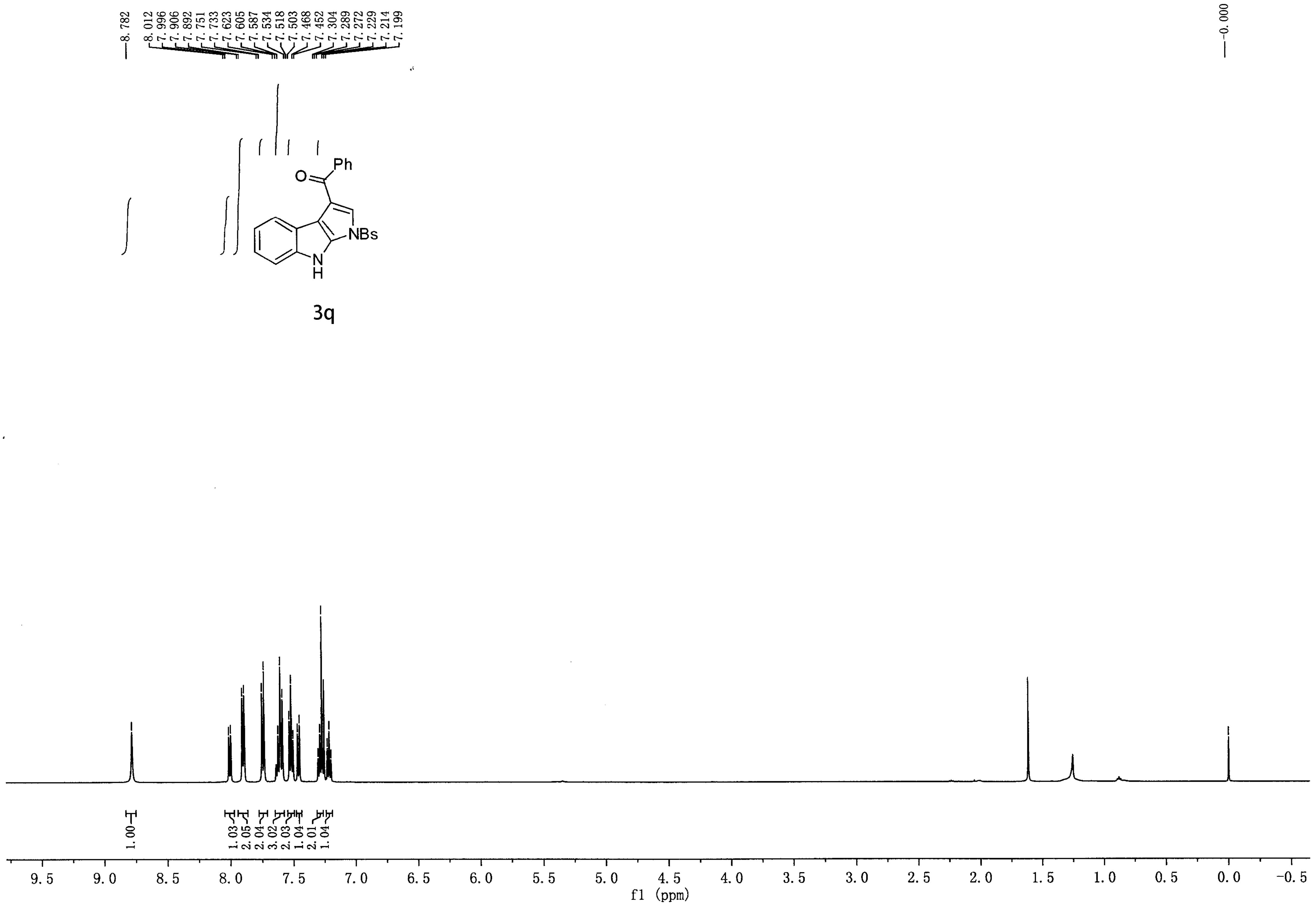

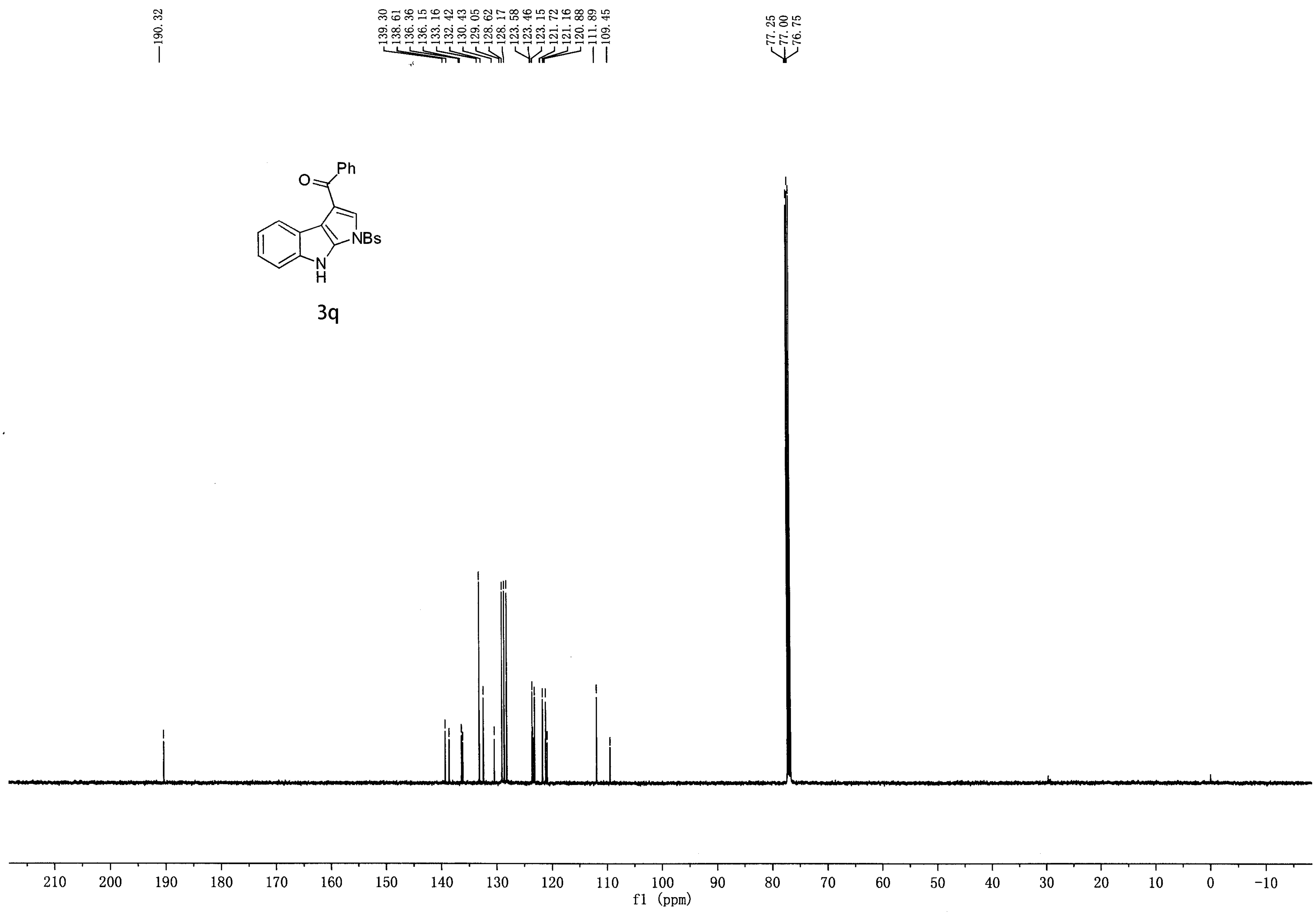

Supplementary Figure 58. ^1H and ^{13}C NMR spectra for 3q

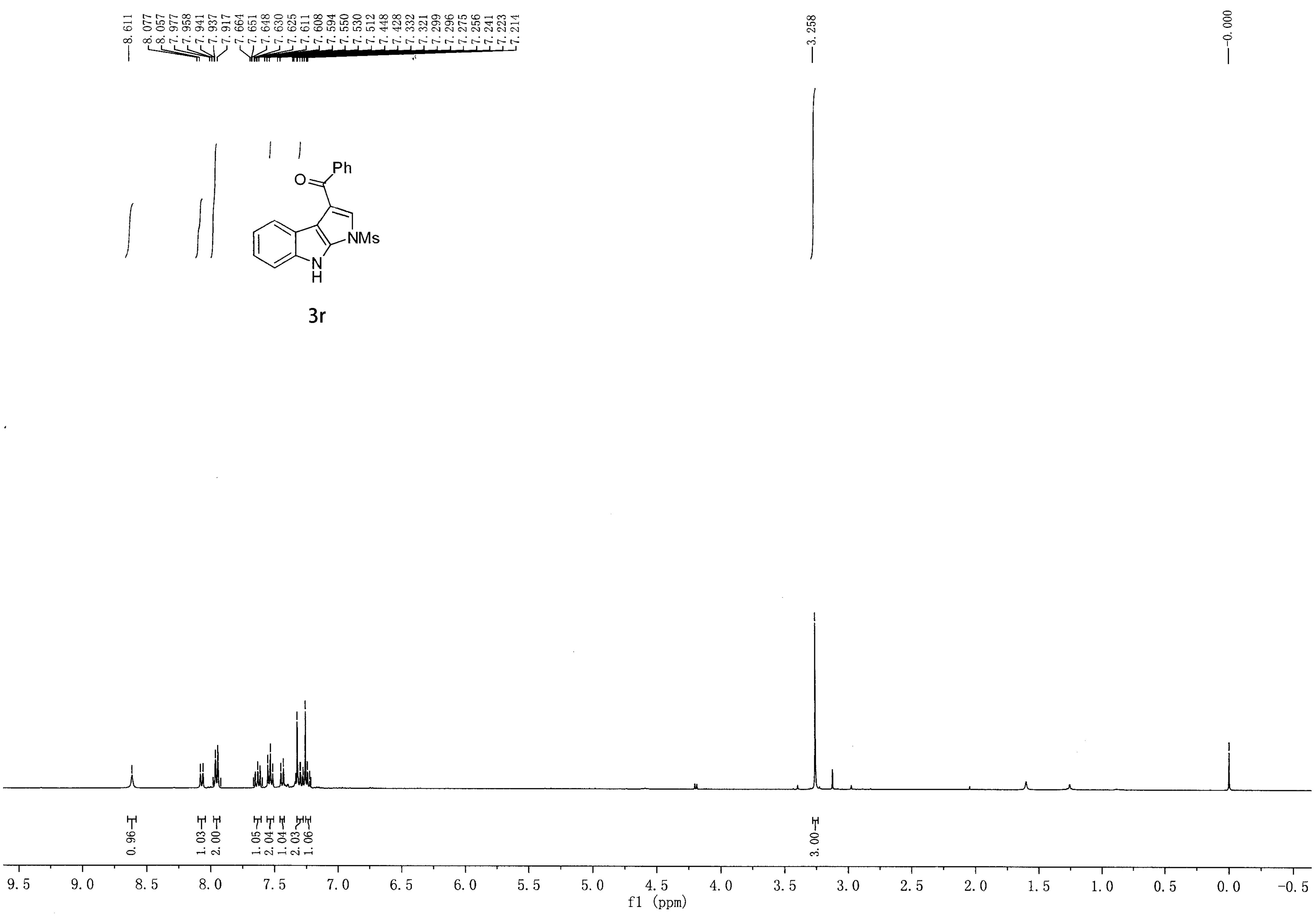

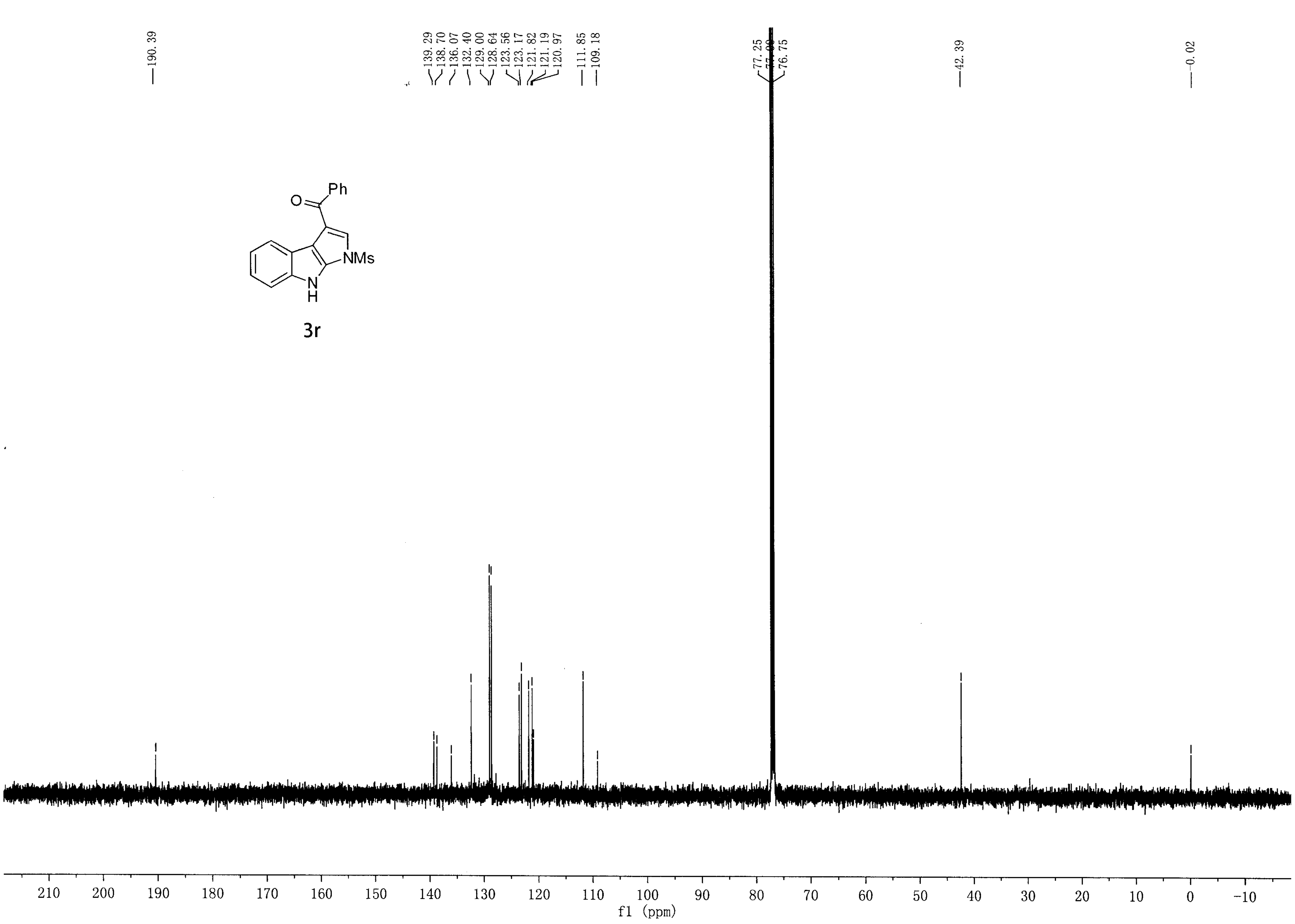

Supplementary Figure 59. ^{1}H and ^{13}C NMR spectra for 3r

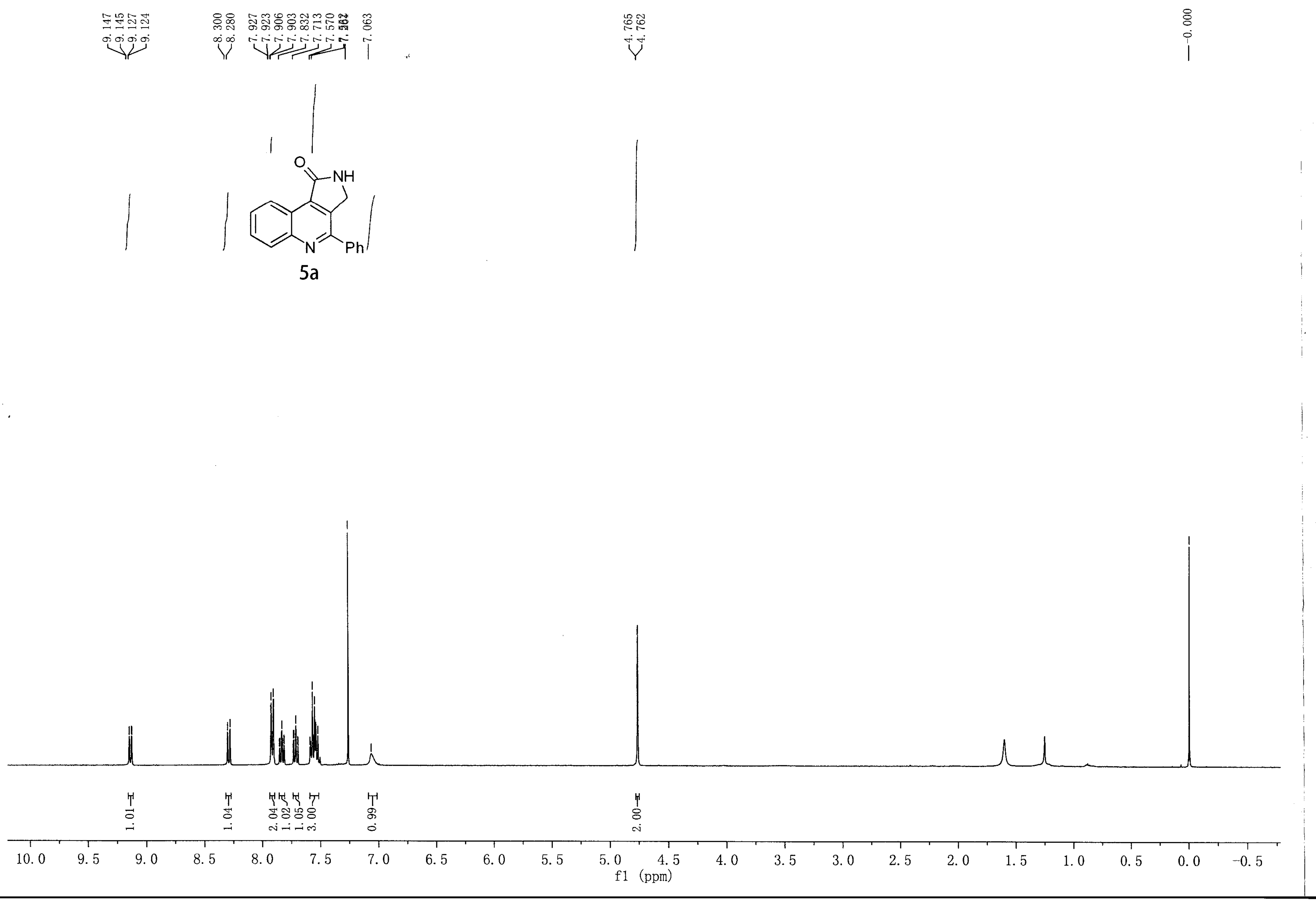

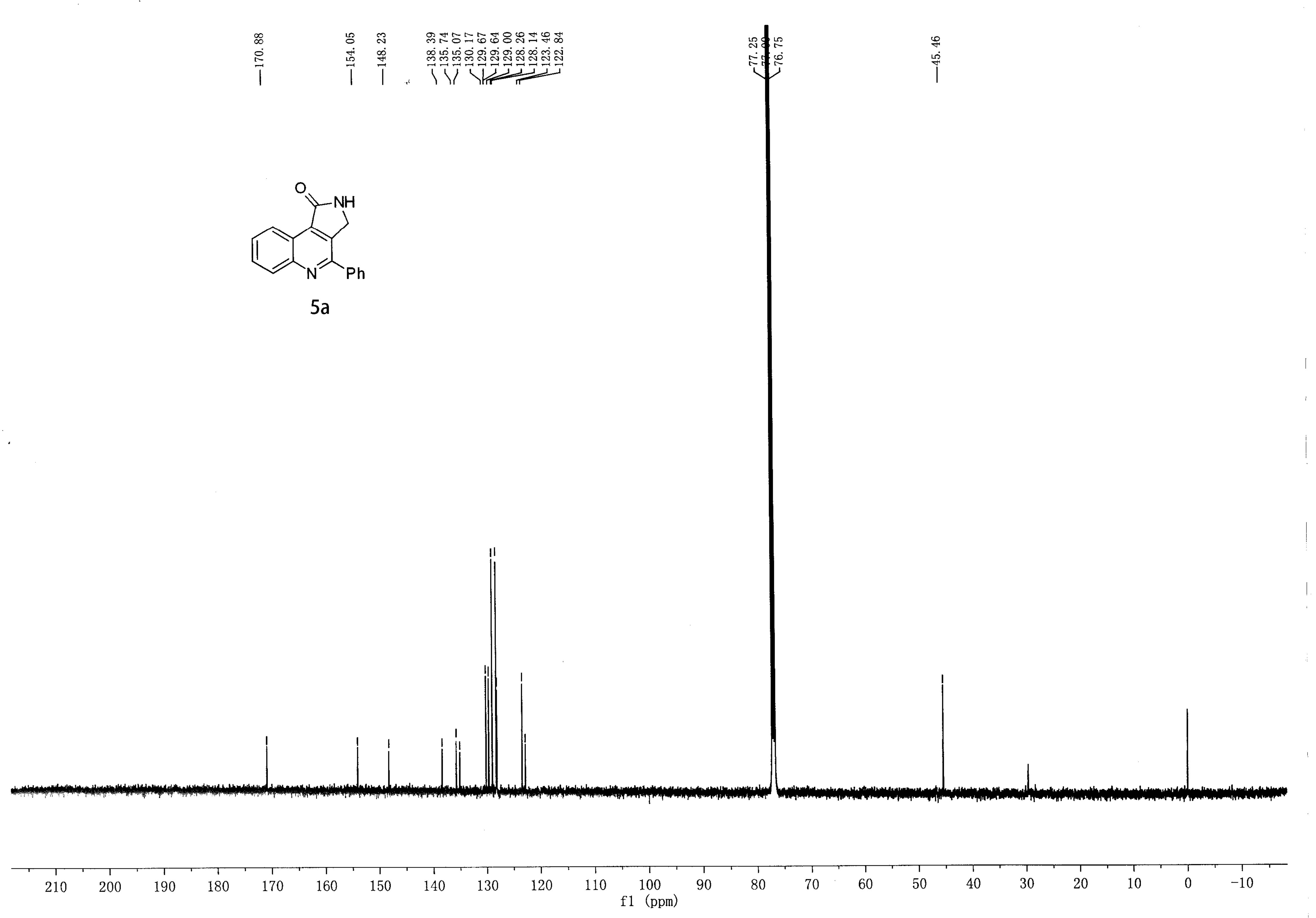

Supplementary Figure 60. ^{1}H and ^{13}C NMR spectra for 5a

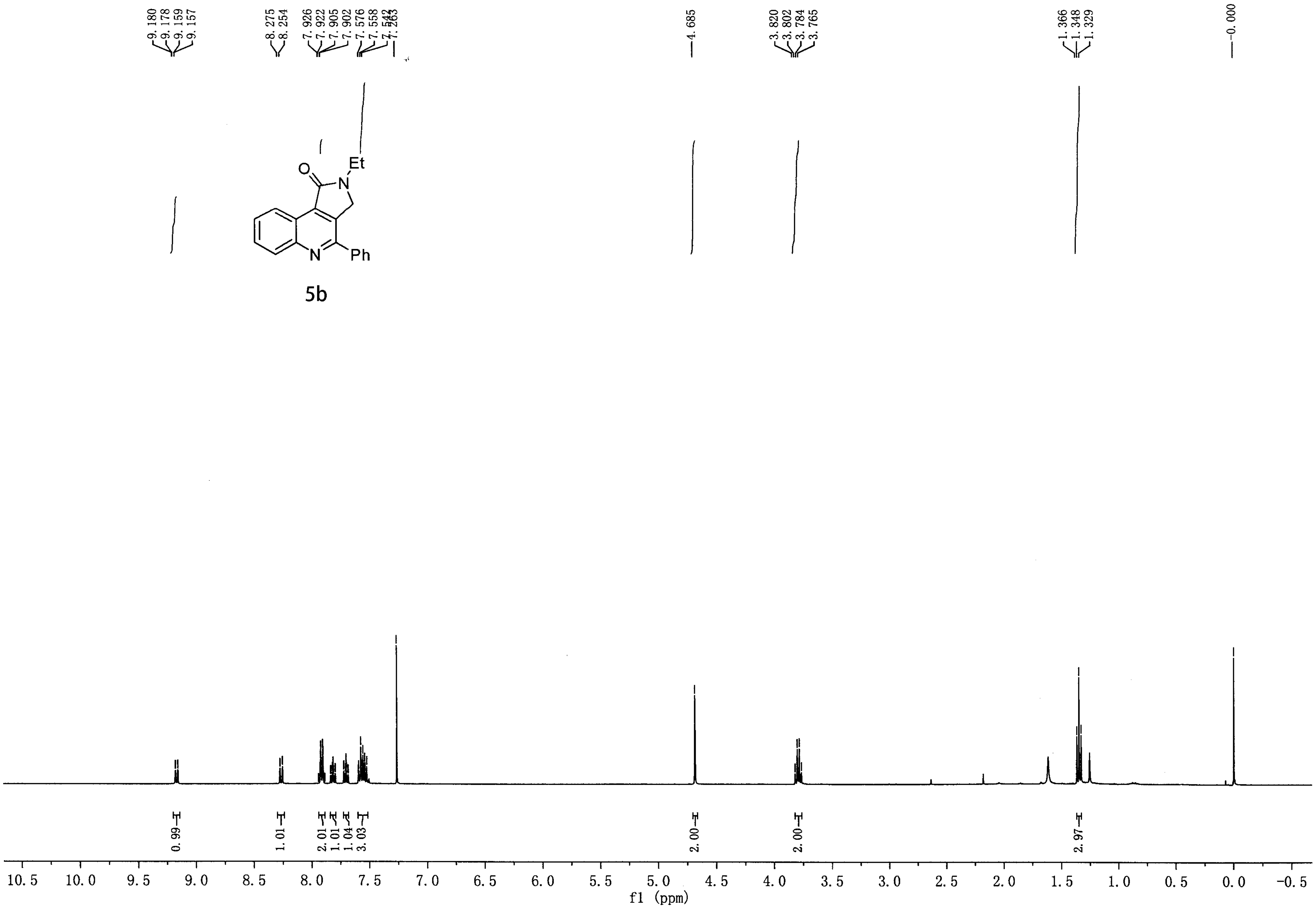

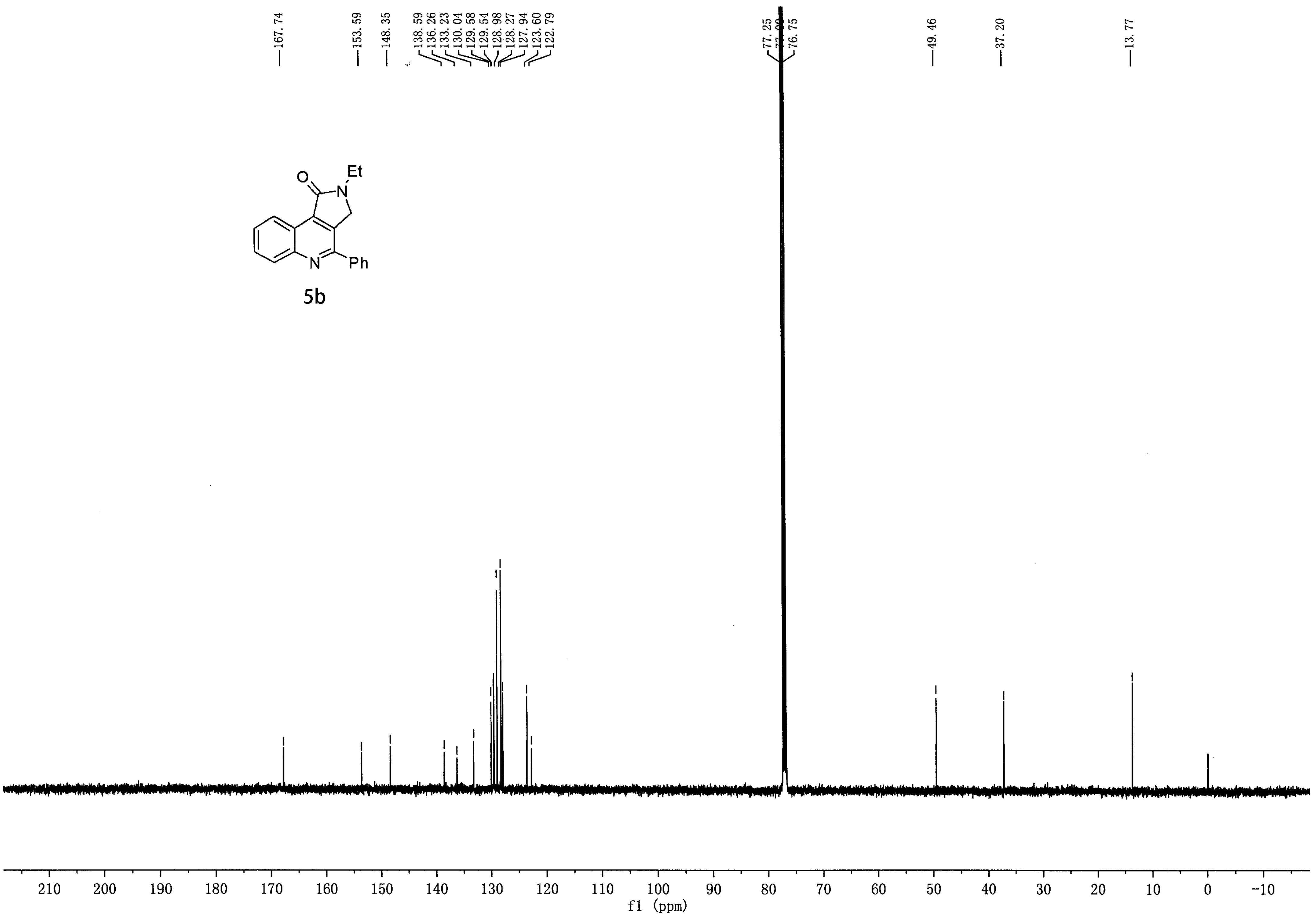

Supplementary Figure 61. ^{1}H and ^{13}C NMR spectra for 5b

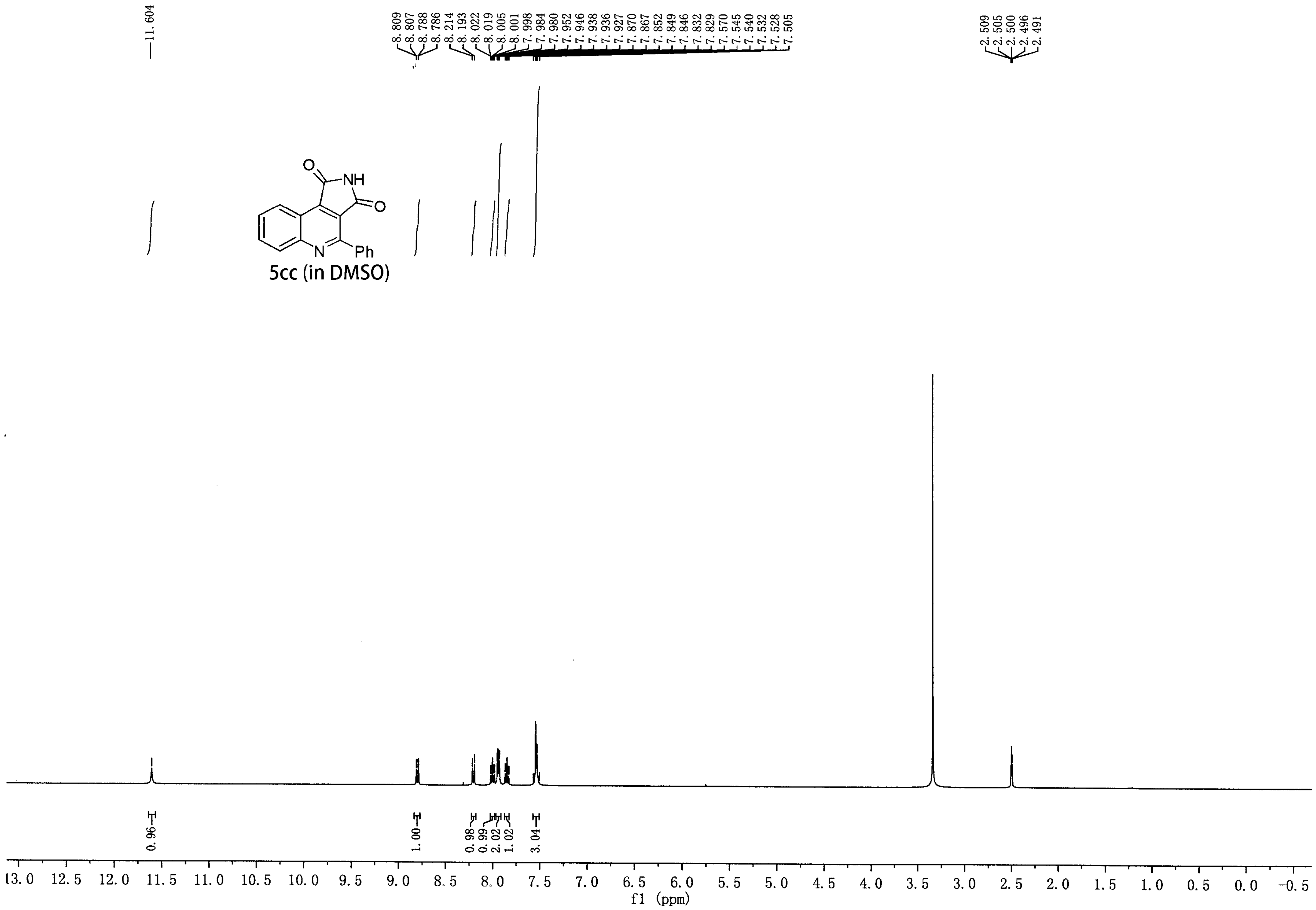

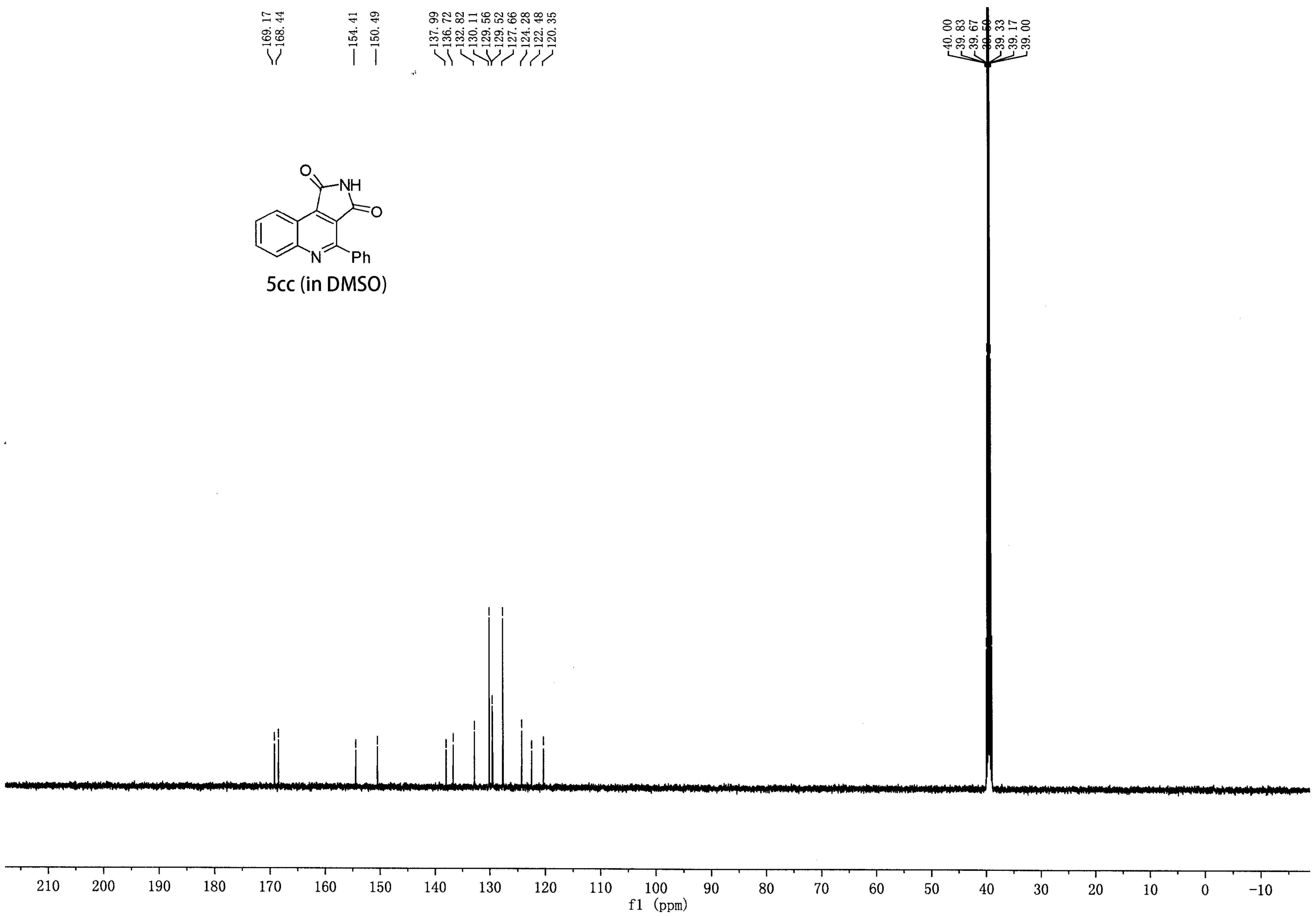

Supplementary Figure 62. ^{1}H and ^{13}C NMR spectra for 5cc

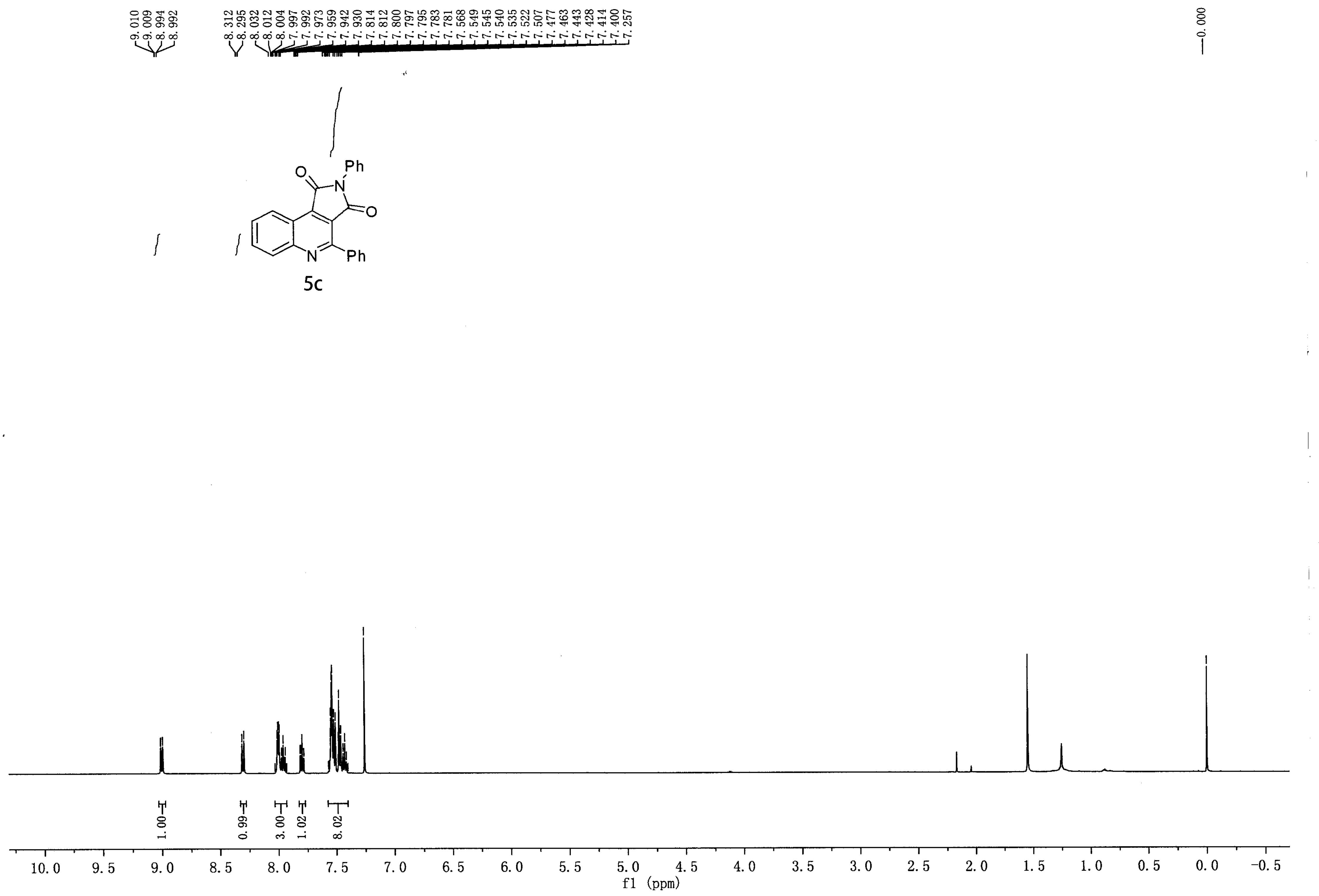

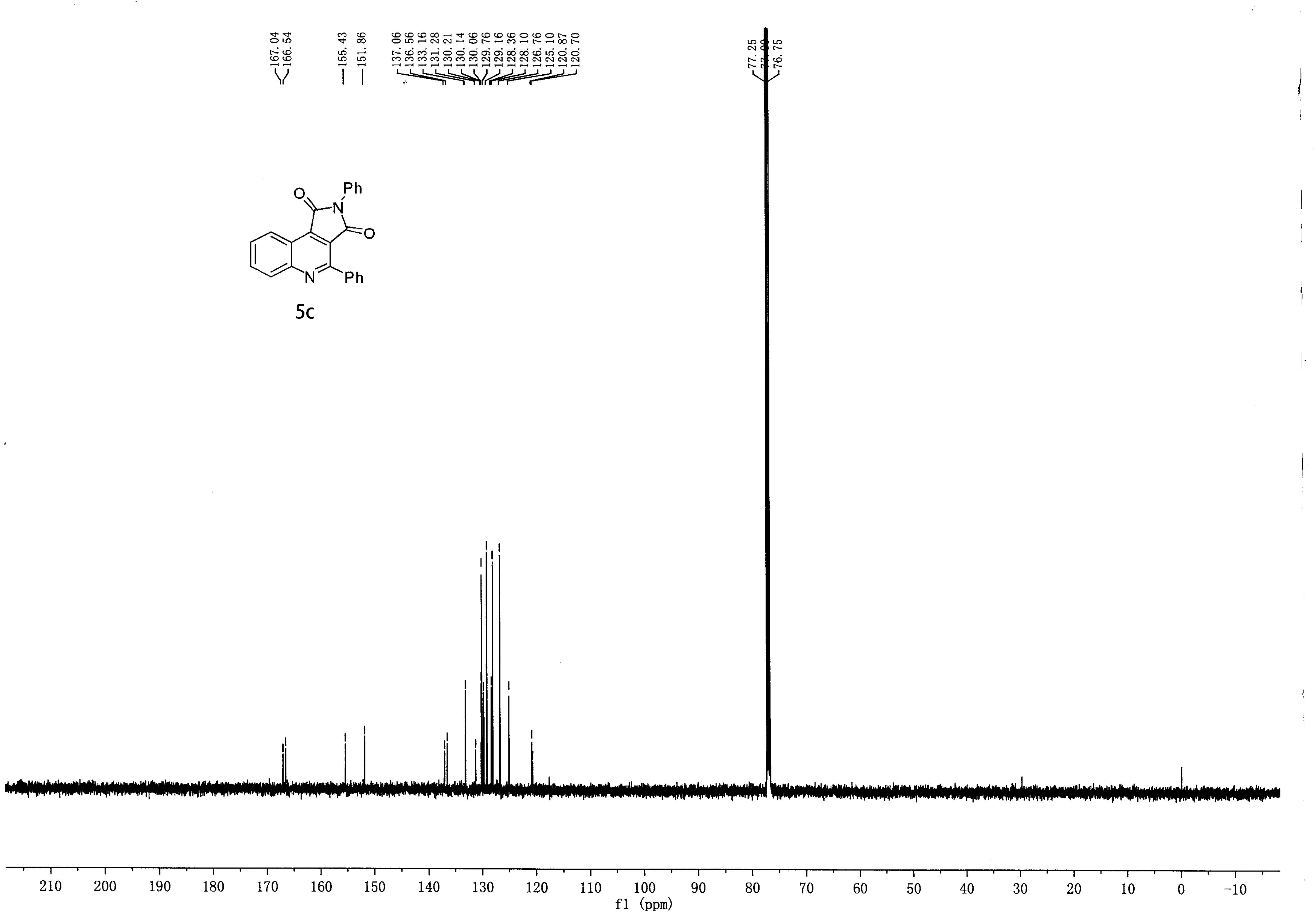

Supplementary Figure 63. ^{1}H and ^{13}C NMR spectra for 5c

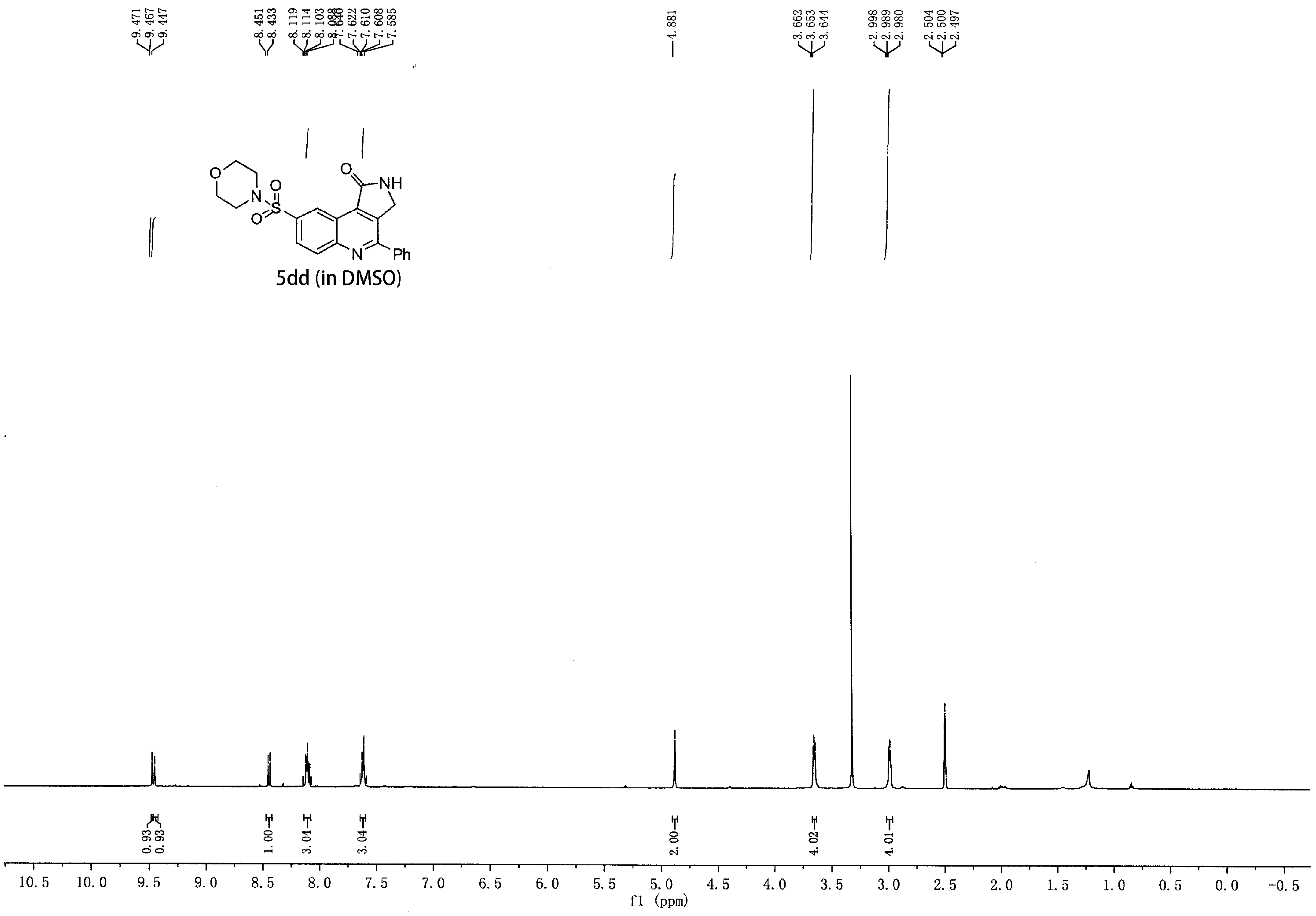

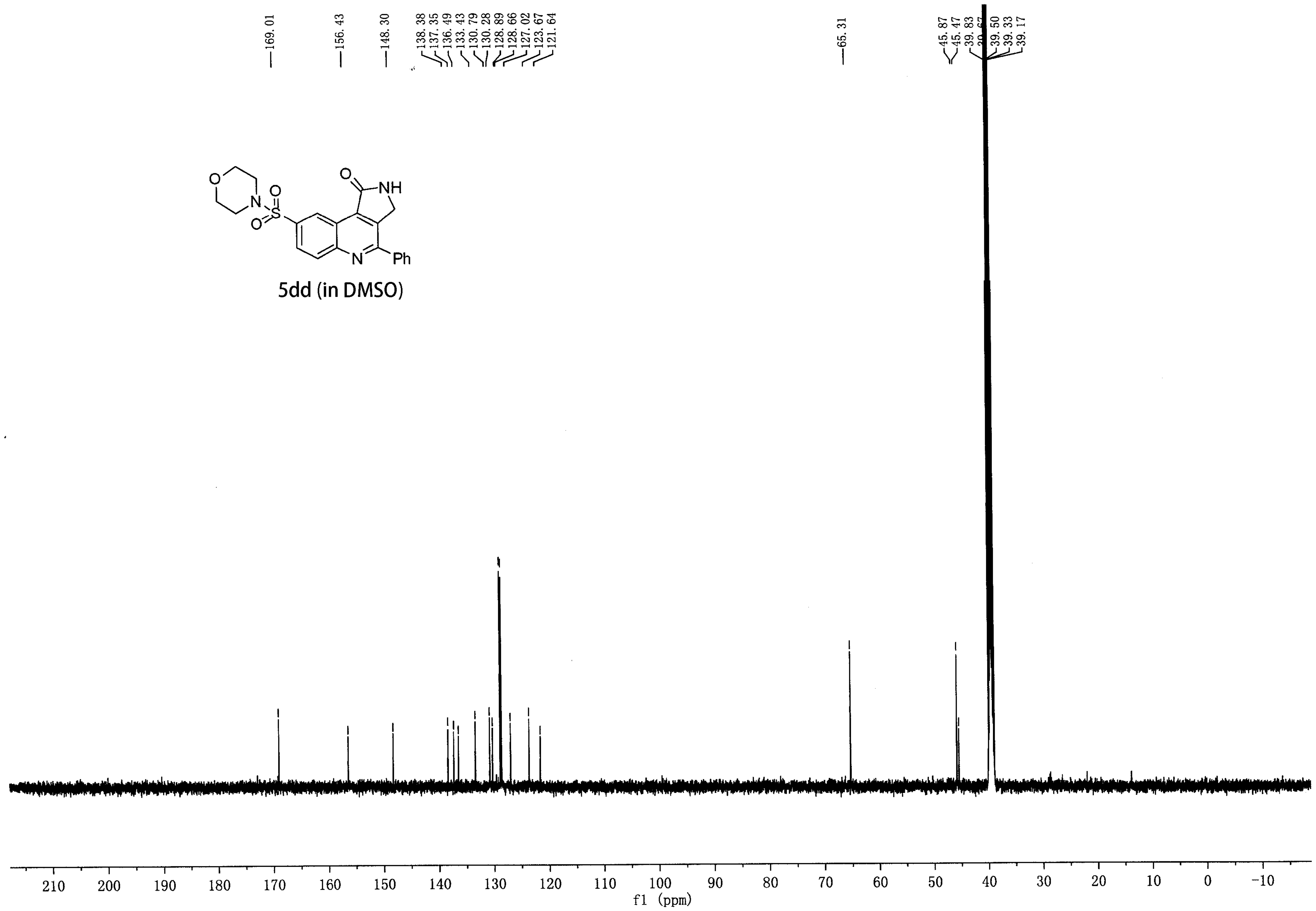

Supplementary Figure 64. ^{1}H and ^{13}C NMR spectra for 5dd

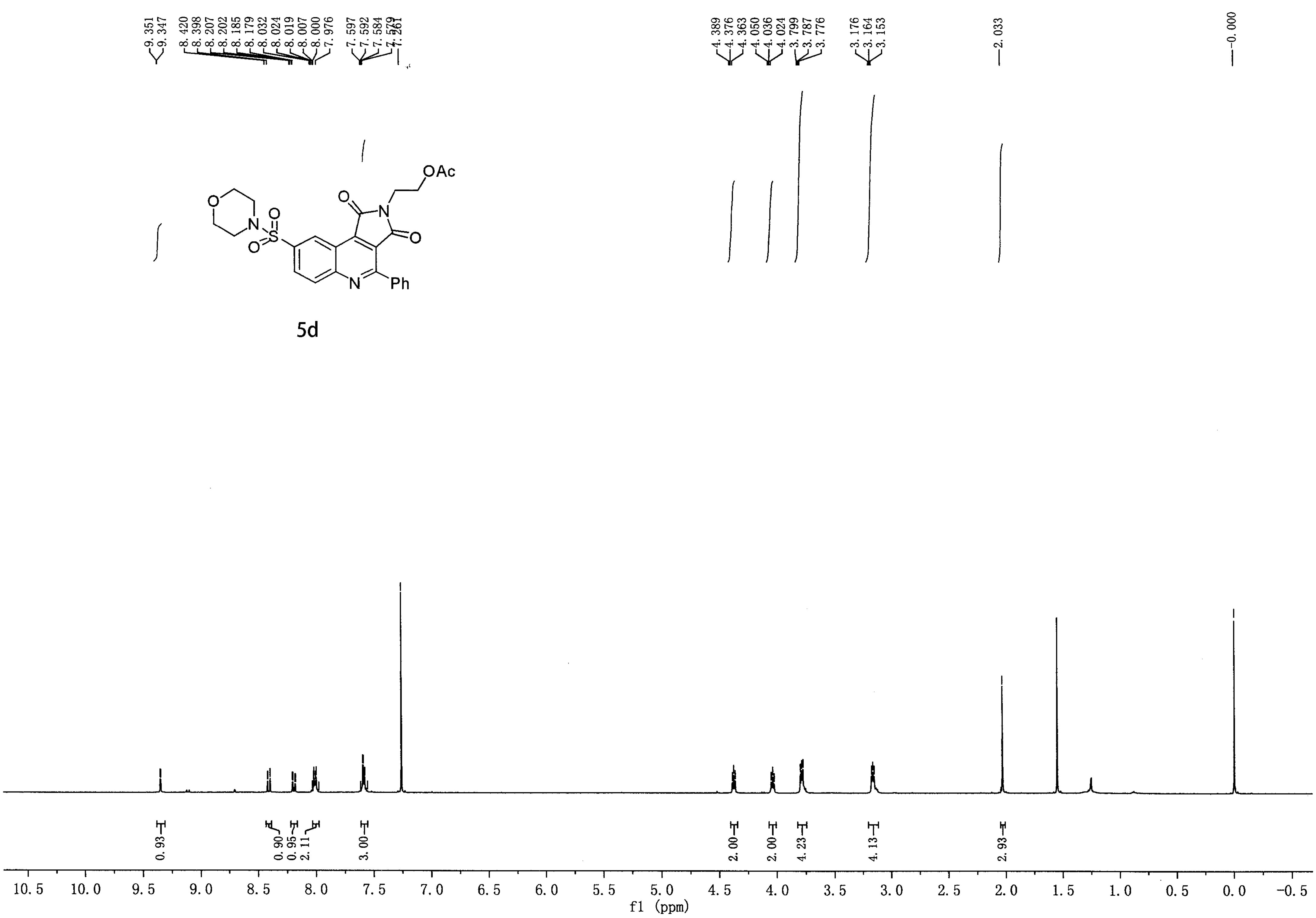

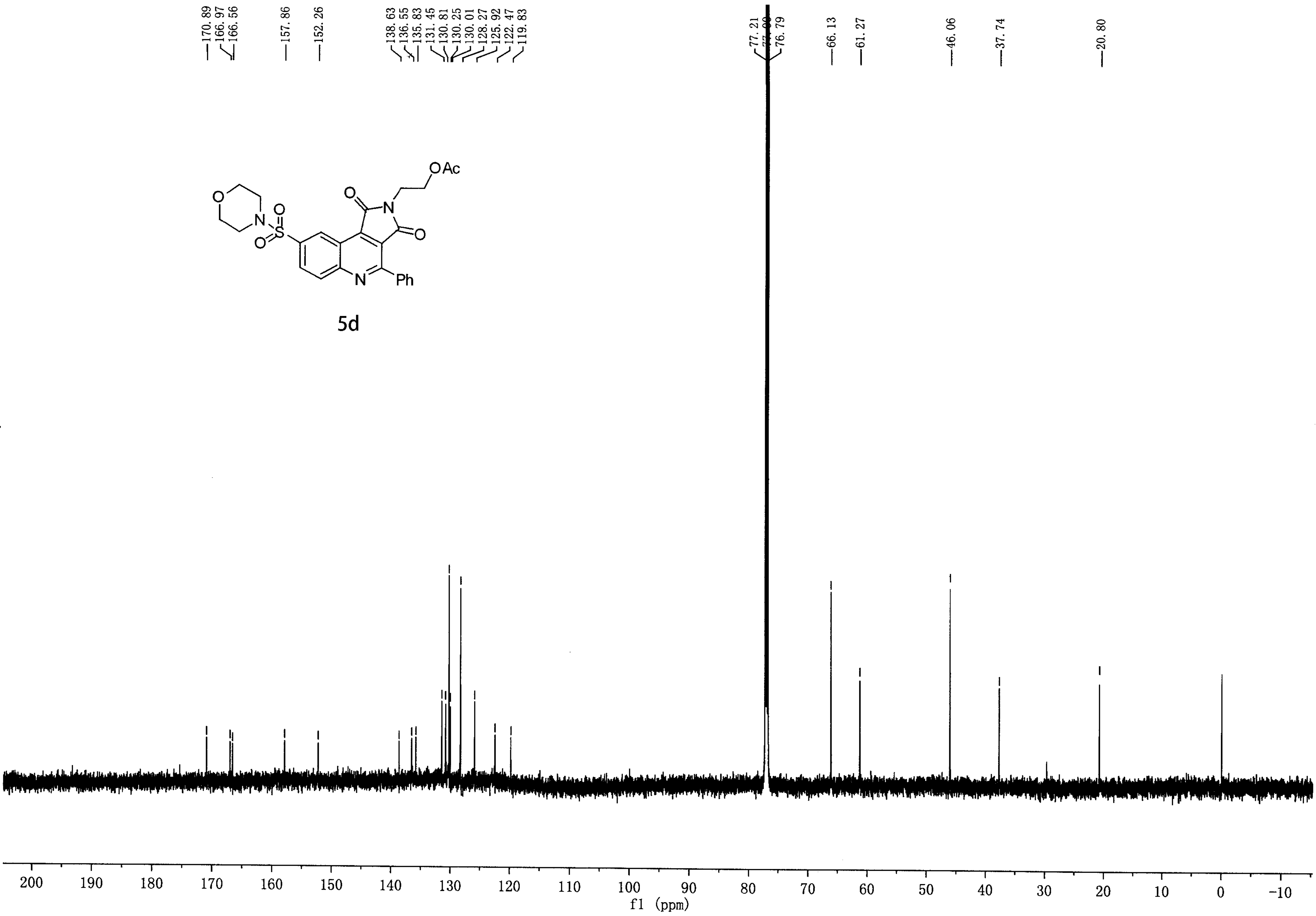

Supplementary Figure 65. ^{1}H and ^{13}C NMR spectra for 5d

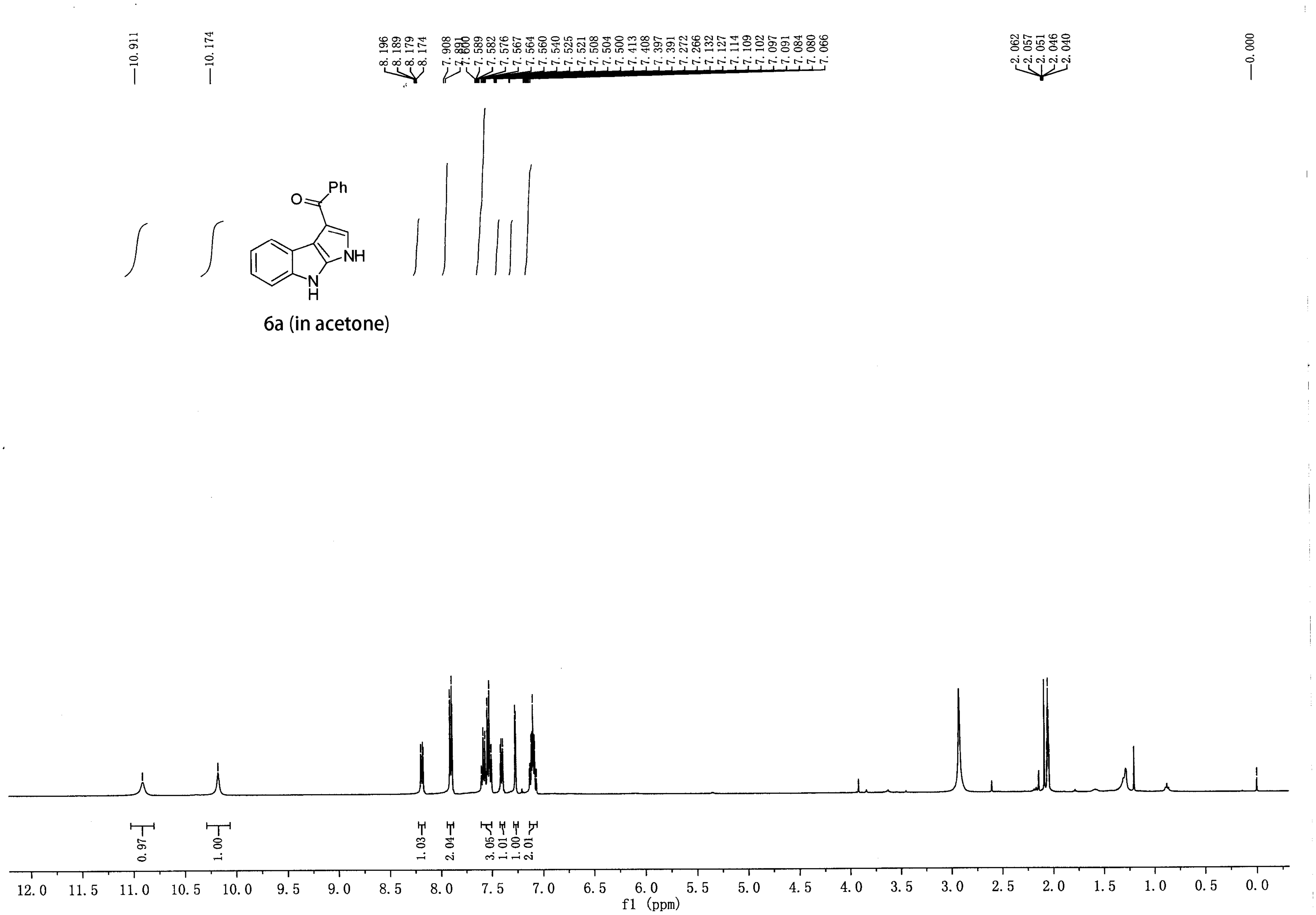

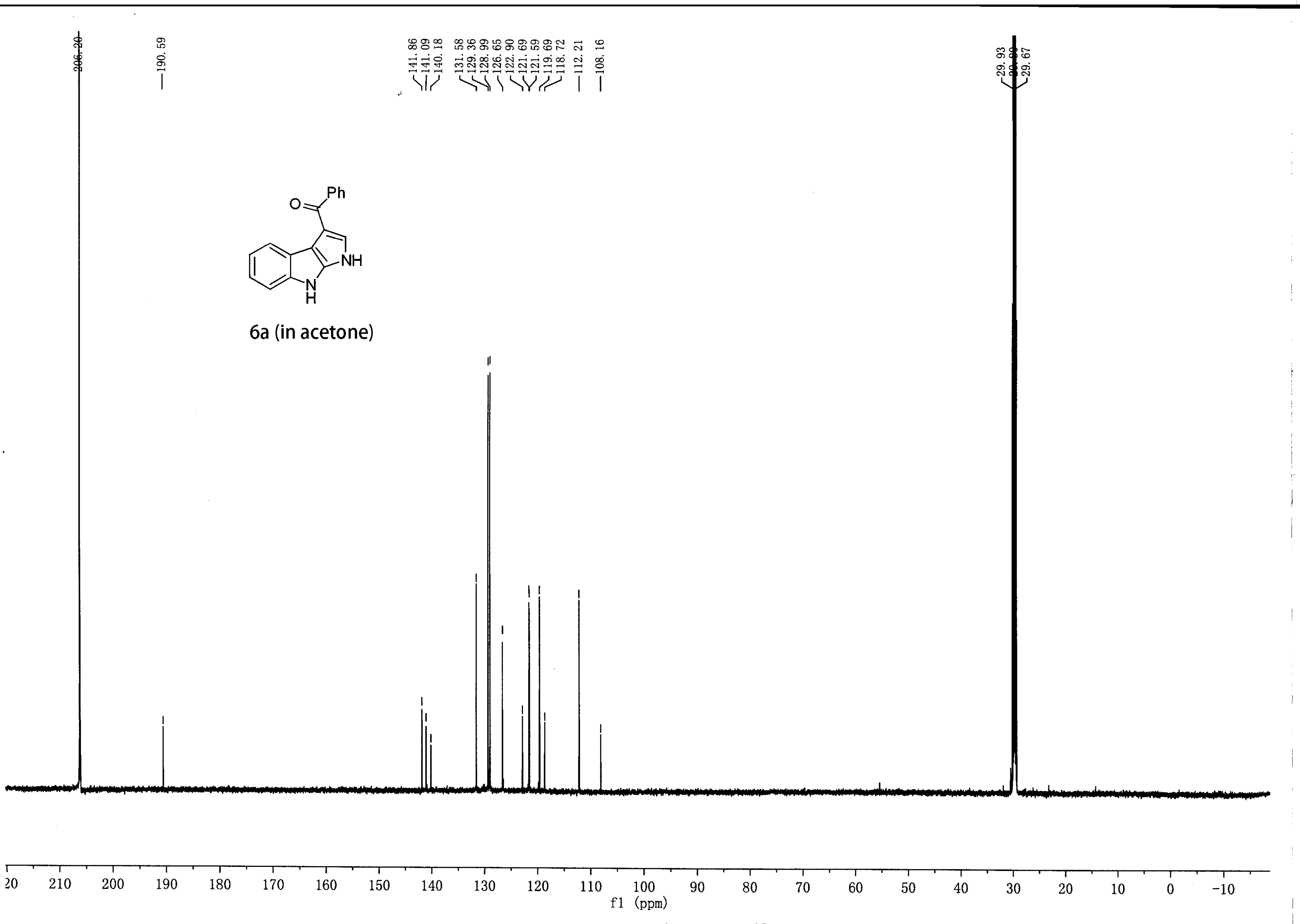

Supplementary Figure 66. ^{1}H and ^{13}C NMR spectra for 6a

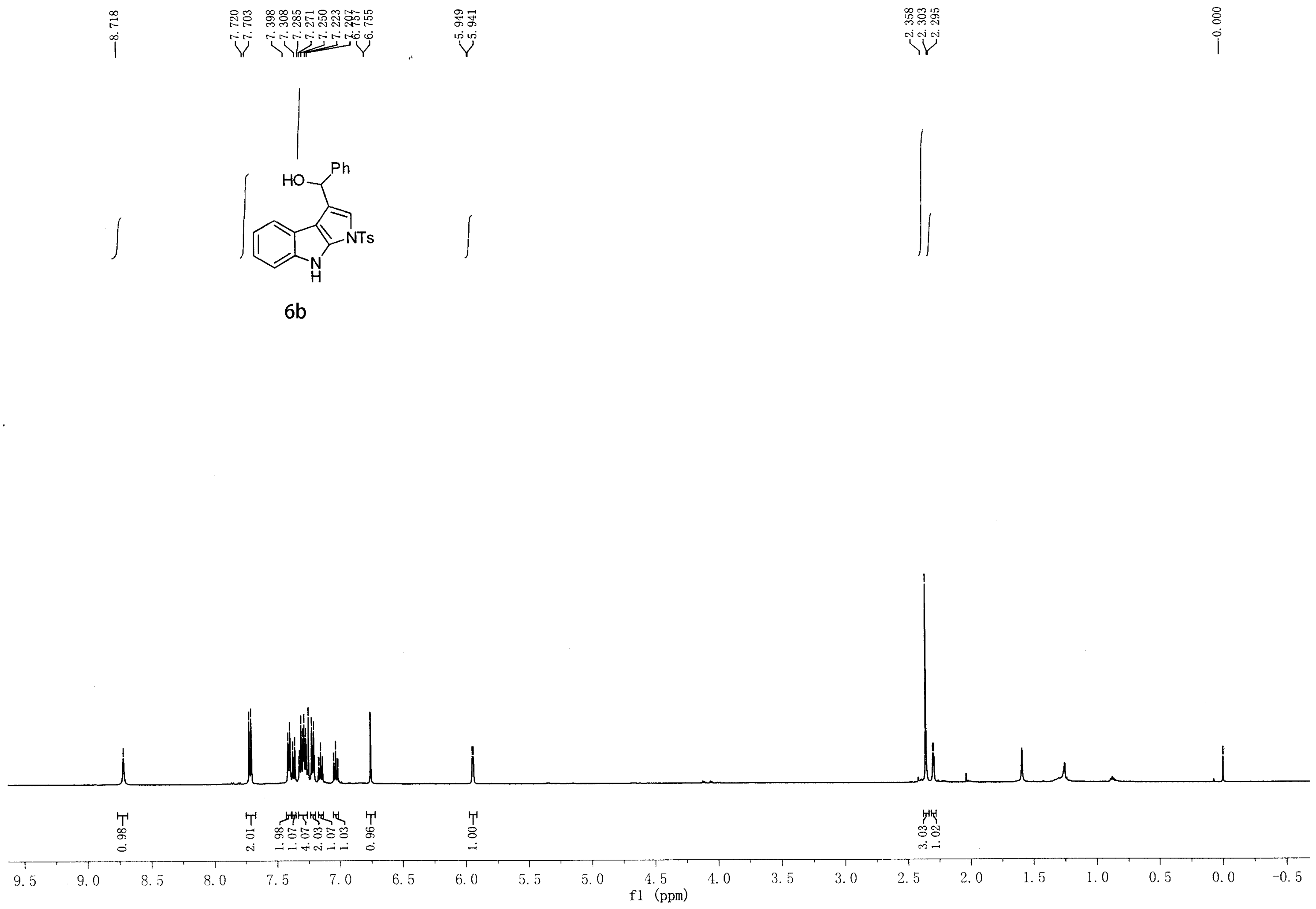

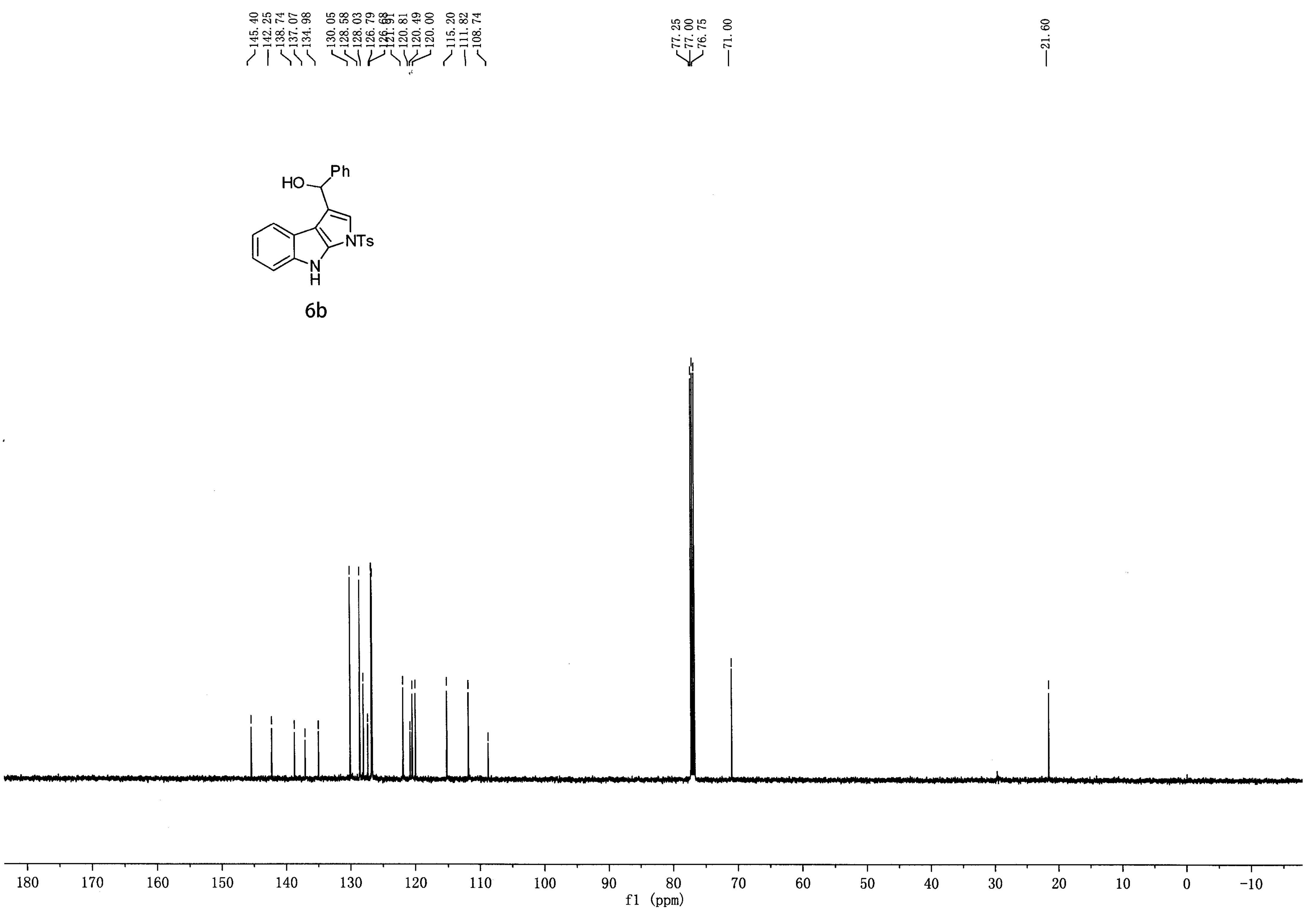

Supplementary Figure 67. ^{1}H and ^{13}C NMR spectra for 6b

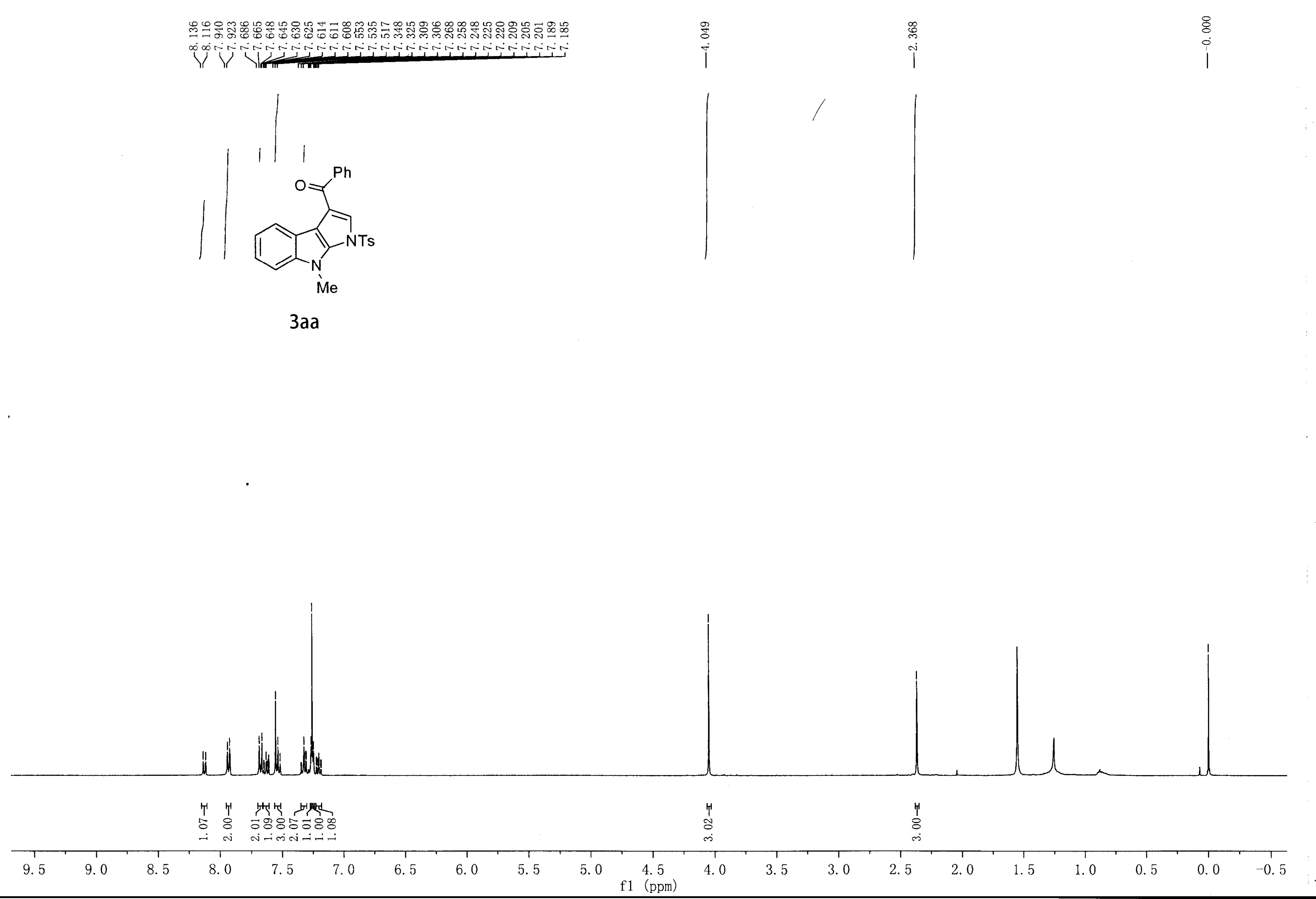

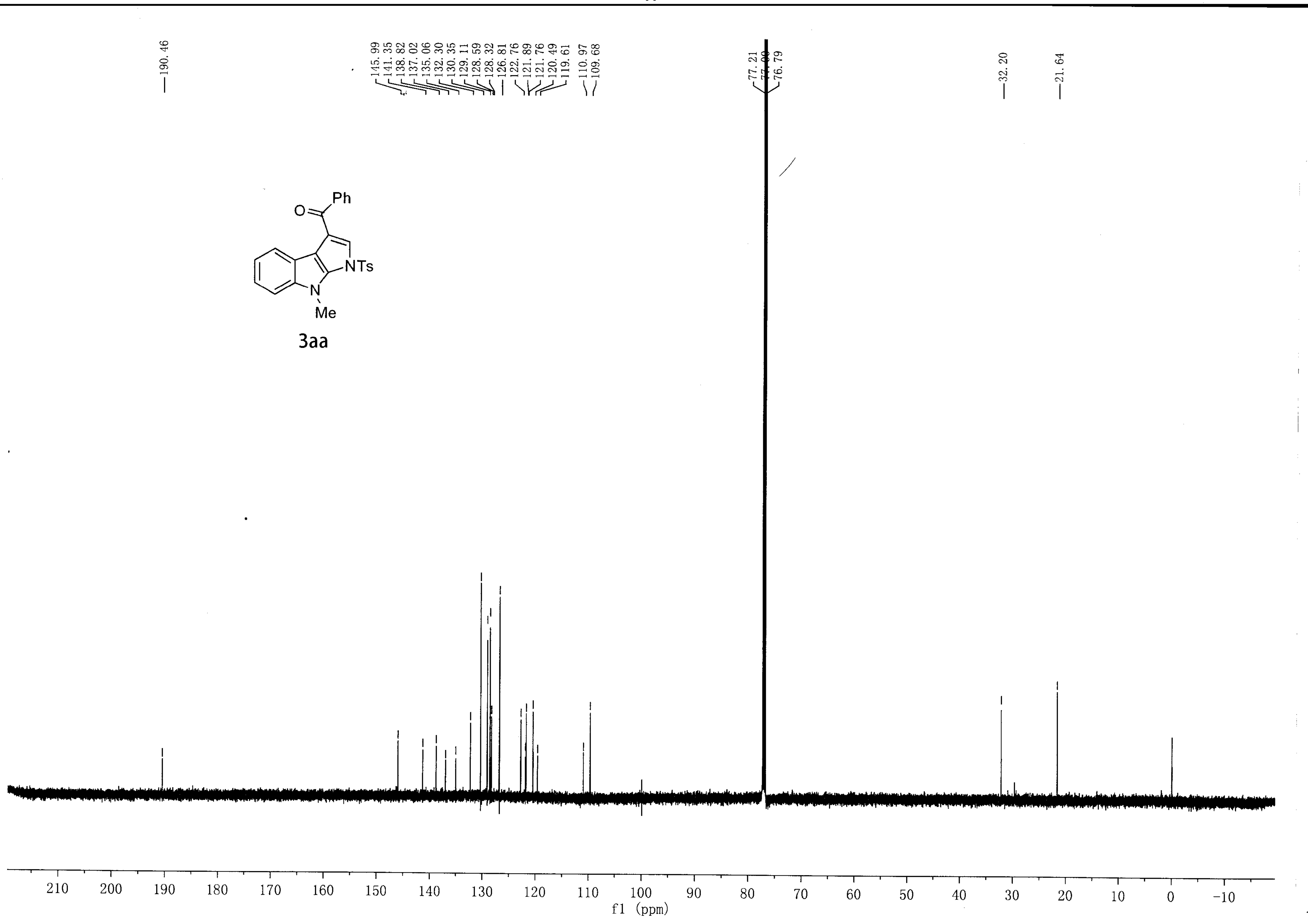

Supplementary Figure 68. ^1H and ^{13}C NMR spectra for 3aa

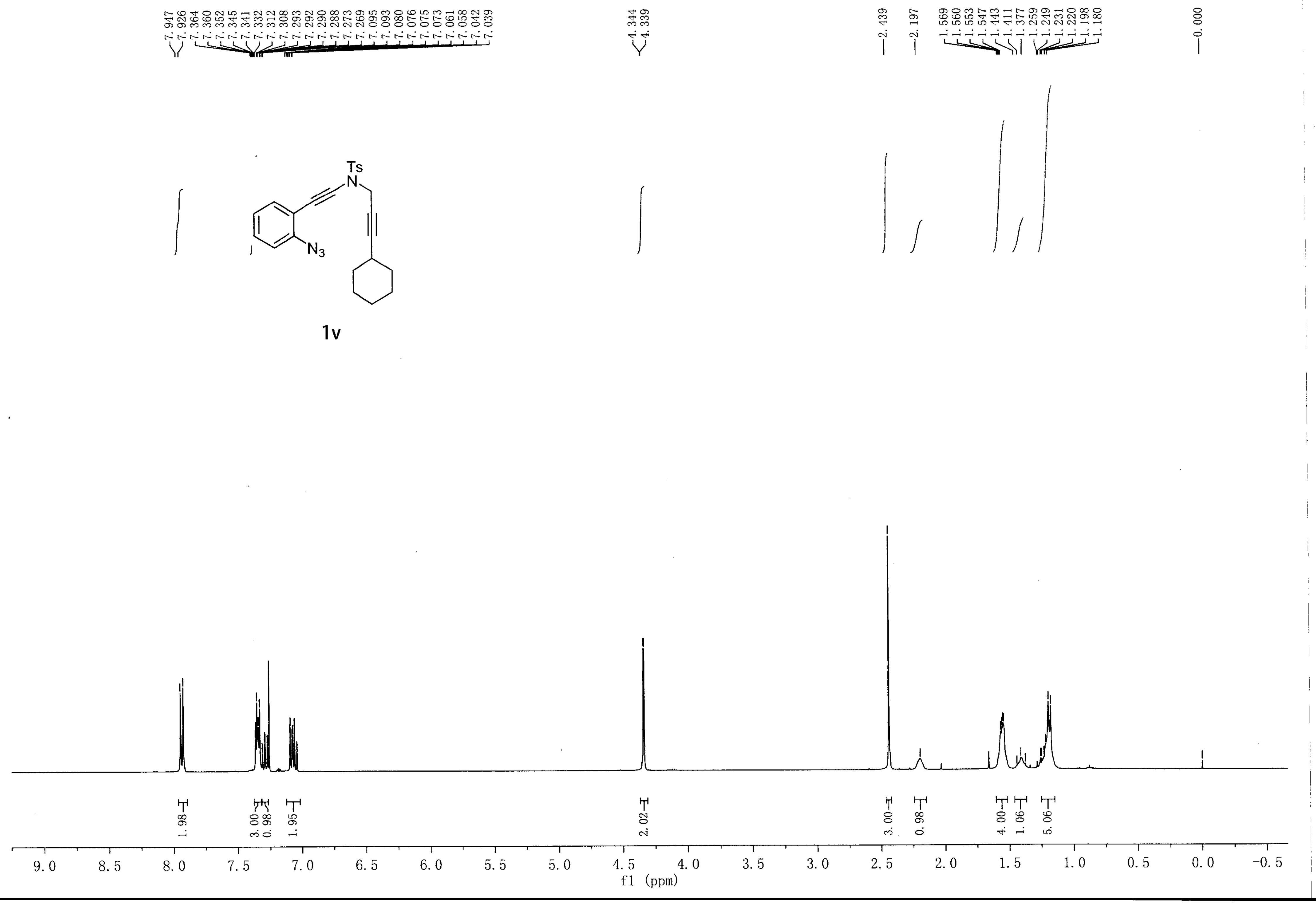

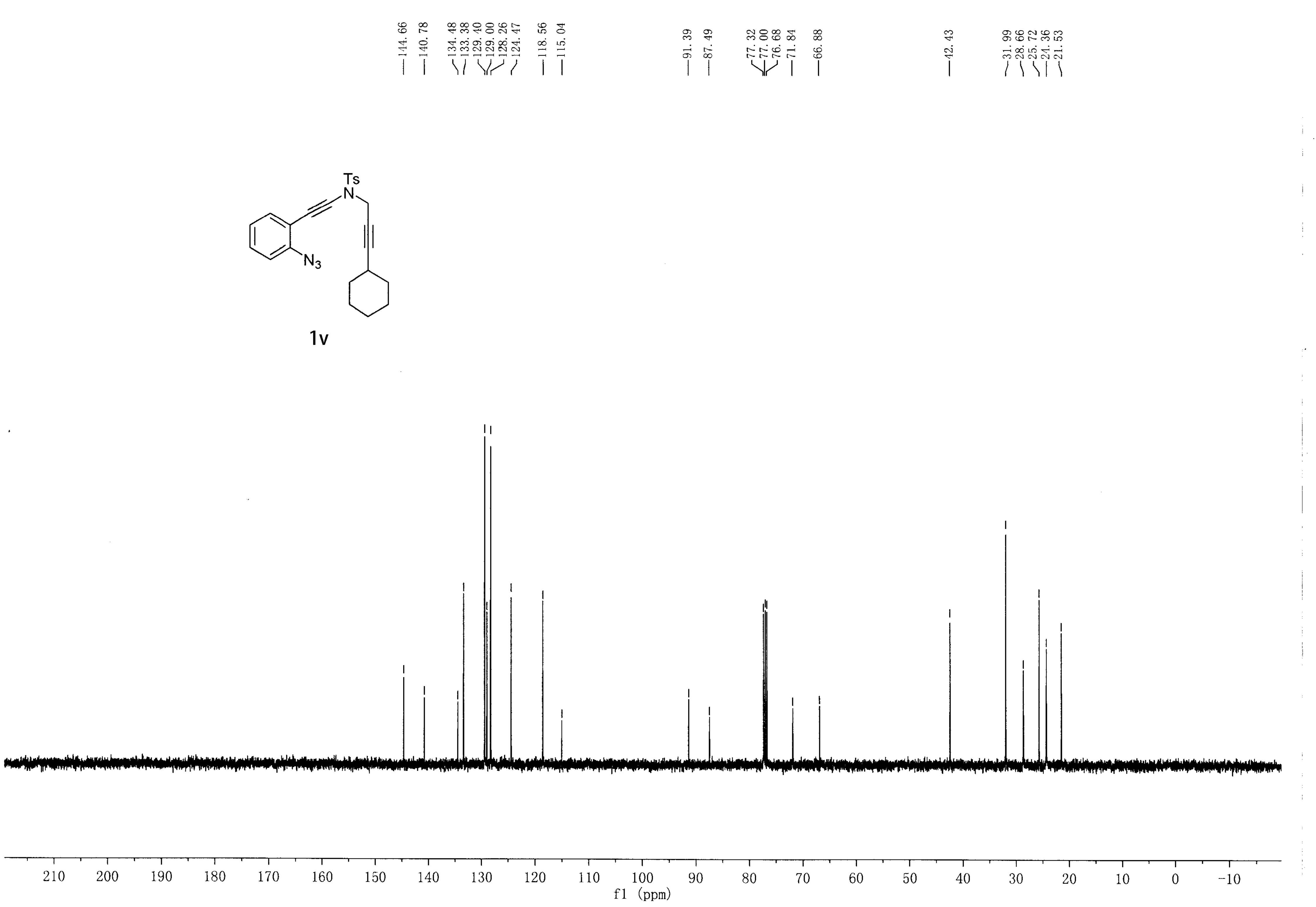

Supplementary Figure 69. ^{1}H and ^{13}C NMR spectra for 1v

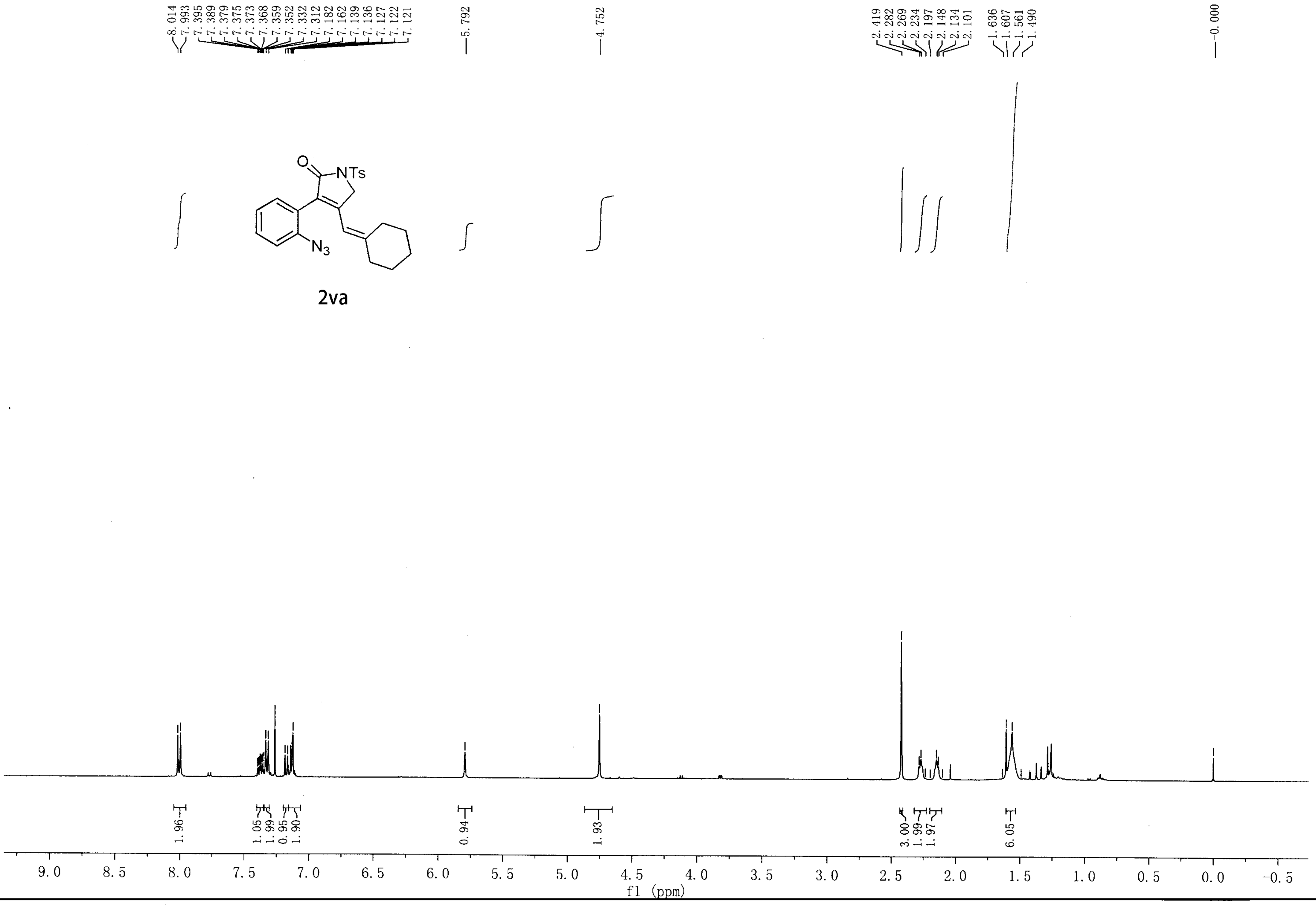

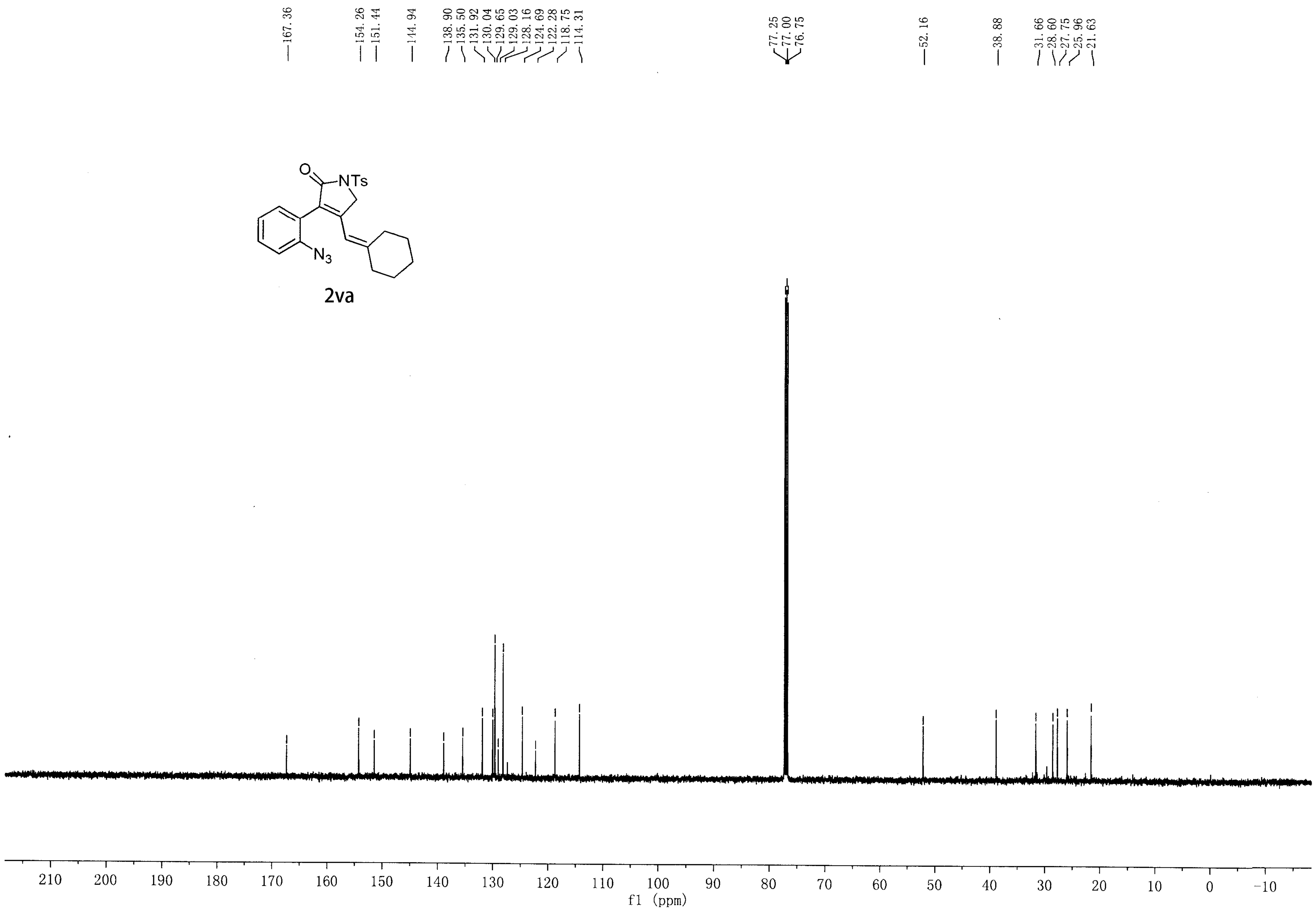

Supplementary Figure 70. ^{1}H and ^{13}C NMR spectra for 2va

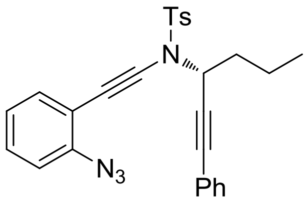

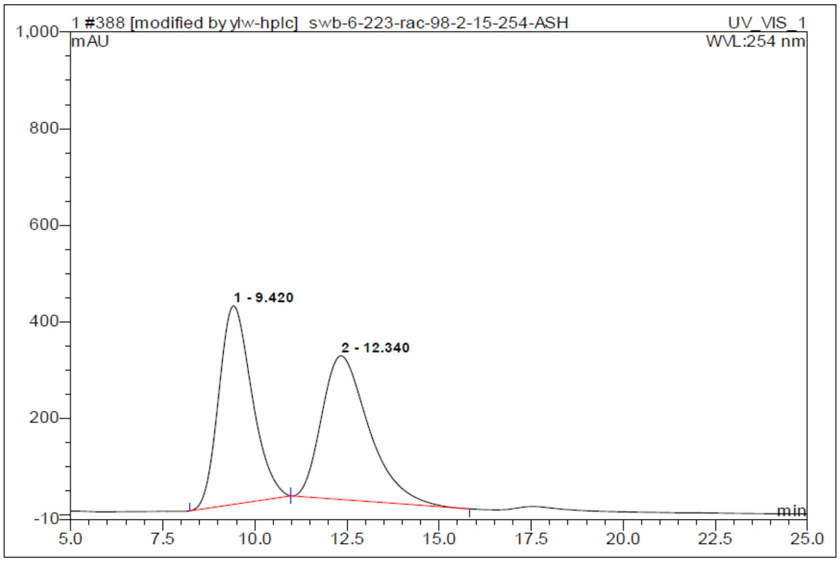

No.	Ret.Time min	Peak Name	Height mAU	Area mAU*min	Rel.Area %	Amount n.a.	Type
1	9.42	n.a.	411.085	429.936	49.25	n.a.	BMb*
2	12.34	n.a.	297.527	442.974	50.75	n.a.	bMB*
Total:			708.612	872.910	100.00	0.000	

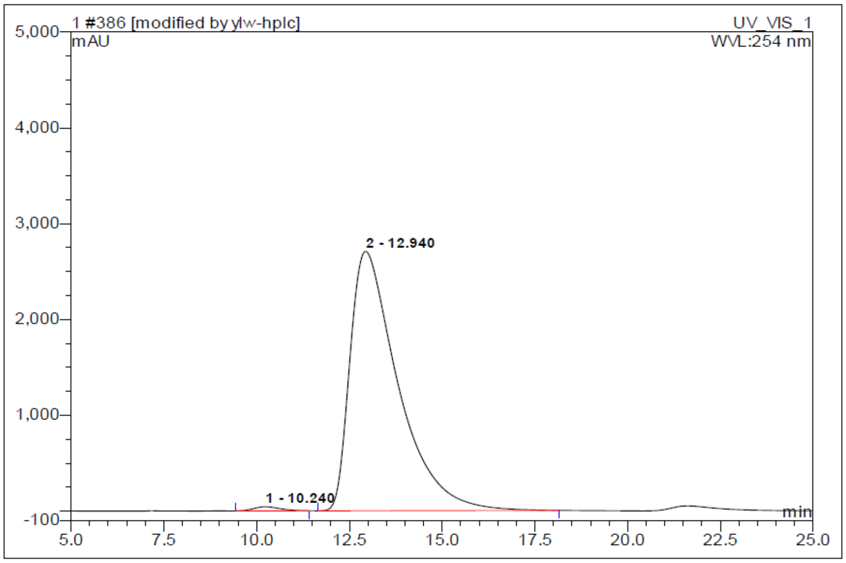

No.	Ret.Time min	Peak Name	Height mAU	Area mAU*min	Rel.Area %	Amount n.a.	Type
1	10.24	n.a.	42.491	35.252	0.86	n.a.	BMB*
2	12.94	n.a.	2707.758	4057.431	99.14	n.a.	BMB*
Total:			2750.249	4092.683	100.00	0.000	

Supplementary Figure 71. HPLC spectrum for compound 1s

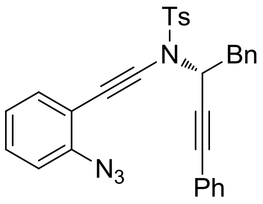

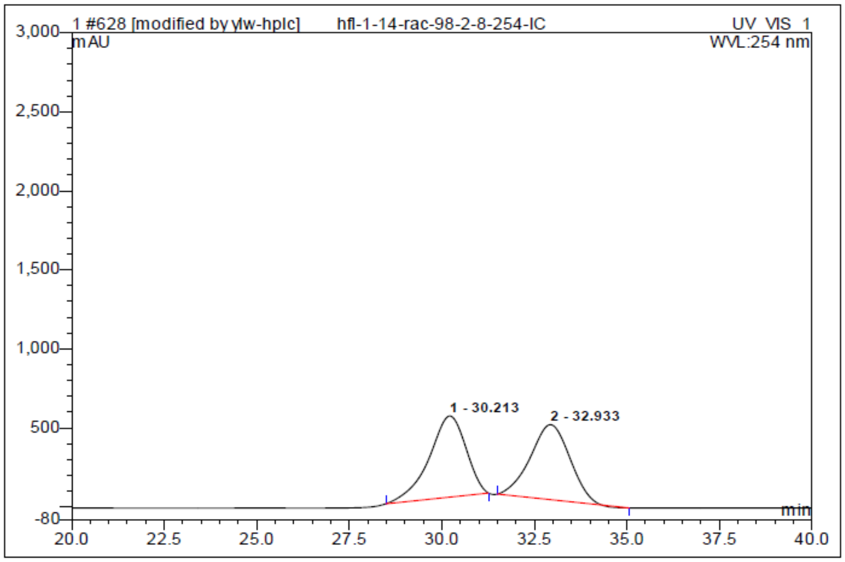

No.	Ret.Time min	Peak Name	Height mAU	Area mAU*min	Rel.Area %	Amount n.a.	Type
1	30.21	n.a.	512.906	592.461	50.24	n.a.	BMB*
2	32.93	n.a.	474.437	586.685	49.76	n.a.	BMB*
Total:			987.343	1179.146	100.00	0.000	

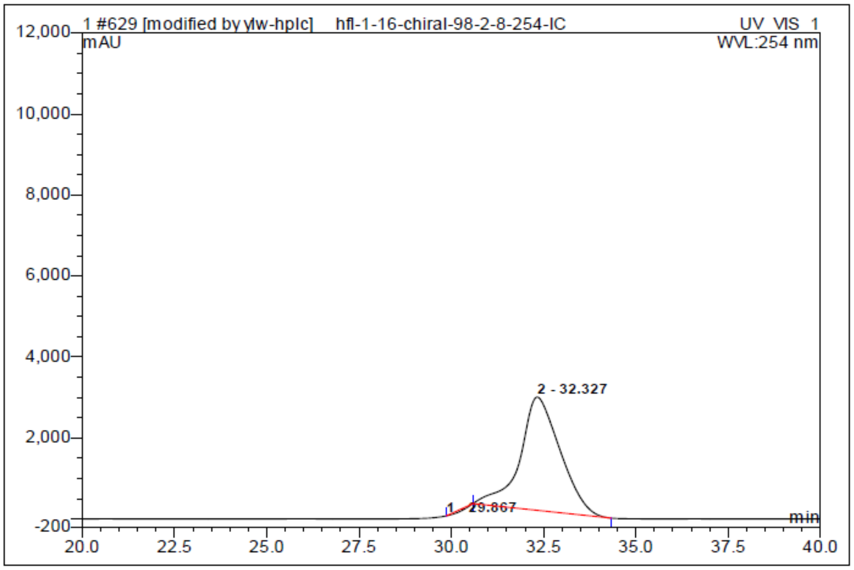

No.	Ret.Time min	Peak Name	Height mAU	Area mAU*min	Rel.Area %	Amount n.a.	Type
1	29.87	n.a.	1.513	21.214	0.59	n.a.	BMb*
2	32.33	n.a.	2796.109	3544.806	99.41	n.a.	bMB*
Total:			2797.622	3566.019	100.00	0.000	

Supplementary Figure 72. HPLC spectrum for compound 1t

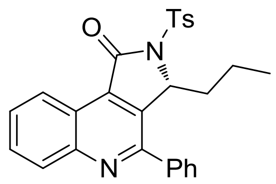

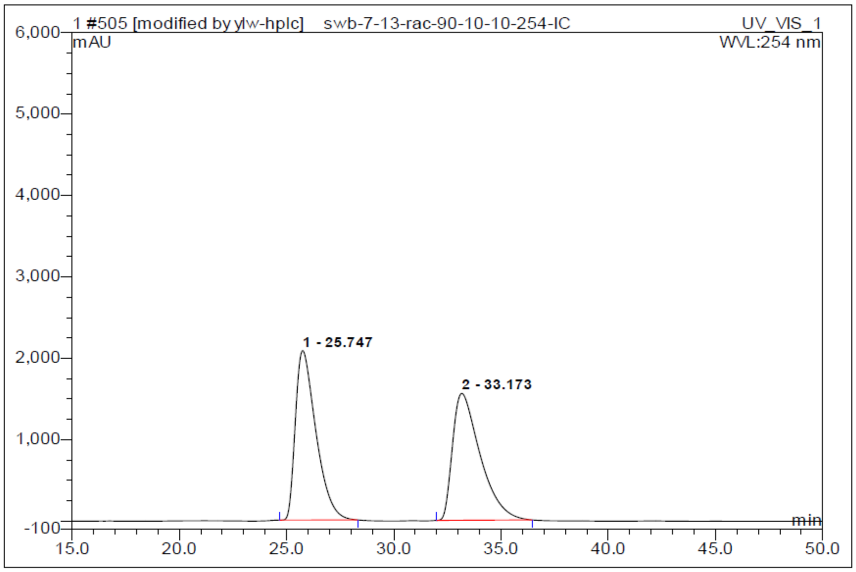

No.	Ret.Time min	Peak Name	Height mAU	Area mAU*min	Rel.Area %	Amount n.a.	Type
1	25.75	n.a.	2081.121	2320.170	49.88	n.a.	BMB*
2	33.17	n.a.	1558.788	2331.456	50.12	n.a.	BMB*
Total:			3639.909	4651.625	100.00	0.000	

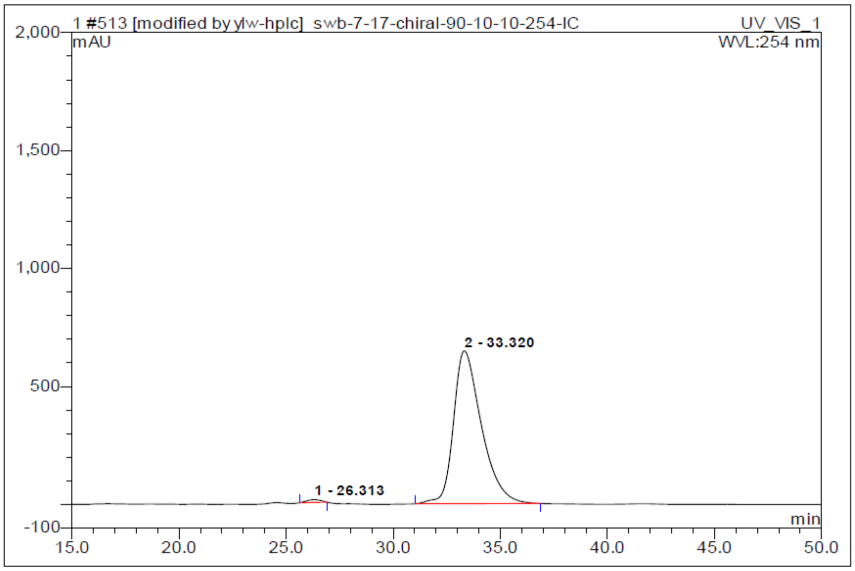

No.	Ret.Time min	Peak Name	Height mAU	Area mAU*min	Rel.Area %	Amount n.a.	Type
1	26.31	n.a.	12.011	9.058	0.91	n.a.	BMB*
2	33.32	n.a.	647.666	981.396	99.09	n.a.	BMB*
Total:			659.677	990.453	100.00	0.000	

Supplementary Figure 73. HPLC spectrum for compound 2s

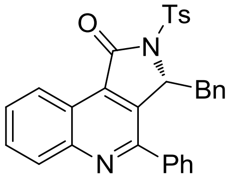

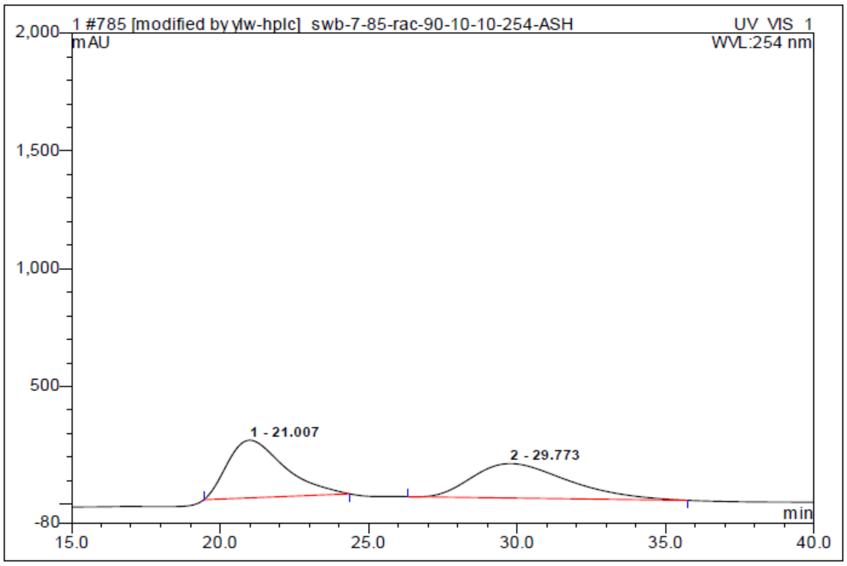

No.	Ret.Time min	Peak Name	Height mAU	Area mAU*min	Rel.Area %	Amount n.a.	Type
1	21.01	n.a.	244.742	546.474	49.98	n.a.	BMB*
2	29.77	n.a.	146.210	546.925	50.02	n.a.	BMB*
Total:			390.952	1093.400	100.00	0.000	

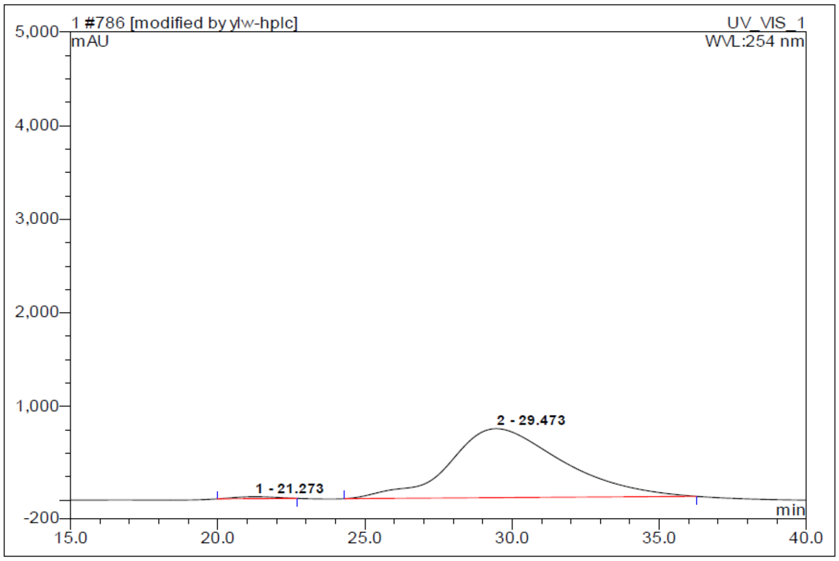

No.	Ret.Time min	Peak Name	Height mAU	Area mAU*min	Rel.Area %	Amount n.a.	Type
1	21.27	n.a.	20.743	32.808	0.99	n.a.	BMB*
2	29.47	n.a.	736.828	3296.182	99.01	n.a.	BMB*
Total:			757.571	3328.990	100.00	0.000	

Supplementary Figure 74. HPLC spectrum for compound 2t

Supplementary Tables

Supplementary Table 1. More reaction condition studies[a]

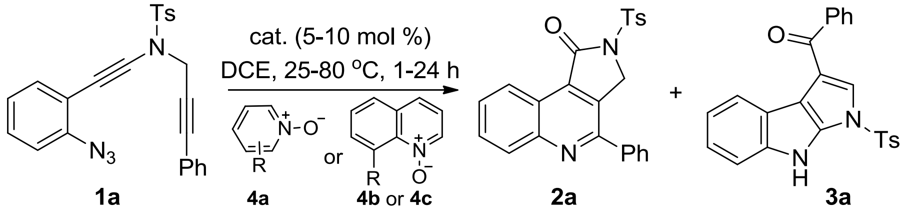

entry	catalyst	oxidant (R)	reaction conditions	yield (%)[b] 2a	3a	1a
1	TfOH (10 mol %)	**4a** (2-Br)	DCE, 80 °C, 2 h	<1	<1	<1
2	MsOH (10 mol %)	**4a** (2-Br)	DCE, 80 °C, 2 h	<1	<1	<1
3	TsOH (10 mol %)	**4a** (2-Br)	DCE, 80 °C, 2 h	<1	<1	<1
4	CF_3CO_2H (10 mol %)	**4a** (2-Br)	DCE, 80 °C, 24 h	<1	<1	10
5	–	**4a** (2-Br)	DCE, 80 °C, 1 h	<1	<1	<1
6	$CyJohnPhosAuNTf_2$ (5 mol %)	**4a** (2-Br)	DCE, 80 °C, 1 h	7	38	<1
7	$XPhosAuNTf_2$ (5 mol %)	**4a** (2-Br)	DCE, 80 °C, 1 h	<5	23	<1
8	$Cu(CH_3CN)_4PF_6$ (10 mol %)	**4c** (*i*-Pr)	DCE, 80 °C, 1 h	70	<1	<1
9	$Cu(CH_3CN)_4PF_6$ (10 mol %)	**4b** (Me)	toluene, 80 °C, 10 h	15	<1	<1
10	$Cu(CH_3CN)_4PF_6$ (10 mol %)	**4b** (Me)	PhCl, 80 °C, 10 h	61	<1	<1
11	$Cu(CH_3CN)_4PF_6$ (10 mol %)	**4b** (Me)	DCE, 40 °C, 3 h	82	<1	<1
12	$Cu(CH_3CN)_4BF_4$ (10 mol %)	**4b** (Me)	DCE, 60 °C, 2 h	74	<1	<1
13	$Cu(hfacac)_2$ (10 mol %)	**4b** (Me)	DCE, 60 °C, 2 h	7	<1	6
14	CuI (10 mol %)	**4b** (Me)	DCE, 60 °C, 5 h	<1	<1	90
15	$CyJohnPhosAuNTf_2$ (5 mol %)	–	DCE, rt, 1 h	<1	47	<1
16	$XPhosAuNTf_2$ (5 mol %)	–	DCE, rt, 24 h	<1	27	44
17	(**Ar**O)$_3$PAuNTf$_2$ (5 mol %)	–	DCE, rt, 1 h	<1	57	<1
18	$IPrAuNTf_2$ (5 mol %)	–	DCE, 80 °C, 1 h	<1	44	<1
19	$Cu(CH_3CN)_4PF_6$ (10 mol %)	–	DCE, 80 °C, 1 h	<1	<1	<1
20	$Cu(OTf)_2$(10 mol %)	–	DCE, 80 °C, 1 h	<1	<1	<1
21	$Ph_3PAuNTf_2$ (5 mol %)	–	toluene, rt, 1 h	<1	<1	<1
22	$Ph_3PAuNTf_2$ (5 mol %)	–	MeCN, rt, 1 h	<1	<1	<1
23[c]	$Ph_3PAuNTf_2$ (2 mol %)	–	CH_3NO_2, rt, 30 min	<1	86	<1

[a] The reaction was performed with **1a** (0.1 mmol), **4** (0.15 mmol), and catalyst (2-10 mol %) in solvent (2 mL) at rt-80 °C. [b] Measured by ^1H NMR using diethyl phthalate as the internal standard. [c] The reaction was performed in a flame-dried vial with dry CH_3NO_2 as the solvent and H_2O (2 equiv) as an additive.

Supplementary Table 2. Crystal data and structure refinement for 2a. CCDC Number = 1535333

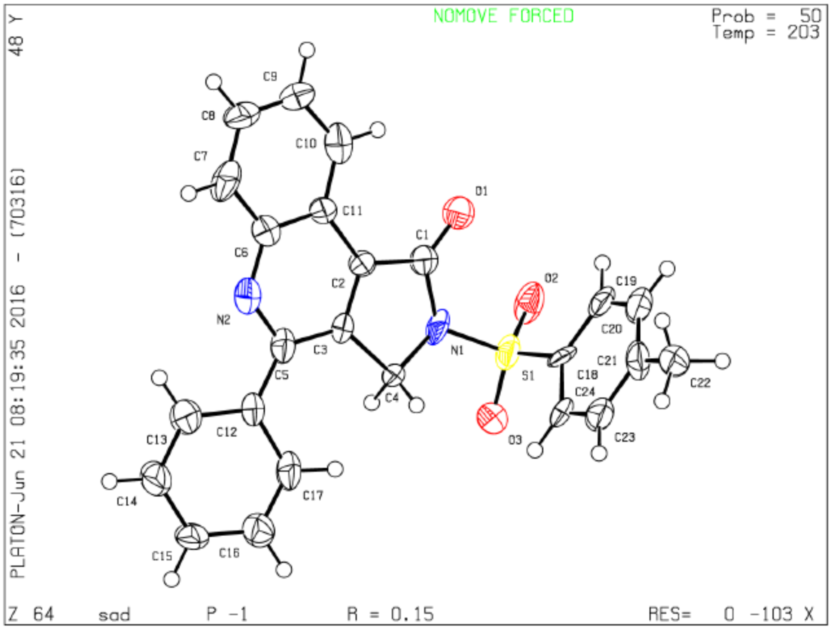

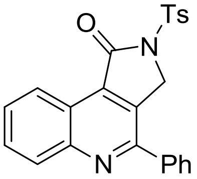

Bond precision: C-C = 0.0159 A Wavelength=0.71073

Cell:	a=8.158(5)	b=10.196(7)	c=11.553(7)
	alpha=88.931(15)	beta=73.956(11)	gamma=83.851(13)
Temperature:	203 K		

	Calculated	Reported
Volume	918.2(10)	918.3(10)
Space group	P -1	P -1
Hall group	-P 1	-P 1
Moiety formula	C24 H18 N2 O3 S	C24 H18 N2 O3 S
Sum formula	C24 H18 N2 O3 S	C24 H18 N2 O3 S
Mr	414.46	414.46
Dx,g cm-3	1.499	1.499
Z	2	2
Mu (mm-1)	0.208	0.208
F000	432.0	432.0
F000'	432.43	
h,k,lmax	9,12,13	9,12,13
Nref	3253	3193
Tmin,Tmax	0.963,0.969	0.116,1.000
Tmin'	0.959	

Correction method= # Reported T Limits: Tmin=0.116 Tmax=1.000
AbsCorr = MULTI-SCAN

Data completeness= 0.982 Theta(max)= 25.018

R(reflections)= 0.1455(922) wR2(reflections)= 0.3851(3193)

S = 0.844 Npar= 272

Supplementary Table 3. Crystal data and structure refinement for 3a. CCDC Number = 1535335

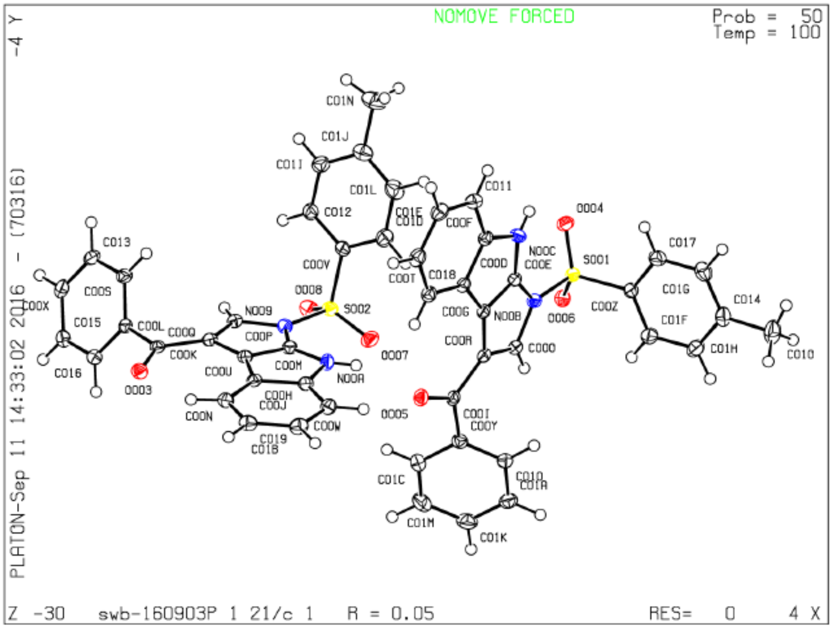

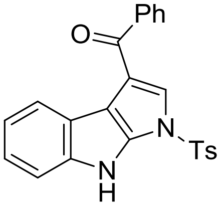

Bond precision: C-C = 0.0023 A Wavelength=1.54184

Cell:	a=12.6556(2)	b=18.6258(3)	c=16.9411(3)
	alpha=90	beta=94.8397(15)	gamma=90
Temperature:	100 K		

	Calculated	Reported
Volume	3979.13(11)	3979.11(12)
Space group	P 21/c	P 1 21/c 1
Hall group	-P 2ybc	-P 2ybc
Moiety formula	C24 H18 N2 O3 S	4(C24 H18 N2 O3 S)
Sum formula	C24 H18 N2 O3 S	C96 H72 N8 O12 S4
Mr	414.46	1657.85
Dx,g cm-3	1.384	1.384
Z	8	2
Mu (mm-1)	1.688	1.688
F000	1728.0	1728.0
F000'	1735.57	
h,k,lmax	15,23,21	15,23,20
Nref	8193	7936
Tmin,Tmax	0.817,0.845	0.771,1.000
Tmin'	0.776	

Correction method= # Reported T Limits: Tmin=0.771 Tmax=1.000
AbsCorr = MULTI-SCAN

Data completeness= 0.969 Theta(max)= 74.887

R(reflections)= 0.0472(6891) wR2(reflections)= 0.1381(7936)

S = 1.033 Npar= 685

Supplementary Notes

Computational methods.

All calculations were carried out with the Gaussian 09 programs[1]. The geometries of all the species were fully optimized by using the density functional theory (DFT) method with the M06[2] functional. The 6-31G (d, p)[3-7] basis set was used for C, H, N, O, F, S and P. For Au, we used the Stuttgart/Dresden small-core RECP (relativistic effective core potential) plus valence double-ζ basis set (SDD) [8], which has been frequently used in the mechanistic investigations on Au-catalyzed organic transformations.[9-12] The Lanl (Los Alamos National Laboratory) basis sets, also known as LanL2DZ (Lanl-2-double zeta)[13] was used for Br. These basis sets are denoted as DZP (double-ζ plus polarization). Frequency calculations at the same theoretical level (SMD-M06/DZP) were performed to confirm each stationary point to be either a local minimum or a transition state (TS). The transition states were verified by intrinsic reaction coordinate (IRC)[14] calculations. The intermediates were characterized by all real frequencies. The solvent effects of DCE (ε = 10.125) for Cu^{I} catalysis and nitromethane (ε = 36.562) for Au^{I} catalysis were taken in account by using the SMD-flavor[15] of self-consistent reaction field (SCRF) theory. For Au^{I} catalysis, the active catalytic species R_3PAu^+ is simplified as H_3PAu^+ in the computations[16,17] in order to reduce the computational cost.

In addition, we should mention that both LanL2DZ and SDD basis sets for Cu have been proven to be appropriate for the mechanistic investigations of Cu-based systems[18-23], despite that we adopted LanL2DZ for Cu in the currently-concerned systems. Indeed, this is supported by the computational data given in Supplementary Table 4 that were obtained by using both LanL2DZ and SDD for Cu for a series of key structures (precursor **A**, transition states $\mathbf{TS_B}$, $\mathbf{TS_C}$, $\mathbf{TS_{B'}}$ and $\mathbf{TS_{C'}}$) in the Cu^{I}-catalyzed reactions.

Supplementary Table 4. Relative free energies (in kcal/mol) of key structures (computed at the SMD-M06/DZP level of theory in DCE solvent for Cu^{I} catalysis at 298K).

Basis set for Cu	A	TS_B	TS_C	$TS_{B'}$	$TS_{C'}$
LanL2DZ	0.0	17.0	25.6	23.4	19.1
SDD	0.0	18.0	25.4	23.0	19.2

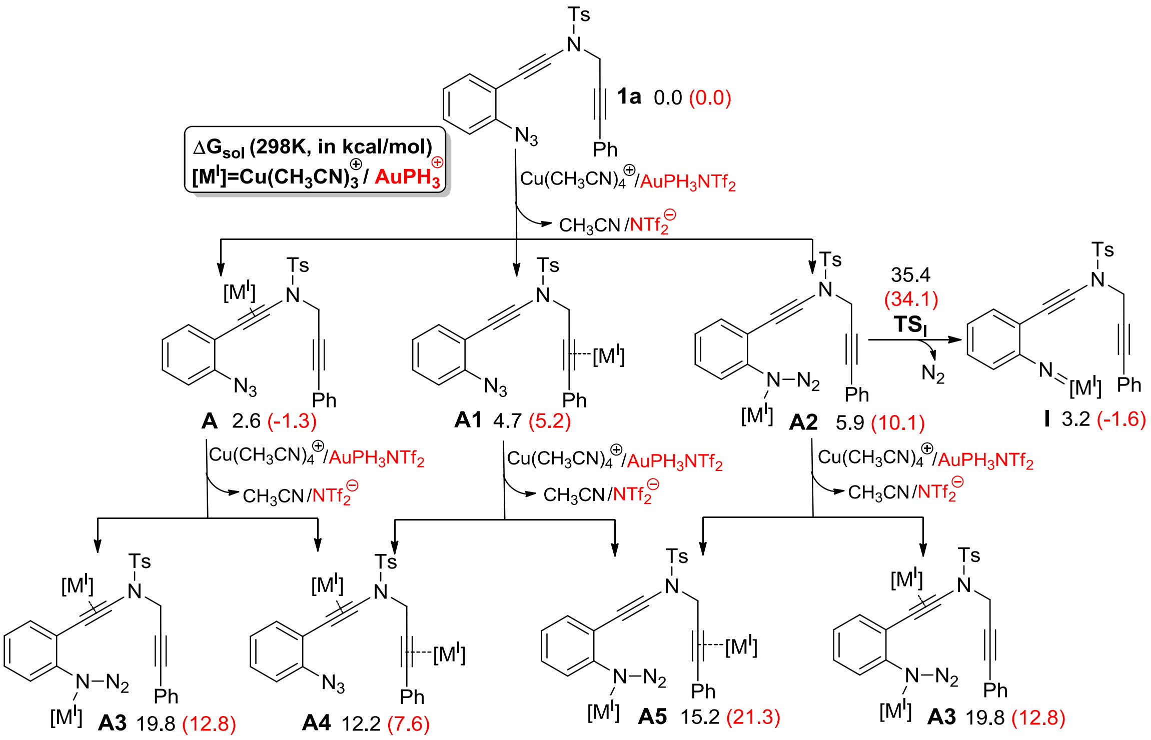

Supplementary Figure 75. The possible coordination of Cu[I] and Au[I] catalyst with substrate **1a** and energies for the formation of nitrene-like intermediates. Relative free energies of key intermediates and transition states were computed at the SMD-M06/DZP level of theory in solvent (DCE for Cu[I] catalysis and CH_3NO_2 for Au[I] catalysis) at 298K. Data for Au[I] catalysis were given in parentheses.

Supplementary Figure 75 shows the complexes formed by the Au[I]/Cu[I]-catalytic species and the substrate **1a**, along with the DFT-computed relative free energies. Based on calculated energies, we can summary our findings as follows. First, the Au[I]/Cu[I]-catalytic species is preferentially coordinated to the electron-richer triple bond neighboring the electron-donative amide group to form precursor **A**. Second, simultaneous coordination of metal to both triple bonds or either one of them and the proximal N atom of azide group seem to be unfavorable. Third, direct generation of metal-nitrene intermediate upon metal coordinating to the azide group appears to be implausible in the currently-concerned systems, as the barrier height (at transition state $\mathbf{TS_I}$) of such a process is too high (> 34 kcal/mol).

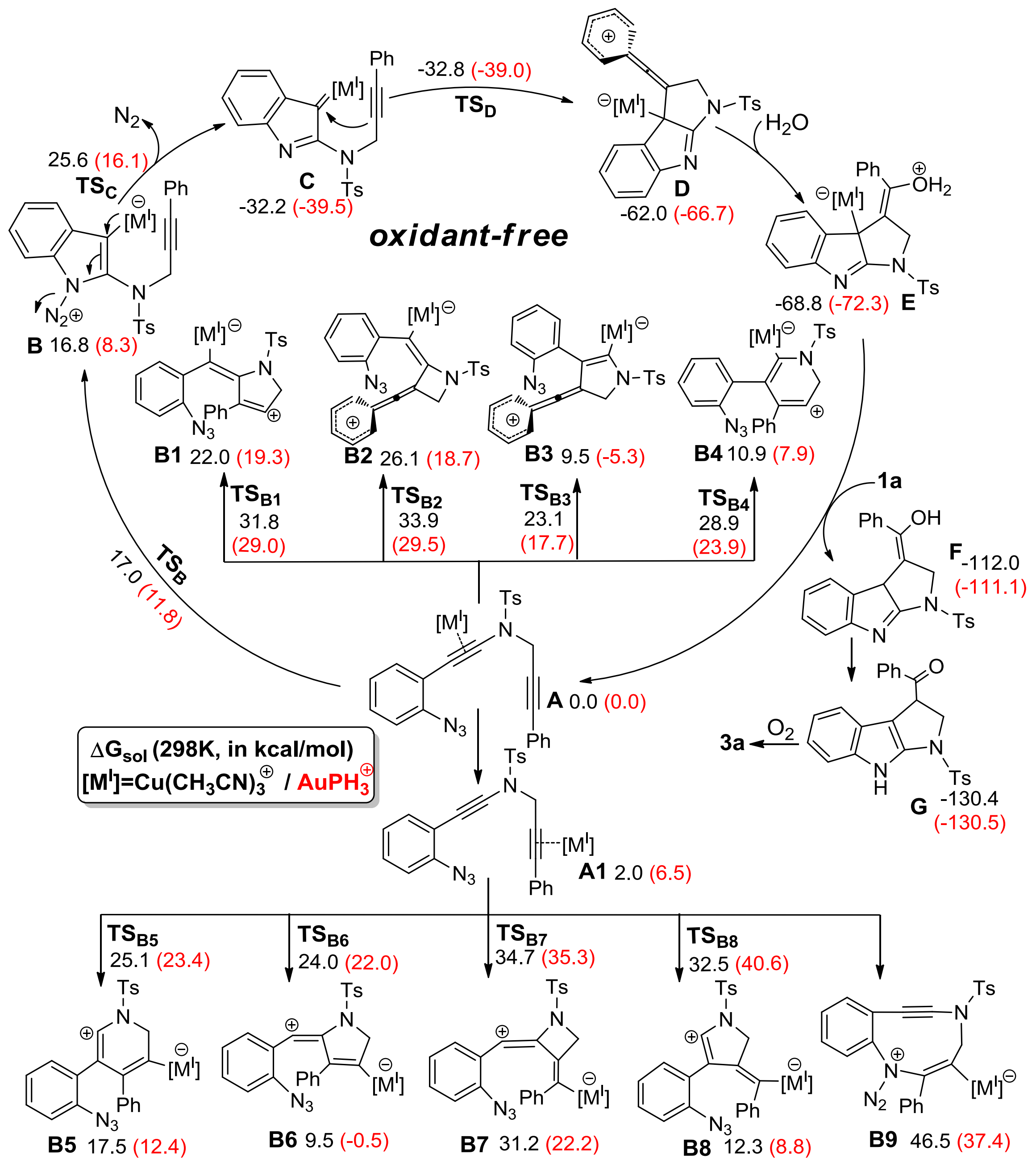

Supplementary Figure 76. Plausible mechanisms for the formation of pyrrolo[2,3-*b*] indoles **3a** in the oxidant-free cycle. Relative free energies of key intermediates and transition states were computed at the SMD-M06/DZP level of theory in solvent (DCE for Cu^{I} catalysis and CH_3NO_2 for Au^{I} catalysis) at 298K. Data for Au^{I} catalysis were given in parentheses.

Supplementary Figure 76 clearly shows that a series of reactions are carried out from precursor **A** in the oxidant-free cycle. Firstly, intramolecular cyclization of

precursor **A** either it is initiated by nucleophilic attack of the proximal N atom of azide to form intermediate **B** or competitive attack of another triple bond to generate intermediate **B1**，**B2**，**B3** and **B4**, respectively. The latters are unfavorable because the barrier heights (at transition state **TS$_{B3}$**, **TS$_{B4}$**, **TS$_{B5}$** and **TS$_{B6}$**: > 23.0 kcal/mol for Cu^{I} catalysis; > 17.0 kcal/mol for Au^{I} catalysis) of these processes are much higher than the former (at transition state **TS$_{B}$**: 17.0 kcal/mol for Cu^{I} catalysis; 11.8 kcal/mol for Au^{I} catalysis). Moreover, precursor **A** can also transform into sub-stable intermediate **A1** and then competitive intramolecular cyclization would occur to generate intermediate **B5**，**B6**，**B7**, **B8** and **B9**, respectively. However, the current calculation results illustrate that these processes are also unfavorable. Then, elimination of **N_2** from **B** affords the metal-carbenoid intermediate **C**, and a second cyclization to the enylium-cationic intermediate **D**. The latter can readily react with ambient H_2O, leading eventually to product **3a** via the following successive steps: H_2O addition, proton demetallization, keto-enol tautomerization and dehydrogenative oxidation.

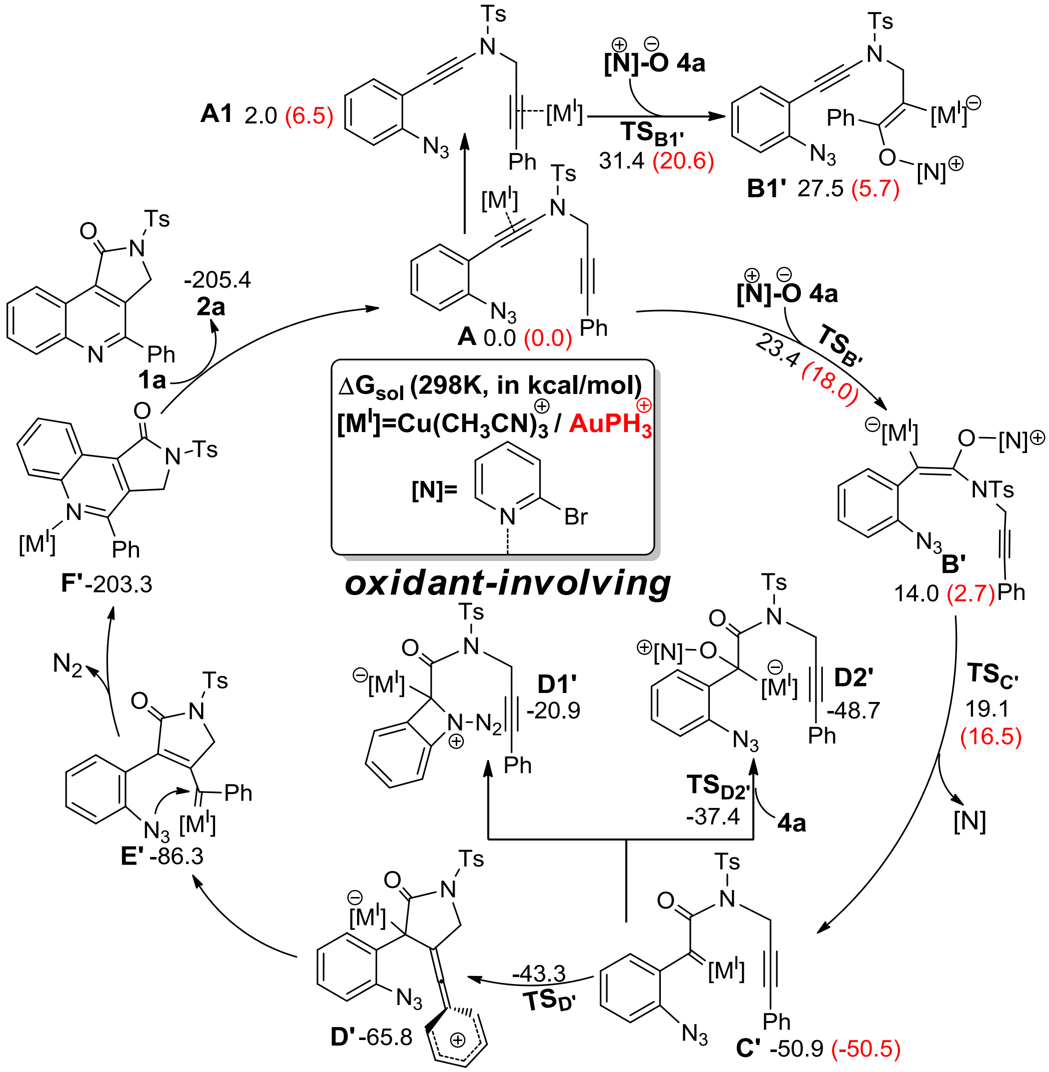

Supplementary Figure 77. Plausible mechanisms for the formation of pyrrolo[3,4-*c*] quinolin-1-ones **2a** in the oxidant-involving cycle. Relative free energies of key intermediates and transition states were computed at the SMD-M06/DZP level of theory in solvent (DCE for Cu^{I} catalysis and CH_3NO_2 for Au^{I} catalysis) at 298K. Data for Au^{I} catalysis were given in parentheses.

Plausible mechanisms for the formation of **2a** in the oxidant-involving cycle are illustrated in **Supplementary Figure 77**. Initially, nucleophilic attack of oxidant **4a** onto the precursor **A** forms vinyl-metal intermediate **B'** or onto the sub-stable intermediate **A1** generates intermediate **B1'**. However, based on calculated energies, the latter is thermodynamically and kinetically unfavorable. Subsequently, upon N–O bond cleavage, **B'** can transform into α-oxo metal-carbenoid intermediate **C'**. Then, penta and tetra-

cyclization as well as overoxidation of intermediate **C'** would occur to form intermediate **D'**, **D1'** and **D2'**, respectively. Note that generating more stable intermediate **D'** is favorable with a lower barrier than intermediate **D2'**. Furthermore, tetra-cyclization of intermediate **C'** to afford intermediate **D1'** is implausible since the process is highly endogenic. Ultimately, **D'** would furnish the product **2a** via catalyst migration, intramolecular nucleophilic cylization, the loss of dinitrogen and substrate exchange.

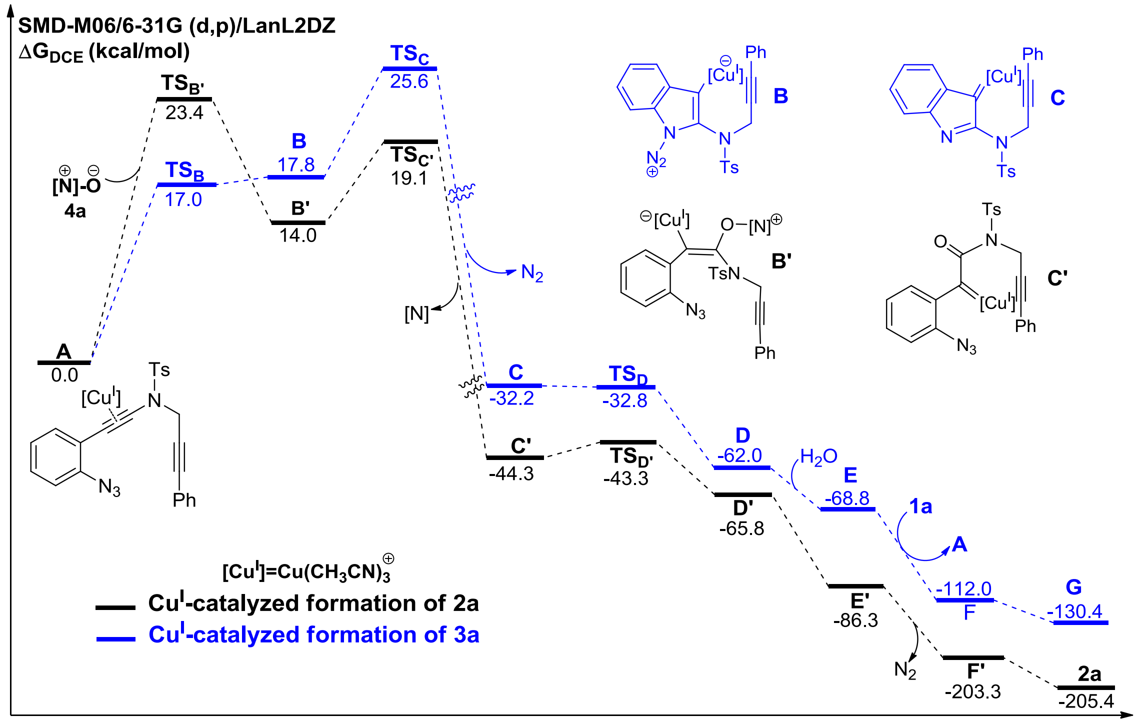

Supplementary Figure 78. Reaction energy profiles for Cu^{I}-catalyzed formation of **2a** (black line) and **3a** (blue line).

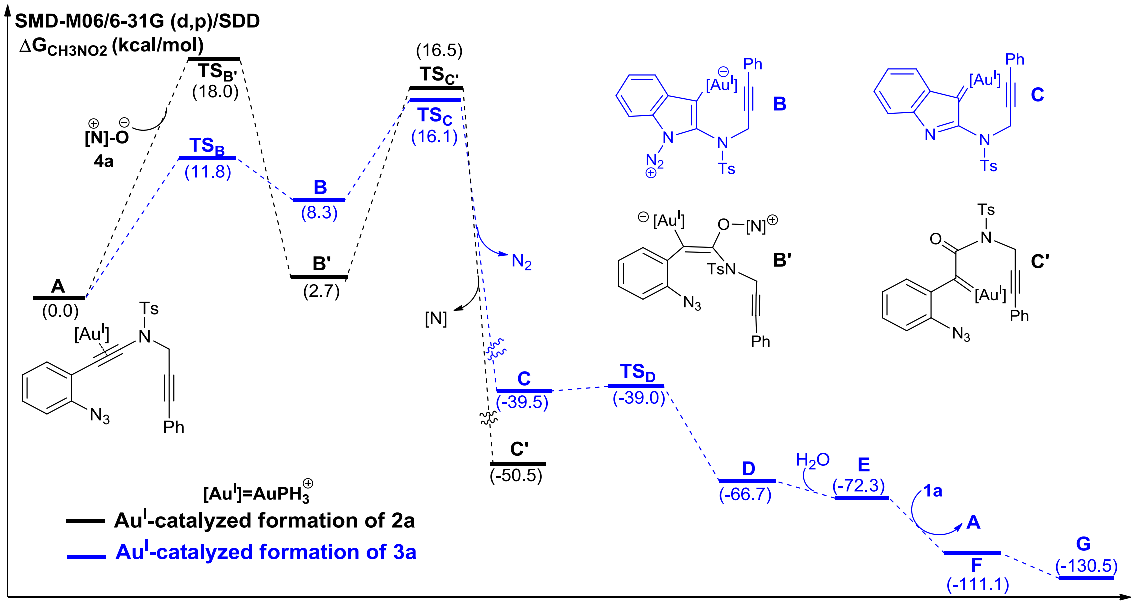

Supplementary Figure 79. Reaction energy profiles for Au^{I}-catalyzed formation of **2a** (black line) and **3a** (blue line).

Supplementary Discussion

More Mechanism Studies

For more mechanism studies on the gold-catalyzed cascade cyclization reaction, please see as followed.

First, the addition of water significantly promoted the cascade reaction when the reaction ran in a flame-dried vial with dry DCE as the solvent (this acceleration is not obvious in CH_3NO_2 as the reaction is too fast). These results support that water presumably participates in this cascade cyclization.

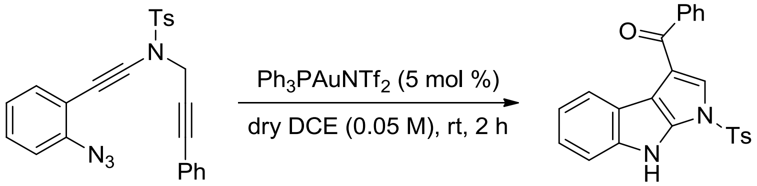

1a **3a**

1) H_2O (10 equiv): >95% conversion, 56% yield
2) H_2O (5 equiv): >95% conversion, 55% yield
3) H_2O (0 equiv): 70% conversion, 29% yield

Supplementary Figure 80. The effect of water on the gold-catalyzed cascade reaction.

Second, control experiments with $H_2{}^{18}O$ and $^{18}O_2$ isotopic labeling proved that the oxygen atom in the carbonyl group of **3a** originates from water but not molecular oxygen. Of note, no incorporation of ^{18}O into the **3a** was observed when **3a** was subjected to the reaction conditions with $H_2{}^{18}O$.

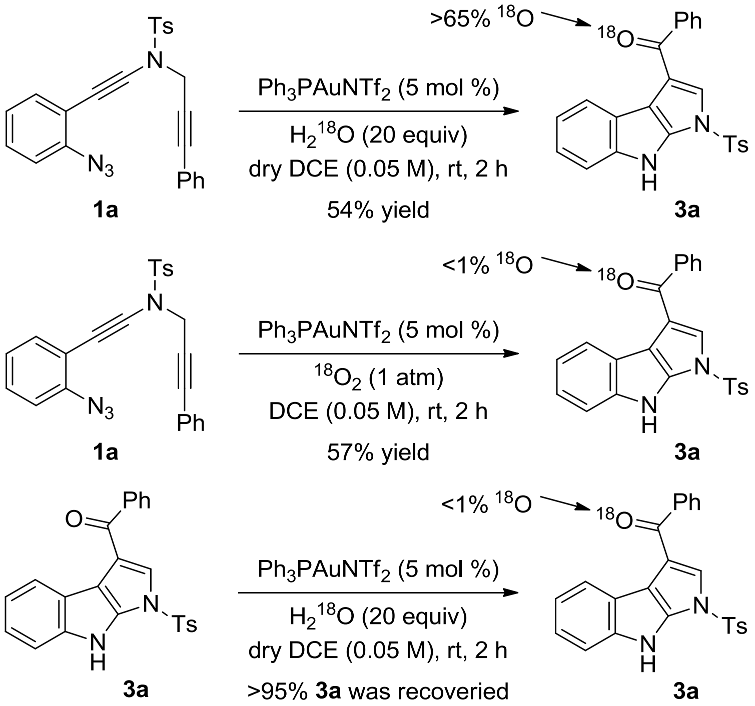

Supplementary Figure 81. Control experiments with $H_2{}^{18}O$ and $^{18}O_2$ isotopic labeling.

Third, the addition of styrene did not significantly affect this cascade cyclization and no cyclopropane formation was observed under standard conditions, which further supports that the reaction presumably does not involve a gold-carbene intermediate **H**.

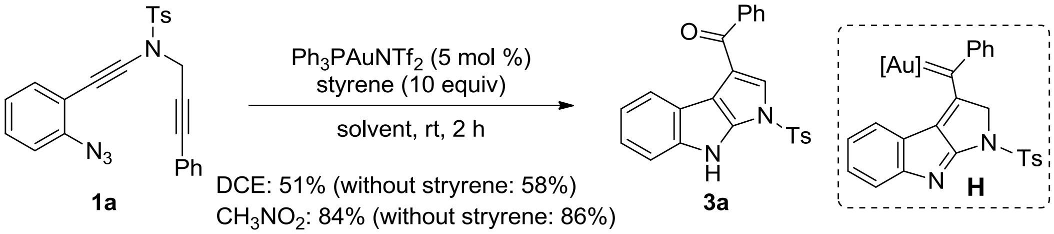

Supplementary Figure 82. Trying to trap the reaction intermediate with styrene.

Thus, the above control experiments clearly indicate that the path b involving the direct oxidation of gold carbene into the corresponding carbonyl group is less likely.

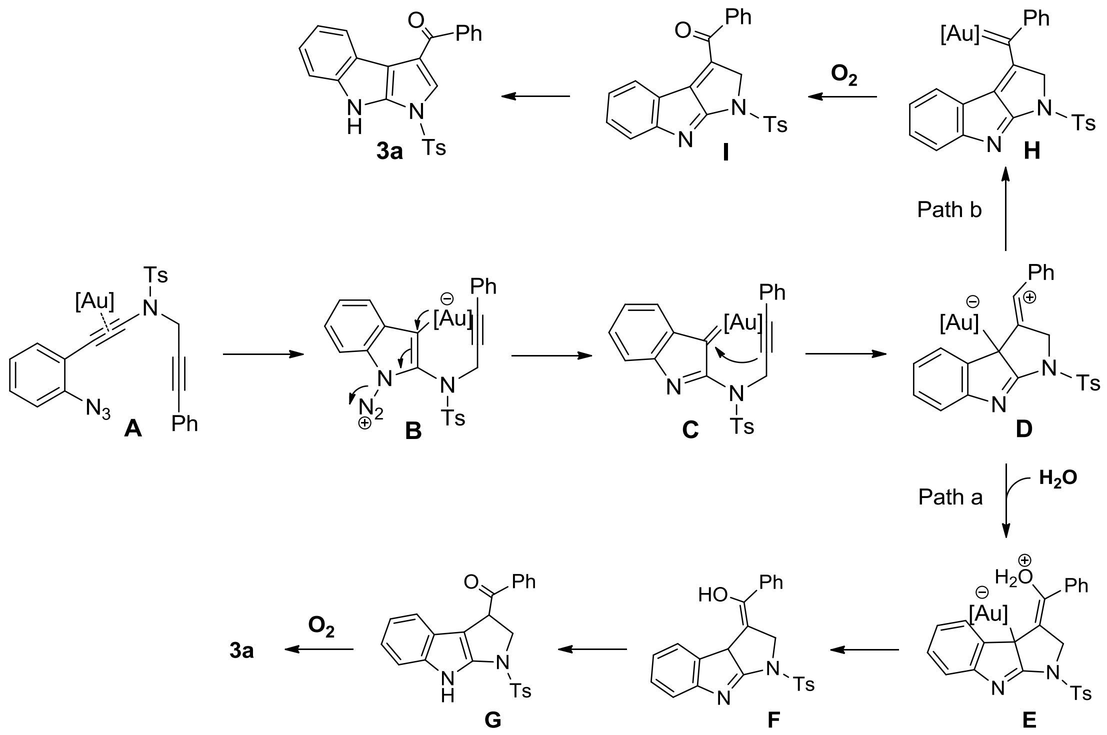

Supplementary Figure 83. Two plausible reaction pathways.

In addition, the reason that gold catalysts show much better reactivity than copper catalysts in this cascade cyclization can be explained by the fact that Au(I), as a cationic late-transition metal, has stronger π-backdonation effect than Cu(II) or Cu(I). As a result, the corresponding α-imino gold carbenes **C** can been readily generated while the generation of such a α-imino copper carbene is very difficult and has not been reported. This result is also well supported by DFT calculations, and please see the section of Computational Studies for details.

Of note, the dehydrogenative oxidation of intermediate **G** (or intermediate **F**) into the final **3a** by trace air in the reaction system is probably assisted by the carbonyl group of **G**[24] via a presumable radical process[25,26], This speculation is well supported by the following control experiment. When we tried to synthesize the key intermediate **3ab** according to the literature method[27], only the dehydrogenative oxidation product **3aa** was obtained.

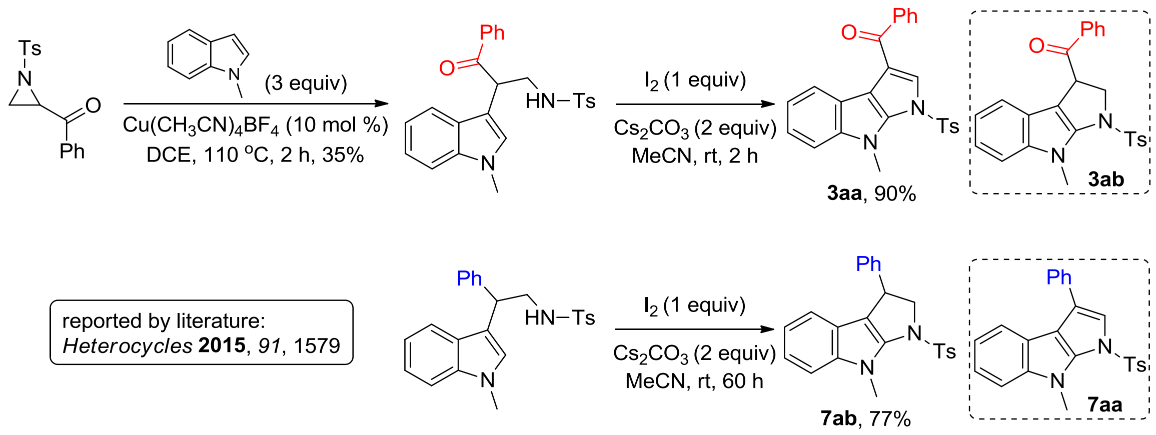

Supplementary Figure 84. Synthesis of dehydrogenative oxidation product **3aa**.

For more mechanism studies on the copper-catalyzed oxidative cyclization reaction, please see as followed.

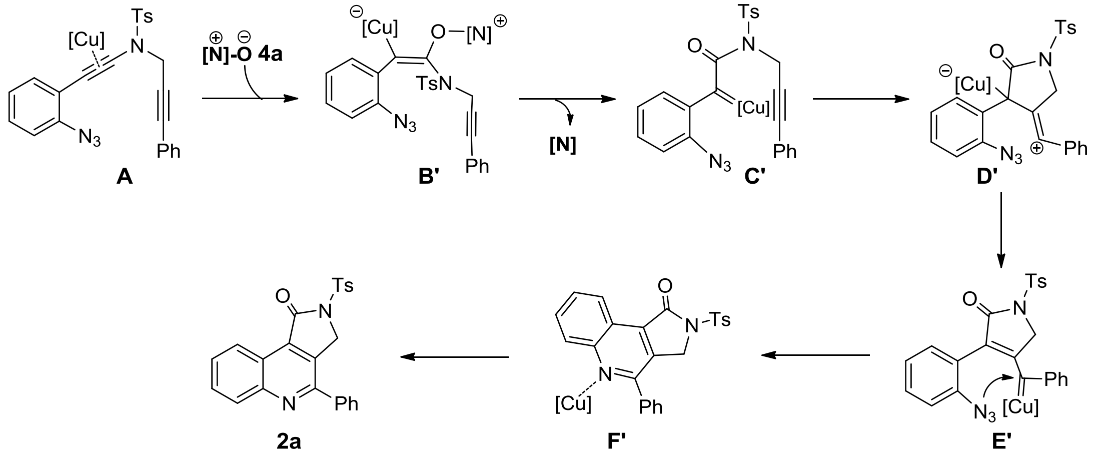

Supplementary Figure 85. Plausible reaction mechanism for the copper-catalyzed oxidative cyclization reaction.

First, as seen from the Supplementary Table 1, the copper catalysts especially the Cu(I) show much better reactivity than other non-noble metals such as In(OTf)$_3$ and Y(OTf)$_3$, which are difficult in the generation of the corresponding α-oxo metal carbene due to their weak π-backdonation effect. These results further support that α-oxo metal carbene intermediate **C'** is presumably involved in this oxidative cyclization.

In addition, the presumable copper carbene intermediates **C'** and **E'** have also been reported in the relevant copper-catalyzed cyclization of alkyne-tethered diazo compounds (R. Yao, G. Rong, B. Yan, L. Qiu, X. Xu, *ACS Catal.* **2016**, *6*, 1024).

Moreover, the above proposed mechanism is also well supported by DFT calculations, and please see the section of Computational Studies for details.

Supplementary Methods

General Information

Unless otherwise noted, materials were obtained commercially and used without further purification. All the solvents were treated according to general methods. Flash column chromatography was performed over silica gel (300-400 mesh). ^{1}H NMR spectra and ^{13}C NMR spectra were recorded on a Bruker AV-400 spectrometer and a Bruker AV-500 spectrometer in chloroform-d_3. For ^{1}H NMR spectra, chemical shifts are reported in ppm with the internal TMS signal at 0.0 ppm as a standard. For ^{13}C NMR spectra, chemical shifts are reported in ppm with the internal chloroform signal at 77.0 ppm as a standard. Infrared spectra were recorded on a Nicolet AVATER FTIR330 spectrometer as thin film and are reported in reciprocal centimeter (cm^{-1}). Mass spectra were recorded with Micromass QTOF2 Quadrupole/Time-of-Flight Tandem mass spectrometer using electron spray ionization.

Experimental Section

Representative synthetic procedures for the preparation of ynamides 1 (1a-1r)[28]:

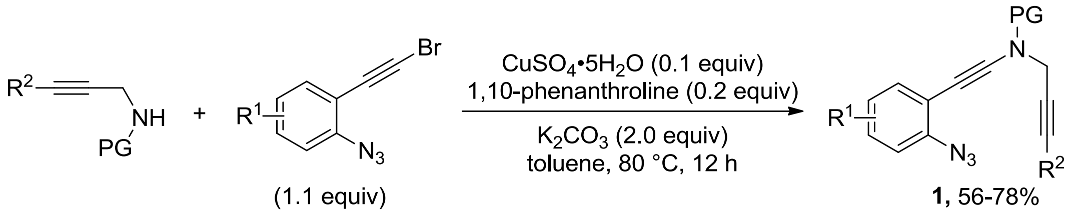

Supplementary Figure 86. Representative synthetic procedures for the preparation of ynamides **1** (**1a**-**1r**).

***N*-((2-azidophenyl)ethynyl)-4-methyl-*N*-(3-phenylprop-2-yn-1-yl)benzene sulfonamide (1a)**

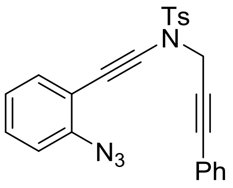

1a

Yellow solid (mp 72-74 ℃). ^{1}H NMR (400 MHz, $CDCl_3$) δ 7.97 (d, *J* = 8.0 Hz, 2H), 7.37 (d, *J* = 7.6 Hz, 1H), 7.30 – 7.23 (m, 6H), 7.18 (d, *J* = 6.9 Hz, 2H), 7.07 (dd, *J* = 13.6, 7.5 Hz, 2H), 4.58 (s, 2H), 2.35 (s, 3H); ^{13}C NMR (100 MHz, $CDCl_3$) δ 144.9, 141.0, 134.3, 133.5, 131.6, 129.5, 129.2, 128.6, 128.4, 128.1, 124.6, 122.1, 118.6, 114.9, 87.5, 86.6, 81.1, 67.1, 42.9, 21.6; IR (neat): 3443, 2234, 2128, 1490, 1368, 1301, 1170, 1088, 1037, 755, 659, 591; HRESIMS Calcd for $[C_{24}H_{18}N_4NaO_2S]^+$ (M + Na^+) 449.1043, found 449.1057.

***N*-((2-azidophenyl)ethynyl)-*N*-(3-(4-fluorophenyl)prop-2-yn-1-yl)-4-methylbenzene sulfonamide (1b)**

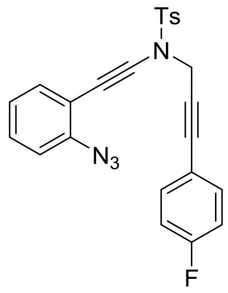

1b

Yellow oil. ^{1}H NMR (500 MHz, $CDCl_3$) δ 7.97 (d, *J* = 8.3 Hz, 2H), 7.37 (d, *J* = 7.7 Hz, 1H), 7.30 (t, *J* = 7.1 Hz, 3H), 7.17 (dd, *J* = 8.6, 5.5 Hz, 2H), 7.07 (dd, *J* = 14.8, 7.7 Hz, 2H), 6.94 (t, *J* = 8.7 Hz, 2H), 4.56 (s, 2H), 2.37 (s, 3H); ^{13}C NMR (125 MHz, $CDCl_3$) δ 162.6 (d, *J* = 248.8 Hz), 144.9, 140.9, 134.3, 133.6 (d, *J* = 7.5 Hz), 133.5, 129.5, 129.2, 128.4, 124.6, 118.6, 118.1 (d, *J* = 3.8 Hz), 115.4 (d, *J* = 21.3 Hz), 114.8, 87.3, 85.5, 80.8 (d, *J* = 1.3 Hz), 67.0, 42.8, 21.6; IR (neat): 3470, 2234, 2127, 1598, 1506, 1490, 1368, 1296, 1170, 837, 754, 586; HRESIMS Calcd for $[C_{24}H_{17}FN_4NaO_2S]^+$ (M + Na^+) 467.0948, found 467.0951.

***N*-((2-azidophenyl)ethynyl)-*N*-(3-(4-chlorophenyl)prop-2-yn-1-yl)-4-methylbenzene sulfonamide (1c)**

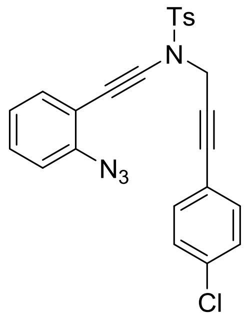

1c

Yellow oil. ^{1}H NMR (500 MHz, $CDCl_3$) δ 7.96 (d, *J* = 8.1 Hz, 2H), 7.36 (d, *J* = 7.7 Hz, 1H), 7.32 – 7.26 (m, 3H), 7.22 (d, *J* = 8.3 Hz, 2H), 7.11 – 7.04 (m, 4H), 4.56 (s, 2H), 2.37 (s, 3H); ^{13}C NMR (125 MHz, $CDCl_3$) δ 144.9, 140.9, 134.6, 134.2, 133.4, 132.8, 129.5, 129.2, 128.4, 128.3, 124.6, 120.5, 118.6, 114.7, 87.3, 85.4, 82.1, 67.0, 42.7, 21.5; IR (neat): 2920, 2234, 2128, 1489, 1369, 1303, 1170, 1089, 752, 662, 599; HRESIMS Calcd for $[C_{24}H_{17}ClN_4NaO_2S]^+$ (M + Na^+) 483.0653, found 483.0652.

N-((2-azidophenyl)ethynyl)-N-(3-(4-bromophenyl)prop-2-yn-1-yl)-4-methylbenzene sulfonamide (1d)

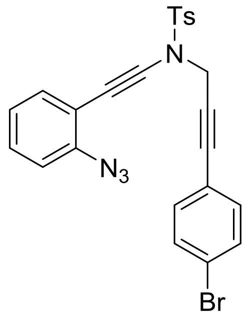

1d

Yellow oil. ^{1}H NMR (500 MHz, $CDCl_3$) δ 7.96 (d, *J* = 8.2 Hz, 2H), 7.39 – 7.35 (m, 3H), 7.32 – 7.28 (m, 3H), 7.07 (t, *J* = 8.0 Hz, 2H), 7.03 (d, *J* = 8.4 Hz, 2H), 4.55 (s, 2H), 2.37 (s, 3H); ^{13}C NMR (125 MHz, $CDCl_3$) δ 144.9, 140.9, 134.2, 133.4, 133.0, 131.4, 129.5, 129.2, 128.3, 124.6, 122.8, 120.9, 118.6, 114.7, 87.3, 85.5, 82.3, 67.0, 42.8, 21.5; IR (neat): 3460, 2919, 2127, 1645, 1486, 1368, 1170, 1089, 754, 594; HRESIMS Calcd for $[C_{24}H_{17}BrN_4NaO_2S]^+$ (M + Na^+) 527.0148, found 527.0156.

N-((2-azidophenyl)ethynyl)-4-methyl-N-(3-(p-tolyl)prop-2-yn-1-yl)benzene sulfonamide (1e)

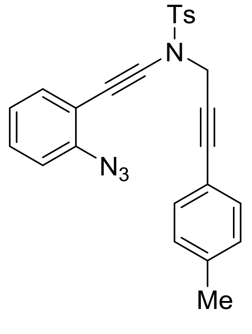

1e

Yellow oil. ^{1}H NMR (400 MHz, $CDCl_3$) δ 7.97 (d, *J* = 8.2 Hz, 2H), 7.36 (d, *J* = 7.6 Hz, 1H), 7.32 – 7.28 (m, 3H), 7.09 – 7.03 (m, 6H), 4.56 (s, 2H), 2.36 (s, 3H), 2.32 (s, 3H); ^{13}C NMR (125 MHz, $CDCl_3$) δ 144.9, 140.9, 138.7, 134.3, 133.5, 131.5, 129.5, 129.1, 128.8, 128.4, 124.5, 118.9, 118.6, 114.9, 87.4, 86.8, 80.3, 67.0, 42.9, 21.6, 21.4; IR (neat):

2234, 2128, 1596, 1491, 1445, 1368, 1304, 1170, 1089, 755, 587; HRESIMS Calcd for $[C_{25}H_{20}N_4NaO_2S]^+$ (M + Na$^+$) 463.1199, found 463.1197.

***N*-((2-azidophenyl)ethynyl)-*N*-(3-(4-methoxyphenyl)prop-2-yn-1-yl)-4-methyl benzenesulfonamide (1f)**

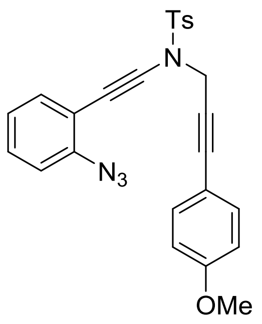

1f

Yellow solid (mp 56-58 ℃). ^{1}H NMR (500 MHz, $CDCl_3$) δ 7.97 (d, *J* = 8.3 Hz, 2H), 7.36 (d, *J* = 7.7 Hz, 1H), 7.32 – 7.26 (m, 3H), 7.12 (d, *J* = 8.7 Hz, 2H), 7.06 (dd, *J* = 15.3, 8.0 Hz, 2H), 6.77 (d, *J* = 8.8 Hz, 2H), 4.56 (s, 2H), 3.78 (s, 3H), 2.37 (s, 3H); ^{13}C NMR (125 MHz, $CDCl_3$) δ 159.7, 144.9, 140.9, 134.3, 133.4, 133.1, 129.5, 129.1, 128.3, 124.5, 118.6, 114.9, 114.1, 113.7, 87.5, 86.6, 79.6, 67.0, 55.2, 43.0, 21.6; IR (neat): 3396, 2918, 2234, 2128, 1606, 1509, 1367, 1293, 1249, 1169, 1034, 755, 588; HRESIMS Calcd for $[C_{25}H_{20}N_4NaO_3S]^+$ (M + Na$^+$) 479.1148, found 479.1160.

***N*-((2-azidophenyl)ethynyl)-*N*-(3-(3-bromophenyl)prop-2-yn-1-yl)-4-methylbenzene sulfonamide (1g)**

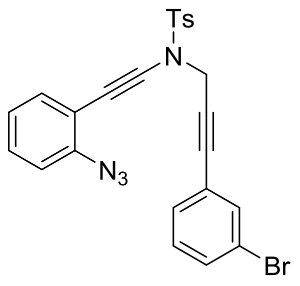

1g

Yellow oil. ^{1}H NMR (500 MHz, $CDCl_3$) δ 7.97 (d, *J* = 8.2 Hz, 2H), 7.43 – 7.41 (m, 1H), 7.38 (d, *J* = 7.7 Hz, 1H), 7.33 – 7.29 (m, 3H), 7.25 (d, *J* = 5.6 Hz, 1H), 7.15 – 7.06 (m, 4H), 4.57 (s, 2H), 2.40 (s, 3H); ^{13}C NMR (125 MHz, $CDCl_3$) δ 145.1, 141.0, 134.4, 134.3, 133.6, 131.7, 130.1, 129.6, 129.5, 129.3, 128.4, 124.6, 124.0, 121.9, 118.6, 114.8, 87.3, 85.0, 82.5, 67.1, 42.7, 21.7; IR (neat): 3477, 2234, 2127, 1490, 1368, 1299, 1170, 1088, 753, 665, 592; HRESIMS Calcd for $[C_{24}H_{17}BrN_4NaO_2S]^+$ (M + Na^+) 527.0148, found 527.0158.

N-((2-azidophenyl)ethynyl)-4-methyl-N-(3-(m-tolyl)prop-2-yn-1-yl)benzene sulfonamide (1h)

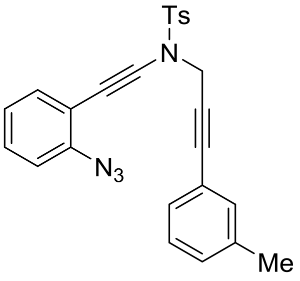

1h

Yellow oil. ^{1}H NMR (500 MHz, $CDCl_3$) δ 7.97 (d, *J* = 8.3 Hz, 2H), 7.37 (dd, *J* = 7.7, 1.3 Hz, 1H), 7.29 – 7.26 (m, 3H), 7.15 – 7.04 (m, 4H), 6.98 (d, *J* = 8.1 Hz, 2H), 4.57 (s, 2H), 2.36 (s, 3H), 2.28 (s, 3H); ^{13}C NMR (125 MHz, $CDCl_3$) δ 144.9, 140.9, 137.7, 134.3, 133.5, 132.2, 129.5, 129.4, 129.1, 128.7, 128.4, 128.0, 124.5, 121.8, 118.6, 114.9, 87.4, 86.8, 80.6, 67.0, 42.9, 21.5, 21.1; IR (neat): 2921, 2234, 2128, 1596, 1490, 1368, 1302, 1171, 1089, 1038, 921, 754, 592; HRESIMS Calcd for $[C_{25}H_{20}N_4NaO_2S]^+$ (M + Na^+) 463.1199, found 463.1195.

N-((2-azidophenyl)ethynyl)-4-methyl-N-(3-(thiophen-3-yl)prop-2-yn-1-yl)benzene sulfonamide (1i)

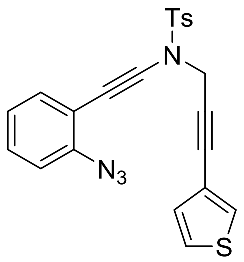

1i

Yellow oil. ^{1}H NMR (500 MHz, $CDCl_3$) δ 7.96 (d, *J* = 8.3 Hz, 2H), 7.36 (dd, *J* = 7.7, 1.2 Hz, 1H), 7.35 – 7.29 (m, 3H), 7.24 (d, *J* = 2.0 Hz, 1H), 7.20 (dd, *J* = 4.9, 3.0 Hz, 1H), 7.08 – 7.03 (m, 2H), 6.88 (dd, *J* = 4.9, 0.9 Hz, 1H), 4.55 (s, 2H), 2.38 (s, 3H); ^{13}C NMR (125 MHz, $CDCl_3$) δ 144.9, 140.9, 134.3, 133.4, 129.6, 129.5, 129.3, 129.1, 128.3, 125.2, 124.5, 121.1, 118.6, 114.9, 87.4, 81.8, 80.7, 67.1, 42.9, 21.6; IR (neat): 3454, 2234, 2128, 1490, 1367, 1302, 1169, 1088, 1037, 922, 754, 664, 591; HRESIMS Calcd for $[C_{22}H_{16}N_4NaO_2S_2]^+$ (M + Na^+) 455.0607, found 455.0616.

***N*-((2-azidophenyl)ethynyl)-*N*-(but-2-yn-1-yl)-4-methylbenzenesulfonamide (1j)**

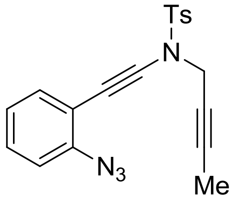

1j

Yellow solid (mp 59-61 ℃). ^{1}H NMR (400 MHz, $CDCl_3$) δ 7.94 (d, *J* = 8.3 Hz, 2H), 7.35 (d, *J* = 8.3 Hz, 3H), 7.31 – 7.23 (m, 1H), 7.14 – 7.01 (m, 2H), 4.29 (d, *J* = 2.3 Hz, 2H), 2.44 (s, 3H), 1.64 (t, *J* = 2.3 Hz, 3H); ^{13}C NMR (100 MHz, $CDCl_3$) δ 144.7, 140.6, 134.3, 133.3, 129.3, 129.0, 128.3, 124.5, 118.6, 115.0, 87.6, 83.0, 71.1, 66.8, 42.5, 21.6, 3.4; IR (neat): 3440, 2920, 2234, 2128, 1596, 1490, 1366, 1302, 1169, 1089, 923, 754, 586; HRESIMS Calcd for $[C_{19}H_{16}N_4NaO_2S]^+$ (M + Na^+) 387.0886, found 387.0893.

***N*-((2-azido-5-methylphenyl)ethynyl)-4-methyl-*N*-(3-phenylprop-2-yn-1-yl)benzene sulfonamide (1k)**

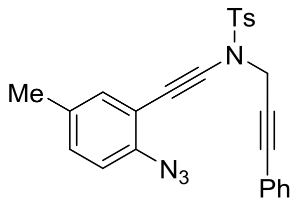

1k

Yellow oil. ^{1}H NMR (400 MHz, $CDCl_3$) δ 7.97 (d, *J* = 8.3 Hz, 2H), 7.30 – 7.23 (m, 5H), 7.22 – 7.17 (m, 3H), 7.08 (d, *J* = 8.2 Hz, 1H), 6.95 (d, *J* = 8.2 Hz, 1H), 4.56 (s, 2H), 2.35 (s, 3H), 2.26 (s, 3H); ^{13}C NMR (100 MHz, $CDCl_3$) δ 144.9, 138.1, 134.3, 134.2, 133.8, 131.6, 130.0, 129.5, 128.5, 128.3, 128.1, 122.0, 118.5, 114.5, 87.0, 86.6, 81.1, 67.2, 42.8, 21.5, 20.5; IR (neat): 2920, 2849, 2239, 2120, 1492, 1368, 1305, 1170, 1089, 1039, 811, 757, 592; HRESIMS Calcd for $[C_{25}H_{20}N_4NaO_2S]^+$ (M + Na^+) 463.1199, found 463.1198.

***N*-((2-azido-5-fluorophenyl)ethynyl)-4-methyl-*N*-(3-phenylprop-2-yn-1-yl)benzene sulfonamide (1l)**

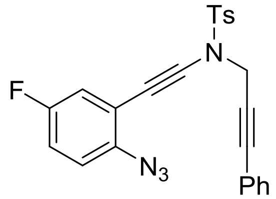

1l

Yellow oil. ^{1}H NMR (500 MHz, $CDCl_3$) δ 7.96 (d, *J* = 8.2 Hz, 2H), 7.31 – 7.23 (m, 5H), 7.17 (d, *J* = 7.1 Hz, 2H), 7.05 (d, *J* = 8.8 Hz, 1H), 7.06 – 6.98 (m, 2H), 4.58 (s, 2H), 2.35 (s, 3H); ^{13}C NMR (125 MHz, $CDCl_3$) δ 159.0 (d, *J* = 243.8 Hz), 145.1, 136.8 (d, *J* = 2.5 Hz), 134.1, 131.6, 129.6, 128.6, 128.3, 128.1, 121.9, 120.0 (d, *J* = 8.8 Hz), 119.5 (d, *J* = 25.0 Hz), 116.3 (d, *J* = 22.5 Hz), 88.4, 86.7, 80.8, 66.4 (d, *J* = 2.5 Hz), 42.8, 21.5; IR (neat): 3440, 2920, 2849, 2241, 2123, 1491, 1370, 1170, 1089, 1037, 876, 757, 591; HRESIMS Calcd for $[C_{24}H_{17}FN_4NaO_2S]^+$ (M + Na^+) 467.0948, found 467.0953.

***N*-((2-azido-5-chlorophenyl)ethynyl)-4-methyl-*N*-(3-phenylprop-2-yn-1-yl)benzene sulfonamide (1m)**

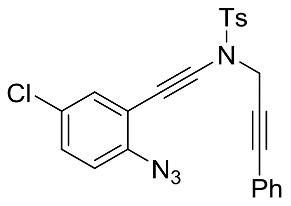

1m

Yellow oil. ^{1}H NMR (500 MHz, $CDCl_3$) δ 7.95 (d, *J* = 8.1 Hz, 2H), 7.32 – 7.24 (m, 7H), 7.18 (d, *J* = 7.3 Hz, 2H), 6.99 (d, *J* = 8.6 Hz, 1H), 4.57 (s, 2H), 2.36 (s, 3H); ^{13}C NMR (125 MHz, $CDCl_3$) δ 145.1, 139.4, 134.2, 132.7, 131.6, 129.7, 129.6, 129.0, 128.6, 128.3, 128.1, 121.9, 119.8, 116.4, 88.6, 86.7, 80.8, 66.2, 42.8, 21.6; IR (neat): 3445, 2919, 2115, 1645, 1488, 1370, 1301, 1169, 757, 591; HRESIMS Calcd for $[C_{24}H_{17}ClN_4NaO_2S]^+$ (M + Na^+) 483.0653, found 483.0659.

***N*-((2-azido-5-bromophenyl)ethynyl)-4-methyl-*N*-(3-phenylprop-2-yn-1-yl)benzene sulfonamide (1n)**

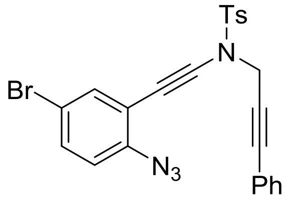

1n

Yellow oil. ^{1}H NMR (400 MHz, $CDCl_3$) δ 7.95 (d, *J* = 8.3 Hz, 2H), 7.46 (d, *J* = 2.2 Hz, 1H), 7.36 (d, *J* = 8.6 Hz, 1H), 7.30 – 7.24 (m, 5H), 7.18 (d, *J* = 6.8 Hz, 2H), 6.92 (d, *J* = 8.6 Hz, 1H), 4.57 (s, 2H), 2.35 (s, 3H); ^{13}C NMR (100 MHz, $CDCl_3$) δ 145.1, 139.9, 135.6, 134.2, 131.9, 131.6, 129.6, 128.6, 128.3, 128.1, 121.9, 120.0, 117.0, 116.8, 88.8, 86.7, 80.9, 66.1, 42.8, 21.5; IR (neat): 3467, 2919, 2234, 2109, 1645, 1482, 1369, 1298, 1169, 1090, 1036, 928, 756, 590; HRESIMS Calcd for $[C_{24}H_{17}BrN_4NaO_2S]^+$ (M + Na^+) 527.0148, found 527.0158.

***N*-((2-azido-4-chlorophenyl)ethynyl)-4-methyl-*N*-(3-phenylprop-2-yn-1-yl)benzene sulfonamide (1o)**

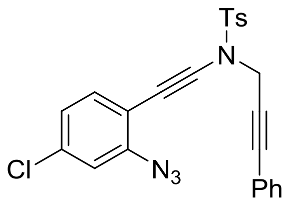

1o

Yellow oil. ^{1}H NMR (500 MHz, $CDCl_3$) δ 7.95 (d, *J* = 8.2 Hz, 2H), 7.30 – 7.23 (m, 6H), 7.17 (d, *J* = 7.2 Hz, 2H), 7.07 – 6.98 (m, 2H), 4.56 (s, 2H), 2.35 (s, 3H); ^{13}C NMR (125 MHz, $CDCl_3$) δ 145.0, 142.1, 134.7, 134.2, 131.6, 129.5, 128.6, 128.3, 128.1, 124.9, 121.9, 118.9, 113.5, 88.3, 86.7, 80.9, 66.2, 42.8, 21.5; IR (neat): 2236, 2109, 1589, 1489, 1401, 1369, 1291, 1171, 1102, 1089, 921, 758, 660, 578; HRESIMS Calcd for $[C_{24}H_{17}ClN_4NaO_2S]^+$ (M + Na^+) 483.0653, found 483.0660.

***N*-((2-azidophenyl)ethynyl)-*N*-(3-phenylprop-2-yn-1-yl)benzenesulfonamide (1p)**

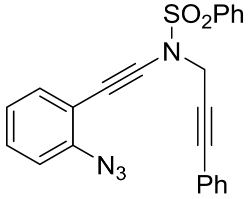

1p

Yellow oil. ^{1}H NMR (400 MHz, $CDCl_3$) δ 8.09 (d, *J* = 7.3 Hz, 2H), 7.59 (t, *J* = 7.4 Hz, 1H), 7.50 (t, *J* = 7.6 Hz, 2H), 7.35 (dd, *J* = 7.6, 1.3 Hz, 1H), 7.28 – 7.16 (m, 6H), 7.12 – 6.95 (m, 2H), 4.60 (s, 2H); ^{13}C NMR (100 MHz, $CDCl_3$) δ 140.9, 137.2, 133.8, 133.5, 131.6, 129.2, 128.9, 128.6, 128.3, 128.1, 124.5, 121.9, 118.6, 114.7, 87.1, 86.7, 80.9, 67.1, 42.9; IR (neat): 3062, 2925, 2235, 2128, 1569, 1490, 1447, 1369, 1302, 1173, 1089, 1038, 920, 754, 595; HRESIMS Calcd for $[C_{23}H_{16}N_4NaO_2S]^+$ (M + Na^+) 435.0886, found 435.0889.

***N*-((2-azidophenyl)ethynyl)-4-bromo-*N*-(3-phenylprop-2-yn-1-yl)benzene sulfonamide (1q)**

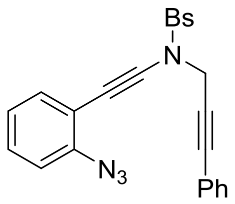

1q

Yellow solid (mp 104-106 ℃). ^{1}H NMR (500 MHz, $CDCl_3$) δ 7.95 (d, *J* = 8.3 Hz, 2H), 7.62 (d, *J* = 8.2 Hz, 2H), 7.37 (d, *J* = 7.7 Hz, 1H), 7.32 – 7.24 (m, 4H), 7.15 (d, *J* = 7.5 Hz, 2H), 7.09 – 7.04 (m, 2H), 4.60 (s, 2H); ^{13}C NMR (125 MHz, $CDCl_3$) δ 141.1, 136.1, 133.5, 132.2, 131.5, 129.8, 129.4, 129.2, 128.7, 128.3, 124.6, 121.6, 118.5, 114.4, 87.0, 86.7, 80.7, 67.2, 43.1; IR (neat):3444, 2925, 2235, 2128, 1734, 1573, 1490, 1372, 1301, 1174, 1086, 1009, 919, 755, 614; HRESIMS Calcd for $[C_{23}H_{15}BrN_4NaO_2S]^+$ (M + Na^+) 512.9991, found 512.9999.

***N*-((2-azidophenyl)ethynyl)-*N*-(3-phenylprop-2-yn-1-yl)methanesulfonamide (1r)**

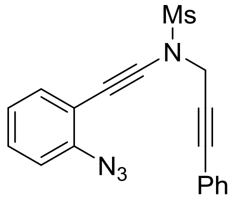

1r

Yellow solid (mp 50-52 ℃). ^{1}H NMR (500 MHz, $CDCl_3$) δ 7.47 (d, *J* = 7.8 Hz, 2H), 7.43 (d, *J* = 7.7 Hz, 1H), 7.38 – 7.31 (m, 4H), 7.13 – 7.04 (m, 2H), 4.62 (s, 2H), 3.32 (s, 3H); ^{13}C NMR (125 MHz, $CDCl_3$) δ 141.2, 133.7, 131.8, 129.5, 129.0, 128.4, 124.6, 121.7, 118.7, 114.4, 87.1, 86.7, 81.4, 67.2, 42.9, 38.8; IR (neat): 3459, 2922, 2236, 2127, 1644, 1490, 1361, 1299, 1166, 756, 692, 515; HRESIMS Calcd for $[C_{18}H_{14}N_4NaO_2S]^+$ (M + Na^+) 373.0730, found 373.0738.

Representative synthetic procedures for the preparation of ynamides 1 (1s-1t) [29]**:**

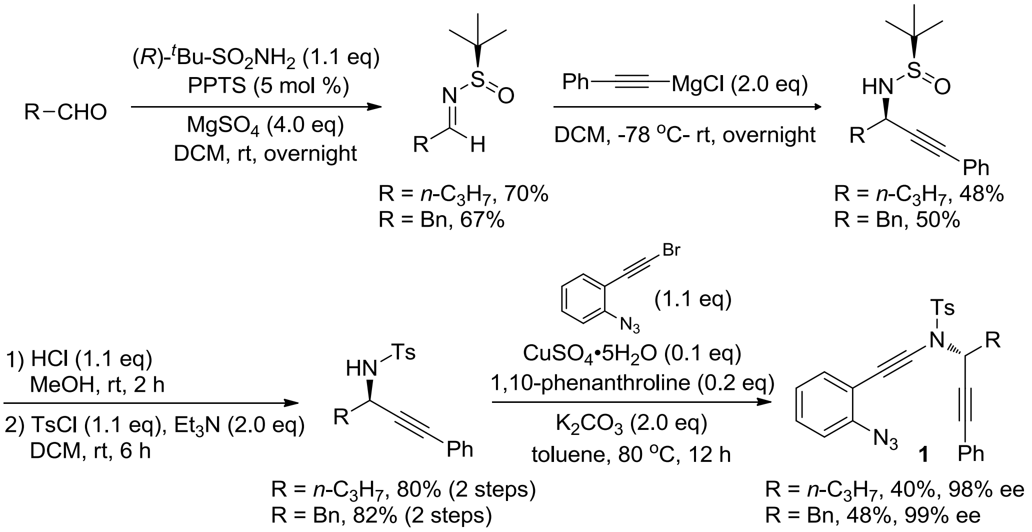

Supplementary Figure 87. Representative synthetic procedures for the preparation of ynamides **1** (**1s**-**1t**).

(*R*)-*N*-((2-azidophenyl)ethynyl)-4-methyl-*N*-(1-phenylhex-1-yn-3-yl)benzenesulfonamide (1s)

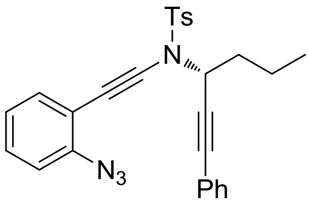

1s

Yellow solid (mp 85-87 ℃). $[\alpha]_D^{20}$ = +97.5 °(c = 1.0, $CHCl_3$). 98% ee (determined by HPLC: Chiralcel AS-H Column, 2/98 *i*-PrOH/hexane, 1.5 mL/min, 254 nm; TR = 12.94 min (major), 10.24 min (minor)). ^1H NMR (400 MHz, $CDCl_3$) δ 7.96 (d, *J* = 8.2 Hz, 2H), 7.38 (dd, *J* = 7.7, 1.1 Hz, 1H), 7.31 – 7.19 (m, 6H), 7.12 – 7.01 (m, 4H), 4.91 (t, *J* = 7.6 Hz, 1H), 2.31 (s, 3H), 2.08 – 1.87 (m, 2H), 1.62 – 1.53 (m, 2H), 1.01 (t, *J* = 7.4 Hz, 3H); ^{13}C NMR (100 MHz, $CDCl_3$) δ 144.7, 141.0, 134.6, 133.4, 131.5, 129.4, 129.0, 128.4, 128.3, 128.0, 124.5, 122.2, 118.5, 115.2, 85.8, 85.3, 85.0, 68.7, 53.2, 36.5, 21.5, 19.1, 13.4; IR (neat): 3443, 2921, 2234, 2128, 1506, 1489, 1369, 1249, 1170, 1089, 756, 669, 588; HRESIMS Calcd for $[C_{27}H_{24}N_4NaO_2S]^+$ (M + Na^+) 491.1512, found 491.1532.

(*R*)-*N*-((2-azidophenyl)ethynyl)-*N*-(1,4-diphenylbut-3-yn-2-yl)-4-methylbenzenesulfonamide (1t)

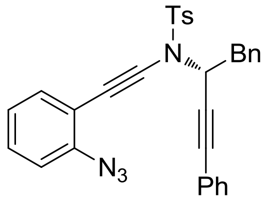

1t

Yellow solid (mp 87-89 ℃). $[\alpha]_D^{20}$ = -7.7 º (c = 1.0, $CHCl_3$). 99% ee (determined by HPLC: Chiralcel IC Column, 2/98 *i*-PrOH/hexane, 0.8 mL/min, 254 nm; TR = 32.33 min (major), 29.87 min (minor)). ^1H NMR (400 MHz, $CDCl_3$) δ 7.85 (d, *J* = 8.2 Hz, 2H), 7.41 (d, *J* = 7.7 Hz, 1H), 7.36 – 7.20 (m, 11H), 7.15 – 7.04 (m, 4H), 5.13 (t, *J* = 7.6 Hz, 1H), 3.38 – 3.30 (m, 1H), 3.27 – 3.19 (m, 1H), 2.32 (s, 3H); ^{13}C NMR (100 MHz, $CDCl_3$) δ 144.6, 141.1, 136.0, 134.6, 133.4, 131.5, 129.6, 129.4, 129.1, 128.5, 128.4, 128.3, 128.0, 127.1, 124.5, 122.1, 118.5, 115.1, 86.7, 85.2, 84.5, 69.5, 54.9, 40.8, 21.5; IR (neat): 3444, 2921, 2236, 2127, 1596, 1490, 1368, 1301, 1169, 1089, 942, 724, 661, 598; HRESIMS Calcd for $[C_{31}H_{24}N_4NaO_2S]^+$ (M + Na^+) 539.1512, found 539.1521.

***N*-((2-azido-5-(morpholinosulfonyl)phenyl)ethynyl)-4-methyl-*N*-(3-phenylprop-2-yn-1-yl)benzenesulfonamide (1u)**

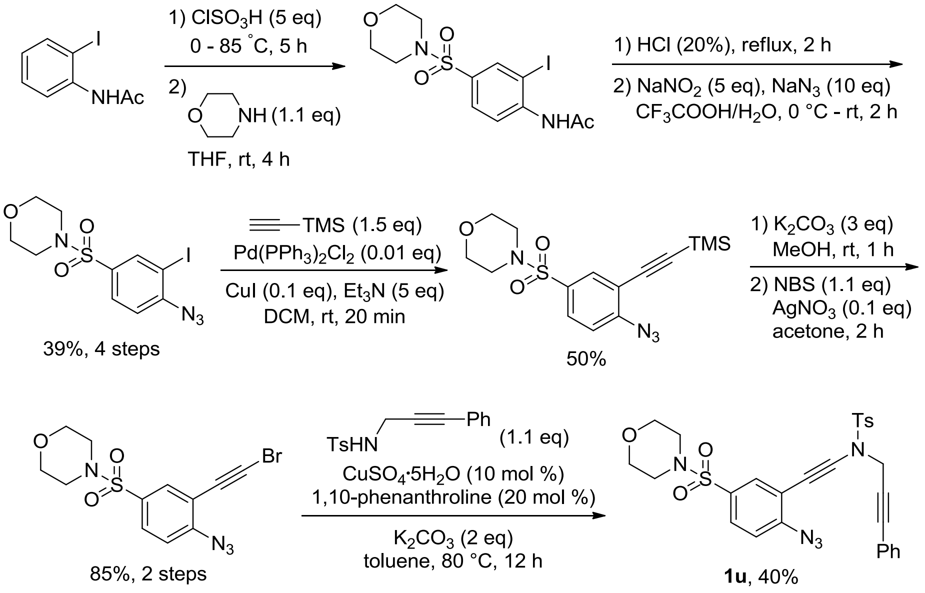

Supplementary Figure 88. Synthesis of ynamide **1u**.

Compound **1u** was prepared according to the known procedures[24-26,30]. Yellow solid (mp 71-73 °C). ^{1}H NMR (500 MHz, $CDCl_3$) δ 7.96 (d, *J* = 8.3 Hz, 2H), 7.71 (d, *J* = 2.1 Hz, 1H), 7.63 (dd, *J* = 8.5, 2.1 Hz, 1H), 7.33 – 7.24 (m, 5H), 7.22 – 7.18 (m, 3H), 4.60 (s, 2H), 3.72 (t, 4H), 2.97 (t, 4H), 2.38 (s, 3H); ^{13}C NMR (125 MHz, $CDCl_3$) δ 145.4, 145.3, 134.2, 132.5, 131.6, 131.3, 129.7, 128.7, 128.3, 128.2, 128.0, 121.9, 119.0, 116.1, 89.8, 86.9, 80.8, 66.1, 66.0, 45.9, 42.8, 21.6; IR (neat): 3440, 2920, 2234, 2128, 1596, 1491, 1369, 1268, 1170, 1088, 758, 664, 546; HRESIMS Calcd for $[C_{28}H_{25}N_5NaO_5S_2]^+$ (M + Na^+) 598.1189, found 598.1196.

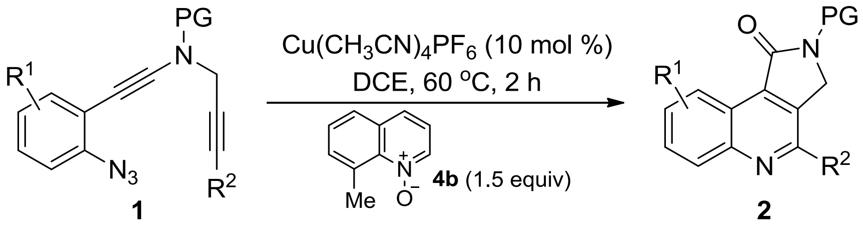

Supplementary Figure 89. Synthesis of pyrrolo[3,4-*c*]quinolin-1-ones **2**.

General procedure for the synthesis of pyrrolo[3,4-*c*]quinolin-1-ones 2:

Methylquinoline *N*-oxide (0.3 mmol, 47.7 mg) and $Cu(CH_3CN)_4PF_6$ (0.02 mmol, 7.5 mg) were added in this order to the ynamide **1** (0.20 mmol) in DCE (4.0 mL) at room temperature. The reaction mixture was stirred at 60 °C and the progress of the reaction was monitored by TLC. The reaction typically took 2 h. Upon completion, the mixture was then concentrated and the residue was purified by chromatography on silica gel (eluent: hexanes/dichloromethane) to afford the desired product **2**.

4-phenyl-2-tosyl-2,3-dihydro-1*H*-pyrrolo[3,4-*c*]quinolin-1-one (2a)

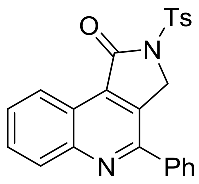

2a

Compound **2a** was prepared in 82% yield (67.9 mg) according to the general procedure (Table 2, entry 1). Yellow solid (mp 206-208 ℃). ^{1}H NMR (400 MHz, $CDCl_3$) δ 8.94 (d, *J* = 8.2 Hz, 1H), 8.25 (d, *J* = 8.5 Hz, 1H), 8.08 (d, *J* = 8.2 Hz, 2H), 7.88 (d, *J* = 6.9 Hz, 2H), 7.82 (t, *J* = 7.7 Hz, 1H), 7.69 (t, *J* = 7.6 Hz, 1H), 7.61 – 7.54 (m, 3H), 7.36 (d, *J* = 8.2 Hz, 2H), 5.19 (s, 2H), 2.43 (s, 3H); ^{13}C NMR (125 MHz, $CDCl_3$) δ 165.8, 154.0, 148.4, 145.7, 137.6, 135.0, 133.6, 133.3, 130.7, 130.1, 130.0, 129.9, 129.2, 129.0, 128.3, 128.2, 123.1, 122.1, 49.6, 21.7; IR (neat): 2923, 1732, 1594, 1494, 1442, 1365, 1330, 1173, 1145, 1089, 1058, 985, 770, 662, 578; HRESIMS Calcd for $[C_{24}H_{18}N_2NaO_3S]^+$ (M + Na^+) 437.0930, found 437.0936.

4-(4-fluorophenyl)-2-tosyl-2,3-dihydro-1*H*-pyrrolo[3,4-*c*]quinolin-1-one (2b)

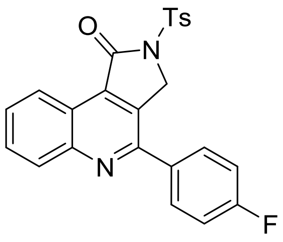

2b

Compound **2b** was prepared in 62% yield (53.6 mg) according to the general procedure (Table 2, entry 2). Yellow solid (mp 229-231 ℃). ^{1}H NMR (500 MHz, $CDCl_3$) δ 8.93 (d, *J* = 8.1 Hz, 1H), 8.23 (d, *J* = 8.5 Hz, 1H), 8.08 (d, *J* = 8.3 Hz, 2H), 7.90 (dd, *J* = 8.7, 5.3 Hz, 2H), 7.82 (t, *J* = 8.3 Hz, 1H), 7.69 (t, *J* = 7.2 Hz, 1H), 7.37 (d, *J* = 8.2 Hz, 2H), 7.32 – 7.26 (m, 2H), 5.17 (s, 2H), 2.43 (s, 3H); ^{13}C NMR (125 MHz, $CDCl_3$) δ 165.6, 163.9 (d, *J* = 250.0 Hz), 152.7, 148.3, 145.7, 135.0, 133.8 (d, *J* = 2.5 Hz), 133.4, 133.2, 130.8, 130.3 (d, *J* = 8.8 Hz), 129.9, 129.8, 129.0, 128.3, 123.1, 122.1, 116.3 (d, *J* = 21.3 Hz), 49.6, 21.7; IR (neat): 3476, 2920, 1729, 1586, 1498, 1459, 1364, 1295, 1171, 1145, 1089, 984, 769, 579; HRESIMS Calcd for $[C_{24}H_{17}FN_2NaO_3S]^+$ (M + Na^+) 455.0836, found 455.0841.

4-(4-chlorophenyl)-2-tosyl-2,3-dihydro-1*H*-pyrrolo[3,4-*c*]quinolin-1-one (2c)

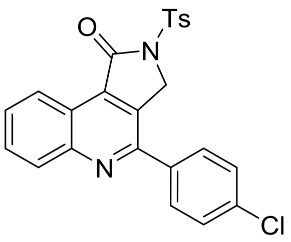

2c

Compound **2c** was prepared in 69% yield (61.8 mg) according to the general procedure (Table 2, entry 3). Yellow solid (mp 221-223 ℃). ^{1}H NMR (400 MHz, $CDCl_3$) δ 8.94 (d, *J* = 8.3 Hz, 1H), 8.23 (d, *J* = 8.6 Hz, 1H), 8.08 (d, *J* = 8.2 Hz, 2H), 7.87 – 7.81 (m, 3H), 7.70 (t, *J* = 7.5 Hz, 1H), 7.57 (d, *J* = 8.4 Hz, 2H), 7.37 (d, *J* = 8.2 Hz, 2H), 5.17 (s, 2H), 2.43 (s, 3H); ^{13}C NMR (125 MHz, $CDCl_3$) δ 165.6, 152.5, 148.3, 145.7, 136.4, 136.0, 135.0, 133.5, 133.2, 130.9, 130.0, 129.9, 129.6, 129.4, 129.2, 128.3, 123.2, 122.2, 49.5, 21.7; IR (neat): 3435, 2999, 2917, 1729, 1659, 1436, 1361, 1144, 1019, 952, 768, 658, 576; HRESIMS Calcd for $[C_{24}H_{17}ClN_2NaO_3S]^+$ (M + Na^+) 471.0541, found 471.0555.

4-(4-bromophenyl)-2-tosyl-2,3-dihydro-1*H*-pyrrolo[3,4-*c*]quinolin-1-one (2d)

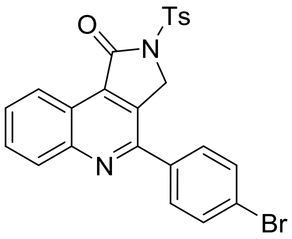

2d

Compound **2d** was prepared in 64% yield (63.0 mg) according to the general procedure (Table 2, entry 4). Yellow solid (mp 234-236 ℃). ^{1}H NMR (400 MHz, $CDCl_3$) δ 8.93 (d, *J* = 8.3 Hz, 1H), 8.22 (d, *J* = 8.4 Hz, 1H), 8.08 (d, *J* = 8.3 Hz, 2H), 7.85 – 7.67 (m, 6H), 7.37 (d, *J* = 8.1 Hz, 2H), 5.16 (s, 2H), 2.42 (s, 3H); ^{13}C NMR (125 MHz, $CDCl_3$) δ 165.6, 152.6, 148.3, 145.7, 136.5, 135.0, 133.5, 133.2, 132.4, 130.9, 130.0, 129.9, 129.8, 129.2, 128.3, 124.7, 123.2, 122.2, 49.5, 21.7; IR (neat): 3433, 1726, 1627, 1458, 1359, 1290,

1143, 1119, 1086, 767, 576; HRESIMS Calcd for $[C_{24}H_{17}BrN_2NaO_3S]^+$ (M + Na^+) 515.0035, found 515.0053.

4-(*p*-tolyl)-2-tosyl-2,3-dihydro-1*H*-pyrrolo[3,4-*c*]quinolin-1-one (2e)

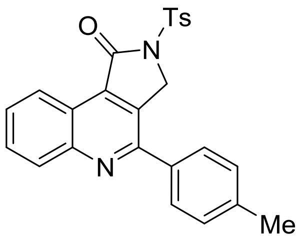

2e

Compound **2e** was prepared in 78% yield (66.8 mg) according to the general procedure (Table 2, entry 5). Yellow solid (mp 226-228 ℃). ^{1}H NMR (500 MHz, $CDCl_3$) δ 8.91 (d, *J* = 8.3 Hz, 1H), 8.22 (d, *J* = 8.4 Hz, 1H), 8.07 (d, *J* = 8.4 Hz, 2H), 7.80 – 7.76 (m, 3H), 7.66 (t, *J* = 7.6 Hz, 1H), 7.41 – 7.33 (m, 4H), 5.17 (s, 2H), 2.46 (s, 3H), 2.42 (s, 3H); ^{13}C NMR (125 MHz, $CDCl_3$) δ 165.8, 153.8, 148.3, 145.6, 140.3, 135.0, 134.8, 133.6, 133.2, 130.6, 129.9, 129.8, 129.7, 128.7, 128.3, 128.1, 123.1, 121.9, 49.7, 21.7, 21.4; IR (neat): 3460, 1731, 1631, 1365, 1336, 1172, 1146, 1123, 1091, 774, 661, 578; HRESIMS Calcd for $[C_{25}H_{20}N_2NaO_3S]^+$ (M + Na^+) 451.1087, found 451.1093.

4-(4-methoxyphenyl)-2-tosyl-2,3-dihydro-1*H*-pyrrolo[3,4-*c*]quinolin-1-one (2f)

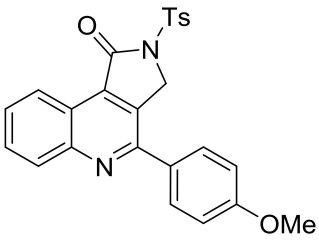

2f

Compound **2f** was prepared in 74% yield (65.7 mg) according to the general procedure (Table 2, entry 6). Yellow solid (mp 198-200 ℃). ^{1}H NMR (400 MHz, $CDCl_3$) δ 8.90 (d, *J* = 8.2 Hz, 1H), 8.21 (d, *J* = 8.4 Hz, 1H), 8.08 (d, *J* = 8.3 Hz, 2H), 7.86 (d, *J* = 8.7 Hz, 2H), 7.79 (t, *J* = 7.2 Hz, 1H), 7.65 (t, *J* = 7.6 Hz, 1H), 7.36 (d, *J* = 8.2 Hz, 2H), 7.08 (d, *J*

= 8.7 Hz, 2H), 5.18 (s, 2H), 3.91 (s, 3H), 2.42 (s, 3H); ^{13}C NMR (125 MHz, $CDCl_3$) δ 165.8, 161.1, 153.4, 148.3, 145.6, 135.1, 133.4, 133.2, 130.6, 130.1, 129.9, 129.7, 129.6, 128.5, 128.3, 123.1, 121.8, 114.6, 55.5, 49.8, 21.7; IR (neat): 3477, 2923, 2852, 2128, 1725, 1596, 1502, 1452, 1366, 1254, 1170, 1146, 1089, 660, 578; HRESIMS Calcd for $[C_{25}H_{20}N_2NaO_4S]^+$ (M + Na^+) 467.1036, found 467.1047.

4-(3-bromophenyl)-2-tosyl-2,3-dihydro-1*H*-pyrrolo[3,4-*c*]quinolin-1-one (2g)

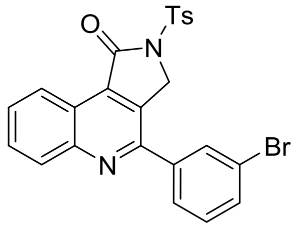

2g

Compound **2g** was prepared in 87% yield (85.6 mg) according to the general procedure (Table 2, entry 7). Yellow solid (mp 254-256 ℃). ^{1}H NMR (500 MHz, $CDCl_3$) δ 8.92 (d, *J* = 8.2 Hz, 1H), 8.23 (d, *J* = 8.3 Hz, 1H), 8.09 – 8.07 (m, 3H), 7.83 (t, *J* = 7.6 Hz, 1H), 7.75 (d, *J* = 7.3 Hz, 1H), 7.69 (dd, *J* = 15.8, 7.9 Hz, 2H), 7.45 (t, *J* = 7.7 Hz, 1H), 7.37 (d, *J* = 7.8 Hz, 2H), 5.16 (s, 2H), 2.42 (s, 3H); ^{13}C NMR (125 MHz, $CDCl_3$) δ 165.5, 152.2, 148.3, 145.7, 139.5, 134.9, 133.5, 133.2, 133.0, 131.5, 130.9, 130.5, 130.0, 129.9, 129.3, 128.3, 126.5, 123.5, 123.1, 122.2, 49.4, 21.7; IR (neat): 3473, 2919, 2359, 1720, 1589, 1466, 1356, 1329, 1173, 1150, 1093, 774, 585; HRESIMS Calcd for $[C_{24}H_{17}BrN_2NaO_3S]^+$ (M + Na^+) 515.0035, found 515.0050.

4-(*m*-tolyl)-2-tosyl-2,3-dihydro-1*H*-pyrrolo[3,4-*c*]quinolin-1-one (2h)

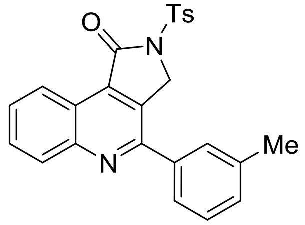

2h

Compound **2h** was prepared in 74% yield (63.4 mg) according to the general procedure (Table 2, entry 8). Yellow solid (mp 228-230 ℃). ^{1}H NMR (400 MHz, $CDCl_3$) δ 8.89 (d,

J = 9.1 Hz, 1H), 8.22 (d, *J* = 8.4 Hz, 1H), 8.07 (d, *J* = 8.4 Hz, 2H), 7.81 – 7.77 (m, 1H), 7.68 – 7.63 (m, 2H), 7.59 (d, *J* = 7.7 Hz, 1H), 7.44 (t, *J* = 7.6 Hz, 1H), 7.36 – 7.32 (m, 3H), 5.15 (s, 2H), 2.47 (s, 3H), 2.41 (s, 3H); ^{13}C NMR (125 MHz, $CDCl_3$) δ 165.7, 154.1, 148.2, 145.6, 139.0, 137.5, 135.0, 133.6, 133.1, 130.7, 130.6, 129.9, 129.8, 128.9, 128.8, 128.2, 125.1, 123.0, 122.0, 49.6, 21.6, 21.5; IR (neat): 2921, 2850, 1730, 1644, 1594, 1505, 1460, 1366, 1336, 1172, 1090, 772, 580; HRESIMS Calcd for $[C_{25}H_{20}N_2NaO_3S]^+$ (M + Na^+) 451.1087, found 451.1094.

8-methyl-4-phenyl-2-tosyl-2,3-dihydro-1*H*-pyrrolo[3,4-*c*]quinolin-1-one (2i)

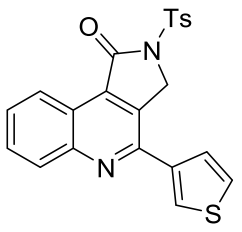

2i

Compound **2i** was prepared in 55% yield (46.2 mg) according to the general procedure (Table 2, entry 9). Yellow solid (mp 210-212 ℃). ^{1}H NMR (500 MHz, $CDCl_3$) δ 8.89 (d, *J* = 8.2 Hz, 1H), 8.19 (d, *J* = 8.3 Hz, 1H), 8.10 (d, *J* = 7.7 Hz, 2H), 7.93 (s, 1H), 7.86 (d, *J* = 4.0 Hz, 1H), 7.80 (t, *J* = 7.2 Hz, 1H), 7.66 (t, *J* = 7.2 Hz, 1H), 7.53 (s, 1H), 7.38 (d, *J* = 7.6 Hz, 2H), 5.24 (s, 2H), 2.43 (s, 3H); ^{13}C NMR (125 MHz, DMSO) δ 165.5, 149.1, 147.0, 145.4, 139.3, 135.1, 134.9, 132.0, 130.6, 129.8, 129.4, 128.8, 128.2, 127.8, 127.7, 127.2, 122.1, 121.2, 50.1, 21.1; IR (neat): 3423, 2254, 2127, 1650, 1366, 1232, 1166, 1015, 825, 763, 629; HRESIMS Calcd for $[C_{22}H_{16}N_2NaO_3S_2]^+$ (M + Na^+) 443.0495, found 443.0499.

8-methyl-4-phenyl-2-tosyl-2,3-dihydro-1*H*-pyrrolo[3,4-*c*]quinolin-1-one (2k)

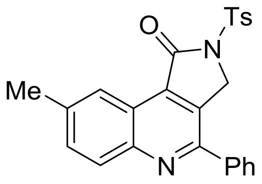

2k

Compound **2k** was prepared in 70% yield (59.9 mg) according to the general procedure (Table 2, entry 11). Yellow solid (mp 233-235 ℃). ^{1}H NMR (400 MHz, $CDCl_3$) δ 8.70 (s, 1H), 8.12 (d, *J* = 8.6 Hz, 1H), 8.07 (d, *J* = 8.3 Hz, 2H), 7.85 (d, *J* = 6.7 Hz, 2H), 7.63 (dd, *J* = 8.6, 1.5 Hz, 1H), 7.59 – 7.51 (m, 3H), 7.36 (d, *J* = 8.2 Hz, 2H), 5.16 (s, 2H), 2.57 (s, 3H), 2.42 (s, 3H); ^{13}C NMR (125 MHz, $CDCl_3$) δ 166.0, 152.8, 147.0, 145.6, 139.6, 137.7, 135.1, 133.5, 132.9, 132.4, 129.9, 129.8, 129.5, 129.1, 128.3, 128.2, 122.1, 122.0, 49.6, 22.0, 21.7; IR (neat): 3463, 2921, 1729, 1594, 1446, 1365, 1290, 1202, 1170, 1141, 1088, 823, 666, 544; HRESIMS Calcd for $[C_{25}H_{20}N_2NaO_3S]^+$ $(M + Na^+)$ 451.1087, found 451.1094.

8-fluoro-4-phenyl-2-tosyl-2,3-dihydro-1*H*-pyrrolo[3,4-*c*]quinolin-1-one (2l)

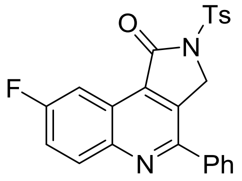

2l

Compound **2l** was prepared in 72% yield (62.2 mg) according to the general procedure (Table 2, entry 12). Yellow solid (mp 265-267 ℃). ^{1}H NMR (400 MHz, $CDCl_3$) δ 8.56 (dd, *J* = 9.1, 2.8 Hz, 1H), 8.24 (dd, *J* = 9.2, 5.3 Hz, 1H), 8.07 (d, *J* = 8.3 Hz, 2H), 7.86 (d, *J* = 6.5 Hz, 2H), 7.63 – 7.52 (m, 4H), 7.37 (d, *J* = 8.2 Hz, 2H), 5.19 (s, 2H), 2.43 (s, 3H); ^{13}C NMR (125 MHz, $CDCl_3$) δ 165.5, 162.1 (d, *J* = 251.3 Hz), 153.2, 145.8, 145.5, 137.3, 134.9, 134.3, 133.0 (d, *J* = 5.0 Hz), 132.4 (d, *J* = 10.0 Hz), 130.1, 130.0, 129.2, 128.3, 128.2, 122.9 (d, *J* = 11.3 Hz), 121.0 (d, *J* = 26.3 Hz), 107.3 (d, *J* = 23.8 Hz), 49.6, 21.7; IR (neat): 3461, 2117, 1745, 1596, 1446, 1369, 1237, 1169, 1141, 1087, 1004, 836, 666, 578; HRESIMS Calcd for $[C_{24}H_{17}FN_2NaO_3S]^+$ $(M + Na^+)$ 455.0836, found 455.0846.

8-chloro-4-phenyl-2-tosyl-2,3-dihydro-1*H*-pyrrolo[3,4-*c*]quinolin-1-one (2m)

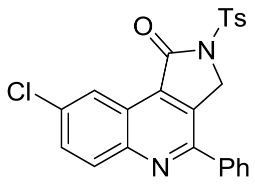

2m

Compound **2m** was prepared in 68% yield (60.9 mg) according to the general procedure (Table 2, entry 13). Yellow solid (mp 203-205 ℃). ^{1}H NMR (400 MHz, $CDCl_3$) δ 8.94 (d, *J* = 2.1 Hz, 1H), 8.17 (d, *J* = 9.0 Hz, 1H), 8.08 (d, *J* = 8.3 Hz, 2H), 7.87 (d, *J* = 6.3 Hz, 2H), 7.75 (dd, *J* = 9.0, 2.3 Hz, 1H), 7.63 – 7.54 (m, 3H), 7.38 (d, *J* = 8.1 Hz, 2H), 5.20 (s, 2H), 2.43 (s, 3H); ^{13}C NMR (125 MHz, $CDCl_3$) δ 165.4, 154.1, 146.7, 145.8, 137.3, 135.4, 134.9, 134.4, 132.7, 131.8, 131.3, 130.3, 130.0, 129.3, 128.4, 128.2, 122.6, 122.3, 49.7, 21.7; IR (neat): 3471, 2920, 2125, 1721, 1658, 1620, 1484, 1449, 1365, 1142, 1075, 916, 659, 541; HRESIMS Calcd for $[C_{24}H_{17}ClN_2NaO_3S]^+$ (M + Na^+) 471.0541, found 471.0556.

8-bromo-4-phenyl-2-tosyl-2,3-dihydro-1*H*-pyrrolo[3,4-*c*]quinolin-1-one (2n)

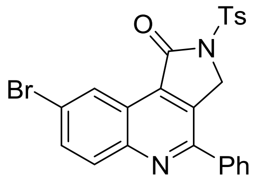

2n

Compound **2n** was prepared in 63% yield (62.0 mg) according to the general procedure (Table 2, entry 14). Yellow solid (mp 258-260 ℃). ^{1}H NMR (400 MHz, $CDCl_3$) δ 9.11 (d, *J* = 2.2 Hz, 1H), 8.11 – 8.06 (m, 3H), 7.92 – 7.85 (m, 3H), 7.63 – 7.55 (m, 3H), 7.38 (d, *J* = 8.3 Hz, 2H), 5.20 (s, 2H), 2.43 (s, 3H); ^{13}C NMR (125 MHz, $CDCl_3$) δ 165.3, 154.2, 146.9, 145.8, 137.3, 134.9, 134.4, 134.3, 132.5, 131.4, 130.3, 130.0, 129.3, 128.4, 128.2, 125.6, 123.7, 122.9, 49.7, 21.7; IR (neat): 3441, 2917, 1738, 1633, 1592, 1491, 1370, 1170, 1148, 1087, 996, 703, 543; HRESIMS Calcd for $[C_{24}H_{17}BrN_2NaO_3S]^+$ (M + Na^+) 515.0035, found 515.0053.

4-phenyl-2-(phenylsulfonyl)-2,3-dihydro-1*H*-pyrrolo[3,4-*c*]quinolin-1-one (2o)

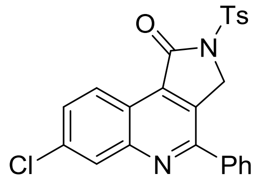

2o

Compound **2o** was prepared in 94% yield (84.2 mg) according to the general procedure (Table 2, entry 15). Yellow solid (mp 278-280 ℃). ^{1}H NMR (500 MHz, $CDCl_3$) δ 8.86 (d, *J* = 8.9 Hz, 1H), 8.23 (d, *J* = 1.6 Hz, 1H), 8.07 (d, *J* = 8.3 Hz, 2H), 7.87 (d, *J* = 7.8 Hz, 2H), 7.65 – 7.55 (m, 4H), 7.37 (d, *J* = 8.2 Hz, 2H), 5.18 (s, 2H), 2.42 (s, 3H); ^{13}C NMR (125 MHz, $CDCl_3$) δ 165.4, 155.0, 148.8, 145.8, 137.2, 136.8, 134.9, 133.6, 133.5, 130.3, 130.0, 129.8, 129.2, 128.9, 128.3, 128.2, 124.4, 120.4, 49.7, 21.7; IR (neat): 3476, 3355, 2919, 2849, 1731, 1658, 1631, 1469, 1364, 1131, 1075, 666, 544; HRESIMS Calcd for $[C_{24}H_{17}ClN_2NaO_3S]^+$ (M + Na$^+$) 471.0541, found 471.0552.

4-phenyl-2-(phenylsulfonyl)-2,3-dihydro-1*H*-pyrrolo[3,4-*c*]quinolin-1-one (2p)

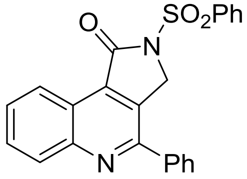

2p

Compound **2p** was prepared in 77% yield (61.6 mg) according to the general procedure (Table 2, entry 16). Yellow solid (mp 247-249 ℃). ^{1}H NMR (400 MHz, $CDCl_3$) δ 8.93 (d, *J* = 8.2 Hz, 1H), 8.25 (d, *J* = 8.5 Hz, 1H), 8.21 (d, *J* = 7.6 Hz, 2H), 7.87 (d, *J* = 6.5 Hz, 2H), 7.83 (t, *J* = 7.3 Hz, 1H), 7.74 – 7.65 (m, 2H), 7.63 – 7.52 (m, 5H), 5.20 (s, 2H); ^{13}C NMR (125 MHz, $CDCl_3$) δ 165.8, 153.9, 148.4, 138.0, 137.6, 134.4, 133.6, 133.2, 130.7, 130.0, 129.9, 129.3, 129.2, 129.0, 128.2, 128.1, 123.1, 122.1, 49.6; IR (neat): 3458, 2920, 1721, 1511, 1445, 1357, 1170, 1122, 1087, 985, 771, 562; HRESIMS Calcd for $[C_{23}H_{16}N_2NaO_3S]^+$ (M + Na$^+$) 423.0774, found 423.0779.

2-((4-bromophenyl)sulfonyl)-4-phenyl-2,3-dihydro-1*H*-pyrrolo[3,4-*c*]quinolin-1-one (2q)

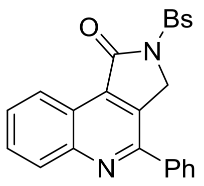

2q

Compound **2q** was prepared in 68% yield (65.0 mg) according to the general procedure (Table 2, entry 17). Yellow solid (mp 280-282 ℃). ^{1}H NMR (400 MHz, $CDCl_3$) δ 8.91 (d, *J* = 8.3 Hz, 1H), 8.26 (d, *J* = 8.4 Hz, 1H), 8.07 (d, *J* = 8.8 Hz, 2H), 7.90 – 7.81 (m, 3H), 7.76 – 7.67 (m, 3H), 7.64 – 7.52 (m, 3H), 5.19 (s, 2H); ^{13}C NMR (125 MHz, $CDCl_3$) δ 165.8, 153.9, 148.4, 137.6, 136.9, 133.6, 133.0, 132.6, 130.8, 130.1, 130.0, 129.9, 129.8, 129.2, 129.1, 128.2, 123.0, 122.0, 49.6; IR (neat): 3092, 2922, 1753, 1573, 1372, 1333, 1287, 1167, 1143, 1085, 1008, 772, 562; HRESIMS Calcd for $[C_{23}H_{15}BrN_2NaO_3S]^+$ (M + Na^+) 500.9879, found 500.9891.

2-(methylsulfonyl)-4-phenyl-2,3-dihydro-1*H*-pyrrolo[3,4-*c*]quinolin-1-one (2r)

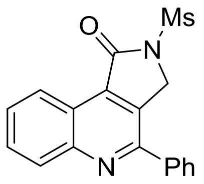

2r

Compound **2r** was prepared in 85% yield (73.1 mg) according to the general procedure (Table 2, entry 18). Pale yellow oil. ^{1}H NMR (500 MHz, $CDCl_3$) δ 8.96 (d, *J* = 8.2 Hz, 1H), 8.28 (d, *J* = 8.5 Hz, 1H), 7.90 – 7.82 (m, 3H), 7.74 (t, *J* = 7.3 Hz, 1H), 7.61 – 7.51 (m, 3H), 5.14 (s, 2H), 3.48 (s, 3H); ^{13}C NMR (125 MHz, $CDCl_3$) δ 166.9, 154.0, 148.4, 137.5, 133.7, 133.0, 130.8, 130.1, 130.0, 129.2, 129.1, 128.2, 123.0, 122.0, 49.0, 41.2; IR (neat): 3475, 2918, 2300, 1732, 1620, 1509, 1457, 1345, 1143, 1122, 1075, 973, 772, 522; HRESIMS Calcd for $[C_{18}H_{14}N_2NaO_3S]^+$ (M + Na^+) 361.0617, found 361.0622.

(*S*)-4-phenyl-3-propyl-2-tosyl-2,3-dihydro-1*H*-pyrrolo[3,4-*c*]quinolin-1-one (2s)

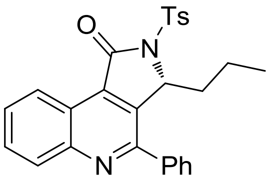

2s

Compound **2s** was prepared in 83% yield (75.2 mg) according to the general procedure (eq 1). Pale yellow oil. $[\alpha]_D^{20}$ = +84.6 °(c = 1.0, $CHCl_3$). 98% ee (determined by HPLC: Chiralcel IC Column, 10/90 *i*-PrOH/hexane, 1.0 mL/min, 254 nm; TR = 33.32 min (major), 26.31 min (minor)). ^{1}H NMR (400 MHz, $CDCl_3$) δ 8.97 (d, *J* = 7.9 Hz, 1H), 8.24 (d, *J* = 8.4 Hz, 1H), 8.10 (d, *J* = 8.3 Hz, 2H), 7.82 (t, *J* = 7.1 Hz, 1H), 7.78 – 7.74 (m, 2H), 7.70 (t, *J* = 7.3 Hz, 1H), 7.63 – 7.52 (m, 3H), 7.37 (d, *J* = 8.2 Hz, 2H), 5.95 (t, *J* = 3.4 Hz, 1H), 2.44 (s, 3H), 2.31 – 2.20 (m, 1H), 1.51 – 1.40 (m, 1H), 0.57 – 0.47 (m, 1H), 0.40 (t, *J* = 7.1 Hz, 3H), 0.20 – 0.07 (m, 1H); ^{13}C NMR (125 MHz, $CDCl_3$) δ 166.3, 154.7, 148.2, 145.4, 138.4, 137.7, 135.8, 132.9, 130.6, 129.9, 129.8, 129.7, 129.2, 129.0, 128.2, 128.1, 123.4, 121.9, 61.9, 31.2, 21.7, 14.9, 13.2; IR (neat): 3451, 2960, 2872, 1727, 1623, 1593, 1494, 1358, 1286, 1172, 1153, 1089, 1050, 777, 662; HRESIMS Calcd for $[C_{27}H_{24}N_2NaO_3S]^+$ (M + Na^+) 479.1400, found 479.1408.

(*S*)-3-benzyl-4-phenyl-2-tosyl-2,3-dihydro-1*H*-pyrrolo[3,4-*c*]quinolin-1-one (2t)

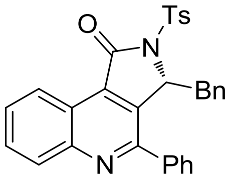

2t

Compound **2t** was prepared in 67% yield (67.2 mg) according to the general procedure (eq 1). Pale yellow oil. $[\alpha]_D^{20}$ = -164.8 °(c = 1.0, $CHCl_3$). 98% ee (determined by HPLC: Chiralcel AS-H Column, 10/90 *i*-PrOH/hexane, 1.0 mL/min, 254 nm; TR = 29.47 min (major), 21.27 min (minor)). ^{1}H NMR (400 MHz, $CDCl_3$) δ 8.68 (dd, *J* = 8.4, 0.8 Hz, 1H), 8.15 (t, *J* = 8.3 Hz, 3H), 7.98 – 7.93 (m, 2H), 7.76 – 7.54 (m, 5H), 7.40 (d, *J* = 8.1 Hz,

2H), 6.86 – 6.80 (m, 3H), 6.52 (dd, *J* = 7.8, 1.6 Hz, 2H), 6.16 (t, 1H), 3.65 (dd, *J* = 14.5, 4.2 Hz, 1H), 2.89 (dd, *J* = 14.5, 3.1 Hz, 1H), 2.45 (s, 3H); ^{13}C NMR (125 MHz, $CDCl_3$) δ 166.0, 154.4, 148.1, 145.6, 138.6, 137.0, 135.6, 133.1, 132.7, 130.5, 130.0, 129.9, 129.7, 129.6, 129.5, 128.8, 128.6, 128.5, 127.8, 126.8, 123.2, 121.5, 62.1, 36.2, 21.7; IR (neat): 3444, 3062, 2923, 2125, 1729, 1596, 1493, 1454, 1359, 1299, 1241, 1171, 1089, 704, 662, 577; HRESIMS Calcd for $[C_{31}H_{24}N_2NaO_3S]^+$ (M + Na^+) 527.1400, found 527.1407.

8-(morpholinosulfonyl)-4-phenyl-2-tosyl-2,3-dihydro-1*H*-pyrrolo[3,4-*c*]quinolin-1-one (2u)

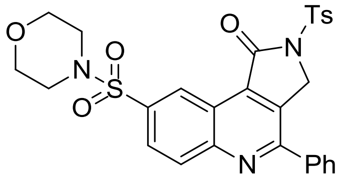

2u

Compound **2u** was prepared in 67% yield (75.6 mg) according to the general procedure (Scheme 2). Yellow solid (mp 275-277 ℃). ^{1}H NMR (500 MHz, $CDCl_3$) δ 9.36 (d, *J* = 1.9 Hz, 1H), 8.39 (d, *J* = 8.8 Hz, 1H), 8.13 – 8.06 (m, 3H), 7.94 – 7.90 (m, 2H), 7.65 – 7.60 (m, 3H), 7.39 (d, *J* = 8.2 Hz, 2H), 5.25 (s, 2H), 3.76 (t, 4H), 3.10 (t, 4H), 2.44 (s, 3H); ^{13}C NMR (125 MHz, $CDCl_3$) δ 164.8, 156.8, 149.3, 146.0, 136.9, 135.5, 135.1, 134.7, 134.5, 131.1, 130.8, 130.1, 129.4, 128.4, 128.3, 128.2, 124.1, 121.3, 66.1, 49.8, 46.1, 21.7; IR (neat): 3434, 2923, 2359, 1726, 1658, 1589, 1450, 1332, 1173, 1089, 952, 774, 544; HRESIMS Calcd for $[C_{28}H_{25}N_3NaO_6S_2]^+$ (M + Na^+) 586.1077, found 586.1085.

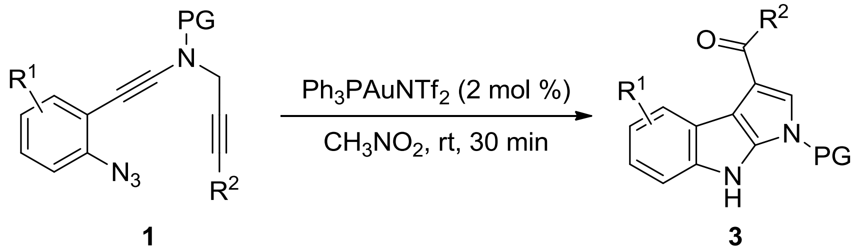

Supplementary Figure 90. Synthesis of pyrrolo[2,3-*b*]indoles **3**.

General procedure for the synthesis of pyrrolo[2,3-*b*]indoles 3:

$Ph_3PAuNTf_2$ (0.004 mmol, 3.0 mg) were added in this order to the ynamide **1** (0.20 mmol) in CH_3NO_2 (4.0 mL) at room temperature. The reaction mixture was stirred at room temperature and the progress of the reaction was monitored by TLC. The reaction typically took 30 min. Upon completion, the mixture was then concentrated and the residue was purified by chromatography on silica gel (eluent: hexanes/ ethyl acetate) to afford the desired product **3**.

phenyl(1-tosyl-1,8-dihydropyrrolo[2,3-*b*]indol-3-yl)methanone (3a)

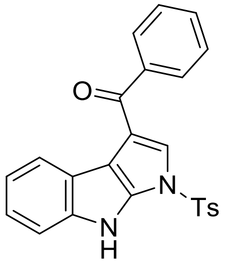

3a

Compound **3a** was prepared in 88% yield (72.8 mg) according to the general procedure (Table 3, entry 1). Yellow solid (mp 182-184 ℃). ^{1}H NMR (500 MHz, $CDCl_3$) δ 8.82 (s, 1H), 8.02 (d, *J* = 7.9 Hz, 1H), 7.90 (d, *J* = 7.5 Hz, 2H), 7.79 (d, *J* = 8.3 Hz, 2H), 7.61 (t, *J* = 7.4 Hz, 1H), 7.51 (t, *J* = 7.6 Hz, 2H), 7.46 (d, *J* = 8.1 Hz, 1H), 7.29 (s, 1H), 7.26 – 7.24 (m, 3H), 7.20 (t, *J* = 7.5 Hz, 1H), 2.36 (s, 3H); ^{13}C NMR (125 MHz, $CDCl_3$) δ 190.5, 146.3, 139.3, 138.8, 136.3, 134.5, 132.3, 130.4, 129.1, 128.6, 126.9, 123.9, 122.9, 122.8, 121.6, 121.0, 120.9, 111.8, 109.1, 21.7; IR (neat): 3395 (br), 2359, 1731, 1638, 1500, 1483, 1378, 1292, 1176, 1142, 1006, 922, 670; HRESIMS Calcd for $[C_{24}H_{18}N_2NaO_3S]^+$ (M + Na^+) 437.0930, found 437.0937.

(4-fluorophenyl)(1-tosyl-1,8-dihydropyrrolo[2,3-*b*]indol-3-yl)methanone (3b)

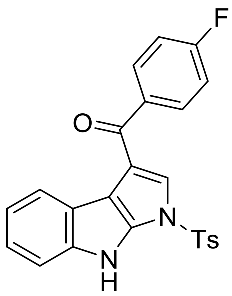

3b

Compound **3b** was prepared in 80% yield (69.1 mg) according to the general procedure (Table 3, entry 2). Pale yellow oil. ^{1}H NMR (500 MHz, $CDCl_3$) δ 9.05 (s, 1H), 7.96 – 7.92 (m, 3H), 7.78 (d, *J* = 8.3 Hz, 2H), 7.45 (d, *J* = 8.1 Hz, 1H), 7.30 (s, 1H), 7.26 – 7.15 (m, 6H), 2.33 (s, 3H); ^{13}C NMR (125 MHz, $CDCl_3$) δ 189.0, 165.3 (d, *J* = 252.5 Hz), 146.4, 139.3, 136.3, 134.9 (d, *J* = 3.8 Hz), 134.3, 131.6 (d, *J* = 10.0 Hz), 130.4, 126.8, 123.6, 122.9, 122.6, 121.4, 120.9, 120.7, 115.7 (d, *J* = 21.3 Hz), 111.9, 109.0, 21.6; IR (neat): 3364 (br), 2924, 1731, 1639, 1598, 1451, 1378, 1230, 1175, 1110, 1007, 925, 671, 544; HRESIMS Calcd for $[C_{24}H_{17}FN_2NaO_3S]^+$ (M + H^+) 455.0836, found 455.0842.

(4-chlorophenyl)(1-tosyl-1,8-dihydropyrrolo[2,3-*b*]indol-3-yl)methanone (3c)

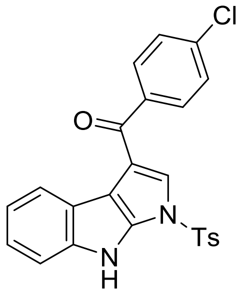

3c

Compound **3c** was prepared in 83% yield (74.4 mg) according to the general procedure (Table 3, entry 3). Pale yellow oil. ^{1}H NMR (500 MHz, $CDCl_3$) δ 9.09 (s, 1H), 7.99 (d, *J* = 7.8 Hz, 1H), 7.83 (d, *J* = 8.3 Hz, 2H), 7.77 (d, *J* = 8.3 Hz, 2H), 7.45 (dd, *J* = 8.4, 3.0 Hz, 3H), 7.29 (s, 1H), 7.23 (t, *J* = 3.9 Hz, 1H), 7.18 (t, *J* = 8.8 Hz, 3H), 2.32 (s, 3H); ^{13}C NMR (125 MHz, $CDCl_3$) δ 189.2, 146.4, 139.3, 138.6, 136.9, 136.3, 134.3, 130.4, 130.3, 128.8, 126.8, 123.7, 122.9, 122.5, 121.4, 120.9, 120.7, 111.9, 108.9, 21.6; IR (neat): 3324

(br), 3134, 2925, 1734, 1639, 1588, 1501, 1484, 1451, 1376, 1293, 1243, 1176, 1006, 924, 671; HRESIMS Calcd for $[C_{24}H_{17}ClN_2NaO_3S]^+$ (M + Na$^+$) 471.0541, found 471.0552.

(4-bromophenyl)(1-tosyl-1,8-dihydropyrrolo[2,3-*b*]indol-3-yl)methanone (3d)

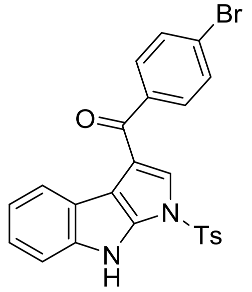

3d

Compound **3d** was prepared in 77% yield (75.8 mg) according to the general procedure (Table 3, entry 4). Yellow solid (mp 188-190 ℃). ^{1}H NMR (500 MHz, $CDCl_3$) δ 9.00 (s, 1H), 8.00 (d, *J* = 7.8 Hz, 1H), 7.79 – 7.75 (m, 4H), 7.62 (d, *J* = 8.3 Hz, 2H), 7.45 (d, *J* = 8.1 Hz, 1H), 7.27 – 7.18 (m, 5H), 2.34 (s, 3H); ^{13}C NMR (125 MHz, $CDCl_3$) δ 189.3, 146.4, 139.3, 137.4, 136.3, 134.3, 131.8, 130.5, 130.4, 127.2, 126.8, 123.8, 122.9, 122.5, 121.5, 120.9, 120.7, 111.9, 108.9, 21.6; IR (neat): 3387 (br), 2923, 2852, 1723, 1637, 1585, 1500, 1451, 1378, 1293, 1176, 1144, 1006, 923, 670; HRESIMS Calcd for $[C_{24}H_{17}BrN_2NaO_3S]^+$ (M + Na$^+$) 515.0035, found 515.0056.

***p*-tolyl(1-tosyl-1,8-dihydropyrrolo[2,3-*b*]indol-3-yl)methanone (3e)**

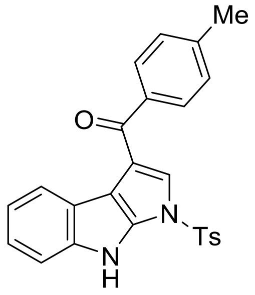

3e

Compound **3e** was prepared in 76% yield (65.1 mg) according to the general procedure (Table 3, entry 5). Pale yellow oil. ^{1}H NMR (500 MHz, $CDCl_3$) δ 9.15 (s, 1H), 8.02 (d, *J*

= 7.8 Hz, 1H), 7.82 (d, *J* = 8.0 Hz, 2H), 7.76 (d, *J* = 8.3 Hz, 2H), 7.43 (d, *J* = 8.1 Hz, 1H), 7.33 (s, 1H), 7.28 (d, *J* = 7.9 Hz, 2H), 7.22 (t, *J* = 7.5 Hz, 1H), 7.18 – 7.14 (m, 3H), 2.43 (s, 3H), 2.29 (s, 3H); ^{13}C NMR (125 MHz, $CDCl_3$) δ 190.3, 146.2, 143.0, 139.3, 136.3, 136.0, 134.3, 130.3, 129.2, 129.1, 126.8, 123.7, 122.9, 122.7, 121.5, 120.8, 120.7, 111.9, 109.1, 21.6, 21.5; IR (neat): 3305 (br), 3137, 2922, 1734, 1605, 1497, 1378, 1299, 1176, 1110, 1007, 925, 740, 671; HRESIMS Calcd for $[C_{25}H_{20}N_2NaO_3S]^+$ (M + Na$^+$) 451.1087, found 451.1099.

(4-methoxyphenyl)(1-tosyl-1,8-dihydropyrrolo[2,3-*b*]indol-3-yl)methanone (3f)

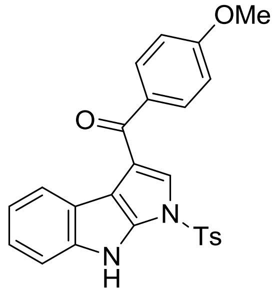

3f

Compound **3f** was prepared in 63% yield (55.9 mg) according to the general procedure (Table 3, entry 6). Pale yellow oil. ^{1}H NMR (500 MHz, $CDCl_3$) δ 8.86 (s, 1H), 7.96 – 7.92 (m, 3H), 7.78 (d, *J* = 8.4 Hz, 2H), 7.45 (d, *J* = 8.1 Hz, 1H), 7.29 (s, 1H), 7.28 – 7.24 (m, 3H), 7.19 (t, *J* = 7.3 Hz, 1H), 6.99 (d, *J* = 8.8 Hz, 2H), 3.90 (s, 3H), 2.36 (s, 3H); ^{13}C NMR (125 MHz, $CDCl_3$) δ 189.1, 163.1, 146.3, 139.3, 136.3, 134.5, 131.4, 131.3, 130.4, 126.8, 123.1, 123.0, 122.8, 121.5, 120.9, 120.8, 113.8, 111.8, 109.3, 55.5, 21.6; IR (neat): 3394 (br), 2923, 2851, 1598, 1498, 1452, 1378, 1259, 1176, 1164, 1110, 1085, 925, 671; HRESIMS Calcd for $[C_{25}H_{20}N_2NaO_4S]^+$ (M + Na$^+$) 467.1036, found 467.1047.

(3-bromophenyl)(1-tosyl-1,8-dihydropyrrolo[2,3-*b*]indol-3-yl)methanone (3g)

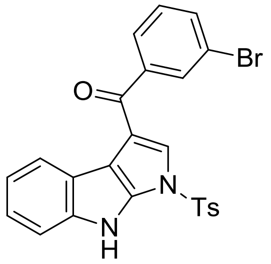

3g

Compound **3g** was prepared in 82% yield (80.7 mg) according to the general procedure (Table 3, entry 7). Pale yellow oil. ^{1}H NMR (500 MHz, $CDCl_3$) δ 8.76 (s, 1H), 8.01 – 7.97 (m, 2H), 7.81 – 7.78 (m, 3H), 7.73 (d, *J* = 8.0 Hz, 1H), 7.46 (d, *J* = 8.1 Hz, 1H), 7.39 (t, *J* = 7.8 Hz, 1H), 7.31 – 7.26 (m, 4H), 7.22 (t, *J* = 7.5 Hz, 1H), 2.38 (s, 3H); ^{13}C NMR (125 MHz, $CDCl_3$) δ 188.7, 146.5, 140.6, 139.3, 136.4, 135.1, 134.5, 131.9, 130.5, 130.1, 127.6, 126.9, 124.0, 123.1, 122.8, 122.6, 121.6, 121.1, 120.8, 111.9, 108.9, 21.7; IR (neat): 3409 (br), 2922, 1731, 1640, 1500, 1451, 1379, 1294, 1175, 1143, 1110, 1008, 934, 670; HRESIMS Calcd for $[C_{24}H_{17}BrN_2NaO_3S]^+$ (M + Na^+) 515.0035, found 515.0049.

***m*-tolyl(1-tosyl-1,8-dihydropyrrolo[2,3-*b*]indol-3-yl)methanone (3h)**

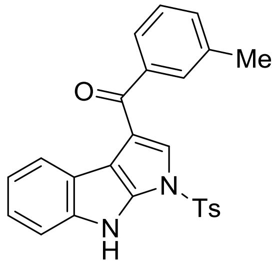

3h

Compound **3h** was prepared in 95% yield (81.3 mg) according to the general procedure (Table 3, entry 8). Pale yellow oil. ^{1}H NMR (500 MHz, $CDCl_3$) δ 8.91 (s, 1H), 8.01 (d, *J* = 7.8 Hz, 1H), 7.78 (d, *J* = 8.3 Hz, 2H), 7.75 – 7.69 (m, 2H), 7.45 – 7.36 (m, 3H), 7.30 (s, 1H), 7.27 – 7.17 (m, 4H), 2.42 (s, 3H), 2.34 (s, 3H); ^{13}C NMR (125 MHz, $CDCl_3$) δ 190.6, 146.3, 139.3, 138.8, 138.4, 136.3, 134.5, 133.0, 130.3, 129.5, 128.4, 126.8, 126.3, 123.9, 123.0, 122.8, 121.6, 120.9, 120.8, 111.8, 109.1, 21.6, 21.4; IR (neat): 3403 (br),

2924, 2854, 1633, 1598, 1499, 1451, 1378, 1298, 1234, 1177, 1159, 1109, 1085, 942, 734, 671; HRESIMS Calcd for $[C_{25}H_{20}N_2NaO_3S]^+$ (M + Na^+) 451.1087, found 451.1095.

thiophen-3-yl(1-tosyl-1,8-dihydropyrrolo[2,3-*b*]indol-3-yl)methanone (3i)

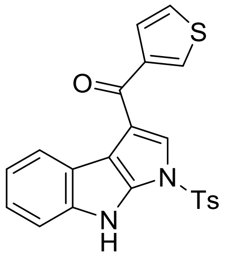

3i

Compound **3i** was prepared in 73% yield (61.3 mg) according to the general procedure (Table 3, entry 9). Pale yellow oil. ^{1}H NMR (500 MHz, $CDCl_3$) δ 8.91 (s, 1H), 8.05 (dd, *J* = 3.6, 2.6 Hz, 2H), 7.79 (d, *J* = 8.3 Hz, 2H), 7.63 (d, *J* = 5.0 Hz, 1H), 7.45 (d, *J* = 8.0 Hz, 1H), 7.41 – 7.39 (m, 2H), 7.27 – 7.18 (m, 4H), 2.34 (s, 3H); ^{13}C NMR (125 MHz, $CDCl_3$) δ 183.6, 146.4, 142.0, 139.3, 136.3, 134.5, 132.0, 130.4, 127.9, 126.8, 126.6, 123.7, 122.9, 122.7, 121.6, 121.0, 120.9, 111.8, 109.1, 21.7; IR (neat): 3394 (br), 2922, 2851, 1620, 1509, 1451, 1378, 1287, 1176, 1109, 1009, 894, 670; HRESIMS Calcd for $[C_{22}H_{16}N_2NaO_3S_2]^+$ (M + Na^+) 443.0495, found 443.0498.

1-(1-tosyl-1,8-dihydropyrrolo[2,3-*b*]indol-3-yl)ethan-1-one (3j)

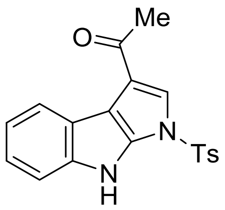

3j

Compound **3j** was prepared in 86% yield (60.6 mg) according to the general procedure (Table 3, entry 10). Pale yellow oil. ^{1}H NMR (400 MHz, $CDCl_3$) δ 8.75 (s, 1H), 8.23 (d, *J* = 6.9 Hz, 1H), 7.82 (d, *J* = 8.4 Hz, 2H), 7.46 – 7.42 (m, 2H), 7.30 – 7.21 (m, 4H), 2.53 (s, 3H), 2.38 (s, 3H); ^{13}C NMR (100 MHz, $CDCl_3$) δ 193.0, 146.3, 139.2, 136.3, 134.6, 130.4, 126.8, 124.6, 122.9, 122.4, 121.8, 121.0, 111.7, 108.2, 27.0, 21.7; IR (neat): 3314

(br), 2924, 1731, 1658, 1503, 1451, 1376, 1288, 1176, 1090, 952, 672, 599; HRESIMS Calcd for $[C_{19}H_{16}N_2NaO_3S]^+$ (M + Na^+) 375.0774, found 375.0778.

(5-methyl-1-tosyl-1,8-dihydropyrrolo[2,3-*b*]indol-3-yl)(phenyl)methanone (3k)

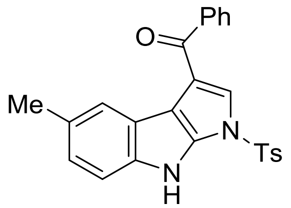

3k

Compound **3k** was prepared in 75% yield (64.2 mg) according to the general procedure (Table 3, entry 11). Pale yellow oil. ^{1}H NMR (400 MHz, $CDCl_3$) δ 8.73 (s, 1H), 7.89 (d, *J* = 7.3 Hz, 2H), 7.84 (s, 1H), 7.76 (d, *J* = 8.3 Hz, 2H), 7.60 (t, *J* = 7.4 Hz, 1H), 7.50 (t, *J* = 7.6 Hz, 2H), 7.32 (d, *J* = 8.3 Hz, 1H), 7.27 (s, 1H), 7.26 – 7.21 (m, 2H), 7.08 (d, *J* = 7.8 Hz, 1H), 2.43 (s, 3H), 2.34 (s, 3H); ^{13}C NMR (100 MHz, $CDCl_3$) δ 190.5, 146.2, 138.8, 137.6, 136.6, 134.5, 132.2, 130.3, 129.0, 128.5, 126.8, 124.2, 123.8, 122.9, 121.5, 121.1, 111.5, 108.9, 21.6, 21.4; IR (neat): 3345 (br), 2922, 1732, 1639, 1596, 1538, 1498, 1455, 1375, 1292, 1176, 1157, 1111, 1086, 941, 800, 670; HRESIMS Calcd for $[C_{25}H_{20}N_2NaO_3S]^+$ (M + Na^+) 451.1087, found 451.1094.

(5-fluoro-1-tosyl-1,8-dihydropyrrolo[2,3-*b*]indol-3-yl)(phenyl)methanone (3l)

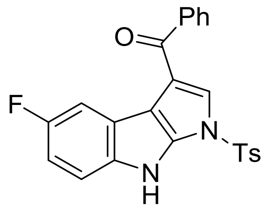

3l

Compound **3l** was prepared in 73% yield (63.1 mg) according to the general procedure (Table 3, entry 12). Pale yellow oil. ^{1}H NMR (400 MHz, $CDCl_3$) δ 9.05 (s, 1H), 7.88 (d, *J* = 7.6 Hz, 2H), 7.78 (d, *J* = 7.9 Hz, 2H), 7.71 (d, *J* = 9.3 Hz, 1H), 7.59 (t, *J* = 7.2 Hz, 1H), 7.49 (t, *J* = 7.5 Hz, 2H), 7.35 – 7.32 (m, 2H), 7.24 – 7.21 (m, 2H), 6.95 (t, *J* = 9.0 Hz, 1H), 2.33 (s, 3H); ^{13}C NMR (125 MHz, $CDCl_3$) δ 190.4, 158.0 (d, *J* = 233.8 Hz), 146.5,

138.5, 137.4, 135.6, 134.2, 132.4, 130.4, 128.9, 128.6, 126.8, 124.1, 122.6, 121.3 (d, *J* = 11.3 Hz), 112.4 (d, *J* = 10.0 Hz), 110.6 (d, *J* = 25.0 Hz), 109.1 (d, *J* = 3.8 Hz), 107.2 (d, *J* = 25.0 Hz), 21.6; IR (neat): 3332 (br), 2925, 1732, 1629, 1596, 1503, 1483, 1455, 1377, 1286, 1176, 1153, 1113, 1085, 945, 670, 543; HRESIMS Calcd for $[C_{24}H_{17}FN_2NaO_3S]^+$ (M + Na^+) 455.0836, found 455.0841.

(5-chloro-1-tosyl-1,8-dihydropyrrolo[2,3-*b*]indol-3-yl)(phenyl)methanone (3m)

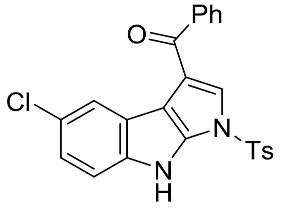

3m

Compound **3m** was prepared in 74% yield (66.3 mg) according to the general procedure (Table 3, entry 13). Pale yellow oil. ^{1}H NMR (400 MHz, $CDCl_3$) δ 8.84 (s, 1H), 8.06 (s, 1H), 7.88 (d, *J* = 7.5 Hz, 2H), 7.79 (d, *J* = 7.8 Hz, 2H), 7.62 (t, *J* = 7.3 Hz, 1H), 7.52 (t, *J* = 7.3 Hz, 2H), 7.35 (d, *J* = 8.7 Hz, 1H), 7.30 – 7.21 (m, 4H), 2.37 (s, 3H); ^{13}C NMR (100 MHz, $CDCl_3$) δ 190.2, 146.6, 138.6, 137.6, 137.0, 134.4, 132.4, 130.5, 129.0, 128.6, 126.9, 126.6, 124.1, 123.1, 122.7, 122.0, 121.4, 112.7, 108.7, 21.7; IR (neat): 3314 (br), 2924, 1712, 1638, 1596, 1500, 1442, 1377, 1284, 1255, 1176, 1113, 1086, 935, 666, 602; HRESIMS Calcd for $[C_{24}H_{17}ClN_2NaO_3S]^+$ (M + Na^+) 471.0541, found 471.0555.

(5-bromo-1-tosyl-1,8-dihydropyrrolo[2,3-*b*]indol-3-yl)(phenyl)methanone (3n)

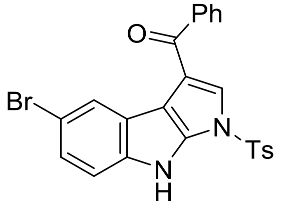

3n

Compound **3n** was prepared in 71% yield (69.9 mg) according to the general procedure (Table 3, entry 14). Pale yellow oil. ^{1}H NMR (400 MHz, $CDCl_3$) δ 8.86 (s, 1H), 8.21 (d, *J*

= 1.3 Hz, 1H), 7.88 (d, *J* = 7.2 Hz, 2H), 7.78 (d, *J* = 8.3 Hz, 2H), 7.62 (t, *J* = 7.4 Hz, 1H), 7.52 (t, *J* = 7.6 Hz, 2H), 7.37 – 7.26 (m, 5H), 2.37 (s, 3H); ^{13}C NMR (125 MHz, $CDCl_3$) δ 190.2, 146.6, 138.5, 137.8, 136.8, 134.3, 132.4, 130.5, 129.0, 128.7, 126.9, 125.7, 124.4, 124.1, 122.7, 122.5, 114.0, 113.2, 108.6, 21.7; IR (neat): 3376 (br), 2924, 2292, 1731, 1638, 1500, 1438, 1376, 1284, 1256, 1176, 1143, 1085, 927, 601; HRESIMS Calcd for $[C_{24}H_{17}BrN_2NaO_3S]^+$ (M + Na^+) 515.0035, found 515.0050.

(6-chloro-1-tosyl-1,8-dihydropyrrolo[2,3-*b*]indol-3-yl)(phenyl)methanone (3o)

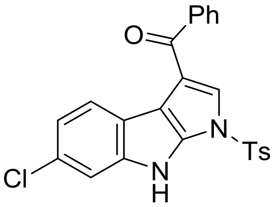

3o

Compound **3o** was prepared in 58% yield (52.0 mg) according to the general procedure (Table 3, entry 15). Pale yellow oil. ^{1}H NMR (500 MHz, $CDCl_3$) δ 8.83 (s, 1H), 7.96 (d, *J* = 8.5 Hz, 1H), 7.88 (d, *J* = 7.3 Hz, 2H), 7.79 (d, *J* = 8.3 Hz, 2H), 7.62 (t, *J* = 7.4 Hz, 1H), 7.52 (t, *J* = 7.6 Hz, 2H), 7.44 (s, 1H), 7.29 – 7.19 (m, 4H), 7.17 (dd, *J* = 8.4, 1.3 Hz, 1H), 2.38 (s, 3H); ^{13}C NMR (125 MHz, $CDCl_3$) δ 190.3, 146.6, 139.5, 138.6, 136.5, 134.4, 132.4, 130.5, 129.0, 128.6, 128.5, 126.9, 124.1, 122.7, 122.5, 121.6, 119.5, 111.9, 108.9, 21.7; IR (neat): 3489 (br), 2924, 1633, 1539, 1498, 1378, 1293, 1175, 1112, 1007, 923, 671; HRESIMS Calcd for $[C_{24}H_{17}ClN_2NaO_3S]^+$ (M + Na^+) 471.0541, found 471.0550.

phenyl(1-(phenylsulfonyl)-1,8-dihydropyrrolo[2,3-*b*]indol-3-yl)methanone (3p)

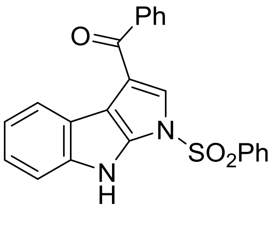

3p

Compound **3p** was prepared in 84% yield (64.0 mg) according to the general procedure (Table 3, entry 16). Pale yellow oil. ^{1}H NMR (400 MHz, $CDCl_3$) δ 8.77 (s, 1H), 8.01 (d, *J* = 7.8 Hz, 1H), 7.92 – 7.88 (m, 4H), 7.62 (t, *J* = 7.4 Hz, 2H), 7.53 – 7.43 (m, 5H), 7.33 – 7.17 (m, 3H); ^{13}C NMR (125 MHz, $CDCl_3$) δ 190.4, 139.3, 138.7, 137.6, 136.3, 134.9, 132.3, 129.8, 129.1, 128.6, 126.8, 123.8, 123.1, 123.0, 121.7, 121.0, 120.9, 111.9, 109.2; IR (neat): 3433 (br), 1727, 1632, 1500, 1483, 1448, 1380, 1291, 1178, 1142, 1110, 1007, 923, 725, 614; HRESIMS Calcd for $[C_{23}H_{16}N_2NaO_3S]^+$ (M + Na^+) 423.0774, found 423.0775.

(1-((4-bromophenyl)sulfonyl)-1,8-dihydropyrrolo[2,3-*b*]indol-3yl)(phenyl)methanone (3q)

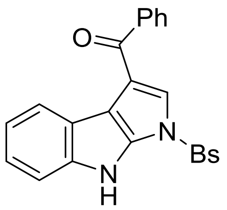

3q

Compound **3q** was prepared in 86% yield (82.2 mg) according to the general procedure (Table 3, entry 17). Yellow solid (mp 194-196 ℃). ^{1}H NMR (500 MHz, $CDCl_3$) δ 8.78 (s, 1H), 8.00 (d, *J* = 7.9 Hz, 1H), 7.90 (d, *J* = 7.4 Hz, 2H), 7.74 (d, *J* = 8.6 Hz, 2H), 7.63 – 7.58 (m, 3H), 7.52 (t, *J* = 7.6 Hz, 2H), 7.46 (d, *J* = 8.1 Hz, 1H), 7.30 – 7.27 (m, 2H), 7.21 (t, *J* = 7.5 Hz, 1H); ^{13}C NMR (125 MHz, $CDCl_3$) δ 190.3, 139.3, 138.6, 136.4, 136.2, 133.2, 132.4, 130.4, 129.1, 128.6, 128.2, 123.6, 123.5, 123.2, 121.7, 121.2, 120.9, 111.9, 109.5; IR (neat): 3388 (br), 2923, 1731, 1639, 1573, 1501, 1484, 1391, 1292, 1186, 1143, 1007, 924, 744, 621; HRESIMS Calcd for $[C_{23}H_{15}BrN_2NaO_3S]^+$ (M + Na^+) 500.9879, found 500.9898.

(1-(methylsulfonyl)-1,8-dihydropyrrolo[2,3-*b*]indol-3-yl)(phenyl)methanone (3r)

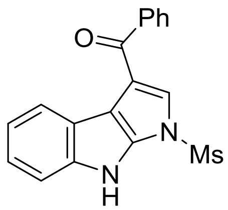

3r

Compound **3r** was prepared in 56% yield (37.9 mg) according to the general procedure (Table 3, entry 18). Pale yellow oil. ^{1}H NMR (400 MHz, $CDCl_3$) δ 8.61 (s, 1H), 8.07 (d, *J* = 7.7 Hz, 1H), 7.98 – 7.92 (m, 2H), 7.66 – 7.60 (m, 1H), 7.53 (t, *J* = 7.5 Hz, 2H), 7.44 (d, *J* = 7.9 Hz, 1H), 7.33 – 7.28 (m, 2H), 7.26 – 7.21 (m, 1H), 3.26 (s, 3H); ^{13}C NMR (125 MHz, $CDCl_3$) δ 190.4, 139.3, 138.7, 136.1, 132.4, 129.0, 128.6, 123.6, 123.2, 121.8, 121.2, 121.0, 111.9, 109.2, 42.4; IR (neat): 3395 (br), 2924, 1769, 1633, 1500, 1485, 1451, 1371, 1292, 1246, 1179, 1141, 1007, 964, 706; HRESIMS Calcd for $[C_{18}H_{14}N_2NaO_3S]^+$ (M + Na^+) 361.0617, found 361.0616.

4-phenyl-2,3-dihydro-1*H*-pyrrolo[3,4-*c*]quinolin-1-one (5a)

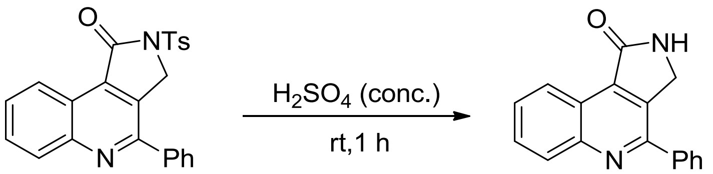

Supplementary Figure 91. Synthesis of compound **5a**.

H_2SO_4 (98%, 8.0 mL) was added to a flame dried *Schlenk*-flask containing **2a** (0.2 mmol, 82.8 mg) at room temperature. The reaction mixture was stirred at room temperature and the progress of the reaction was monitored by TLC. The reaction took 1 h. Upon completion, the solution was diluted with 20 mL EtOAc and neutralized with saturated Na_2CO_3 at 0 ^{o}C. The aqueous phase was extracted with EtOAc three times and the combined organic phase was dried with Mg_2SO_4. The solvent was removed under reduced pressure and the residue was purified by chromatography on silica gel (eluent: petroleum ether/ethyl acetate) to afford the desired product **5a** (38.5 mg, 74% yield) White solid (mp 231-233 ℃). ^{1}H NMR (400 MHz, $CDCl_3$) δ 9.14 (dd, *J* = 8.3, 0.9 Hz,

1H), 8.29 (d, *J* = 8.2 Hz, 1H), 7.91 (dd, *J* = 8.1, 1.5 Hz, 2H), 7.85 – 7.81 (m, 1H), 7.73 – 7.69 (m, 1H), 7.59 – 7.52 (m, 3H), 7.06 (s, 1H), 4.76 (d, *J* = 0.9 Hz, 2H); ^{13}C NMR (125 MHz, $CDCl_3$) δ 170.9, 154.1, 148.2, 138.4, 135.7, 135.1, 130.2, 129.7, 129.6, 129.0, 128.3, 128.1, 123.5, 122.8, 45.5; IR (neat): 3442 (br), 2915, 2846, 2088, 1700, 1633, 1492, 1363, 1178, 1074, 906, 769; HRESIMS Calcd for $[C_{17}H_{12}N_2NaO]^+$ (M + Na^+) 283.0842, found 283.0846.

2-ethyl-4-phenyl-2,3-dihydro-1*H*-pyrrolo[3,4-*c*]quinolin-1-one (5b)

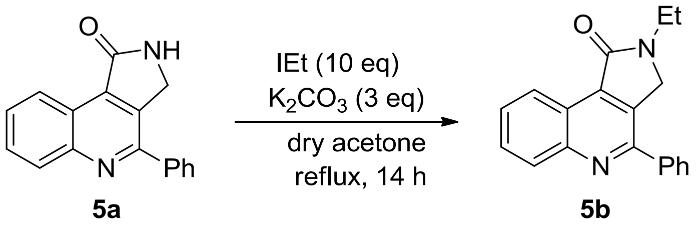

Supplementary Figure 92. Synthesis of compound **5b**.

Potassium carbonate (0.6 mmol, 82.8 mg) and ethyl iodide (2 mmol, 308 mg) were added in this order to the **5a** (0.20 mmol, 52 mg) in dry acetone (4.0 mL) at room temperature. The reaction mixture was refluxed for 14 h under the nitrogen. The reaction mixture was colled and filtered. The filtrate was evaporated and the residue was purified by chromatography on silica gel (eluent: hexanes/ ethyl acetate) to afford the desired product **5b** (37.3 mg, 65% yield, white solid); This compound is known and the spectroscopic data match those reported[31]. ^{1}H NMR (400 MHz, $CDCl_3$) δ 9.17 (dd, *J* = 8.3, 0.9 Hz, 1H), 8.26 (d, *J* = 8.1 Hz, 1H), 7.94 – 7.88 (m, 2H), 7.84 – 7.80 (m, 1H), 7.72 – 7.68 (m, 1H), 7.60 – 7.53 (m, 3H), 4.69 (s, 2H), 3.79 (q, *J* = 7.3 Hz, 2H), 1.35 (t, *J* = 7.3 Hz, 3H); ^{13}C NMR (125 MHz, $CDCl_3$) δ 167.7, 153.6, 148.4, 138.6, 136.3, 133.2, 130.0, 129.6, 129.5, 129,0, 128.3, 127.9, 123.6, 122.8, 49.5, 37.2, 13.8; HRESIMS Calcd for $[C_{19}H_{16}N_2NaO]^+$ (M + Na^+) 311.1155, found 311.1173.

4-phenyl-1*H*-pyrrolo[3,4-*c*]quinoline-1,3(2*H*)-dione (5cc)

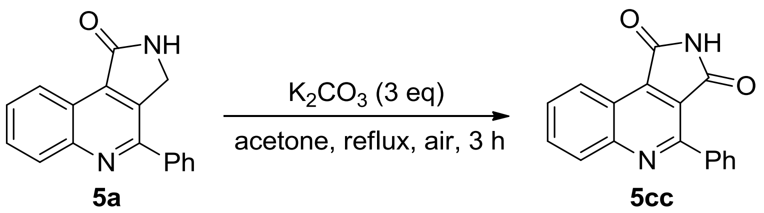

Supplementary Figure 93. Synthesis of compound **5cc**.

Potassium carbonate (0.6 mmol, 82.8 mg) were added in this order to the **5a** (0.2 mmol, 52.0 mg) in acetone (4.0 mL) at room temperature. The reaction mixture was refluxed in air for 3 h. The reaction mixture was colled and filtered. The filtrate was evaporated and the residue was purified by chromatography on silica gel (eluent: hexanes/ ethyl acetate) to afford the desired product **5cc** (40.0 mg, 71% yield, yellow solid) [32,33]. This compound is known and the spectroscopic data match those reported[34]. ^{1}H NMR (400 MHz, DMSO) δ 11.60 (s, 1H), 8.80 (dd, J = 8.3, 0.7 Hz, 1H), 8.20 (d, J = 8.5 Hz, 1H), 8.02 – 7.98 (m, 1H), 7.95 – 7.93 (m, 2H), 7.87 – 7.83 (m, 1H), 7.57 – 7.51 (m, 3H); ^{13}C NMR (125 MHz, DMSO) δ 169.2, 168.4, 154.4, 150.5, 138,0, 136.7, 132.8, 130.1, 129.6, 129.5, 127.7, 124.3, 122.5, 120.4; HRESIMS Calcd for $[C_{17}H_{10}N_2NaO_2]^+$ (M + Na^+) 297.0634, found 297.0639.

2,4-diphenyl-1*H*-pyrrolo[3,4-*c*]quinoline-1,3(2*H*)-dione (5c)

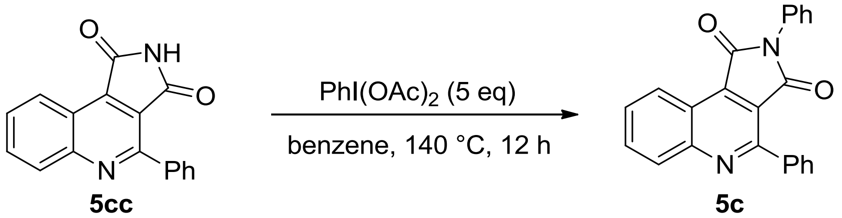

Supplementary Figure 94. Synthesis of compound **5c**.

$PhI(OAc)_2$ (1.0 mmol, 322.0 mg) were added in this order to the **5cc** (0.2 mmol, 54.8 mg) in benzene (4.0 mL) at room temperature. The reaction mixture was stirred at 140 ^{o}C for 12 h. The mixture was then concentrated and the residue was purified by chromatography on silica gel (eluent: hexanes/ ethyl acetate) to afford the desired product **5c** (48.7 mg,

70% yield, yellow solid)[35]. This compound is known and the spectroscopic data match those reported[36]. ^{1}H NMR (500 MHz, $CDCl_3$) δ 9.00 (dd, *J* = 8.4, 0.6 Hz, 1H), 8.30 (d, *J* = 8.6 Hz, 1H), 8.03 – 7.93 (m, 3H), 7.81 – 7.78 (m, 1H), 7.57 – 7.40 (m, 8H); ^{13}C NMR (125 MHz, $CDCl_3$) δ 167.0, 166.5, 155.4, 151.9, 137.1, 136.6, 133.2, 131.3, 130.2, 130.1, 130.0, 129.8, 129.2, 128.4, 128.1, 126.8, 125.1, 120.9, 120.7; HRESIMS Calcd for $[C_{23}H_{14}N_2NaO_2]^+$ (M + Na^+) 373.0947, found 373.0948.

8-(morpholinosulfonyl)-4-phenyl-2,3-dihydro-1*H*-pyrrolo[3,4-*c*]quinolin-1-one (5dd)

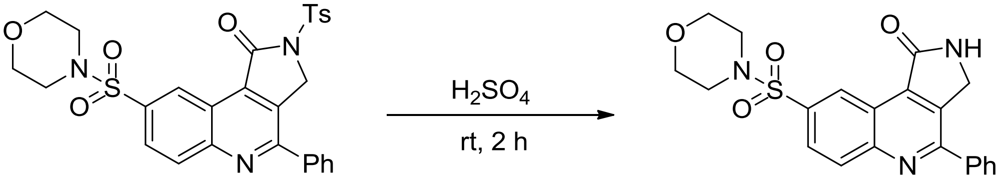

2u **5dd**

Supplementary Figure 95. Synthesis of compound **5dd**.

H_2SO_4 (98%, 8.0 mL) was added to a flame dried *Schlenk*-flask containing **2u** (0.2 mmol, 112.6 mg) at room temperature. The reaction mixture was stirred at room temperature and the progress of the reaction was monitored by TLC. The reaction took 2 h. Upon completion, the solution was diluted with 20 mL EtOAc and neutralized with saturated Na_2CO_3 at 0 ^{o}C. The aqueous phase was extracted with EtOAc three times and the combined organic phase was dried with Mg_2SO_4. The solvent was removed under reduced pressure and the residue was purified by chromatography on silica gel (eluent: petroleum ether/ethyl acetate) to afford the desired product **5dd** (45.6 mg, 56% yield). Yellow solid (mp 246-248 ℃). ^{1}H NMR (500 MHz, DMSO) δ 9.47 (d, *J* = 2.0 Hz, 1H), 9.45 (s, 1H), 8.44 (d, *J* = 8.9 Hz, 1H), 8.14 – 8.07 (m, 3H), 7.64 – 7.59 (m, 3H), 4.88 (s, 2H), 3.65 (t, 4H), 2.99 (t, 4H); ^{13}C NMR (125 MHz, DMSO) δ 169.0, 156.4, 148.3, 138.4, 137.4, 136.5, 133.4, 130.8, 130.3, 128.9, 128.7, 127.0, 123.7, 121.6, 65.3, 45.9, 45.5; IR (neat): 3445 (br), 2925, 2127, 1732, 1644, 1594, 1498, 1366, 1254, 1170, 1088, 985, 774, 663; HRESIMS Calcd for $[C_{21}H_{19}N_3NaO_4S]^+$ (M + Na^+) 432.0988, found 432.0994.

2-(8-(morpholinosulfonyl)-1,3-dioxo-4-phenyl-1*H*-pyrrolo[3,4-*c*]quinolin-2(3*H*)-yl)ethyl acetate (5d)

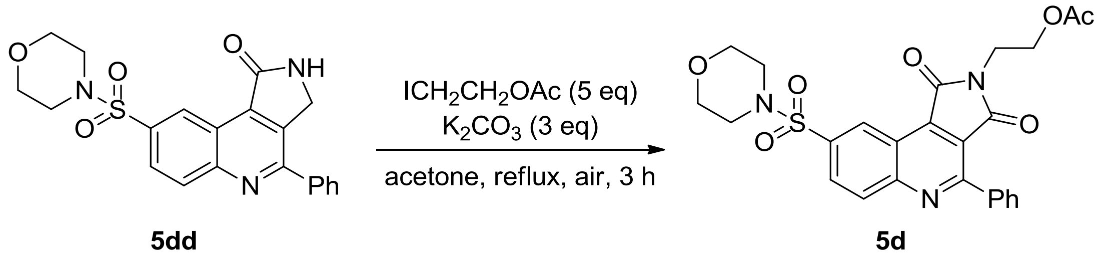

Supplementary Figure 96. Synthesis of compound **5d**.

Potassium carbonate (0.3 mmol, 41.4 mg) and ICH_2CH_2OAc (0.5 mmol, 107 mg) were added in this order to the **5dd** (0.1 mmol, 40.9 mg) in acetone (2.0 mL) at room temperature. The reaction mixture was refluxed in air for 3 h. The reaction mixture was colled and filtered. The filtrate was evaporated and the residue was purified by chromatography on silica gel (eluent: hexanes/ ethyl acetate) to afford the desired product **5d** (31.9 mg, 67% yield). This compound is known and the spectroscopic data match those reported[37]. ^1H NMR (400 MHz, $CDCl_3$) δ 9.35 (d, *J* = 1.6 Hz, 1H), 8.41 (d, *J* = 9.0 Hz, 1H), 8.19 (dd, *J* = 9.0, 2.1 Hz, 1H), 8.03 – 7.98 (m, 2H), 7.61 – 7.56 (m, 3H), 4.38 (t, *J* = 5.2 Hz, 2H), 4.04 (t, *J* = 5.2 Hz, 2H), 3.79 (t, *J* = 4.8 Hz, 4H), 3.16 (t, *J* = 4.8 Hz, 4H), 2.03 (s, 3H); ^{13}C NMR (150 MHz, $CDCl_3$) δ 170.9, 167.0, 166.6, 157.9, 152.3, 138.6, 136.6, 135.8, 131.5, 130.8, 130.3, 130.0, 128.3, 125.9, 122.5, 119.8, 66.1, 61.3, 46.1, 37.7, 20.8; HRESIMS Calcd for $[C_{25}H_{23}N_3NaO_7S]^+$ (M + Na^+) 532.1149, found 532.1160.

(1,8-dihydropyrrolo[2,3-*b*]indol-3-yl)(phenyl)methanone (6a)

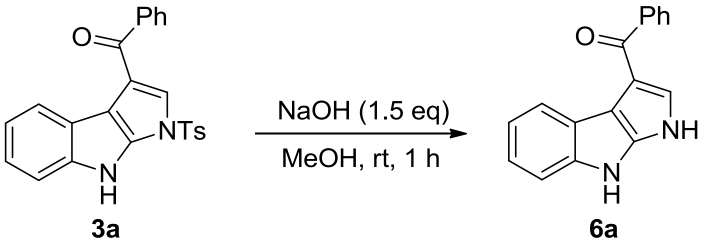

Supplementary Figure 97. Synthesis of compound **6a**.

NaOH (0.3 mmol, 12.0 mg) was added in this order to the **3a** (0.2 mmol, 82.8 mg) in MeOH (4.0 mL) at room temperature. The reaction mixture was stirred at room temperature and the progress of the reaction was monitored by TLC. The reaction took 1 h. Upon completion, the mixture was then concentrated and the residue was purified by chromatography on silica gel (eluent: hexanes/ ethyl acetate) to afford the desired product **6a** (38.5 mg, 74% yield). Yellow solid (mp 135-137 ℃). ^{1}H NMR (400 MHz, acetone) δ 10.91 (s, 1H), 10.17 (s, 1H), 8.18 (dd, *J* = 6.5, 2.4 Hz, 1H), 7.90 (dd, *J* = 8.1, 1.3 Hz, 2H), 7.60 – 7.50 (m, 3H), 7.40 (dd, *J* = 6.6, 2.1 Hz, 1H), 7.27 (d, *J* = 2.5 Hz, 1H), 7.13 – 7.07 (m, 2H); ^{13}C NMR (150 MHz, acetone) δ 190.6, 141.9, 141.1, 140.2, 131.6, 129.4, 129.0, 126.7, 122.9, 121.7, 121.6, 119.7, 118.7, 112.2, 108.2; IR (neat): 3388 (br), 2923, 2852, 1606, 1573, 1483, 1456, 1357, 1294, 1175, 1096, 888, 732; HRESIMS Calcd for $[C_{17}H_{12}N_2NaO]^+$ (M + Na$^+$) 283.0842, found 283.0844.

phenyl(1-tosyl-1,8-dihydropyrrolo[2,3-*b*]indol-3-yl)methanol (6b)

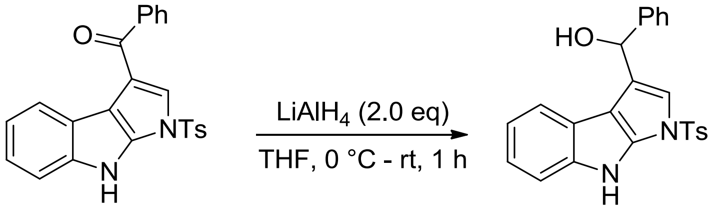

Supplementary Figure 98. Synthesis of compound **6b**.

$LiAlH_4$ (0.4 mmol, 15.2 mg) was added in this order to the **3a** (0.2 mmol, 82.8 mg) in dry THF (4.0 mL) at 0 ^{o}C. The reaction mixture was stirred at room temperature and the progress of the reaction was monitored by TLC. The reaction took 1 h. Upon completion, the mixture was then concentrated and the residue was purified by chromatography on silica gel (eluent: hexanes/ ethyl acetate) to afford the desired product **6b** (58.2 mg, 70% yield). Yellow solid (mp 97-99 ℃). ^{1}H NMR (500 MHz, $CDCl_3$) δ 8.72 (s, 1H), 7.71 (d, *J* = 8.4 Hz, 2H), 7.40 (d, *J* = 6.9 Hz, 2H), 7.37 (d, *J* = 8.2 Hz, 1H), 7.33 – 7.26 (m, 4H), 7.22 (d, *J* = 8.1 Hz, 2H), 7.15 (t, *J* = 8.3 Hz, 1H), 7.03 (t, *J* = 8.0 Hz, 1H), 6.76 (d, *J* = 1.0

Hz, 1H), 5.94 (d, J = 3.7 Hz, 1H), 2.36 (s, 3H), 2.30 (d, J = 4.2 Hz, 1H); ^{13}C NMR (125 MHz, $CDCl_3$) δ 145.4, 142.3, 138.7, 137.1, 135.0, 130.1, 128.6, 128.0, 127.4, 126.8, 126.7, 121.9, 120.8, 120.5, 120.0, 115.2, 111.8, 108.7, 71.0, 21.6; IR (neat): 3396 (br), 2923, 2853, 1725, 1596, 1535, 1452, 1370, 1235, 1118, 1085, 908, 671; HRESIMS Calcd for $[C_{24}H_{20}N_2NaO_3S]^+$ (M + Na^+) 439.1087, found 439.1103.

(8-methyl-1-tosyl-1,8-dihydropyrrolo[2,3-*b*]indol-3-yl)(phenyl)methanone (3aa)

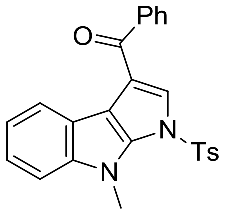

3aa

Yellow solid (mp 97-99 ℃). ^{1}H NMR (500 MHz, $CDCl_3$) δ 8.13 (d, J = 7.8 Hz, 1H), 7.93 (d, J = 7.0 Hz, 2H), 7.68 (d, J = 8.4 Hz, 2H), 7.65 – 7.61 (m, 1H), 7.56 – 7.52 (m, 3H), 7.35 – 7.31 (m, 2H), 7.27 – 7.19 (m, 3H), 4.05 (s, 3H), 2.37 (s, 3H); ^{13}C NMR (125 MHz, $CDCl_3$) δ 190.5, 146.0, 141.4, 138.8, 137.0, 135.1, 132.3, 130.4, 129.1, 128.6, 128.3, 126.8, 122.8, 121.9, 121.8, 120.5, 119.6, 111.0, 109.7, 32.2, 21.6; IR (neat): 2922, 2851, 1640, 1513, 1459, 1375, 1319, 1174, 1087, 1008, 897, 720, 665; HRESIMS Calcd for $[C_{25}H_{20}N_2NaO_3S]^+$ (M + Na^+) 451.1087, found 451.1088.

***N*-((2-azidophenyl)ethynyl)-*N*-(3-cyclohexylprop-2-yn-1-yl)-4-methylbenzenesulfonamide (1v)**

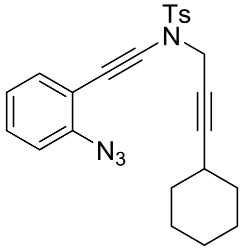

1v

Yellow oil. ^{1}H NMR (400 MHz, $CDCl_3$) δ 7.94 (d, J = 8.3 Hz, 2H), 7.36 – 7.33 (m, 3H), 7.31 – 7.27 (m, 1H), 7.10 – 7.04 (m, 2H), 4.34 (d, J = 2.1 Hz, 2H), 2.44 (s, 3H), 2.20 (s, 1H), 1.57 – 1.55 (m, 4H), 1.44 – 1.38 (m, 1H), 1.26 – 1.18 (m, 5H); ^{13}C NMR (100 MHz, $CDCl_3$) δ 144.7, 140.8, 134.5, 133.4, 129.4, 129.0, 128.3, 124.5, 118.6, 115.0, 91.4, 87.5, 71.8, 66.9, 42.4, 32.0, 28.7, 25.7, 24.4, 21.5; IR (neat): 2928, 2356, 2234, 2127, 1731, 1650, 1633, 1596, 1493, 1446, 1387, 1304, 1170, 1090, 754, 665; HRESIMS Calcd for $[C_{24}H_{24}N_4NaO_2S]^+$ (M + Na^+) 455.1512, found 455.1518.

3-(2-azidophenyl)-4-(cyclohexylidenemethyl)-1-tosyl-1,5-dihydro-2*H*-pyrrol-2-one (2va)

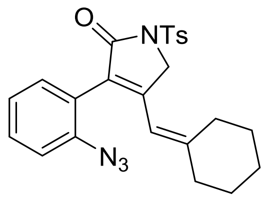

2va

Compound **2va** was prepared in 66% yield (59.1 mg) according to the general procedure. Yellow oil. ^{1}H NMR (400 MHz, $CDCl_3$) δ 8.00 (d, J = 8.4 Hz, 2H), 7.40 – 7.35 (m, 1H), 7.32 (d, J = 8.0 Hz, 2H), 7.17 (d, J = 7.9 Hz, 1H), 7.14 – 7.12 (m, 2H), 5.79 (s, 1H), 4.75 (s, 2H), 2.42 (s, 3H), 2.28 – 2.23 (m, 2H), 2.20 – 2.10 (m, 2H), 1.64 – 1.49 (m, 6H); ^{13}C NMR (125 MHz, $CDCl_3$) δ 167.4, 154.3, 151.4, 144.9, 138.9, 135.5, 131.9, 130.0, 129.7, 129.0, 128.2, 124.7, 122.3, 118.8, 114.3, 52.2, 38.9, 31.7, 28.6, 27.8, 26.0, 21.6; IR (neat): 2928, 2854, 2125, 1714, 1633, 1465, 1446, 1360, 1159, 1092, 665, 583; HRESIMS Calcd for $[C_{24}H_{24}N_4NaO_3S]^+$ (M + Na^+) 471.1461, found 471.1466.

Supplementary References

1. Gaussian 09, Revision D.01, M. J. Frisch, G. W. Trucks, H. B. Schlegel, G. E. Scuseria, M. A. Robb, J. R. Cheeseman, G. Scalmani, V. Barone, B. Mennucci, G. A. Petersson, H. Nakatsuji, M. Caricato, X. Li, H. P. Hratchian, A. F. Izmaylov, J. Bloino, G. Zheng, J. L. Sonnenberg, M. Hada, M. Ehara, K. Toyota, R. Fukuda, J.

Hasegawa, M. Ishida, T. Nakajima, Y. Honda, O. Kitao, H. Nakai, T. Vreven, J. A. Montgomery, Jr., J. E. Peralta, F. Ogliaro, M. Bearpark, J. J. Heyd, E. Brothers, K. N. Kudin, V. N. Staroverov, R. Kobayashi, J. Normand, K. Raghavachari, A. Rendell, J. C. Burant, S. S. Iyengar, J. Tomasi, M. Cossi, N. Rega, J. M. Millam, M. Klene, J. E. Knox, J. B. Cross, V. Bakken, C. Adamo, J. Jaramillo, R. Gomperts, R. E. Stratmann, O. Yazyev, A. J. Austin, R. Cammi, C. Pomelli, J. W. Ochterski, R. L. Martin, K. Morokuma, V. G. Zakrzewski, G. A. Voth, P. Salvador, J. J. Dannenberg, S. Dapprich, A. D. Daniels, O. Farkas, J. B. Foresman, J. V. Ortiz, J. Cioslowski, D. J. Fox, Gaussian, Inc., Wallingford CT, **2013**.

2. Y. Zhao, D. G. Truhlar, *Theor. Chem. Acc.* **2008**, *120*, 215.
3. R. Ditchfield, W. J. Hehre, J. A. Pople, *J. Chem. Phys.* **1971**, *54*, 724.
4. W. J. Hehre, R. Ditchfield, J. A. Pople, *J. Chem. Phys.* **1972**, *56*, 2257.
5. P. C. Hariharan, J. A. Pople, *Theo. Chim. Acta.* **1973**, *28*, 213.
6. P. C. Hariharan, J. A. Pople, *Mol. Phys.* **1974**, *27*, 209.
7. M. S. Gordon, *Chem. Phys. Lett.* **1980**, *76*, 163.
8. D. Andrae, U. Häussermann, M. Dolg, H. Stoll, H. Preuss, *Theor. Chim. Acta.* **1990**, *77*, 123.
9. W.-B. Shen, X.-Y. Xiao, Q. Sun, B. Zhou, X.-Q. Zhu, J.-Z. Yan, X. Lu, L.-W. Ye, *Angew. Chem. Int. Ed.* **2017**, *56*, 605.
10. C. Shu, Y.-H. Wang, B. Zhou, X.-L. Li, Y.-F. Ping, X. Lu, L.-W. Ye, *J. Am. Chem. Soc.* **2015**, *137*, 9567.
11. A.-H. Zhou, Q. He, C. Shu, Y.-F. Yu, S. Liu, T. Zhao, W. Zhang, X. Lu, L.-W. Ye, *Chem. Sci.* **2015**, *6*, 1265.
12. C. Shu, R.-F. Liu, S. Liu, J.-Q. Li, Y.-F. Yu, Q. He, X. Lu, L.-W. Ye, *Chem. Asian J.* **2015**, *10*, 91.
13. P. J. Hay, W. R. Wadt, *J. Chem. Phys.* **1985**, *82*, 270.
14. C. Gonzalez, H. B. Schlegel, *J. Phsy. Chem.* **1990**, *94*, 5523.
15. A. V. Marenich, C. J. Cramer, D. G. Truhlar, *J. Phys. Chem. B* **2009**, *113*, 6378.
16. B. Lu, Y. Li, Y. Wang, D. H. Aue, Y. Luo, L. Zhang, *J. Am. Chem. Soc.*, **2013**, *135*, 8512.

17. Y. Wang, A. Yepremyan, S. Ghorai, R. Todd, D. H. Aue, L. Zhang, *Angew. Chem. Int. Ed.* **2013**, *52*, 7795.
18. A. T. Tran, P. Liu, K. N. Houk, K. M. Nicholas, *J. Org. Chem.* **2014**, *79*, 5617.
19. N. Yoshikai, K. Miura, E. Nakamura, *Adv. Synth. Catal.* **2009**, *351*, 1014.
20. J. Comas-Barceló, R. S. Foster, B. Fiser, E. Gomez-Bengoa, J. P. A. Harrity, *Chem. Eur. J.* **2014**, *20*, 1.
21. S. Özcubukcu, E. Ozkal, C, Jimeno, M. A. Pericàs, *Org. Lett.* **2009**, *11*, 4680.
22. C. Jarava-Barrera, A. Parra, A. López, F. Cruz-Acosta, D. Collado-Sanz, D. J. Cárdenas, M. Tortosa, *ACS Catal.* **2016**, *6*, 442.
23. D. Cantillo, M. Ávalos, R. Babiano, P. Cintas, J. L. Jiménez, J. C. Palacios, *Org. Biomol. Chem.* **2011**, *9*, 2952.
24. M. E. Bunnage, S. G. Davies, P. M. Roberts, A. D. Smith, J. M. Withey, *Org. Biomol. Chem.* **2004**, *2*, 2763.
25. M.-M. Wang, X.-S. Ning, J.-P. Qu, Y.-B. Kang, *ACS Catal.* **2017**, *7*, 4000.
26. X. Jie, Y. Shang, X. Zhang, W. Su, *J. Am. Chem. Soc.* **2016**, *138*, 5623.
27. T. Miura, Q. Zhao, Y. Funakoshi, M. Murakami, *Heterocycles* **2015**, *91*, 1579.
28. B. Prabagar, S. Nayak, R. Prasad, A. K. Sahoo, *Org. Lett.* **2016**, *18*, 3066.
29. C. Shu, M.-Q. Liu, S.-S. Wang, L. Li, L.-W. Ye, *J. Org. Chem.* **2013**, *78*, 3292.
30. M. A. Ilies, D. Vullo, J. Pastorek, A. Scozzafava, M. Ilies, M. T. Caproiu, S. Pastorekova, C. T. Supuran, *J. Med. Chem.* **2003**, *46*, 2187.
31. A. N. Boa, S. P. Canavan, P. R. Hirst, C. Ramsey, A. M. W. Stead, G. A. McConkey, *Bioorg. Med. Chem.* **2005**, *13*, 1945.
32. J. H. Lee, S. R. Byeon, Y. S. Kim, S. J. Lim, S. J. Oh, D. H. Moon, K, H. Yoo, B. Y. Chung, D. J. Kim, *Bioorg. Med. Chem. Lett.* **2008**, *18*, 5701.
33. J. H. Shah, B. P. Conner, G. M. Jr. Swartz, K. A. Hunsucker, J. Rougas, R. D'Amato, V. Pribluda, A. Treston, PCT Int. Appl. WO 2003014315A2, **2003**.
34. S. Mollin, S. Blanck, K. Harms, E. Meggers, *Inorg. Chim. Act*, **2012**, *393*, 261.
35. H. J. Kim, J. Kim, S. H. Cho, S. Chang, *J. Am. Chem. Soc.* **2011**, *133*, 16382.
36. L. Xia, A. Idhayadhulla, Y. R. Lee, S. H. Kim, Y.-J. Wee, *ACS Comb. Sci.* **2014**, *16*, 333.

37. D. V. Kravchenko, Y. A. Kuzovkova, V. M. Kysil, S. E. Tkachenko, S. Maliarchouk, I. M. Okun, K. V. Balakin, A. V. Ivachtchenko, *J. Med. Chem.* **2005**, *48*, 3680.
