# Supplementary material for: Divergent synthesis of N-heterocycles via controllable cyclization of azido-diynes catalyzed by copper and gold
Source: Nat Commun. 2017 Nov 23;8:1748. doi: 10.1038/s41467-017-01853-1 (PMC5701061; doi:10.1038/s41467-017-01853-1)
Supplement: Supplementary file 3 — Description of Additional Supplementary Files [file 41467_2017_1853_MOESM3_ESM.pdf]

Description of Additional Supplementary Files

File Name: Supplementary Data 1
Description: Molecular Geometries and Energies.
